# Nickel‐Catalyzed Hydro‐ and Deutero‐dehalogenations of (Hetero)Aryl Halides under Aqueous Micellar Catalysis Conditions

**DOI:** 10.1002/cssc.202500043

**Published:** 2025-04-24

**Authors:** Monica S. Lopez Lemus, Rahul D. Kavthe, Rohan M. Thomas, Max Baumann, Karthik S. Iyer, Bruce H. Lipshutz

**Affiliations:** ^1^ Department of Chemistry and Biochemistry University of California Santa Barbara CA 93106 USA

**Keywords:** nickel catalysis, hydrodehalogenation, deuterodehalogenation, aqueous micellar catalysis

## Abstract

Efficient Ni‐catalyzed hydrodehalogenations and deuterodehalogenations of aryl/heteroaryl halides are reported herein. This new technology can be used to incorporate not only hydrogen, but also deuterium into various aromatic/heteroaromatic compounds with high efficiency, using 2–6 mol % nickel in the presence of stoichiometric NaBH_4_. Over 40 examples have been successfully converted to the corresponding (hetero)arenes in excellent yields. The process is conducted under green chemistry conditions: in water enabled by designer surfactants, a medium which can be readily recycled. Minimal organic solvent, needed given the small (academic) scale of the reactions, is used for product isolation, resulting in low E‐Factors. Additionally, sterically hindered substrates are amenable, as are selected APIs that feature carbon‐fluorine bonds.

## Graphical abstract

Efficient Ni‐catalyzed hydrodehalogenations and deuterodehalogenations of aryl/heteroaryl halides are reported. This new technology applies to highly functionalized drugs and drug intermediates. The overall greenness is demonstrated via recyclability of the aqueous reaction media and associated overall low E‐Factors.

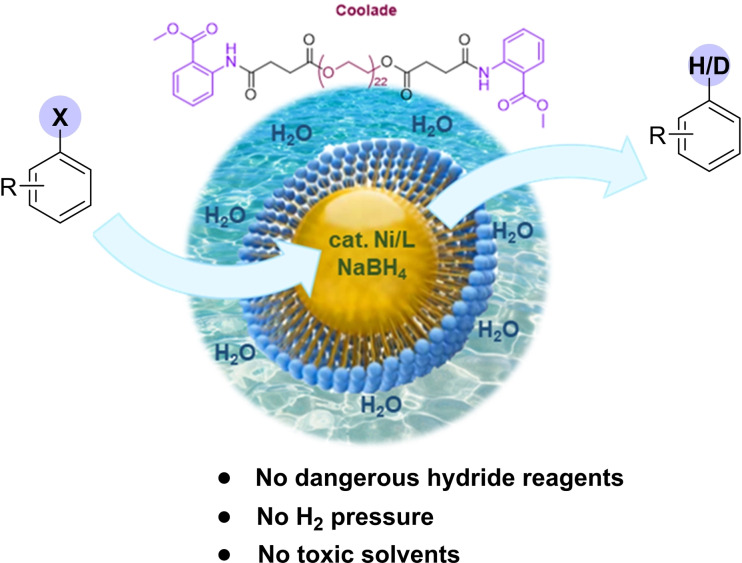

## Introduction

Hydrodehalogenation is an important organic transformation in synthesis, where a halogen on an sp^2^ carbon is formally replaced with a hydrogen atom.[^1^, ^2^, ^3^, ^4^] The techniques to accomplish this are applied broadly for industrial detoxification of environmentally hazardous chemicals, such as polychlorinated biphenyls, pesticides, flame retardants, chlorofluorocarbons, and hydrofluorocarbons.[^5^, ^6^, ^7^, ^8^, ^9^, ^10^, ^11^, ^12^, ^13^, ^14^, ^15^] Moreover, reductive removal of halogens from aryl/heteroaryl halides is used in deprotection chemistry, as an aryl/heteroaryl halogen can be employed as a blocking group in multistep syntheses without strongly influencing the electronics of the system.[^16^, ^17^, ^18^, ^19^, ^20^, ^21^, ^22^, ^23^, ^24^, ^25^, ^26^, ^27^] This strategy can also be of value as a means of introducing deuterium into medicinally useful compounds,[^28^, ^29^] since replacement of hydrogen by deuterium may improve absorption, distribution, and excretion (ADME) properties in pharma‐ceuticals.[^30^, ^31^, ^32^, ^33^, ^34^] Moreover, deuterium‐labeled compounds oftentimes find utility for purposes of elucidating questions regarding metabolism of small molecule drugs.[^35^, ^36^, ^37^]

**Figure 1:**
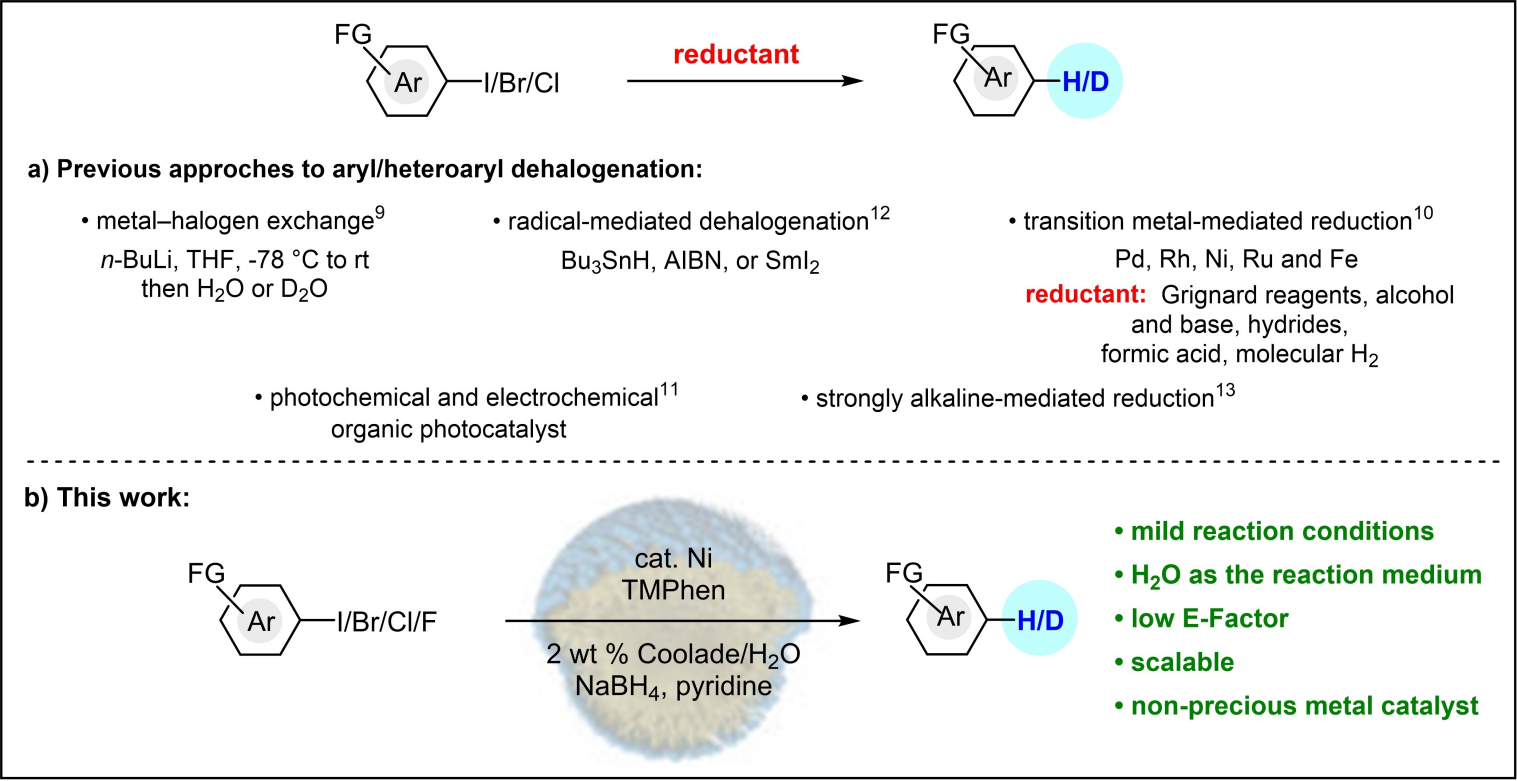
Approaches to dehalogenation and dehalogenative deuteration of aryl/heteroaryl halides.

Several methods for hydrodehalogenation have been reported and are widely used,[^38^, ^39^, ^40^, ^41^, ^42^, ^43^, ^44^, ^45^, ^46^, ^47^, ^48^, ^49^, ^50^, ^51^, ^52^, ^53^, ^54^, ^55^, ^56^, ^57^, ^58^, ^59^, ^60^, ^61^, ^62^, ^63^, ^64^, ^65^, ^66^, ^67^, ^68^, ^69^, ^70^, ^71^] such as lithium‐halogen exchange,[^32^, ^33^, ^34^, ^35^, ^36^, ^37^, ^38^, ^39^, ^40^, ^41^, ^42^, ^43^, ^44^, ^45^, ^46^, ^47^, ^48^, ^49^, ^50^, ^51^, ^52^, ^53^, ^54^, ^55^, ^56^, ^57^, ^58^, ^59^, ^60^, ^61^, ^62^, ^63^, ^64^, ^65^, ^66^, ^67^, ^68^, ^69^, ^70^, ^71^, ^72^, ^73^] transition metal‐mediated reduction,[^40^, ^41^, ^42^, ^43^, ^44^, ^45^] photochemical reduction,[^46^, ^47^, ^48^, ^49^] reductive radical dehalogenation,[^50^, ^51^, ^52^, ^53^] and strong base‐promoted transition‐metal‐free dehalogenations (Figure 1).[^54^, ^55^, ^56^, ^57^] Several of these protocols, however, may give rise to potential environmental or health risks.[^58^, ^59^] Examples include stoichiometric use of toxic reagents Bu_3_SnH^[60]^ and SmI_2,_[^58^, ^59^] highly reactive hydride reagents under cryogenic conditions (LiAlH_4_),[^61^, ^62^, ^63^, ^64^, ^65^] and use of precious metal catalysts/photocatalysts.[^40^, ^41^, ^42^, ^43^, ^44^, ^45^, ^46^, ^47^, ^48^, ^49^] Additionally, one of the major challenges and potential limitations in halogen/lithium exchange is poor functional group tolerance. Some methods are limited due to relatively strong bond dissociation energies (BDEs), *e. g*., of C−F bonds (C−F activation).[^66^, ^67^, ^68^, ^69^, ^70^] Nonetheless, there are a few reports on hydrodechlorination and hydrodefluorination, although these require high catalyst loadings[^66^, ^67^, ^68^, ^69^, ^70^] harsh reaction conditions,[^71^, ^72^, ^73^] and are usually limited to substrates containing electron‐withdrawing groups for activation purposes.[^74^, ^75^, ^76^, ^77^, ^78^, ^79^] Traditionally, Pd‐catalyzed hydrodehalogenation has proven to be both robust and high yielding, which explains why, from a historical point of view, this is among the most commonly used metal for this purpose. However, aside from the risk of availability over the next 100 years,^[80]^ Pd/C can be dangerous and calls for careful handling to prevent solvent ignition given the hydrogen atmosphere that is required for its use.^[81]^ Especially egregious is the reliance on organic solvents as reaction media, which from an environmental perspective constitutes the majority of organic waste being created.[^38^, ^39^, ^40^, ^41^, ^42^, ^43^, ^44^, ^45^, ^46^, ^47^, ^48^, ^49^, ^50^, ^51^, ^52^, ^53^, ^54^, ^55^, ^56^, ^57^, ^58^, ^59^, ^60^, ^61^, ^62^, ^63^, ^64^, ^65^, ^66^, ^67^, ^68^, ^69^, ^70^, ^71^, ^72^, ^73^] Considering their environmental impact on sustainability alone, given that most solvents are burned and hence, lead to formation of large amounts of the greenhouse gas CO_2_ thereby contributing to climate change, development of an efficient, practical, safe, and precious metal‐free process used in an aqueous medium for dehalogenation of aryl and heteroaryl halide is long overdue. Thus, we report a straightforward, cost‐ effective and environmentally responsible technology for hydrodehalogenation and dehalogenative deuteration of (hetero)aryl halides, including selected cases of fluorides, catalyzed by earth‐abundant nickel in aqueous solutions of Coolade^[82]^ in the presence of NaBH_4_ as the source of hydride.

## Results and Discussion

Catalyst screening began on a model system using chloride **1** (Table 1), with NiCl_2_ (4 mol %) chelated by monodentate triphenylphosphine (2 equiv relative to Ni; entry 1). Most of the starting material remained unreacted and only a trace amount of product was detected. Replacing nickel chloride with Ni(OAc)_2_⋅6H_2_O (entry 2) afforded dehalogenated product **2**, albeit in only 17 % yield. Changing PPh_3_ for bidentate phosphine ligand dppf did not lead to a significant improvement (entry 3). Other nickel salts (entries 4–8) also failed to give better results. While both 2,2’‐bipyridine (bpy) and TMEDA (8 mol %; entry 11) also failed to give the product in good yield, switching to 3,4,9,10‐tetramethylphenanthroline (**L1**) afforded full conversion along with an associated 94 % isolated yield, using pyridine and excess NaBH_4_ (5 equiv; entry 10). In the absence of the Ni catalyst only unreacted starting material was observed (entry 16). To determine the optimal amount of hydride, screening this variable was performed (see SI, Table S2), where the use of five equivalents of NaBH_4_ was the most effective. Lowering this amount led to poorer levels of conversion and hence, lower yields. The base was also evaluated (see SI, Table S3), where both pyridine and 2‐picoline were found to perform equally well. Adding base before or after the addition of nickel did not affect the reaction outcome.

**Table 1:**
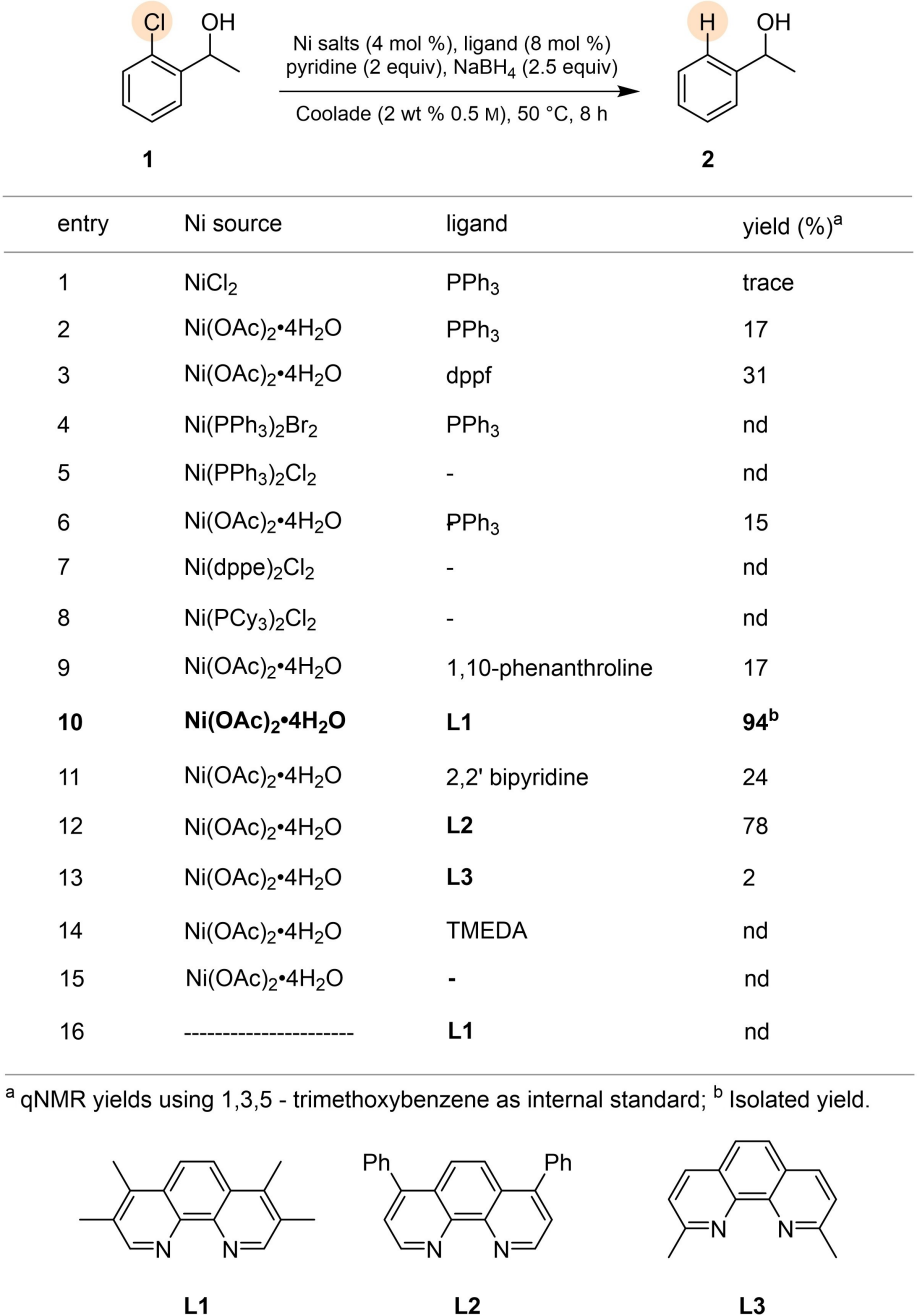
Nickel source and ligand screening.

The reaction medium also played an important role in determining the outcome of these reductions. Screening of several amphiphiles indicated that nanomicelles derived from Coolade^[82]^ led to superior results relative to those obtained using Brij‐30, TPGS‐750‐M, Savie, and pure water (Table 2).[^83^, ^84^] Although, water (entry 5) by itself allows for this reaction toÐproceed with high efficiency, Coolade was ultimately chosen for use withÐmore functionalized substrates. Using Coolade, it aidedÐwith solubility in addition to the option of using co‐solvent if needed for solubilization of highly crystalline substrates. For instance, use of Coolade, a low foaming surfactant, in the presence of 10 % (v/v), THF afforded similar results (entry 6).

**Table 2:**
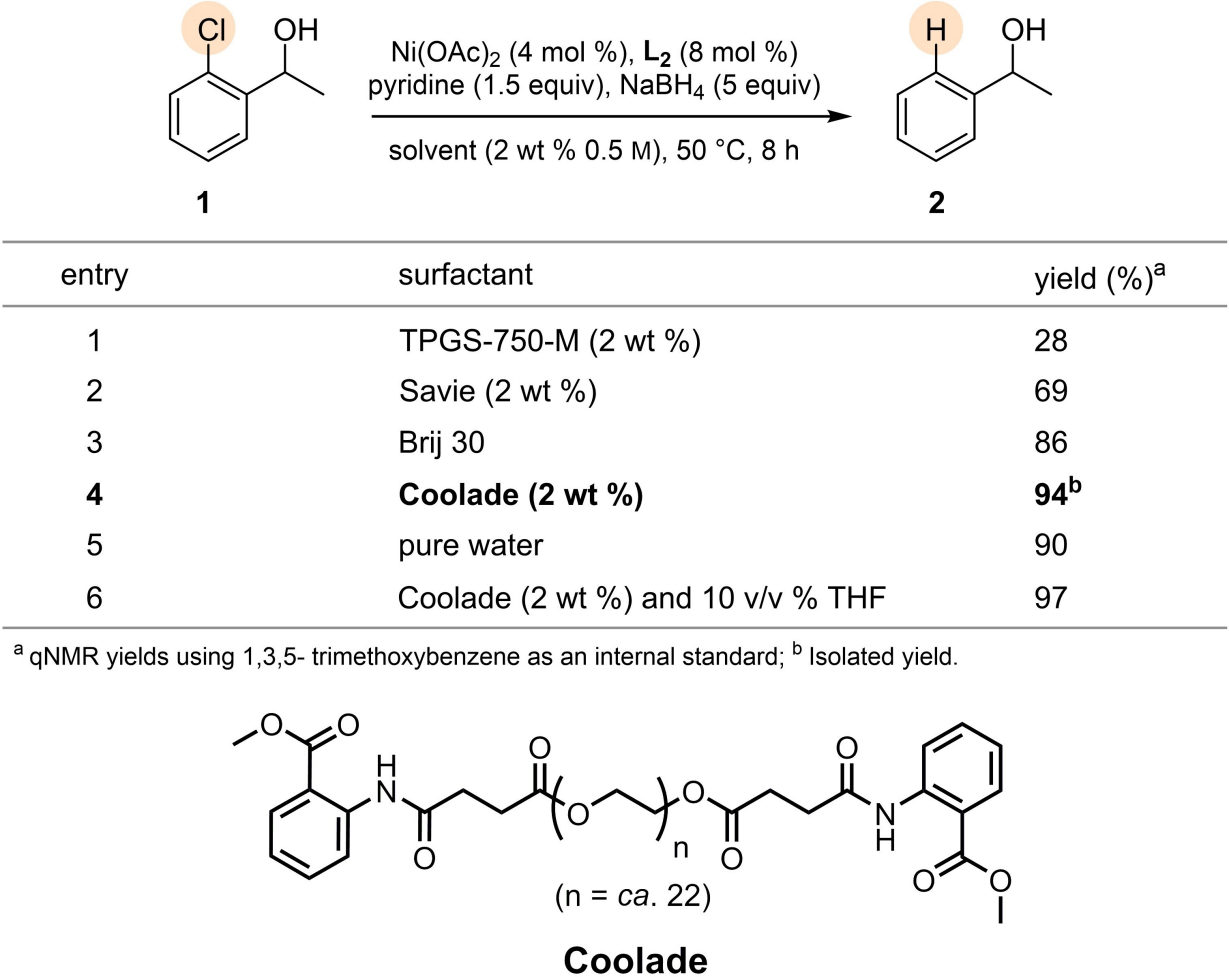
Surfactant screen.

**Figure 2:**
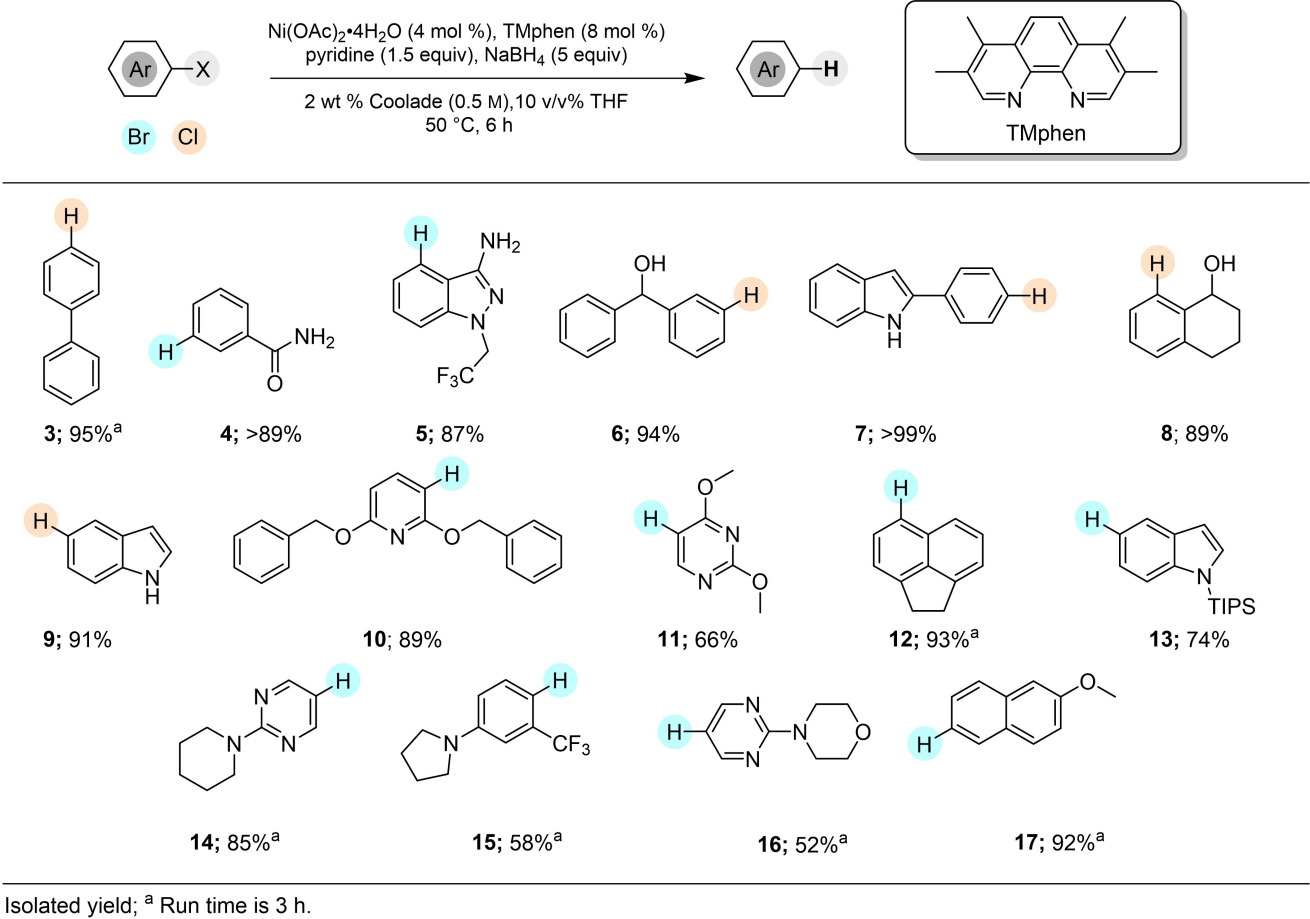
Substrate scope consisting of aryl chlorides and bromides.

The generality of this Ni‐catalyzed hydrodehalogenation was examined next (Figure 2). Several aryl/heteroaryl chlorides and bromides undergo reductive dehalogenation to provide the desired products in moderate‐to‐high yields. Many common, yet important, heterocyclic scaffolds, including indole (**7, 9, 13**), and pyrimidine (**11, 14, 16**), provided the dehalogenated heterocycles in good yields.

**Figure 3:**
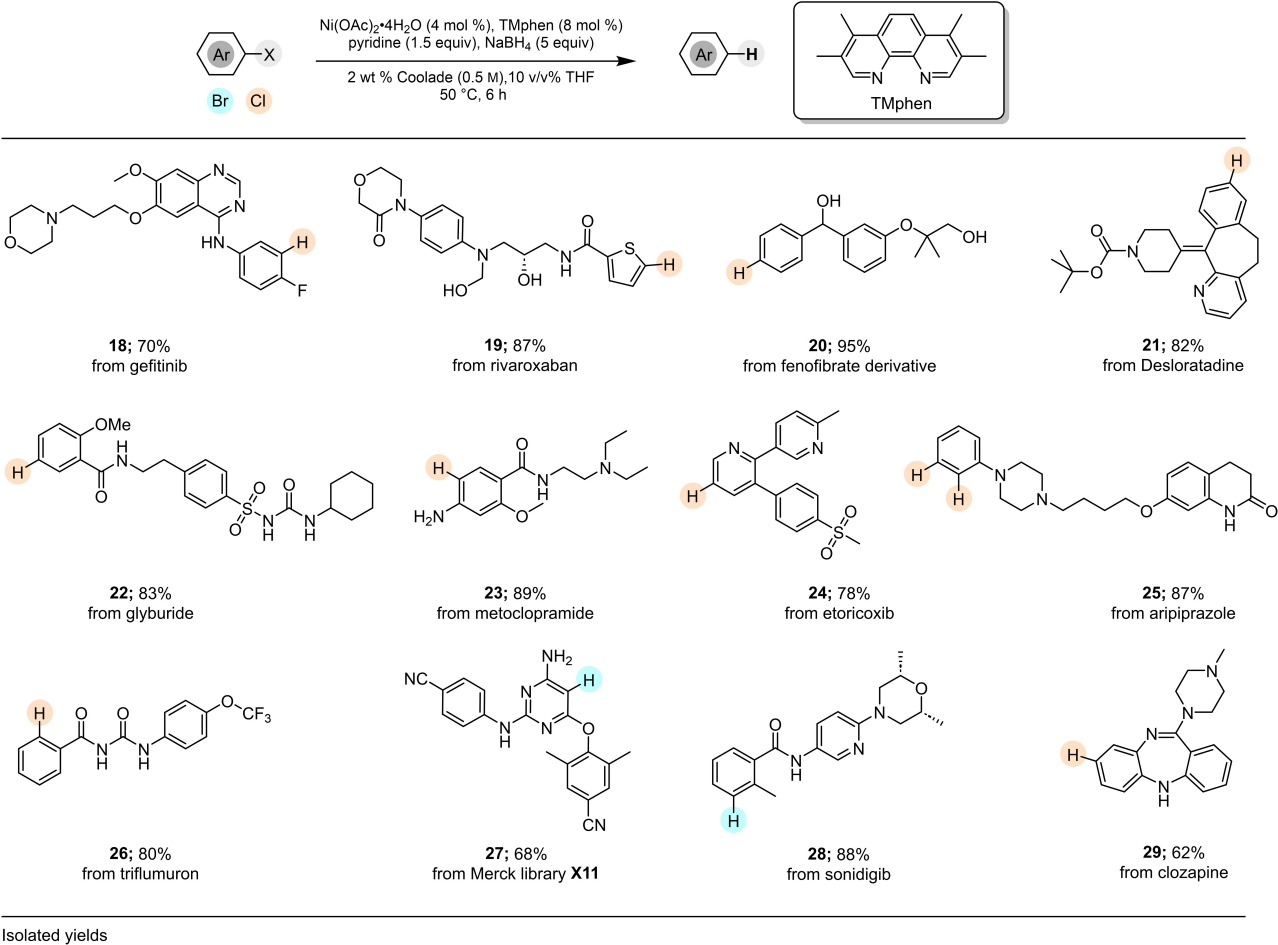
Dehalogenation of highly functionalized substrates in water.

The promising functional group tolerance and mild reaction conditions associated with this protocol suggested it could be applied to the pharmaceutical area, and in general, the fine chemicals industry. Thus, as illustrated in Figure 3, dehalogenation of several known drugs, such as gefitinib (**18**), desloratadine (**21**), glyburide (**22**), the anticancer analogue of sonidegib (**28**), and clozapine (**29**) readily participate and led to the dehalogenated products in good isolated yields. In the case of aripiprazole (**25**), both original chlorine groups were smoothly dehalogenated (87 %). The highly complex substrate **X11**, from the Merck Informer Library,^[85]^ which is rich in nitrogen, afforded product **27** (68 %). Noteworthy is that ICP‐MS analysis of **21** following reduction of the precursor chloride and standard work up showed only 3 ppm of residual Ni present, which is very low compared to the allowed levels of nickel (20 ppm/dose/day) by the FDA.^[86]^ Use of other metal‐based processes[^38^, ^39^, ^40^, ^41^, ^42^, ^43^, ^44^, ^45^] typically do not offer any indication as to residual metal expected in the dehalogenated products.

**Figure 4:**
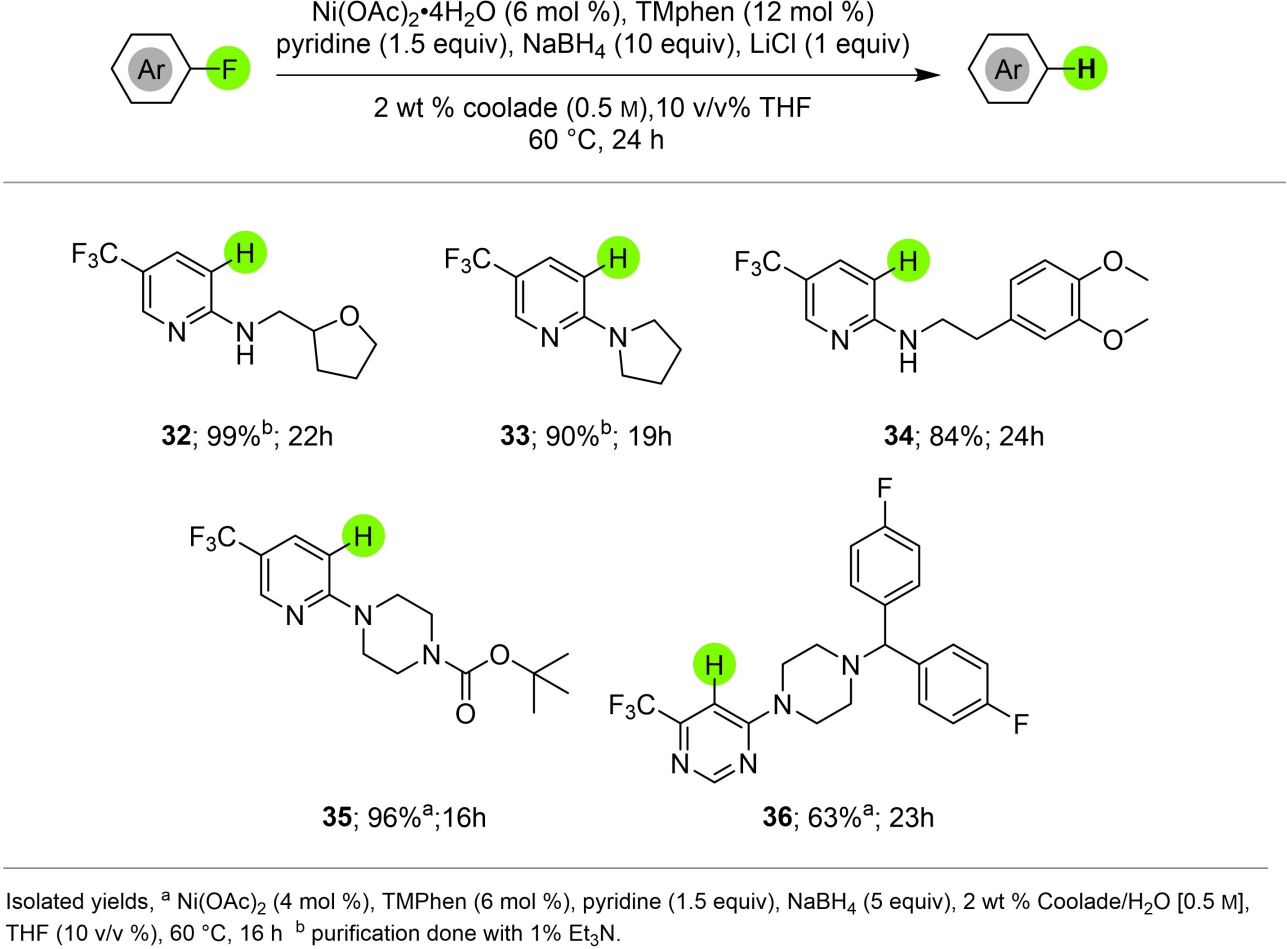
Dehalogenation of selected heteroaryl fluorides.

Further optimization was necessary for *defluorinations*, specifically involving a fluoro‐substituted pyridine derivative that appears to be one of the few systems found (thus far) to be amenable to these reductions (Table 3). Screening additives such as Ph_3_P at 80 °C led to a modest result (**31**; 73 %). However, addition of NaBH_4_ over time (entry 7) showed an increase in conversion to 83 % at 70 °C. The general impact of portion‐wise addition of NaBH_4_ was studied in greater detail (see SI; Table S6), indicating that with every sequential addition, an increase in conversion was observed. Hence, both sets of conditions were tested in all cases. A further improvement was made by increasing the level of Ni to 6 mol %, with the corresponding increase of the TMPhen loading to 12 mol %. This, together with the changes in hand (*vide supra*) led to 100 % conversion (see SI; Table S7). Unfortunately, these modified conditions were shown not to be general, leading to incomplete substrate conversion. Ultimately, addition of one equivalent of LiCl in the presence of the full amount of NaBH_4_ afforded an 88 % conversion and 84 % isolated yield (*e. g*., of product **34**; see SI; Table S8). Using these modified conditions a total of five activated heteroaryl fluorides could be successfully defluorinated (Figure 4).

**Table 3:**
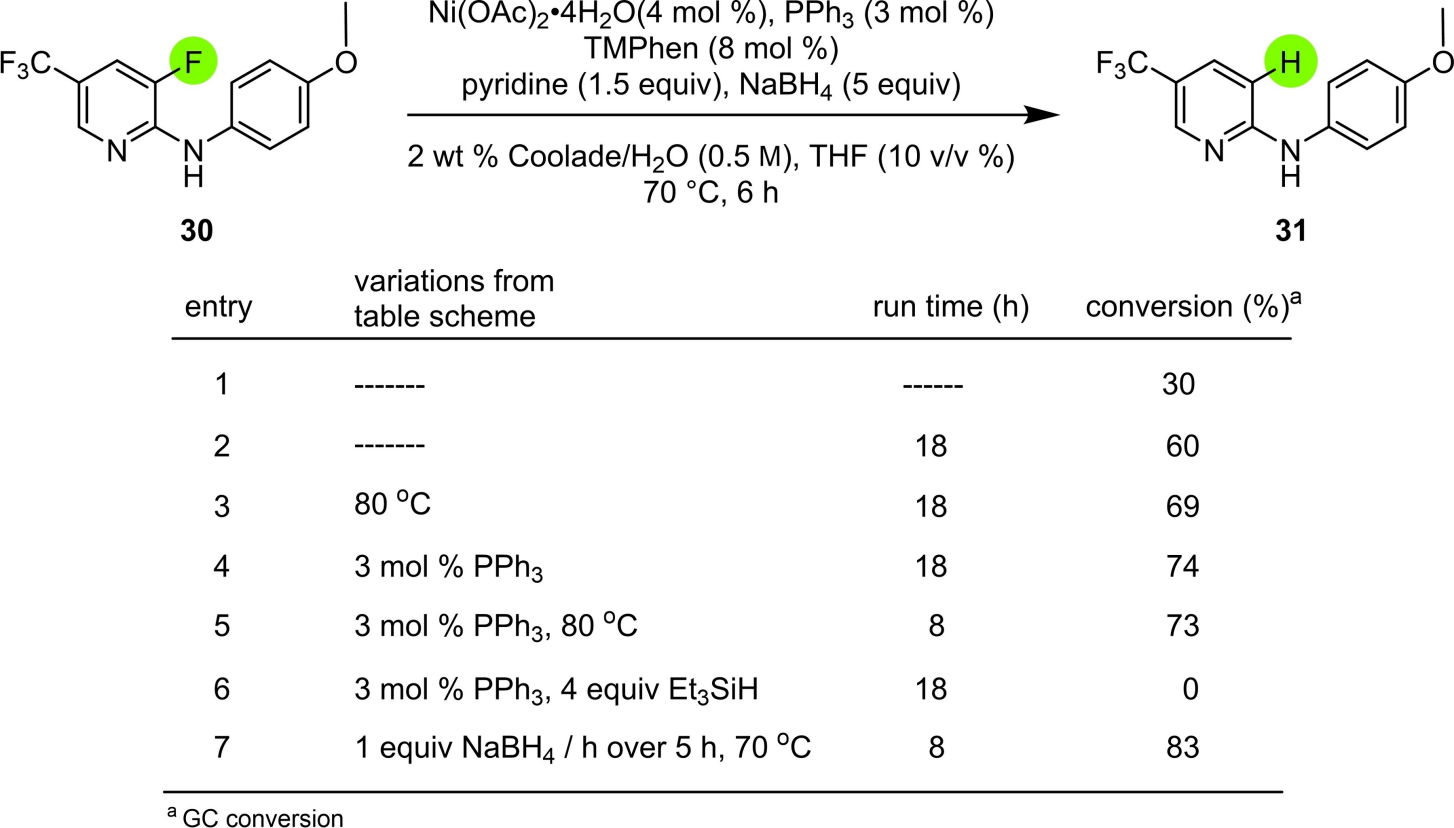
Additive screening.

**Figure 5:**
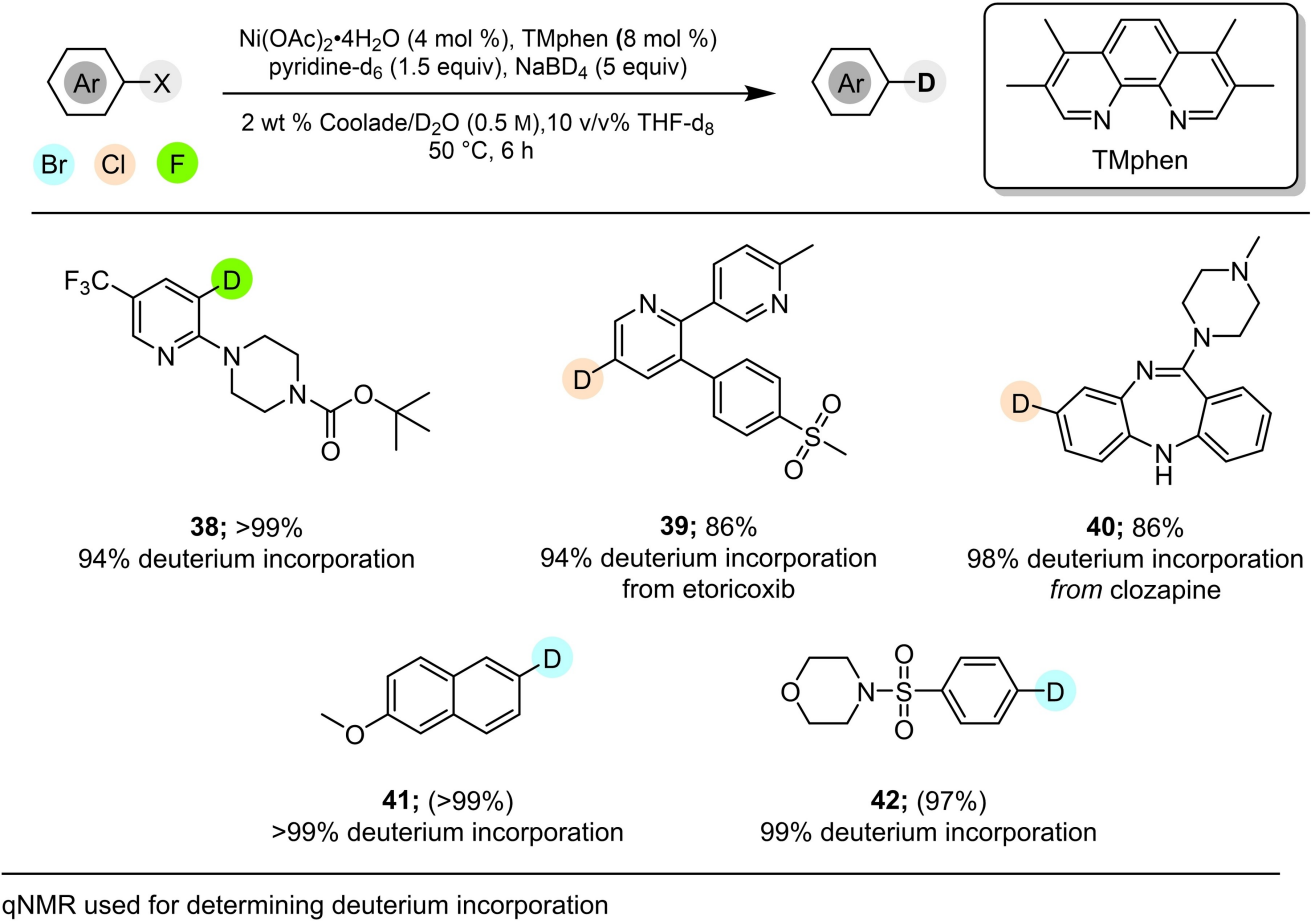
Deuterium incorporation into aryl chloride, bromides and fluorides.

Incorporation of deuterium rather than hydrogen was then investigated, starting with the highly functionalized pyridyl fluoride **37** (Table 4). The main source of deuterium incorporation was determined to derive from the presence of both NaBD_4_ and D_2_O (entry 3). The resulting optimized conditionsÐcould then be applied to the medically valued fluorinatedÐpharmaceutical bitopertin via intermediate 38, as well as theÐdechlorinated product 39 (from etoricoxib) and 40 (from clozapine; Figure 5). Two additional aryl bromides were also successfully converted to products **41** and **42** in high yields.

**Table 4:**
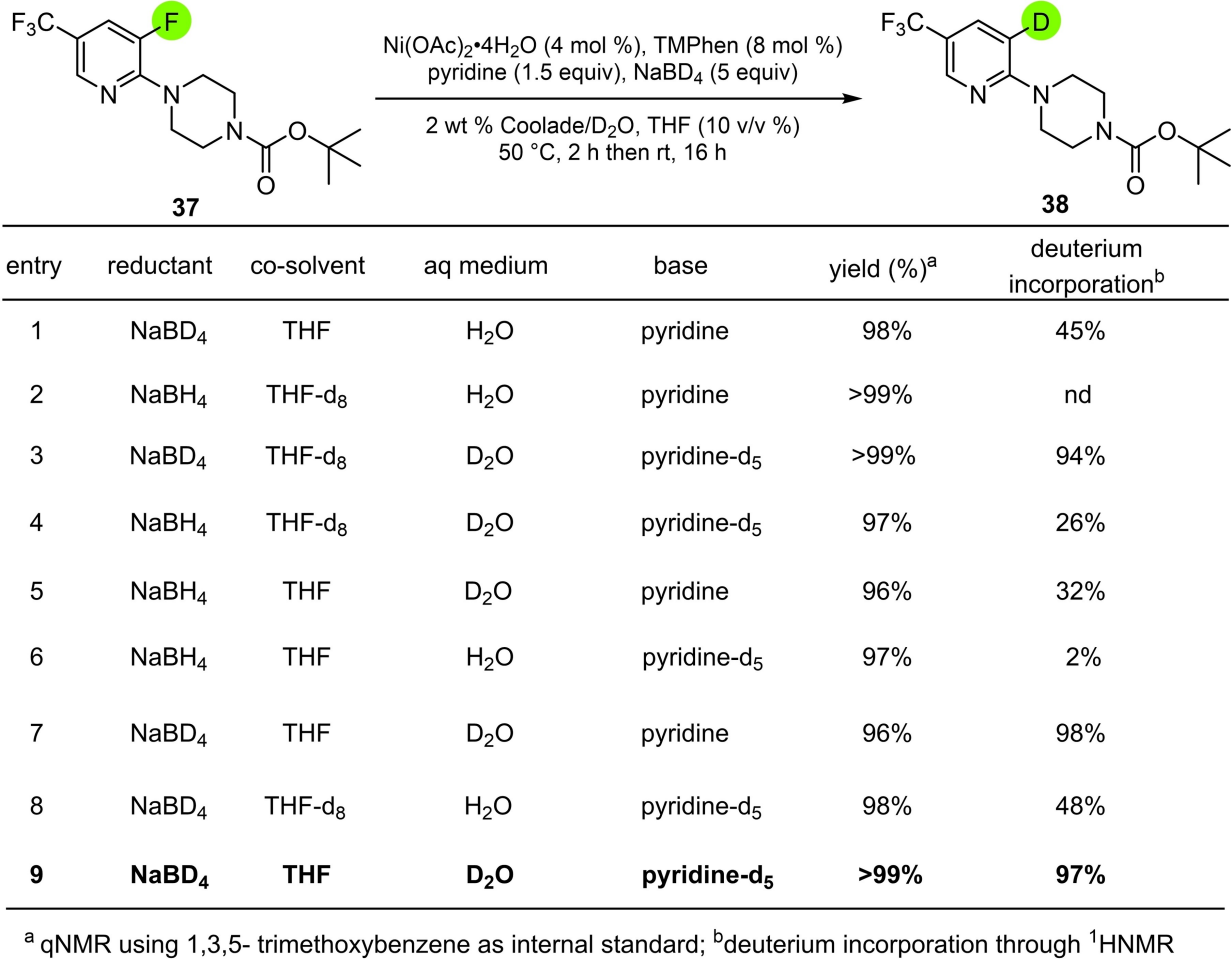
Optimization of deuterium incorporation in fluoropyridines.

**Figure 6:**
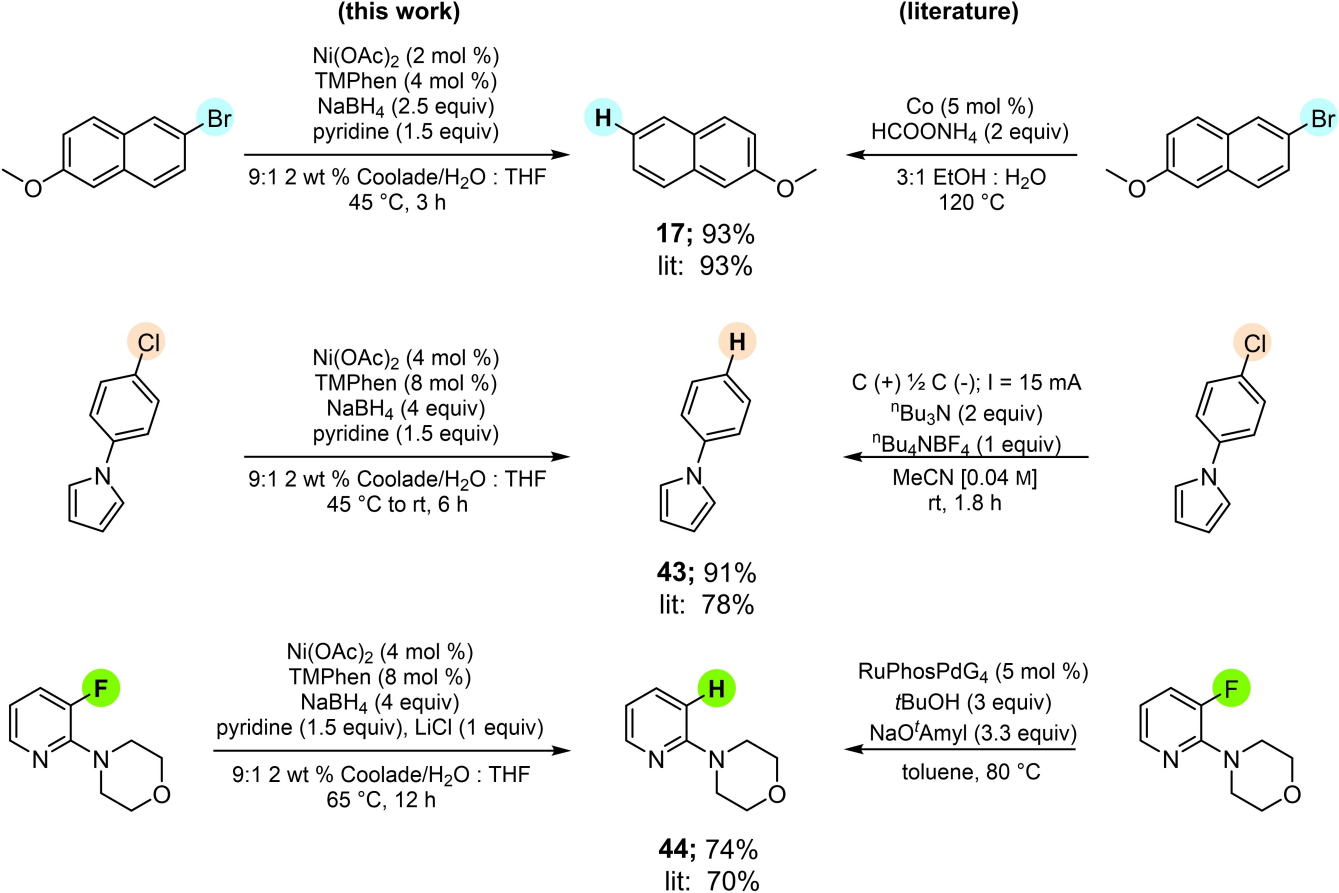
Direct comparisons with existing literature.

In direct comparisons to existing literature protocols, these reductions typically outperformed known methodologies (Figure 6). Thus, product **17** was efficiently formed from the precursor bromide in 93 % yield using 2 mol % nickel over three hours at 45 °C, while the literature procedure relies on 5 mol % of a cobalt complex with heating to 120 °C (80 h).^[87]^ *N*‐Phenylpyrrole, product **43**, was isolated in 91 % yield compared to the known electrochemical hydrodehalogenation which afforded the same arene to the extent of 78 %.^[88]^ Lastly, morpholinopyridine **44** was obtained in 74 % yield using 4 mol % nickel at 65 °C, compared to the literature protocol requiring 5 mol % of an expensive palladium catalyst at 80 °C (70 %), also being run in an organic solvent (toluene).^[89]^

**Scheme 1:**
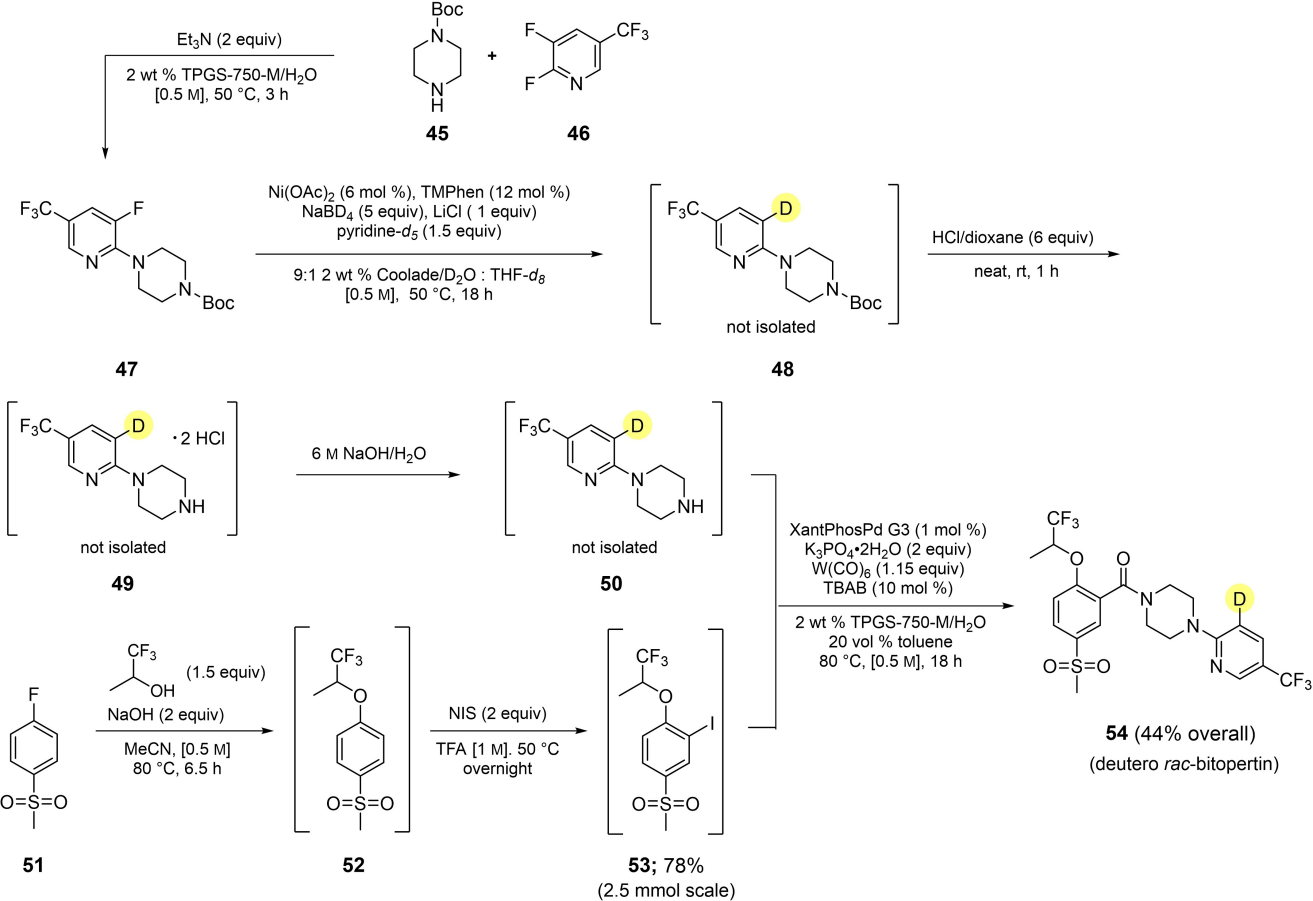
Synthesis of deutero‐*rac*‐ bitopertin.

For purposes of demonstrating utility of the method, and as part of a sequence using aqueous micellar conditions throughout, a route was developed to deutero *rac*‐bitopertin (Scheme 1). Deuteration of the key *N*‐Boc‐protected intermediate **47**, obtained via an S_N_Ar reaction between **45** and **46**, was performed leading to crude product **48**. Without isolation, its subsequent *N*‐Boc deprotection followed by conversion of the derived HCl salt (**49**), formed initially, to its free base provided deuterated pyridine **50**. This material, also used without isolation (as with **48** and **49**), was coupled with iodide **53** using our previously described carbonylative amide‐forming conditions^[90]^ to arrive at deuterated *rac*‐bitopertin **54** in 44 % overall yield.

**Figure 7:**
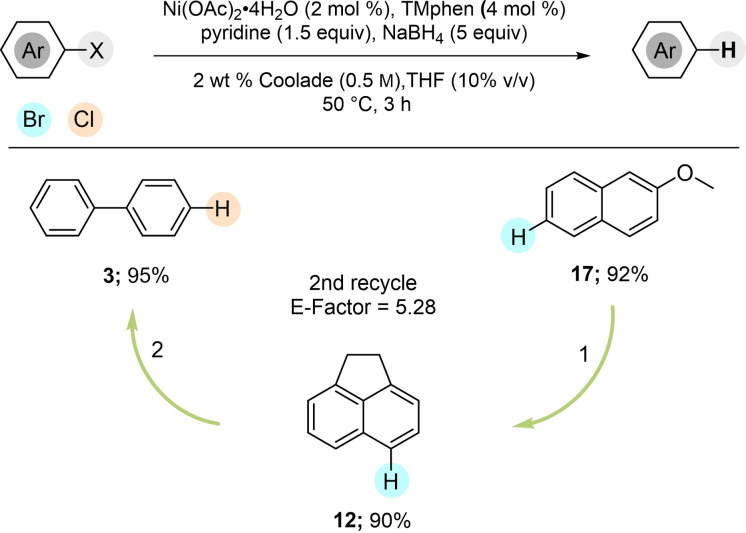
Recycle study.

To further document the greenness associated with this technology, the aqueous reaction medium was recycled twice with no significant loss in efficiency (Figure 7). However, when trying to recycle the nickel, there was a loss in efficiency where the yield dropped to 60 %. Due to this observation only the aqueous media was recovered and recycled. The Sheldon E‐Factor^[91]^ for the conversion of **12** to **3** was calculated to be 5.28, not including the EtOAc used for extraction (essential given the small scale involved). Including the extraction solvent, the E‐Factor was calculated to be 18.4.

## Conclusions

A new Ni‐catalyzed protocol has been developed for the hydrodehalogenation of (hetero)aryl halides using water as the reaction medium. This process is also amenable to halide replacement with deuterium. This method features operationally simple procedures that require no special equipment, a non‐precious metal, mild reaction conditions, readily available reagents, and good functional group tolerance. A diverse range of halogen‐containing substrates is amenable, including aromatic bromides and chlorides, and selected heteroaromatic fluorides. The method, likewise, can be applied to incorporation of deuterium using inexpensive and readily available D₂O and NaBD₄ as the sources of deuterium. The potential for applications to the fine chemicals industry, specifically for the modification of APIs, is documented. A sequence leading to a deuterated (and racemic) form of the known drug *rac‐*bitopertin currently in use for treatment of schizophrenia has also been illustrated. This protocol is environmentally attractive as it utilizes recyclable water, rather than egregious organic solvents. Consequently, the E‐Factors associated with these reductions are relatively low, indicating significant savings in both the cost and environmental impact of such reductions.

## Experimental Section

### Bromides and Chlorides

In a 1‐dram vial equipped with a PTFE coated magnetic stir bar were added Ni(OAc)_2_⋅4H_2_O (4 mol % relative to substrate) and the 3,4,7,8‐tetramethyl‐1,10‐phenanthroline (TMPhen, 8 mol %). The vial was sealed with a rubber septum, evacuated, and backfilled with argon three times using an argon/vacuum manifold. Subsequently, THF (10 v/v%) was added followed by 2 wt % solution of Coolade/H_2_O (0.5 M) and the vial was allowed to stir at rt for 10 min. After 10 min pyridine (1.5 equiv) was added. The halogenated starting material is then added, if solid by quickly removing the septum, sealing the vial with a rubber septum, and finally, evacuating and backfilling with argon three times. If the halogenated starting material is a liquid it is added via syringe. After the halogenated starting material has been added, the reaction mixture was stirred for 5 min at rt. After 5 min, NaBH_4_ (5 equiv) was added in two portions, by quickly removing the rubber septum. The vial was then re‐sealed with the rubber septum, evacuated, under reduced pressure, and back filled with argon three times. A small amount of foaming was observed, but after 5 min of stirring at rt the foaming subsides. The vial is then placed for 4–6 h at 50 °C. Upon completion (as monitored by TLC), the reaction was extracted with EtOAc (3 x 1 mL). The combined extracts were dried over anhydrous Na_2_SO_4_, filtered, and concentrated *in vacuo*. Column chromatography was used to purify all of the samples.

### Fluorides with Sequential Addition of NaBH_4_

In a 1‐dram vial equipped with a PTFE coated magnetic stir bar were added Ni(OAc)_2_⋅4H_2_O (6 mol %), and 3,4,7,8‐tetramethyl‐1,10‐phenanthroline (TMPhen 12 mol %). The vial was sealed with a rubber septum, evacuated, and backfilled with argon three times using an argon/vacuum manifold. Subsequently, THF (10 v/v%) was added followed by 2 wt % solution of Coolade/H_2_O (0.5 M) and the vial allowed to stir at rt for 10 min. After 10 min, pyridine (1.5 equiv) was added. The halogenated starting material was then added, if solid by quickly removing the septum and then re‐sealing the vial with the rubber septum, evacuating and backfilling with argon three times. If the halogenated starting material is a liquid, it is added via syringe. After the halogenated starting material has been added, it is allowed to stir for 5 min at rt. After 5 min, the first 2 equiv. of NaBH_4_ were added to the vial. A small amount of foaming was observed, but after 5 min of stirring at rt the foaming subsides. The vial was stirred for 1 h at 60 °C. After an hour the vial was cooled to rt and the next 2 equiv. of NaBH_4_ were added. This process was repeated until all 10 equiv. of NaBH_4_ had been added to the vial. It was then stirred at 60 °C for 18–24 h. Upon completion (as monitored by TLC), the reaction was extracted with EtOAc (3 x 1 mL). The combined extracts were dried over anhydrous Na_2_SO_4_, filtered, and concentrated *in vacuo*. Column chromatography was used to purify all of the samples.

### Fluorides Using LiCl

In a 1‐dram vial equipped with a PTFE coated magnetic stir bar were added Ni(OAc)_2_⋅4H_2_O (6 mol %), 3,4,7,8‐tetramethyl‐1,10‐phenanthroline (TMPhen, 12 mol %), and then LiCl (1 eqiuv). The vial was sealed with a rubber septum, evacuated, and backfilled with argon three times using an argon/vacuum manifold. Subsequently, THF (10 v/v%) was added followed by a 2 wt % solution of Coolade/H_2_O (0.5 M) and the vial was allowed to stir at rt for 10 min. After 10 min, pyridine (1.5 equiv) was added. The halogenated starting material was then added; if solid, by quickly removing the septum adding the starting material, and then re‐sealing the vial with the rubber septum, evacuating and backfilling with argon three times. If the halogenated starting material is a liquid, it is added via syringe. After the halogenated starting material has been added, the reaction mixture is allowed to stir for 5 min at rt. After 5 min, NaBH_4_ (10 equiv) was added in two portions, by quickly removing the rubber septum. The vial was sealed again with the rubber septum, evacuated, and back filled with argon three times. A small amount of foaming was observed, but after 5 min of stirring at rt the foaming subsides. The vial is then placed for 4–6 h at 70 °C. Upon completion (as monitored by TLC), the reaction was extracted with EtOAc (3 x 1 mL). The combined extracts were dried over anhydrous Na_2_SO_4_, filtered, and concentrated *in vacuo*. Column chromatography was used to purify all of the samples.

### Deuterations

In a 1‐dram vial equipped with a PTFE coated magnetic stir bar were added Ni(OAc)_2_⋅4H_2_O (4 mol %), and then 3,4,7,8‐tetramethyl‐1,10‐phenanthroline (TMPhen, 8 mol %). The vial was sealed with a rubber septum, evacuated, and backfilled with argon three times using an argon/vacuum manifold. Subsequently, THF (10 v/v%) was added followed by 2 wt % solution of Coolade/D_2_O (0.5 M) and the vial was allowed to stir at rt for 10 min. After 10 min, pyridine (1.5 equiv) was added. The halogenated starting material is then added, if solid by quickly removing the septum adding the starting material, sealing the vial again the vial with a rubber septum, evacuating and backfilling with argon three times. If the halogenated starting material is a liquid, it is added via syringe. After the halogenated starting material has been added, the reaction mixture is stirred for 5 min at rt. After 5 min NaBD_4_ was added to the vial in two portions, by quickly removing the rubber septum. The vial was re‐sealed with the rubber septum, evacuated, and back filled with argon three times. A small amount of foaming was observed, but after 5 min of stirring at rt the foaming subsides. The vial was placed on a hot plate at 50 °C for 6–16 h. Upon completion (as monitored by TLC), the reaction was extracted with EtOAc (3 x 1 mL). The combined extracts were dried over anhydrous Na_2_SO_4_, filtered, and concentrated *in vacuo*. Column chromatography was used to purify all the samples.

## Supporting Information

General information about materials, methods, optimization procedures, multistep synthesis of *rac*‐bitopertin, recycle study, analytical and spectral data are all provided in the Supporting Information. The authors have cited additional references within the Supporting Information.[^92^, ^93^, ^94^, ^95^, ^96^, ^97^, ^98^, ^99^, ^100^, ^101^, ^102^, ^103^, ^104^, ^105^, ^106^, ^107^, ^108^, ^109^, ^110^, ^111^, ^112^, ^113^]

## Conflict of Interests

The authors declare no conflict of interest.

1

## Supporting information

As a service to our authors and readers, this journal provides supporting information supplied by the authors. Such materials are peer reviewed and may be re‐organized for online delivery, but are not copy‐edited or typeset. Technical support issues arising from supporting information (other than missing files) should be addressed to the authors.

Supporting Information

## Data Availability

The data that support the findings of this study are available.
