# Supplementary material for: Nickel‐Catalyzed Hydro‐ and Deutero‐dehalogenations of (Hetero)Aryl Halides under Aqueous Micellar Catalysis Conditions
Source: ChemSusChem. 2025 Apr 24;18(11):e202500043. doi: 10.1002/cssc.202500043 (PMC12131707; doi:10.1002/cssc.202500043)

ChemSusChem

Supporting Information

Nickel-Catalyzed Hydro- and Deutero-dehalogenations of (Hetero)Aryl Halides under Aqueous Micellar Catalysis Conditions

Monica S. Lopez Lemus, Rahul D. Kavthe, Rohan M. Thomas, Max Baumann, Karthik S. Iyer, and Bruce H. Lipshutz*

Supporting Information

Nickel-Catalyzed Hydro- and Deutero-dehalogenations of (Hetero)Aryl Halides under Aqueous Micellar Catalysis Conditions

Monica Lopez Lemus, Rahul D. Kavthe, Rohan M. Thomas, Max Baumann, Karthik S. Iyer, and Bruce H. Lipshutz*

Department of Chemistry & Biochemistry, University of California, Santa Barbara, CA 93106 USA

Table of Contents

Contents

1. General information: Materials and methods S3

2. Optimization of reaction conditions for chlorides and bromides S4

2.1 Catalyst and Ligand screening S4

2.2 $NaBH_4$ loading: screening S5

2.3 Base screening S6

2.4 Screening of the reaction medium S7

3. Optimization conditions for fluorides S8

3.1 Additive screening S8

3.2 Sequential addition of $NaBH_4$ S9

3.3 Nickel and Ligand Loading S10

3.4 LiCl Screening S11

4. Optimization conditions for deuterium incorporation S12

4.1: Deuterium source screening using a fluoropyridine S12

5. Synthesis of deuterium-containing *rac*- bitopertin S13

Synthesis of compound **47** S13

Synthesis of **50** S14

Synthesis of **53** S14

Synthesis of **54** S15

6. Recycle Study S16

6.1 Procedure for recycling S16

6.2 Determination of E-Factors S17

7. Analytical Data S19

8. References S46

9. NMR Spectra S47

1. General information: Materials and methods

Reagents: All commercially available reagents were used without further purification. Reagents were purchased from Sigma–Aldrich, Combi–Blocks, Ambeed Inc., Alfa Aesar, Acros Organics, A2B chemicals, BLD Pharma, AK Scientific, or Aaron Chem. HPLC–grade water was obtained from both Sigma Aldrich and Fischer Scientific, and was purged with argon before use.

Surfactant Solution Preparation: A solution of 2 wt % surfactant/H_2O was prepared by dissolving the surfactant in degassed HPLC grade water and stored under argon. Coolade[1] was made as described previously and is also commercially available from Sigma–Aldrich (catalog # 907014).

Chromatography: Silica gel TLC plates (UV 254 indicator, thickness 200 mm standard grade, glass backed and 230–400 mesh from Merck, and Silicycle) were used. The developed TLC plate were analyzed with a UV lamp (254 nm). The plates were further analyzed with use of an aqueous ceric ammonium molybdate stain, potassium permanganate stain, or ethanolic vanillin and developed with a heat gun. Automated Flash chromatography was performed using CombiFlash NextGen 300+ using Silicycle Silicaflash® P60 unbonded grade silica.

Nuclear Magnetic Resonance Spectroscopy (NMR): ^{1}H, ^{13}C, and ^{19}F NMR were recorded at 25 ºC on an Agilent Technologies 400 MHz, a Bruker Avance III HD 400 MHz, a Bruker Avance NEO 500 MHz, a Varian Unity Inova 500 MHz, or a Varian Unity Inova 600 MHz spectrometer in $CDCl_3$ or DMSO–d_6 with residual $CHCl_3$ (^{1}H = 7.26 ppm, ^{13}C = 77.16 ppm) or DMSO (1 H = 2.54 ppm, 13C = 40.45 ppm) as the internal standard. Deuterated solvents were purchased from Cambridge Isotope Laboratories. Chemical shifts are reported in parts per million (ppm). The data presented will be reported as follows; chemical shift, multiplicity (s = singlet, bs = broad singlet, d = doublet, dd = doublet of doublet, t = triplet, q = quartet, quin = quintet, m = multiplet), coupling constant (if applicable), and integration.

Mass Spectrometry (MS): HRMS analyses (ESI–MS, CI–MS, GC–EI, or GC–CI) were performed by the UC Santa Barbara mass spectrometry facility. ICP–MS analysis was performed at the California NanoSystems Institute (CNSI), UCLA. ESI–MS analysis was performed on a Waters LCT Premier mass spectrometer equipped with an Alliance 2695 Separations module. EI–MS analysis was performed on a Waters GCT Premier mass spectrometer equipped with an Agilent 7890A GC oven and J&W Scientific DB–5ms+DG narrow bore column using helium carrier gas.

Inductively coupled plasma mass spectrometry (ICP–MS, NexION 2000, PerkinElmer) analysis was performed to detect nickel in powder samples. All samples were used as received without further purification or modification. Each sample was transferred to a clean Teflon vessel for acid digestion. Digestion was carried out with a mixture of concentrated HNO_3 (65–70%, Trace Metal Grade, Fisher Scientific) and HCl (35–38%, Trace Metal Grade, Fisher Scientific) in a ratio of 1:3 with a supplement of H_2O_2 (30%, Certified ACS, Fisher Scientific) at 200 °C for 50 min in a

microwave digestion system (Titan MPS, PerkinElmer). Once the sample was cooled to rt, it was subsequently diluted to make a final volume of 50 mL by adding filtered DI water for analysis. The calibration curve was established using a standard solution while the dwell time was 50 ms with thirty sweeps and three replicates with background correction.

2. Optimization of reaction conditions for chlorides and bromides

2.1 Catalyst and Ligand screening

In a 2-dram vial equipped with a PTFE coated magnetic stir bar were added Ni salts (2-4 mol %) and the ligand (4-8 mol %). The vial was sealed with a rubber septum, evacuated, and backfilled with argon three times using an argon/vacuum manifold. Subsequently, 2 wt % solution of Coolade/H_2O (0.45 mL, 0.5 M) was added and the vial was allowed to stir at rt for 10 min. Pyridine (2 equiv) was then added. Then, 1-(2-chlorophenyl)ethan-1-ol (39.0 mg, 0.25 mmol, 1 equiv) was dissolved in THF (50 µL, 10 v/v %) and added as a solution to the flask through the septum. After 5 min, $NaBH_4$ (2.5 equiv) was added in two portions; a small amount of foam was observed but after 5 min of stirring the foam disappeared. The vial was sealed with a rubber septum and the reaction was stirred for 4-6 h at 50 °C. Upon completion (as monitored by TLC), the reaction was extracted with EtOAc (3 x 1 mL). The combined extracts were dried over anhydrous Na_2SO_4, filtered, and concentrated *in vacuo*. Subsequently, 1 mL of $CDCl_3$ was added followed by the addition of 1,3,5–trimethoxybenzene as internal standard and the sample was analyzed by ^{1}H NMR (20 s relaxation delay).

Table S1: Catalyst and Ligand screening

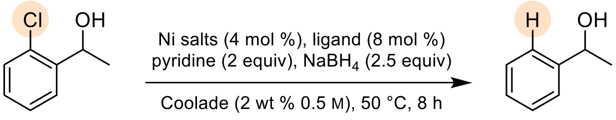

1 **2**

entry	Ni source	ligand	yield (%)[a]
1	$NiCl_2$	PPh_3	trace
2	$Ni(OAc)_2$•$4H_2O$	PPh_3	17
3	$Ni(OAc)_2$•$4H_2O$	dppf	31
4	$Ni(PPh_3)_2Br_2$	PPh_3	nd
5	$Ni(PPh_3)_2Cl_2$	-	nd
6	$Ni(OAc)_2$•$4H_2O$	PPh_3	15
7	$Ni(dppe)_2Cl_2$	-	nd
8	$Ni(PCy_3)_2Cl_2$	-	nd
9	$Ni(OAc)_2$•$4H_2O$	1,10-phenanthroline	17
**10**	**$Ni(OAc)_2$•$4H_2O$**	**L1**	**94**
11	$Ni(OAc)_2$•$4H_2O$	2,2' bipyridine	24
12	$Ni(OAc)_2$•$4H_2O$	**L2**	78
13	$Ni(OAc)_2$•$4H_2O$	**L3**	2
14	$Ni(OAc)_2$•$4H_2O$	TMEDA	nd
15	$Ni(OAc)_2$•$4H_2O$	**----**	nd
16	----------------------	**L1**	nd

[a]qNMR yields using 1,3,5 - trimethoxybenzene as internal standard

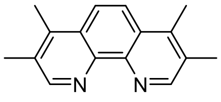

L1

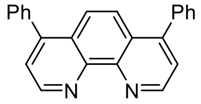

L2

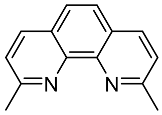

L3

2.2 $NaBH_4$ loading screening

In a 2-dram vial equipped with a PTFE coated magnetic stir bar were added $Ni(OAc)_2·4H_2O$ (4 mol %) and the 3,4,7,8-tetramethyl-1,10-phenanthroline (TMPhen, 8 mol %). The vial was sealed with a rubber septum, evacuated, and backfilled with argon three times using an argon/vacuum manifold. Subsequently, 2 wt % solution of Coolade/H_2O (0.45 mL, 0.5 M) was added and the vial was allowed to stir at rt for 10 min. Pyridine (30 µL, 0.37 mmol, 1.5 equiv) was then added. The 1-(2-chlorophenyl)ethan-1-ol (39.0 mg, 0.25 mmol, 1 equiv) was dissolved in THF (50 µL, 10 v/v %) and added as a solution to the flask through the septum. After 5 min, $NaBH_4$ (1-5 equiv) was added in two portions; a small amount of foam was observed but after 5 min stirring the foam

disappeared. The vial was sealed with a rubber septum and the reaction was stirred for 4-6 h at 50 °C. Upon completion (as monitored by TLC), the reaction was extracted with EtOAc (3 x 1 mL). The combined extracts were dried over anhydrous Na_2SO_4, filtered, and concentrated *in vacuo*. Subsequently, 1 mL of $CDCl_3$ was added followed by the addition of 1,3,5–trimethoxybenzene as internal standard and the sample was analyzed by ^{1}H NMR (20 s relaxation delay).

Table S2: $NaBH_4$ Loading: Screening

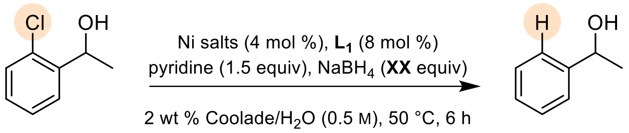

1 2

entry	$NaBH_4$ equiv	yield (%)[a]
1	1	47
2	2	79
3	3	99
4	4	85
**5**	**5**	**94**

[a]qNMR yields using 1,3,5- trimethoxybenzene as internal standard

2.3 Base screening

In a 2-dram vial was added $Ni(OAc)_2$•$4H_2O$ (4 mol %) and 3,4,7,8-tetramethyl-1,10-phenanthroline (TMPhen, 8 mol %). The vial was sealed with a rubber septum, evacuated, and backfilled with argon three times using an argon/vacuum manifold. A 2 wt % solution of Coolade/H_2O (0.4 mL, 0.5 M) was added. The base (0.3 mmol, 1.5 equiv) was then added. The 1-(2-chlorophenyl)ethan-1-ol (39.0 mg, 0.25 mmol, 1 equiv) was dissolved in THF (50 µL, 10 v/v %) and added as a solution to the flask through the septum. After 5 min, $NaBH_4$ (5 equiv) was added in two portions; a small amount of foam was observed but after 5 min stirring the foam disappeared. The reaction was stirred for 8 h at 50 °C. Upon completion (as monitored by TLC), the reaction was extracted with EtOAc (3 x 1 mL). The combined extracts were dried over anhydrous Na_2SO_4, filtered, and concentrated *in vacuo*. Subsequently, 1 mL of $CDCl_3$ was added followed by the addition of 1,3,5–trimethoxybenzene as internal standard and the sample was analyzed by ^{1}H NMR.

Table S3: Base Screening

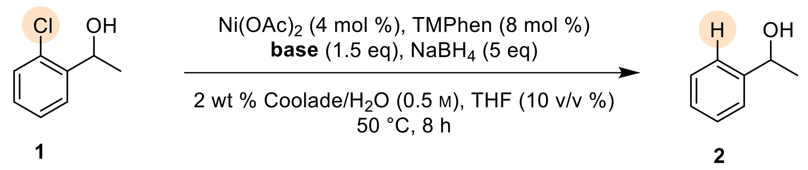

entry	**base**	yield (%)[a]
1	Et_3N	nd
2	DMAP	trace
3	2-picoline	94
**4**	**pyridine**	**97**
5	2,6 lutidine	36
6	K_3PO_4	nd
7	Cs_2CO_3	nd
8	NaOH	nd
9	*t*-BuOK	nd

[a]qNMR yields using 1,3,5- triomethoxybenzene as internal standard

2.4 Screening of the reaction medium

In a 2-dram vial was added $Ni(OAc)_2$•$4H_2O$ (4 mol %) and 3,4,7,8-tetramethyl-1,10-phenanthroline (TMPhen, 8 mol %). The vial was sealed with a rubber septum, evacuated, and backfilled with argon three times using an argon/vacuum manifold. The solvent (0.5 mL, 0.5 M) was added. The formation of a Ni-TMPhen complex was observed upon stirring the solution for 10 min. Pyridine (0.3 mmol, 1.5 equiv) was then added. If no co-solvent is used, or water is the only reaction medium, the 1-(2-chlorophenyl)ethan-1-ol (0.25 mmol, 1 equiv) substrate is added and stirred. After 5 min, $NaBH_4$ (1 mmol, 5 equiv) was added in one portion and the vial was re-capped under argon. The reaction was stirred at a specified temperature under argon. Upon completion (as monitored by TLC), the reaction was extracted with EtOAc (3 x 1 mL). The combined extracts were dried over anhydrous Na_2SO_4, filtered, and concentrated *in vacuo*. Subsequently, 1 mL of $CDCl_3$ was added followed by the addition of 1,3,5–trimethoxybenzene as internal standard and the sample was analyzed by ^1H NMR.

Table S4: Surfactant Screening

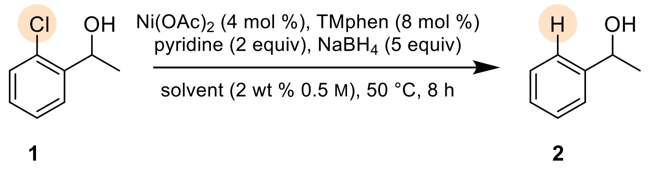

entry	surfactant	yield (%)[a]
1	TPGS-750-M (2 wt %)	28
2	Savie (2 wt %)	69
3	Brij 30	86
**4**	**Coolade (2 wt %)**	**94**
5	pure water	90
6	Coolade (2 wt %) and 10 v/v % THF	97

[a]qNMR yields using 1,3,5- trimethoxybenzene as internal standard

3. Optimization conditions for fluorides

3.1 Additive screening

In a 1-dram vial was added Ni(OAc)$_2$•4H$_2$O (2.5 mg, 4 mol %) and 3,4,7,8-tetramethyl-1,10-phenanthroline (TMPhen, 4.7 mg, 8 mol %). The vial was sealed with a rubber septum, evacuated, and backfilled with argon three times using an argon/vacuum manifold. THF (50 µL, 10 v/v%) was added followed by a 2 wt % solution of Coolade/H$_2$O (0.450 mL, 0.5 M), and then pyridine (30 µL, 1.5 equiv). The reaction mixture was left to stir at rt for 5 min. Afterwards, **30** (71.5 mg, 0.25 mmol, 1 equiv) was added by removing the septum to add (quickly) **30**, after which the vial was re-sealed with the rubber septum, evacuated, and backfilled with argon three times using an argon/vacuum manifold. After 5 min of stirring at rt, NaBH$_4$ (5 equiv) was added in two portions; a small amount of foam was observed but after 5 min stirring the foam disappeared. The reaction was stirred for 6 h at 70 °C. After completion, EtOAc (2 mL) was added and the reaction mixture extracted with EtOAc (3 x 2 mL). The combined extracts were dried over anhydrous Na$_2$SO$_4$, filtered, and concentrated *in vacuo*. Subsequently, a sample was taken to determine by GC conversion the extent of product formation *vs.* residual starting material remaining.

Table S5: Additive Screening

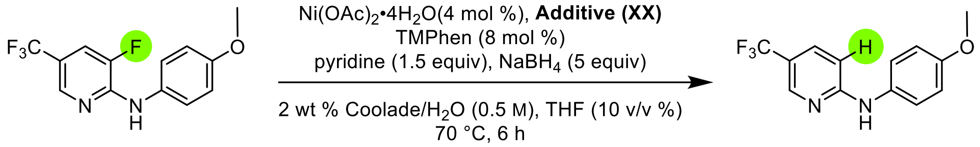

entry	additive	run time (h)	conversion (%)[a]
1	-------	------	30
2	-------	18	60
3	80 °C	18	69
4	3 mol % PPh_3	18	74
5	3 mol % PPh_3, 80 °C	8	73
6	3 mol % PPh_3, 4 equiv Et_3SiH	18	0
7	1 equiv $NaBH_4$ / h over 5 h, 70 °C	8	83

[a]GC conversion

3.2 Sequential addition of $NaBH_4$

In a 1-dram vial was added $Ni(OAc)_2•4H_2O$ (2.5 mg, 4 mol %) and 3,4,7,8-tetramethyl-1,10-phenanthroline (TMPhen, 4.7 mg, 8 mol %). The vial was sealed with a rubber septum, evacuated, and backfilled with argon three times using an argon/vacuum manifold. THF (50 µL, 10 v/v%) was added followed by a 2 wt % solution of Coolade/H_2O (0.450 mL, 0.5 M), and then pyridine (30 µL, 1.5 equiv). This mixture was stirred at rt for 5 min. Afterwards, **30** (71.5 mg, 0.25 mmol, 1 equiv) was added quickly by opening the septum, after which the vial was re-sealed with the rubber septum, evacuated, and backfilled with argon three times using an argon/vacuum manifold. After 5 min of stirring at rt, 1 equiv of $NaBH_4$ was added at time zero, with small amounts of foaming observed. After each h, the reaction while stirring was cooled to rt and another 1 equiv of $NaBH_4$ was added, capped, evacuated 3 times and backfilled with argon, after which the reaction mixture was then placed on a 70 °C aluminum block. This was repeated every h until all 5 equivalents of $NaBH_4$ had been added. The reaction was stirred for a total of 6 h at 70 °C. After completion, the reaction was dissolved in EtOAc (2 mL), extracted with EtOAc, and the combined extracts were dried over anhydrous Na_2SO_4, filtered, and concentrated *in vacuo*. Subsequently, a sample was taken to determine by GC conversion the extent of product formation compared to the amount of residual starting material remaining.

Table S6: Sequential addition of $NaBH_4$

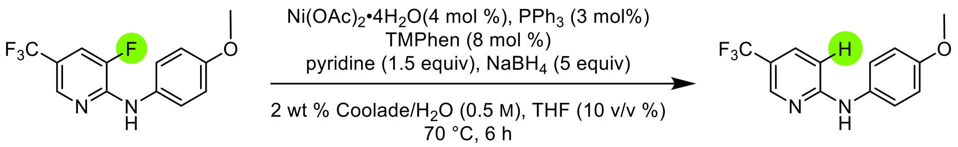

30 **31**

entry	NaBH4 equiv	run time (h)	conversion (%)[a]
1	1	0	NA
2	2	1	52
3	3	2	68
4	4	3	78
5	5	4	77
6	-----	6	75

[a]GC conversion

3.3 Nickel and Ligand Loading

In a 1-dram vial was added $Ni(OAc)_2•4H_2O$ (4-8 mol %) and 3,4,7,8-tetramethyl-1,10-phenanthroline (TMPhen 8-16 mol %). The vial was sealed with a rubber septum, evacuated, and backfilled with argon three times using an argon/vacuum manifold. THF (50 µL, 10 v/v%) was added followed by a 2 wt % solution of Coolade/H_2O (0.45 mL, 0.5 M) along with pyridine (30 µL, 1.5 equiv). This was left to stir at rt for 5 min. Afterwards, **30** (71.5 mg, 0.25 mmol, 1 equiv) was added by opening the septum to quickly after which the vial was re-sealed with the rubber septum, evacuated, and backfilled with argon three times using an argon/vacuum manifold. After 5 min of stirring at rt, $NaBH_4$ (10 equiv) was added in two portions; a small amount of foam was observed but after 5 min of stirring the foam disappeared. The reaction was stirred for 24 h at 60 °C. After completion, the reaction was dissolved in EtOAc (2 mL), extracted, and combined extracts were dried over anhydrous Na_2SO_4, filtered, and concentrated *in vacuo*. Subsequently, a sample was taken to determine the extent of conversion to product by GC compared to the amount of starting material remaining.

Table S7: Nickel and ligand loading screen: Defluorinations

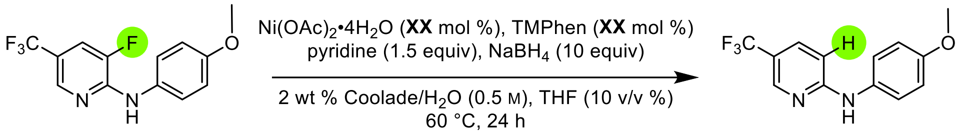

30 **31**

entry	Ni(OAc)2 Loading	TMPhen Loading	conversion (%)[a]
1	4 mol%	8 mol%	24
2	6 mol%	12 mol%	89
3	8 mol %	16 mol %	60
4	6 mol%	12 mol%	78
5	6 mol%	18 mol%	76
6	6 mol%	24 mol%	100
**7**	**6 mol%**	**12 mol%**	**100[b]**
8	6 mol%	24 mol%	100[b]
9	6 mol%	12 mol%	60[c]

[a]GC conversion, [b] sequential addition of $NaBH_4$, [c] no sequential addition of $NaBH_4$, PPh_3 (3 mol%)

3.4 LiCl Screening

In a 1-dram vial was added $Ni(OAc)_2$•$4H_2O$ (6 mol %) and 3,4,7,8-tetramethyl-1,10-phenanthroline (TMPhen, 12 mol %). A septum was installed and the flask was purged with argon. THF (50 µL, 10 v/v%) was added followed by a 2 wt % solution of Coolade/H_2O (0.450 mL, 0.5 M] along with pyridine (30 µL, 1.5 equiv). This was left to stir at rt for 5 min. Afterwards, *N*-(3,4-dimethoxyphenethyl)-3-fluoro-5-(trifluoromethyl)pyridin-2-amine was added (86.0 mg, 0.25 mmol, 1 equiv) quickly by opening the septum, after which the vial was re-sealed with the rubber septum, evacuated, and backfilled with argon three times using an argon/vacuum manifold. After 5 min of stirring at rt, $NaBH_4$ (10 equiv) was either added sequentially over 5 h, or in two portions. Small amounts of foaming were observed but after 5 min the foaming subsided and the reaction was then stirred at 60 °C for 24 h. After completion, the reaction was extracted with EtOAc (3 x 1mL), extracted, and the combined extracts were dried over anhydrous Na_2SO_4, filtered, and concentrated *in vacuo*. Subsequently, a sample was taken to determine the extent of conversion to product by GC. Some products where isolated *via* column chromatography using 20% EtOAc/ hexanes.

Table S8: LiCl screening

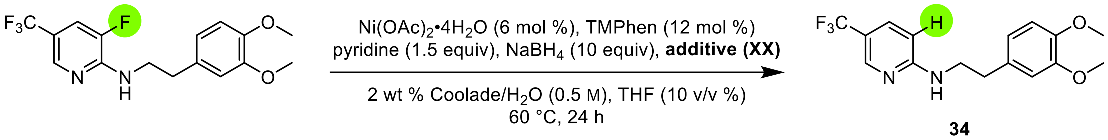

entry	additive	additive loading	conversion (%)[a]	Isolated yield
1	-------	--------	--------	68[b]
2	$P(N\text{-}Bu_2)_3$	12 mol%	NR	------[c]
3	$ZnCl_2$	1 equiv.	50	------[c]
**4**	**LiCl**	**1 equiv.**	**88**	**84[c]**
5	LiCl	1 equiv.	96	71[b]
6	LiCl	2 equiv.	100	77[c]

[a]GC conversion, [b] sequential addition of $NaBH_4$, [c] no sequential addition of $NaBH_4$

4. Optimization conditions for deuterium incorporation

4.1: Deuterium source screening using a fluoropyridine

In a 1-dram vial equipped with a PTFE coated magnetic stir bar were added $Ni(OAc)_2{\cdot}4H_2O$ (4 mol %) and TMPhen (8 mol %). The vial was sealed with a rubber septum, evacuated, and backfilled with argon three times using an argon/vacuum manifold. Subsequently, 2 wt % solution of Coolade/D_2O (0.45 mL) was added and the vial was allowed to stir at rt for 10 min. THF-d_8 (50 µL, 10 v/v%) and pyridine-d_5 (30 µL, 0.37 mmol, 1.5 equiv) were added and the vial was allowed to stir for 5 min, after which **37** (39.0 mg, 0.25 mmol, 1 equiv) was then added quickly. The vial was re-sealed with the rubber septum, evacuated and back filled with argon three times. After 5 min, $NaBD_4$ (5 equiv) was added in two portions. The vial was re-sealed with the rubber septum, evacuated and back filled with argon three times. A small amount of foaming was observed, but after 5 min of stirring at rt the foaming subsided. The vial was stirred at 50 °C for 2 h and then at rt for 16 h. Upon completion (as monitored by TLC), the reaction was extracted with EtOAc (3 x 1 mL). The combined extracts were dried over anhydrous Na_2SO_4, filtered, and concentrated *in vacuo*. Subsequently, 1 mL of $CDCl_3$ was added followed by the addition of 1,3,5–trimethoxybenzene as internal standard and the sample was analyzed by ^1H NMR (20 s relaxation delay).

For compounds that are not fluorides, reaction times between 3-6 h was enough for deuterium incorporation following the same protocol.

Table S9: Optimization of deuterium incorporation in fluoropyridines

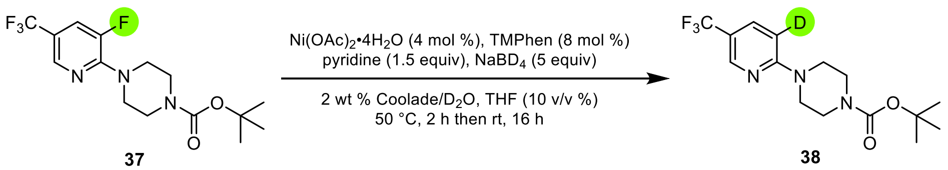

entry	reductant	organic co-solvent	aqueous medium	base	yield (%)[a]	deuterium incorporation[b]
1	$NaBD_4$	THF	H_2O	pyridine	98%	45% deuterium incorporation
2	$NaBH_4$	THF-d_8	H_2O	pyridine	>99%	no deuterium incorporation
3	$NaBD_4$	THF-d_8	D_2O	pyridine-d_5	>99%	94% deuterium incorporation
4	$NaBH_4$	THF-d_8	D_2O	pyridine-d_5	97%	26% deuterium incorporation
5	$NaBH_4$	THF	D_2O	pyridine	96%	32% deuterium incorporation
6	$NaBH_4$	THF	H_2O	pyridine-d_5	97%	02% deuterium incorporation
7	$NaBD_4$	THF	D_2O	pyridine	96%	98% deuterium incorporation
8	$NaBD_4$	THF-d_8	H_2O	pyridine-d_5	98%	48% deuterium incorporation
**9**	**$NaBD_4$**	**THF**	**D_2O**	**pyridine-d_5**	**>99%**	**97% deuterium incorporation**

[a]qNMR using 1,3,5- trimethoxybenzene as internal standard; [b]deuterium incorporation through ^{1}HNMR

5. Synthesis of deuterium-containing *rac*- bitopertin

Synthesis of compound **47**

To a stirring solution of 1-*N*-Boc-piperazine (**45**; 558.78 mg, 3 mmol, 1 equiv) in 3 mL of 2 wt % TPGS-750-M/H_2O in a 3-dram vial were added 2,3-difluoro-5-trifluoromethyl pyridine (**46**) (549.27 mg, 3 mmol, 375 µL, 1 equiv) followed by triethylamine (303 mg, 3 mmol, 400 µL, 1 equiv). The vial was then capped and left to stir for 3 h at 50 °C, or until all **46** was consumed as judged by TLC. The precipitate produced was too fine to filter, so brine was added (3 mL) and the aqueous solution was extracted with EtOAc (3 x 5 mL). The combined organic extracts were dried with anhydrous $MgSO_4$ and the EtOAc removed *in vacuo* to yield **47** (1027 mg, 98% yield) as an off-white powder.

Synthesis of 50

A 1-dram vial equipped with a Teflon-coated magnetic stir bar was charged with Ni(OAC)$_2$·4 H_2O (7.5 mg, 0.03 mmol, 6 mol %) and TMPhen (14.2 mg, 0.06 mmol). The vial was then brought into an argon filled glovebox where LiCl (21.2 mg, 0.5 mmol) was added and it was then sealed with a rubber septum. Outside the glovebox, 0.1 mL THF-d$_8$ was added, followed by 0.9 mL of 2 wt % Coolade/D_2O. This stock solution was stirred for 15 min at rt.

To a second 1-dram vial, **47** was added (87.3 mg, 0.25 mmol, 1 equiv), and the vial capped with a rubber septum and then evacuated and backfilled with argon 3 times. To this was then added pyridine-d$_5$ (31.5 mg, 0.375 mmol,32 µL, 1.5 equiv) via microsyringe, followed by 0.5 mL of the catalyst stock of aqueous surfactant solution. The mixture was allowed to stir at rt for 5 min before the septum was removed and $NaBD_4$ (52.3 mg, 1.25 mmol, 5 equiv) was quickly added and then the septum was re-affixed to the vial and secured with Teflon tape. The vial was then allowed to stir at 65 °C for 18 h before being extracted with EtOAc (3 x 1 mL) and the organic solvent then evaporated to leave crude **48** as an off-white powder.

A 2-dram vial with Teflon coated magnetic stir bar was charged with **48** and then 4 M HCl/dioxane (0.375 mL, 1.5 mmol, 6 equiv). The reaction was left to stir at rt and the solid **48** rapidly dissolved to form a clear solution, after which white material began to precipitate out. After one hour the reaction was diluted with Et_2O and transferred to a centrifuge to enhance precipitation. Diethyl ether was decanted off and the reaction then triturated with Et_2O two additional times. The remaining ether was removed *in vacuo* to leave **49** as a white solid cake.

To this cake, 6 M NaOH was added dropwise until all the solid had dissolved. After stirring for 15 min the solution was extracted with EtOAc until no more material was visible in the extract by TLC. The combined organics were removed *in vacuo* to leave **50** as an off-white powder.

Synthesis of 53

A 6-dram vial was charged with 1-(methylsulfonyl)-4-fluorobenzene (870 mg, 5 mmol, 1 equiv), NaOH (400 mg, 10 mmol, 2 equiv), dry CH_3CN (10 mL) and 3,3,3-trifluoromethyl-2-propanol (855 mg, 7.5 mmol, 1.5 equiv, 0.660 mL) and the vial sealed and heated to 70 °C. After 2.5 h the reaction was removed from heating and allowed to cool to rt before the solvent was removed *in vacuo*. The residue was dissolved in 10 mL of EtOAc and washed with sat NH_4Cl, H_2O, and brine before the EtOAc was evaporated to leave **52** as a white solid.

Compound **52** was dissolved in 5 mL of trifluoroacetic acid to which was then added NIS (2250 mg, 10 mmol, 2 equiv) and the vial sealed and heated at 50 °C overnight. The vial was removed from heating and cooled to 0 °C after which the reaction was quenched on ice with saturated sodium carbonate solution. Due to the volume of sodium carbonate required, the mixture was transferred to a larger vessel to limit losses due to bubbling. Upon complete quenching of the trifluoroacetic acid, the chunky suspension was extracted with EtOAc until no more material could

be observed by TLC. The combined organic layers were washed with brine and the solvent removed *in vacuo* to afford **52** as a beige powder (1537 mg, 78% yield).

Synthesis of 54

A 1-dram vial containing crude **50** was charged with XantPhosPdG3 (2.5 mg, 1 mol %), $K_3PO_4 \cdot 3H_2O$ (133 mg, 0.5 mmol, 2 equiv), $W(CO)_6$ (100 mg, mmol, 1.15 equiv), and TBAB (8 mg, 0.025 mmol, 10 mol %) and finally, **53** (147.8 mg, 0.375 mmol, 1.5 equiv). The vial was sealed with a rubber septum and evacuated and backfilled with argon 3 x. Through the septum, toluene (0.10 mL) followed by degassed 2 wt % TPGS-750-M/H_2O were added and the septum quickly removed and the vial capped with a phenolic plastic cap. The vial was then sealed and placed in a heating block at 80 °C for 18 h. The reaction was then cooled to rt and extracted with EtOAc before being purified by column chromatography eluting with hexanes to 80% EtOAc/hexanes after which the material was dried to afford **54** in 44% overall yield.

6. Recycle Study

6.1 Procedure for recycling

In a 1-dram vial equipped with a PTFE coated magnetic stir bar were added $Ni(OAc)_2$•$4H_2O$ (2 mol %) and TMPhen (4 mol %). The vial was sealed with a rubber septum, evacuated, and backfilled with argon three times using an argon/vacuum manifold. Subsequently, THF (100 μL, 10 v/v%) was added followed by a 2 wt % solution of Coolade/H_2O (0.900 mL; 0.5 M) and pyridine (60 μL, 1.5 equiv). This mixture was left to stir at rt for 5 min. Afterwards, 2-bromo-6-methoxynaphthalene (**17**) was added (118.1mg, 0.50 mmol, 1 equiv) by quickly removing the septum, adding **17,** and then re-sealing the vial with the rubber septum, evacuating and backfilling with argon three times. After 5 min of stirring at rt, $NaBH_4$ (5 equiv) was added in two portions. The vial was re-sealed with the rubber septum, evacuated and back filled with argon three times. After the foaming subsided it was placed on 50 °C aluminum heating block for 3 h. After the reaction had reached completion, tracking by TLC, the reaction was extracted with EtOAc (3 x 0.50mL). Between extractions, the EtOAc was stirred for 1 min before removing from the system. The remaining aqueous layer was filtered through a pad of Celite and collected in a 1-dram vial, to which fresh $Ni(OAc)_2$•$4H_2O$ (2 mol %) and TMPhen (4 mol %) were added. The vial was sealed with a rubber septum, evacuated, and backfilled with argon three times using an argon/vacuum manifold. Subsequently, THF (100 μL, 10 v/v%) was added followed by pyridine (60 μL, 1.5 equiv). This was left to stir at rt for 5 min. Afterwards, **17** was added (116.1mg, 0.50 mmol, 1 equiv) by quickly removing the septum, adding **17**, and re-sealing the vial with the rubber septum, and then evacuating and backfilling with argon three times. After 5 min of stirring at rt, $NaBH_4$ (5 equiv) was added in two portions. The vial was then sealed again with the rubber septum, evacuated and back filled with argon three times. After the foaming subsided it was placed on a 50 °C aluminum heating block for 3 h. The above extraction protocol was followed for the first recycle. Lastly, the same set up and extraction protocol was followed for 4-chlorobiphenyl (**3**).

6.2 Determination of E-Factors

Initial

Mass of reagents:

2-bromo-6-methoxynaphthalene	0.1181 g	
$Ni(OAc)_2 \cdot 4H_2O$	.00248 g	
TMPhen	0.00472 g	
pyridine	0.05868 g	
$NaBH_4$	0.10 g	
THF	0.089 g	
2 wt % Coolade/H_2O	0.90 g	Reagent waste: 1.27 g
Extraction		
EtOAc	0.902 g	Total waste: 2.17 g
Product	0.0707 g	

$$E - Factor\ including\ extraction: \quad \frac{2.17}{0.0707} = 31$$

$$E - Factor\ not\ including\ extraction: \quad \frac{1.27}{0.0707} = 18$$

1st Recycle

Mass of reagents:

5- bromoacenaphthlene	0.1165 g	
$Ni(OAc)_2 \cdot 4H_2O$	0.00248 g	
TMPhen	0.00472 g	
pyridine	0.05868 g	
$NaBH_4$	0.10 g	
THF	0.089 g	Reagent Waste: 0.371 g
Extraction		
EtOAc	0.902 g	Total waste: 1.27 g
Product	0.0693 g	

$$E - Factor\ with\ extraction: \frac{1.27}{0.0693} = \ 18$$

$$E - Factor\ without\ extraciton: \frac{0.37}{0.0693} = 5$$

2nd Recycle

Mass of reagents:		
4-chlorobiphenyl	0.0933 g	
$Ni(OAc)_2 \cdot 4H_2O$	0.00248 g	
TMPhen	0.00472 g	
pyridine	0.05868 g	
$NaBH_4$	0.10 g	
THF	0.089 g	Reagent Waste: 0.348 g
Extraction		
EtOAc	0.902 g	Total waste: 1.25 g
Product	0.0627 g	

$$E - Factor\ with\ extraction\colon\ \frac{1.25}{0.0627} = 20$$

$$E - Factor\ without\ extraction\colon \frac{0.348}{0.627} = 6$$

7. Analytical Data

1-Phenylethan-1-ol (2)

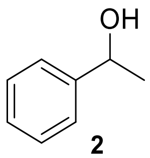

2

Product **2** was prepared according to General Procedure A at 50 °C for 5 h using 1-(2-chlorophenyl)ethan-1-ol (78.3 mg, 0.5 mmol) and $Ni(OAc)_2$•$4H_2O$ (5 mg, 4 mol %) as catalyst, and TMPhen (8 mol % 10 mg) as ligand. Chromatography conditions: 5–10% EtOAc/hexanes. Yield: 98%, 59.8 mg; colorless thick oil; $\mathbf{R_f}$ = 0.35 (10% EtOAc/hexanes).

^{1}H NMR (400 MHz, $CDCl_3$) δ 7.31 – 7.24 (m, 4H), 7.22 – 7.16 (m, 1H), 4.86 – 4.75 (m, 1H), 1.41 (d, J = 6.5 Hz, 3H).

^{13}C NMR (101 MHz, $CDCl_3$) δ 145.94, 128.61, 127.58, 125.51, 70.52, 25.27.

Spectral data matches that previously reported in the literature.[2]

1,1'-Biphenyl (3)

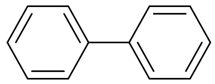

3

Product **3** was prepared according to General Procedure A at 50 °C for 3 h using 4-chloro-1,1'-biphenyl (94.33 mg, 0.5 mmol) and $Ni(OAc)_2$•$4H_2O$ (5 mg, 4 mol %) as catalyst, and TMPhen (8 mol % 10 mg) as ligand. Chromatography conditions: 100% hexanes. Yield: 95%, 73.5 mg; white solid; $\mathbf{R_f}$ = 0.70 (1% EtOAc/hexanes).

^{1}H NMR (400 MHz, $CDCl_3$) δ 7.64 – 7.55 (m, 4H), 7.54 – 7.40 (m, 4H), 7.39 – 7.32 (m, 2H).

^{13}C NMR (101 MHz, $CDCl_3$) δ 141.31, 128.83, 127.32, 127.24.

Spectral data matches that previously reported in the literature.[3]

Benzamide (4)

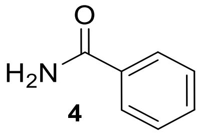

4

Product **4** was prepared according to General Procedure A at 50 °C for 6 h 3-bromobenzamide (77.79 mg, 0.5 mmol) and $Ni(OAc)_2{\bullet}4H_2O$ (5 mg, 4 mol %) as catalyst, and TMPhen (8 mol % 10 mg) as ligand. Chromatography conditions: 50% EtOAc/hexanes. Yield: 89%, 53.86 mg; off white solid; $\mathbf{R_f}$ = 0.40 (40% EtOAc/hexanes).

^{1}H NMR (500 MHz, $CDCl_3$) δ 8.01 – 7.84 (m, 3H), 7.56 – 7.49 (m, 2H).

^{13}C NMR (126 MHz, $CDCl_3$) δ 178.15, 160.76, 147.76, 147.60, 139.05, 138.97, 132.59, 129.25, 127.77, 126.54, 125.33, 120.80.

Spectral data matches that previously reported in the literature.[4]

1-(2,2,2-Trifluoroethyl)-1H-indazol-3-amine (5)

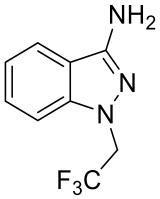

5

Product **5** was prepared according to General Procedure A at 50 °C for 6 h using 4-bromo-1-(2,2,2-trifluoroethyl)-1H-indazol-3-amine (124.5 mg, 0.5 mmol) and $Ni(OAc)_2{\bullet}4H_2O$ (5 mg, 4 mol %) as catalyst, and TMPhen (8 mol % 10 mg) as ligand. Chromatography conditions: 10% EtOAc/hexanes. Yield: 87%, 93.4 mg; $\mathbf{R_f}$ = 0.30 (30% EtOAc/hexanes).

^{1}H NMR (400 MHz, $CDCl_3$) δ 7.57 (dt, J = 8.1, 1.0 Hz, 1H), 7.42 (ddd, J = 8.2, 6.9, 1.1 Hz, 1H), 7.26 (s, 1H), 7.12 (ddd, J = 7.9, 7.0, 0.8 Hz, 1H), 4.70 (q, J = 8.5 Hz, 2H), 3.65 (s, 2H).

^{13}C NMR (101 MHz, $CDCl_3$) δ 148.84, 148.82, 142.01, 128.02, 127.90, 125.23, 122.43, 119.79, 119.68, 119.63, 115.72, 108.58, 49.73 ($q_{c\text{-}f}$ J = 34.96 Hz).

Spectral data matches that previously reported in the literature.[5]

Diphenylmethanol (6)

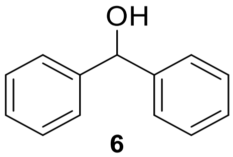

6

Product **6** was prepared according to General Procedure A at 50 °C for 6 h using (3-chlorophenyl)(phenyl)methanol (109.3 mg, 0.5 mmol) and $Ni(OAc)_2 \cdot 4H_2O$ (5 mg, 4 mol %) as catalyst, and TMPhen (8 mol % 10 mg) as ligand. Chromatography conditions: 10-15% EtOAc/hexanes. Yield: 94%, 86.5 mg; colorless thick liquid; $\mathbf{R_f}$ = 0.4 (20% EtOAc/hexanes).

^{1}H NMR (400 MHz, $CDCl_3$) δ 7.45 – 7.31 (m, 8H), 7.26 (s, 2H), 5.85 (d, *J* = 3.3 Hz, 1H), 2.21 (t, *J* = 3.1 Hz, 1H).

^{13}C NMR (101 MHz, $CDCl_3$) δ 143.85, 128.54, 127.61, 126.60, 76.28.

Spectral data matches that previously reported in the literature.[6]

2-Phenyl-1H-indole (7)

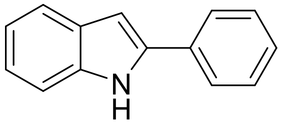

7

Product **7** was prepared according to General Procedure A at 50 °C for 8 h using 2-(4-chlorophenyl)-1H-indole (113.9 mg, 0.5 mmol) and $Ni(OAc)_2 \cdot 4H_2O$ (5 mg, 4 mol %) as catalyst, and TMPhen (8 mol % 10 mg) as ligand. Chromatography conditions: 5-10% EtOAc/hexanes. Yield : 99%, 95.5 mg; colorless thick liquid; $\mathbf{R_f}$ = 0.45 (10% EtOAc/hexanes).

H NMR (400 MHz, $CDCl_3$) δ 8.32 (s, 1H), 7.75 – 7.57 (m, 3H), 7.49 – 7.38 (m, 3H), 7.38 – 7.29 (m, 1H), 7.20 (ddd, *J* = 8.2, 7.1, 1.3 Hz, 1H), 7.13 (ddd, *J* = 8.0, 7.0, 1.1 Hz, 1H), 6.84 (dd, *J* = 2.2, 0.9 Hz, 1H).

^{13}**C NMR (101 MHz, DMSO-d$_6$)** δ 138.09, 137.61, 132.68, 129.67, 129.38, 129.11, 127.87, 125.44, 122.05, 120.53, 119.86, 111.79, 99.16.

Spectral data matches that previously reported in the literature.[7]

1,2,3,4-Tetrahydronaphthalen-1-ol (8)

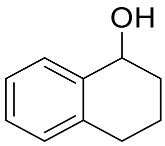

8

Product **8** was prepared according to General Procedure A at 50 °C for 6 h using 8-chloro-1,2,3,4-tetrahydronaphthalen-1-ol (45.5 mg, 0.25 mmol) and $Ni(OAc)_2$•$4H_2O$ (2.5 mg, 4 mol %) as catalyst, and TMPhen (8 mol % 5 mg) as ligand. Chromatography conditions: 10-20% EtOAc/hexanes. Yield: 89%, 70.1 mg; colorless thick liquid; $\mathbf{R_f}$ = 0.25 (10% EtOAc/hexanes).

^{1}**H NMR (400 MHz, CDCl$_3$)** δ 7.48 – 7.38 (m, 1H), 7.24 – 7.16 (m, 2H), 7.15 – 7.02 (m, 1H), 4.79 (d, *J* = 5.3 Hz, 1H), 2.84 (dt, *J* = 16.4, 5.4 Hz, 1H), 2.78 – 2.68 (m, 1H), 2.08 – 1.88 (m, 3H), 1.85 – 1.74 (m, 1H), 1.68 (d, *J* = 9.8 Hz, 1H).

^{13}**C NMR (101 MHz, CDCl$_3$)** δ 138.88, 138.84, 137.14, 129.03, 129.01, 128.73, 128.69, 127.59, 127.56, 126.19, 126.18, 68.15, 32.46, 32.29, 29.29, 29.27, 18.89, 18.83.

Spectral data matches that previously reported in the literature.[8]

1H-Indole (9)

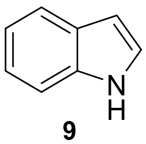

9

Product **9** was prepared according to General Procedure A at 50 °C for 6 h using 5-chloro-1H-indole (75.8 mg, 0.5 mmol) and $Ni(OAc)_2$•$4H_2O$ (5 mg, 4 mol %) as catalyst, and TMPhen (8 mol % 10 mg) as ligand. Chromatography conditions: 1-5% EtOAc/hexanes. Yield: 91%, 53.3 mg; white solid; $\mathbf{R_f}$ = 0.6 (5% EtOAc/hexanes).

^{1}H NMR (500 MHz, CDCl$_3$) δ 8.12 – 7.86 (m, 1H), 7.78 (t, *J* = 8.1 Hz, 1H), 7.43 (dd, *J* = 8.0, 1.1 Hz, 1H), 7.38 – 7.16 (m, 3H), 6.66 (qd, *J* = 3.4, 2.2 Hz, 1H).

^{13}C NMR (126 MHz, CDCl$_3$) δ 135.84, 127.92, 124.33, 124.29, 122.07, 120.84, 120.83, 119.92, 119.91, 111.20, 111.16, 102.62, 102.61.

Spectral data matches that previously reported in the literature.[9]

2,6-*bis*(Benzyloxy)pyridine (10)

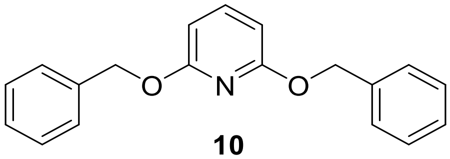

10

Product **10** was prepared according to General Procedure A at 50 °C for 6 h using 4-bromo-1,1'-biphenyl (162.9 mg, 0.5 mmol) and Ni(OAc)$_2$•4H$_2$O (5 mg, 4 mol %) as catalyst, and TMPhen (8 mol % 10 mg) as ligand. Chromatography conditions: 5-20% EtOAc/hexanes. Yield: 89%, 130.0mg mg; colorless solid; **R$_f$** = 0.63 (20% EtOAc/hexanes).

^{1}H NMR (500 MHz, CDCl$_3$) δ 7.50 (t, *J* = 7.9 Hz, 1H), 7.42 (d, *J* = 6.9 Hz, 4H), 7.39 – 7.33 (m, 4H), 7.33 – 7.28 (m, 2H), 6.37 (d, *J* = 7.9 Hz, 2H), 5.34 (s, 4H).

^{13}C NMR (101 MHz, CDCl$_3$) δ 162.40, 141.22, 137.70, 128.56, 127.91, 127.86, 102.08, 101.78, 101.70, 77.48, 77.16, 76.84, 67.96, 67.72.

Spectral data matches that previously reported in the literature.[10]

2,4-Dimethoxypyrimidine (11)

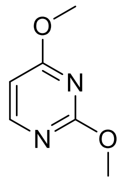

11

Product **11** was prepared according to General Procedure A at 50 °C for 6 h using 5-bromo-2,4-dimethoxypyrimidine (87.01 mg, 0.5 mmol) and $Ni(OAc)_2$•$4H_2O$ (5 mg, 4 mol %) as catalyst, and TMPhen (8 mol % 10 mg) as ligand. Chromatography conditions: 20-40% hexanes/EtOAc. Yield: 65%, 45.0 mg; colorless oil; $\mathbf{R_f}$ = 0.40 (40% EtOAc/hexanes).

^{1}H NMR (500 MHz, $CDCl_3$) δ 8.18 (d, *J* = 5.7 Hz, 1H), 6.37 (d, *J* = 5.7 Hz, 1H), 3.98 (d, *J* = 12.5 Hz, 6H).

^{13}C NMR (101 MHz, $CDCl_3$) δ 171.48, 165.50, 158.29, 102.07, 54.73, 53.72.

Spectral data matches that previously reported in the literature.[11]

1,2-Dihydroacenaphthylene (12)

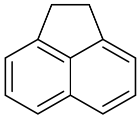

12

Product **12** was prepared according to General Procedure A at 50 °C for 3 h using 5-bromo-1-(triisopropylsilyl)-1H-indole (153.1 mg, 0.5 mmol) and $Ni(OAc)_2$•$4H_2O$ (5 mg, 4 mol %) as catalyst, and TMPhen (8 mol % 10 mg) as ligand. Chromatography conditions: 100% hexanes. Yield: 94%, 71.4 mg; white crystalline solid; $\mathbf{R_f}$ = 0.55 (100% hexanes).

^{1}H NMR (500 MHz, $CDCl_3$) δ 7.61 (d, *J* = 8.2 Hz, 2H), 7.46 (dd, *J* = 8.2, 6.9 Hz, 2H), 7.28 (s, 5H), 3.43 (s, 4H).

^{13}C NMR (101 MHz, $CDCl_3$) δ 146.06, 139.35, 131.68, 131.67, 127.85, 127.84, 122.29, 122.27, 119.23, 77.42, 77.10, 76.78, 30.41.

Spectral data matches that previously reported in the literature.[12]

1-(Triisopropylsilyl)-1H-indole (13)

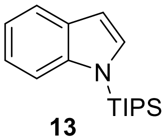

13

Product **13** was prepared according to General Procedure A at 50 °C for 6 h using 5-bromo-1-(triisopropylsilyl)-1H-indole (153.1 mg, 0.5 mmol) and $Ni(OAc)_2$•$4H_2O$ (5 mg, 4 mol %) as catalyst, and TMPhen (8 mol % 10 mg) as ligand. Chromatography conditions:10-20% hexanes/EtOAc. Yield: 74%, 100.4 mg; Red oil; $\mathbf{R_f}$ = 0.74 (20% EtOAc/hexanes).

^{1}H NMR (500 MHz, $CDCl_3$) δ 7.65 (dd, *J* = 7.5, 1.6 Hz, 1H), 7.54 (d, *J* = 8.0 Hz, 1H), 7.28 (s, 3H), 7.15 (dtd, *J* = 17.9, 7.1, 1.3 Hz, 2H), 6.65 (d, *J* = 3.2 Hz, 1H), 1.73 (hept, *J* = 7.5 Hz, 3H), 1.17 (d, *J* = 7.6 Hz, 18H).

^{13}C NMR (101 MHz, $CDCl_3$) δ 140.84, 131.45, 131.16, 121.35, 120.56, 119.77, 113.89, 104.73, 18.17, 12.88.

HRMS (TOF, MS, EI+): Calcd for $C_{17}H_{27}NSi$, $[M]^+$ 273.1908; found 273.1913.

2-(Piperidin-1-yl)pyrimidine (14)

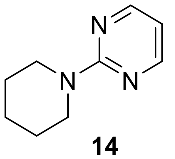

14

Product **14** was prepared according to General Procedure A at 50 °C for 6 h using 5-bromo-2-(piperidin-1-yl)pyrimidine (98.6 mg, 0.5 mmol) and $Ni(OAc)_2$•$4H_2O$ (5 mg, 4 mol %) as catalyst, and TMPhen (8 mol % 10 mg) as ligand. Chromatography conditions: 5-20 % EtOAc/hexanes. Yield: 85%, 63.9 mg; yellow oil; $\mathbf{R_f}$ = 0.45 (20% EtOAc/hexanes).

^{1}H NMR (500 MHz, $CDCl_3$) δ 8.28 (d, *J* = 4.7 Hz, 2H), 6.41 (t, *J* = 4.7 Hz, 1H), 3.95 – 3.46 (m, 4H), 1.67 (q, *J* = 5.9 Hz, 2H), 1.64 – 1.56 (m, 4H).

^{13}C NMR (126 MHz, CDCl$_3$) δ 161.71, 157.70, 109.06, 108.82, 44.76, 25.74, 25.62, 24.99, 24.89.

Spectral data matches that previously reported in the literature.[13]

1-(3-(Trifluoromethyl)phenyl)pyrrolidine (15)

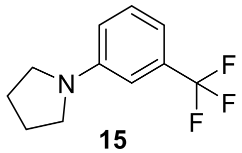

15

Product **15** was prepared according to General Procedure A at 50 °C for 3 h using 1-(4-bromo-3-(trifluoromethyl)phenyl)pyrrolidine (125.0 mg, 0.5 mmol) and Ni(OAc)$_2$•4H$_2$O (5 mg, 4 mol%) as catalyst, and TMPhen (8 mol% 10 mg) as ligand. Chromatography conditions: 1-5% hexanes/EtOAc. Yield: 58%, 62.0 mg; orange oil, **Rf** = 0.69 (5% EtOAc/hexanes).

^{1}H NMR (400 MHz, CDCl$_3$) δ 7.33 – 7.27 (m, 1H), 6.92 – 6.84 (m, 1H), 6.74 (t, *J* = 2.2 Hz, 1H), 6.68 (dd, *J* = 8.3, 2.5 Hz, 1H), 3.36 – 3.26 (m, 4H), 2.13 – 1.99 (m, 4H).

^{13}C NMR (101 MHz, CDCl$_3$) δ 147.95, 131.64, 131.32, 129.56, 126.11, 123.41, 114.65, 114.64, 111.76, 111.72, 111.68, 111.64, 107.97, 107.93, 107.89, 107.85, 47.75, 25.60.

Spectral data matches that previously reported in the literature.[14]

4-(5-Bromopyrimidin-2-yl)morpholine (16)

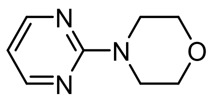

16

Product **16** was prepared according to General Procedure A at 50 ℃ for 3 h using 4-(5-bromopyrimidin-2-yl)morpholine (123.1mg, 0.5 mmol) and Ni(OAc)$_2$•4H$_2$O (2.5 mg, 2 mol%) as catalyst, and TMPhen (4.73 mg, 4 mol %) as ligand. Chromatography conditions: 7.5-30% EtOAc/hexanes. Yield: 52%, 72.7 mg; Colorless solid; **R$_f$** = 0.43 (30% EtOAc/hexanes).

^{1}H NMR (400 MHz, $CDCl_3$) δ 7.33 – 7.27 (m, 1H), 6.92 – 6.84 (m, 1H), 6.74 (t, *J* = 2.2 Hz, 1H), 6.68 (dd, *J* = 8.3, 2.5 Hz, 1H), 3.36 – 3.26 (m, 4H), 2.13 – 1.99 (m, 4H).

^{13}C NMR (101 MHz, $CDCl_3$) δ 161.90, 157.84, 110.39, 66.95, 44.29.

Spectral data matches that previously reported in the literature.[15]

2-Methoxynaphthalene (17)

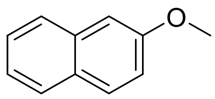

17

Product **17** was prepared according to General Procedure A at 50 °C for 3 h using 2-bromo-6-methoxynaphthalene (96.0 mg, 0.5 mmol) and $Ni(OAc)_2 \bullet 4H_2O$ (5 mg, 4 mol%) as catalyst, and TMPhen (8 mol% 10 mg) as ligand. Chromatography conditions: 1-5% EtOA/hexanes. Yield: 92%, 72.7 mg; white solid; **R_f** = 0.48 (5% EtOAc/hexanes).

^{1}H NMR (500 MHz, $CDCl_3$) δ 7.76 (dd, *J* = 14.5, 8.3 Hz, 3H), 7.45 (dd, *J* = 8.2, 6.8 Hz, 1H), 7.34 (dd, *J* = 8.1, 6.8 Hz, 1H), 7.16 (d, *J* = 8.0 Hz, 2H), 3.93 (s, 3H).

^{13}C NMR (126 MHz, $CDCl_3$) δ 157.74, 134.71, 129.53, 129.10, 127.80, 126.88, 126.51, 123.73, 118.85, 105.89, 77.41, 77.16, 76.91, 55.43.

Spectral data matches that previously reported in the literature.[16]

***N*-(4-Fluorophenyl)-7-methoxy-6-(3-morpholinopropoxy) quinazolin-4-amine (18):**

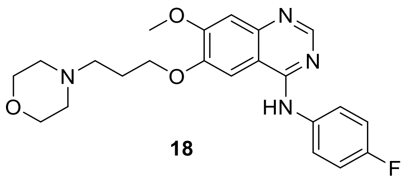

18

Product **18** was prepared according to General Procedure A at 50 °C for 6 h using 4-chloro-1,1'-biphenyl (111.7 mg, 0.25 mmol) and $Ni(OAc)_2$•$4H_2O$ (2.5 mg, 4 mol%) as catalyst, and TMPhen (8 mol% 5 mg) as ligand. Chromatography conditions: 2-5% MeOH/CH_2Cl_2. Yield: 70%, 71.8 mg; white solid; **R_f** = 0.35 (5% MeOH/CH_2Cl_2).

^{1}H NMR (400 MHz, DMSO-d_6) δ 10.34 (s, 1H), 8.61 (s, 1H), 7.94 (s, 1H), 7.74 – 7.55 (m, 3H), 7.36 – 7.21 (m, 2H), 4.20 (t, *J* = 6.4 Hz, 2H), 3.96 (s, 3H), 3.56 (t, *J* = 4.6 Hz, 4H), 2.45 (d, *J* = 7.0 Hz, 2H), 2.38 (d, *J* = 4.7 Hz, 5H), 1.99 (p, *J* = 6.7 Hz, 2H).

^{13}C NMR (101 MHz, DMSO-d_6) δ 161.15, 158.74, 158.11, 155.52, 153.52, 149.28, 140.84, 134.47, 134.44, 126.78, 126.70, 124.92, 124.84, 115.95, 115.72, 108.25, 103.69, 67.82, 66.65, 56.49, 55.34, 53.89, 26.21.

^{19}F NMR (376 MHz, DMSO-d_6) δ -116.77.

HRMS (ESI+): Calcd for $C_{22}H_{26}FN_4O_3$, $[M+H]^+$ 413.1989; found 413.1995.

(*S*)-*N*-(2-Hydroxy-3-((hydroxymethyl)(4-(3-xomorpholino)phenyl)amino)propyl)thiophene-2-carboxamide (19)

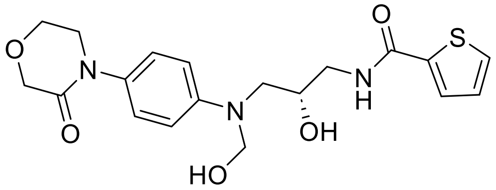

19

Product **19** was prepared according to General Procedure A at 50 °C for 6 h using (*S*)-5-chloro-N-((2-oxo-3-(4-(3-oxomorpholino) phenyl) oxazolidin-5-yl)methyl)thiophene-2-carboxamide (108.97 mg, 0.25 mmol) and $Ni(OAc)_2$•$4H_2O$ (2.5 mg, 4 mol %) as catalyst, and TMPhen (8 mol % 5 mg) as ligand. Chromatography conditions: 5% MeOH/CH_2Cl_2. Yield: 87%, 87.3 mg; thick liquid; **R_f** = 0.30 (5% MeOH/CH_2Cl_2).

^{1}H NMR (400 MHz, DMSO-d$_6$) δ 8.83 (t, *J* = 5.9 Hz, 1H), 7.87 – 7.73 (m, 2H), 7.25 – 7.15 (m, 2H), 7.14 (dd, *J* = 5.0, 3.7 Hz, 1H), 6.66 – 6.51 (m, 2H), 5.47 (t, *J* = 5.8 Hz, 1H), 4.75 (dq, *J* = 8.7, 5.7 Hz, 1H), 4.58 (t, *J* = 5.5 Hz, 1H), 4.10 – 3.95 (m, 1H), 3.73 (dd, *J* = 9.1, 6.0 Hz, 1H), 3.62 – 3.37 (m, 8H), 3.14 (q, *J* = 5.8 Hz, 2H).

^{13}C NMR (126 MHz, DMSO-d$_6$) δ 170.82, 162.26, 154.93, 146.27, 139.86, 131.56, 128.96, 128.42, 127.87, 121.25, 121.05, 112.80, 112.58, 112.41, 112.31, 72.72, 71.52, 69.41, 60.72, 60.24, 48.69, 43.43, 42.76, 40.56, 40.47, 40.40, 40.30, 40.23.

HRMS (ESI+): Calcd for $C_{19}H_{24}N_3O_5S$, $[M+H]^+$ 406.1437; found 406.1452.

2-(3-(Hydroxy(phenyl)methyl)phenoxy)-2-methylpropan-1-ol (20)

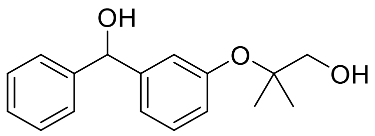

20

Product **20** was prepared according to General Procedure A at 50 °C for 6 h using 2-(3-((4-chlorophenyl)(hydroxy)methyl)phenoxy)-2-methylpropan-1-ol (90.71 mg, 0.25 mmol) and $Ni(OAc)_2$•$4H_2O$ (1.25 mg, 2 mol %) as catalyst, and TMPhen (4 mol % 2.5 mg) as ligand. Chromatography conditions: 30% EtOAc/hexanes. Yield: 95%, 65.0 mg; white solid; **R$_f$** = 0.25 (50% EtOAc/hexanes).

^{1}H NMR (400 MHz, CDCl$_3$) δ 7.42 – 7.30 (m, 4H), 7.30 – 7.22 (m, 3H), 6.98 – 6.85 (m, 2H), 5.82 (d, *J* = 2.0 Hz, 1H), 3.57 (d, *J* = 1.4 Hz, 2H), 2.20 (s, 2H), 1.26 (d, *J* = 0.9 Hz, 7H).

^{13}C NMR (101 MHz, DMSO-d$_6$) δ 154.05, 146.28, 140.83, 128.52, 127.35, 127.10, 126.65, 123.90, 123.83, 80.84, 80.79, 74.36, 68.31, 23.95.

HRMS (ESI+): Calcd for $C_{17}H_{20}NaO_3$, $[M+H]^+$ 295.1310; found 295.1319.

t-Butyl 4-(5,6-dihydro-11H-benzo[5,6]cyclohepta[1,2-b]pyridin-11-ylidene)piperidine-1-carboxylate (21)

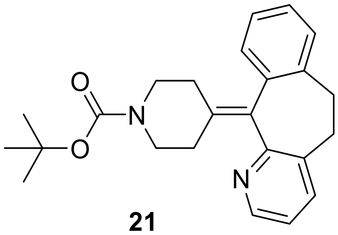

21

Product **21** was prepared according to General Procedure A at 50 °C for 6 h using tert-butyl 4-(8-chloro-5,6-dihydro-11H-benzo[5,6]cyclohepta[1,2-b]pyridin-11-ylidene)piperidine-1-carboxylate (205.44 mg, 0.5 mmol) and $Ni(OAc)_2$•$4H_2O$ (5 mg, 4 mol%) as catalyst, and TMPhen (8 mol % 10 mg) as ligand. Chromatography conditions: 20-40% EtOAc/hexanes. Yield: 82%, 155.2 mg; white solid; $\mathbf{R_f}$ = 0.45 (30% EtOAc/hexanes).

^{1}H NMR (400 MHz, DMSO-d$_6$) δ 8.31 (dd, *J* = 4.7, 1.7 Hz, 1H), 7.55 (dd, *J* = 7.7, 1.7 Hz, 1H), 7.23 – 7.09 (m, 4H), 7.09 – 7.00 (m, 1H), 3.53 (ddd, *J* = 13.3, 9.0, 4.9 Hz, 2H), 3.39 – 3.24 (m, 3H), 3.13 (s, 2H), 2.78 (dddd, *J* = 13.6, 11.0, 8.8, 5.9 Hz, 2H), 2.28 (tdd, *J* = 13.2, 8.8, 4.5 Hz, 2H), 2.13 (dddd, *J* = 17.8, 13.9, 6.1, 3.9 Hz, 2H), 1.37 (s, 9H).

^{13}C NMR (101 MHz, DMSO-d$_6$) δ 157.77, 154.32, 146.83, 146.69, 139.48, 138.13, 137.82, 137.15, 136.15, 135.09, 133.86, 133.70, 132.07, 129.69, 129.37, 127.70, 126.18, 122.82, 122.67, 79.15, 45.13, 31.69, 31.41, 30.84, 30.73, 28.52.

HRMS (ESI+): Calcd for $C_{24}H_{29}N_2O_2$, $[M+H]^+$ 377.2229; found 377.2240.

***N*-(4-(*N*-(Cyclohexylcarbamoyl)sulfamoyl)phenethyl)-2-methoxybenzamide (22)**

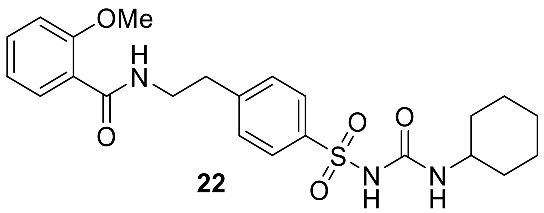

22

Product **22** was prepared according to General Procedure A at 50 °C for 6 h using 5-chloro-*N*-(4-(*N*-(cyclohexylcarbamoyl) sulfamoyl) phenethyl)-2-methoxybenzamide (247 mg, 0.5 mmol) and Ni(OAc)$_2$•4H$_2$O (5 mg, 4 mol %) as catalyst, and TMPhen (8 mol % 9.46 mg) as ligand. Yield: 83%, 189.1 mg; off white solid; **R$_f$** = 0.30 (2% MeOH/CH$_2$Cl$_2$).

^{1}H NMR (400 MHz, DMSO-d$_6$) δ 8.15 (t, *J* = 5.8 Hz, 1H), 7.70 (dd, *J* = 29.0, 7.7 Hz, 3H), 7.43 (t, *J* = 7.8 Hz, 1H), 7.22 (d, *J* = 7.8 Hz, 2H), 7.13 – 6.78 (m, 2H), 5.68 (s, 1H), 3.77 (s, 3H), 3.50 (q, *J* = 6.7 Hz, 2H), 3.19 (s, 1H), 2.82 (t, *J* = 7.2 Hz, 2H), 2.48 (s, 2H), 1.87 – 1.34 (m, 5H), 1.34 – 0.75 (m, 5H).

^{13}C NMR (101 MHz, DMSO-d$_6$) δ 165.24, 161.07, 157.43, 145.89, 141.37, 132.68, 130.92, 128.45, 126.73, 123.24, 120.93, 112.43, 56.26, 48.34, 40.93, 35.18, 33.80, 25.89, 25.32.

HRMS (ESI+): Calcd for $C_{23}H_{29}N_3NaO_5S$, $[M+H]^+$ 482.1726; found 482.1730.

4-Amino-*N*-(2-(Diethylamino)ethyl)-2-methoxybenzamide (23)

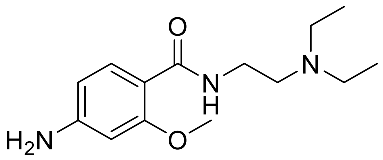

23

Product **23** was prepared according to General Procedure A at 50 °C for 6 h using 4-amino-5-chloro-*N*-(2-(diethylamino)ethyl)-2-methoxybenzamide (88.57 mg, 0.25 mmol) and Ni(OAc)$_2$•4H$_2$O (2.5 mg, 4 mol %) as catalyst, and TMPhen (8 mol % 5 mg) as ligand. Chromatography conditions: 20-2% MeOH/CH$_2$Cl$_2$ and 0.5% NH$_4$OH. Yield: 89%, 59.0 mg; light yellow color thick liquid; **R$_f$** = 0.30 (5% MeOH/CH$_2$Cl$_2$).

¹H NMR (400 MHz, CDCl₃) δ 8.22 (s, 1H), 8.02 (d, *J* = 8.4 Hz, 1H), 6.33 (ddt, *J* = 8.5, 2.0, 0.9 Hz, 1H), 6.19 (d, *J* = 2.1 Hz, 1H), 3.96 (s, 2H), 3.92 – 3.85 (m, 3H), 3.50 (q, *J* = 5.6 Hz, 2H), 2.78 – 2.47 (m, 6H), 1.05 (dt, *J* = 8.5, 4.3 Hz, 6H).

¹³C NMR (101 MHz, CDCl₃) δ 165.57, 159.33, 159.27, 151.22, 150.30, 133.64, 133.33, 111.58, 111.22, 107.63, 107.35, 105.32, 97.15, 95.65, 55.58, 55.44, 51.67, 46.92, 46.74, 39.51, 37.39, 22.32, 14.05, 12.07.

HRMS (ESI+): Calcd for $C_{14}H_{24}N_3O_2$, $[M+H]^+$ 266.1869; found 266.1872.

6'-Methyl-3-(4-(methylsulfonyl)phenyl)-2,3'-bipyridine (24)

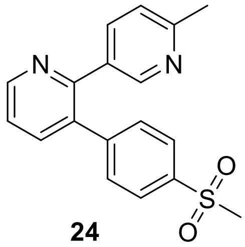

24

Product **24** was prepared according to General Procedure A at 50 °C for 6 h using 5-chloro-6'-methyl-3-(4-(methylsulfonyl)phenyl)-2,3'-bipyridine (89.71 mg, 0.25 mmol) and $Ni(OAc)_2$•$4H_2O$ (2.5 mg, 4 mol %) as catalyst, and TMPhen (8 mol % 5 mg) as ligand. Chromatography conditions: 5% MeOH/CH_2Cl_2. Yield: 79%, 59.0 mg; off white solid; $\mathbf{R_f}$ = 0.30 (2% MeOH/CH_2Cl_2).

¹H NMR (400 MHz, CDCl₃) δ 8.82 (dd, *J* = 4.7, 1.6 Hz, 1H), 8.39 (dd, *J* = 2.4, 0.8 Hz, 1H), 8.02 – 7.91 (m, 3H), 7.62 (ddd, *J* = 7.8, 6.0, 3.5 Hz, 2H), 7.57 (dq, *J* = 8.6, 2.1 Hz, 2H), 7.29 – 7.13 (m, 1H), 3.31 (s, 3H), 2.50 (s, 3H).

¹³C NMR (101 MHz, DMSO-d₆) δ 157.83, 154.06, 149.96, 149.83, 144.91, 140.27, 139.96, 139.31, 137.77, 134.76, 132.76, 130.92, 127.62, 123.38, 122.84, 43.83, 40.63, 40.43, 40.22, 40.01, 39.80, 39.59, 39.38, 24.24.

HRMS (ESI+): Calcd for $C_{18}H_{17}N_2O_2S$, $[M+H]^+$ 325.1011; found 325.1020.

7-(4-(4-Phenylpiperazin-1-yl) butoxy)-3,4-dihydroquinolin-2(1H)-one (25)

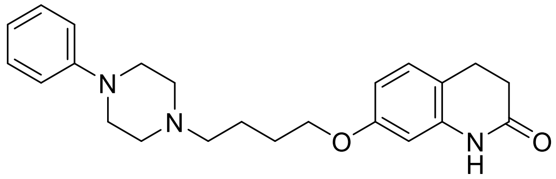

25

Product **25** was prepared according to General Procedure A at 50 °C for 6 h using 7-(4-(4-(2,3-dichlorophenyl)piperazin-1-yl)butoxy)-3,4-dihydroquinolin-2(1H)-one (112.1 mg, 0.25 mmol) and $Ni(OAc)_2$•$4H_2O$ (2.5 mg, 4 mol %) as catalyst, and TMPhen (8 mol % 5 mg) as ligand. Chromatography conditions: 2% MeOH/CH_2Cl_2. Yield: 87%, 82.4 mg; light pink color solid; $\mathbf{R_f}$ = 0.40 (5% MeOH/CH_2Cl_2).

^{1}H NMR (400 MHz, DMSO-d$_6$) δ 9.95 (s, 1H), 7.24 – 7.12 (m, 2H), 7.02 (d, *J* = 8.3 Hz, 1H), 6.95 – 6.80 (m, 2H), 6.74 (tt, *J* = 7.3, 1.1 Hz, 1H), 6.47 (dd, *J* = 8.2, 2.5 Hz, 1H), 6.41 (d, *J* = 2.5 Hz, 1H), 3.90 (t, *J* = 6.4 Hz, 2H), 3.13 – 3.04 (m, 4H), 2.75 (dd, *J* = 8.5, 6.5 Hz, 2H), 2.43 – 2.28 (m, 4H), 1.70 (dq, *J* = 8.4, 6.5 Hz, 2H), 1.57 (qd, *J* = 7.3, 3.8 Hz, 2H).

^{13}C NMR (101 MHz, DMSO-d$_6$) δ 170.75, 158.36, 151.54, 139.67, 129.36, 128.85, 119.19, 115.92, 115.75, 115.43, 108.03, 102.19, 67.78, 57.89, 53.20, 48.68, , 31.24, 27.13, 24.48, 23.23.

HRMS (ESI+): Calcd for $C_{23}H_{30}N_3O_2$, $[M+H]^+$ 380.2338; found 380.2337.

***N*-((4-(Trifluoromethoxy)phenyl)carbamoyl)benzamide (26)**

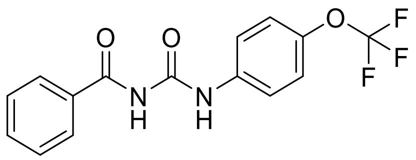

26

Product **26** was prepared according to General Procedure A at 50 °C for 6 h using 2-chloro-N-((4-(trifluoromethoxy)phenyl)carbamoyl)benzamide (170.35 mg, 0.25 mmol) and $Ni(OAc)_2 \cdot 4H_2O$ (5 mg, 4 mol %) as catalyst, and TMPhen (8 mol % 10 mg) as ligand. Purification by EtOAc/hexanes (1:2) wash and centrifugation. Yield: 80%, 130.4 mg; off white solid; **R_f** = 0.25 (30% EtOAc/hexanes).

^{1}H NMR (400 MHz, DMSO-d$_6$) δ 11.09 (s, 1H), 10.90 (s, 1H), 8.17 – 7.90 (m, 2H), 7.67 (dd, *J* = 28.1, 8.0 Hz, 3H), 7.53 (t, *J* = 7.6 Hz, 2H), 7.35 (d, *J* = 8.3 Hz, 2H).

^{13}C NMR (126 MHz, DMSO-d$_6$) δ 169.05, 151.70, 144.40, 137.43, 133.55, 132.74, 129.05, 128.78, 122.25, 121.97, 121.80, 121.63, 119.19, 70.25.

HRMS (ESI+): Calcd for $C_{15}H_{11}F_3N_2NaO_3$, $[M+Na]^+$ 347.0619; found 347.0625.

4-((6-Amino-2-((4-cyanophenyl)amino)pyrimidin-4-yl)oxy)-3,5-dimethylbenzonitrile (27)

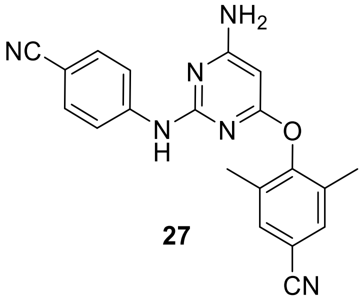

Product **27** was prepared according to General Procedure A at 50 °C for 6 h using 4-((6-amino-5-bromo-2-((4-cyanophenyl)amino)pyrimidin-4-yl)oxy)-3,5-dimethylbenzonitrile (109 mg, 0.25 mmol) and $Ni(OAc)_2 \cdot 4H_2O$ (2.5 mg, 4 mol %) as catalyst, and TMPhen (8 mol % 5 mg) as ligand. Chromatography conditions: 1% MeOH/CH_2Cl_2. Yield: 68%, 61.3 mg; white solid; **R_f** = 0.40 (2% MeOH/CH_2Cl_2).

^{1}H NMR (400 MHz, DMSO-d$_6$) δ 9.52 (s, 1H), 7.81 – 7.59 (m, 4H), 7.44 (d, *J* = 8.5 Hz, 2H), 6.75 (s, 2H), 5.44 (d, *J* = 1.9 Hz, 1H), 2.10 (s, 6H).

^{13}C NMR (101 MHz, DMSO-d$_6$) δ 168.62, 166.87, 159.32, 154.36, 145.78, 133.39, 132.97, 132.91, 120.09, 119.15, 118.46, 108.48, 102.13, 79.12, 16.27, 14.54.

Spectral data matches that previously reported in the literature.[17]

N-(6-((2R,6S)-2,6-Dimethylmorpholino)pyridin-3-yl)-2-methylbenzamide (28)

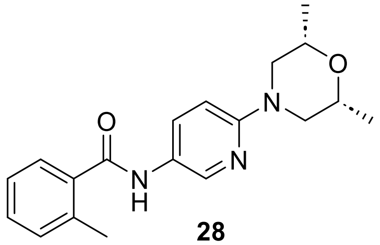

28

Product **28** was prepared according to General Procedure A at 50 °C for 6 h using 3-bromo-*N*-(6-((2R,6S)-2,6-dimethylmorpholino)pyridin-3-yl)-2-methylbenzamide (101.07 mg, 0.25 mmol) and $Ni(OAc)_2 \cdot 4H_2O$ (2.5 mg, 4 mol %) as catalyst, and TMPhen (8 mol % 5 mg) as ligand. Chromatography conditions: 3% MeOH/CH_2Cl_2. Yield: 88%, 71.4 mg; pink color solid; $\mathbf{R_f}$ = 0.45 (2% MeOH/CH_2Cl_2).

^{1}H NMR (400 MHz, $CDCl_3$) δ 8.19 (d, *J* = 2.7 Hz, 1H), 8.05 (dd, *J* = 9.1, 2.8 Hz, 1H), 7.57 – 7.43 (m, 1H), 7.43 – 7.31 (m, 2H), 7.26 (s, 3H), 6.67 (d, *J* = 9.1 Hz, 1H), 4.07 – 3.90 (m, 2H), 3.74 (dqd, *J* = 12.5, 6.2, 2.4 Hz, 2H), 2.59 – 2.41 (m, 5H), 1.27 (d, *J* = 6.3 Hz, 6H).

^{13}C NMR (101 MHz, $CDCl_3$) δ 168.45, 156.68, 140.28, 136.37, 136.22, 136.03, 131.31, 131.14, 130.19, 126.76, 126.01, 125.75, 106.90, 71.55, 51.23, 19.82, 19.19, 18.99.

HRMS (ESI+): Calcd for $C_{19}H_{23}N_3O_2$, $[M+H]^+$ 326.1869; found 326.1879.

11-(4-Methylpiperazin-1-yl)-5H-dibenzo[b,e][1,4]diazepine (29)

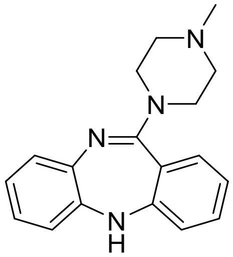

29

Product **29** was prepared according to General Procedure at 50 °C for 6 h using 8-chloro-11-(4-methylpiperazin-1-yl)-5H-dibenzo[b,e][1,4]diazepine (205.4 mg, 0.5 mmol) and $Ni(OAc)_2$•$4H_2O$ (5 mg, 4 mol %) as catalyst, and TMPhen (8 mol % 9.5 mg) as ligand. Chromatography conditions: 20-50% EtOAc/hexanes. Yield: 82%, 155.2 mg; pink color solid; $\mathbf{R_f}$ = 0.45 (30% EtOAc/hexanes).

^{1}H NMR (400 MHz, DMSO-d$_6$) δ 7.29 (td, *J* = 7.6, 1.6 Hz, 1H), 7.17 (dd, *J* = 7.7, 1.6 Hz, 1H), 7.06 (s, 1H), 7.04 – 6.91 (m, 2H), 6.89 – 6.75 (m, 4H), 3.25 (s, 3H), 2.38 (t, *J* = 5.1 Hz, 4H), 2.20 (s, 3H).

^{13}C NMR (101 MHz, CDCl$_3$) δ 162.44, 153.20, 141.85, 140.42, 131.73, 130.30, 130.00, 127.20, 124.28, 123.81, 123.54, 122.80, 120.02, 119.35, 54.96, 47.22, 46.04.

Spectral data matches that previously reported in the literature.[18]

***N*-((Tetrahydrofuran-2-yl)methyl)-5-(trifluoromethyl)pyridin-2-amine (32)**

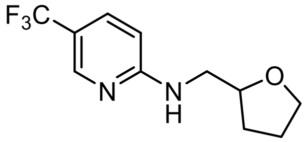

32

Product **32** was prepared according to General Procedure C at 60 °C for 22 h using 3-fluoro-*N*-((tetrahydrofuran-2-yl)methyl)-5-(trifluoromethyl)pyridin-2-amine (61.5 mg, 0.25 mmol) and $Ni(OAc)_2$•$4H_2O$ (3.73 mg, 6 mol %) as catalyst, and TMPhen (7.09 mg, 12 mol %) as ligand. Chromatography conditions: 2-10% EtOAc/hexanes. Yield: 84%, 51.5 mg; white solid; $\mathbf{R_f}$ = 0.26 (10% EtOAc/hexanes).

^{1}H NMR (400 MHz, CDCl$_3$) δ 8.32 (dt, *J* = 2.2, 1.0 Hz, 1H), 7.54 (dd, *J* = 8.8, 2.5 Hz, 1H), 6.43 (dt, *J* = 8.8, 0.7 Hz, 1H), 5.13 (s, 1H), 4.10 (qd, *J* = 7.2, 3.5 Hz, 1H), 3.90 (dt, *J* = 8.3, 6.6 Hz, 1H), 3.78 (dt, *J* = 8.4, 6.8 Hz, 1H), 3.65 (ddd, *J* = 13.4, 6.5, 3.5 Hz, 1H), 3.30 (ddd, *J* = 13.4, 7.5, 4.9 Hz, 1H), 2.03 (dddd, *J* = 11.9, 8.4, 7.0, 5.6 Hz, 1H), 1.97 – 1.88 (m, 2H), 1.69 – 1.59 (m, 1H).

^{13}C NMR (101 MHz, CDCl$_3$) δ 160.50, 146.12, 146.08, 146.04, 145.99, 134.27, 134.24, 134.21, 134.18, 128.80, 126.12, 123.43, 120.74, 115.95, 115.62, 115.30, 114.97, 107.15, 77.70, 77.36, 68.25, 45.69, 29.82, 28.95, 25.94.

^{19}F NMR (376 MHz, CDCl$_3$) δ -61.15.

HRMS (ESI+): Calcd for $C_{11}H_{13}F_3N_2O$, $[M+H]^+$ 247.1058; found 247.1065.

2-(Pyrrolidin-1-yl)-5-(trifluoromethyl)pyridine (33)

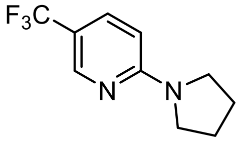

33

Product **33** was prepared according to General Procedure C at 60 °C for 19 h using 3-fluoro-2-(pyrrolidin-1-yl)-5-(trifluoromethyl)pyridine (58.5 mg, 0.25 mmol) and $Ni(OAc)_2$•$4H_2O$ (3.73 mg, 6 mol %) as catalyst, and TMPhen (7.09 mg, 12 mol %) as ligand. Chromatography conditions: 2-10% EtOAc/hexanes + 1% Et_3N. Yield: 90%, 51.5 mg; yellow solid; **R_f** = 0.49 (10% EtOAc/hexanes).

^{1}H NMR (400 MHz, CDCl$_3$) δ 8.40 – 8.35 (m, 1H), 7.57 (dd, *J* = 9.0, 2.5 Hz, 1H), 6.35 (d, *J* = 8.9 Hz, 1H), 3.48 (t, *J* = 6.3 Hz, 5H), 2.03 (dd, *J* = 9.9, 3.5 Hz, 3H)

^{13}C NMR (101 MHz, CDCl$_3$) δ 158.56, 146.25, 146.21, 146.17, 146.12, 133.96, 133.93, 133.90, 133.86, 126.49, 123.80, 113.89, 113.56, 105.76, 77.36, 46.96, 29.85, 25.60.

^{19}F NMR (376 MHz, CDCl$_3$) δ -60.82.

Spectral data matches that previously reported in the literature.[19]

N-(3,4-Dimethoxyphenethyl)-5-(trifluoromethyl)pyridin-2-amine (34)

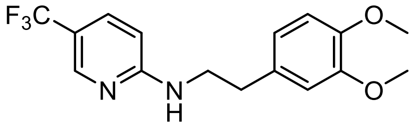

34

Product **34** was prepared according to General Procedure C at 60 °C for 24 h using *N*-(3,4-dimethoxyphenethyl)-3-fluoro-5-(trifluoromethyl)pyridin-2-amine (86.02 mg, 0.25 mmol) and $Ni(OAc)_2 \cdot 4H_2O$ (3.73 mg, 6 mol %) as catalyst, and TMPhen (7.09 mg, 12 mol %) as ligand. Chromatography conditions: 5-20% EtOAc/hexanes + 1% Et_3N. Yield: 84%, 68.5 mg; white solid; $\mathbf{R_f}$ = 0.26 (20% EtOAc/hexanes).

^{1}H NMR (400 MHz, $CDCl_3$) δ 8.34 (dt, *J* = 2.2, 1.1 Hz, 1H), 7.57 (dd, *J* = 8.8, 2.5 Hz, 1H), 6.82 (d, *J* = 8.1 Hz, 1H), 6.79 – 6.70 (m, 2H), 6.37 (d, *J* = 8.8 Hz, 1H), 4.84 (s, 1H), 3.87 (d, *J* = 1.9 Hz, 6H), 3.60 (td, *J* = 6.8, 5.7 Hz, 2H), 2.88 (t, *J* = 6.9 Hz, 2H).

^{13}C NMR (126 MHz, $CDCl_3$) δ 160.33, 149.26, 147.96, 146.36, 146.33, 146.29, 146.26, 134.51, 134.49, 134.46, 134.43, 131.37, 127.98, 125.83, 123.68, 121.53, 120.87, 116.04, 115.77, 115.51, 115.25, 112.09, 111.58, 106.36, 56.10, 56.03, 43.26, 35.17.

^{19}F NMR (471 MHz, $CDCl_3$) δ -61.12.

HRMS (ESI+): Calcd for $C_{16}H_{17}F_3N_2O_2$, $[M+H]^+$ 327.1320; found 327.1331.

t-Butyl 4-(5-(trifluoromethyl)pyridin-2-yl)piperazine-1-carboxylate (35)

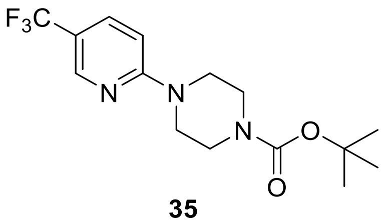

35

Product **35** was prepared according to General Procedure A at 60 °C for 16 h using *t*-butyl 4-(3-chloro-5-(trifluoromethyl)pyridin-2-yl)piperazine-1-carboxylate (87.26 mg, 0.25 mmol) and $Ni(OAc)_2 \cdot 4H_2O$ (2.5 mg, 4 mol %) as catalyst, and TMPhen (8 mol % 5 mg) as ligand. Chromatography conditions: 10% EtOAc/hexanes. Yield: 96%, 79.4 mg; light pink color solid; $\mathbf{R_f}$ = 0.5 (20% EtOAc/hexanes).

^{1}H NMR (400 MHz, $CDCl_3$) δ 8.38 (h, *J* = 0.9 Hz, 1H), 7.73 – 7.50 (m, 1H), 6.62 (d, *J* = 9.0 Hz, 1H), 3.85 – 3.03 (m, 8H), 1.47 (s, 9H).

^{13}C NMR (126 MHz, $CDCl_3$) δ 160.23, 154.74, 145.82, 145.78, 145.75, 145.71, 134.65, 134.62, 134.59, 134.57, 127.75, 125.60, 123.45, 121.30, 115.92, 115.66, 115.40, 115.14, 105.65, 80.17, 77.29, 77.03, 76.78, 44.49, 43.64, 42.73, 28.41.

^{19}F NMR (376 MHz, $CDCl_3$) δ -61.25.

Spectral data matches that previously reported in the literature.[18]

HRMS (ESI+): Calcd for $C_{15}H_{20}F_3N_3O_2$, $[M+H]^+$ 332.1586; found 332.1595.

4-(4-(*bis*(4-Fluorophenyl)methyl)piperazin-1-yl)-6-ethylpyrimidine (36)

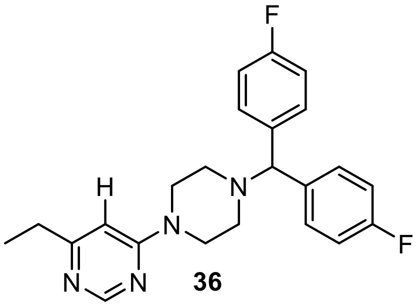

36

Product **36** was prepared according to General Procedure A at 60 °C for 23 h using 4-(4-(*bis*(4-fluorophenyl)methyl)piperazin-1-yl)-6-ethyl-5-fluoropyrimidine (86.02 mg, 0.25 mmol) and $Ni(OAc)_2•4H_2O$ (3.73 mg, 6 mol %) as catalyst, and TMPhen (7.09 mg, 12 mol %) as ligand. Chromatography conditions: 5-20% EtOAc/hexanes + 1% Et_3N. Yield: 63%, 68.5 mg; white solid; $\mathbf{R_f}$ = 0.13 (20% EtOAc/hexanes).

^{1}H NMR (400 MHz, CDCl$_3$) δ 8.52 (d, *J* = 1.1 Hz, 1H), 7.42 – 7.33 (m, 4H), 7.05 – 6.95 (m, 4H), 6.30 (d, *J* = 1.2 Hz, 1H), 4.25 (s, 1H), 3.63 (t, *J* = 5.1 Hz, 4H), 2.60 (q, *J* = 7.6 Hz, 2H), 2.49 – 2.37 (m, 4H), 1.24 (t, *J* = 7.6 Hz, 5H).

^{13}C NMR (101 MHz, CDCl$_3$) δ 170.36, 163.28, 162.33, 160.84, 158.11, 137.91, 137.87, 129.41, 129.33, 115.82, 115.61, 100.25, 77.36, 74.53, 51.51, 44.05, 31.22, 29.84, 13.06.

^{19}F NMR (376 MHz, CDCl$_3$) δ -115.22 (ddd, *J* = 14.1, 8.8, 5.3 Hz).

HRMS (ESI+): Calcd for $C_{23}H_{24}F_2N_4$, $[M+H]^+$ 395.2047; found 395.2049

t-Butyl 4-(5-(trifluoromethyl)pyridin-2-yl-3-d)piperazine-1-carboxylate (39)

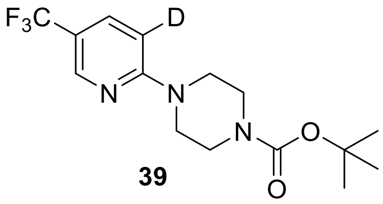

39

Product **39** was prepared according to General Procedure D at 60 °C for 2 h, then 16 at rt. using *t*-butyl 4-(3-chloro-5-(trifluoromethyl)pyridin-2-yl)piperazine-1-carboxylate (91.27 mg, 0.25 mmol) and $Ni(OAc)_2•4H_2O$ (2.5 mg, 4 mol %) as catalyst, and TMPhen (4 mol % 5 mg) as ligand. Chromatography conditions: 15% EtOAc/hexanes. Yield: 99%, 82.1 mg; white solid; $\mathbf{R_f}$ = 0.25 (10% EtOAc/hexanes).

^{1}H NMR (400 MHz, CDCl$_3$) δ 8.40 (dt, *J* = 2.5, 1.0 Hz, 1H), 7.64 (d, *J* = 2.5 Hz, 1H), 3.83 – 3.39 (m, 8H), 1.49 (s, 9H).

^{13}C NMR (101 MHz, $CDCl_3$) δ 160.21, 154.74, 145.83, 145.79, 145.75, 145.71, 134.55, 134.52, 134.49, 134.46, 128.56, 125.87, 123.18, 120.50, 116.01, 115.68, 115.36, 115.03, 105.63, 105.38, 105.13, 80.17, 44.48, 43.47, 28.60, 28.41.

^{19}F NMR (376 MHz, $CDCl_3$) δ -61.18.

Spectral data matches that previously reported in the literature.[20]

HRMS (ESI+): Calcd for C15H19DF3N3O2, $[M+H]^+$ 333.1649; found 333.1653.

11-(4-Methylpiperazin-1-yl)-5H-dibenzo[b,e][1,4]diazepine-8-d (40)

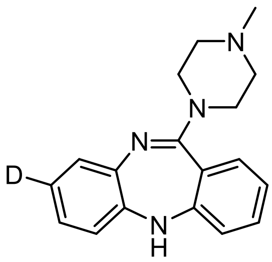

40

Product **40** was prepared according to General Procedure D at 50 °C for 2 h, then 14 h at rt using 8-chloro-11-(4-methylpiperazin-1-yl)-5H-dibenzo[b,e][1,4]diazepine (81.705 mg, 0.25 mmol) and $Ni(OAc)_2$•$4H_2O$ (2.5 mg, 4 mol %) as catalyst, and TMPhen (4.73 mg, 8 mol %) as ligand. Chromatography conditions: 5% MeOH/CH_2Cl_2. Yield: 86%, 79.4 mg; off yellow solid; $\mathbf{R_f}$ = 0.26 (5% MeOH/CH_2Cl_2).

^{1}H NMR (400 MHz, $CDCl_3$) δ 7.27 (td, *J* = 7.5, 1.4 Hz, 2H), 7.08 (d, *J* = 1.5 Hz, 1H), 6.99 (td, *J* = 7.6, 1.1 Hz, 1H), 6.90 – 6.85 (m, 1H), 6.82 (dt, *J* = 7.2, 1.2 Hz, 1H), 6.69 (d, *J* = 7.8 Hz, 1H), 4.92 (s, 1H), 3.47 (d, *J* = 8.4 Hz, 4H), 2.51 (t, *J* = 5.2 Hz, 4H), 2.35 (s, 3H).

^{13}C NMR (101 MHz, $CDCl_3$) δ 162.55, 153.29, 141.90, 140.59, 131.79, 130.44, 127.34, 127.23, 124.40, 123.86, 123.75, 122.90, 120.10, 119.42, 77.36, 55.23, 53.56, 47.49, 46.32.

HRMS (ESI+): Calcd for $C_{18}H_{19}DN_4$ $[M+H]^+$ 294.1829; found 294.1820

2-Methoxynaphthalene-6-d (41)

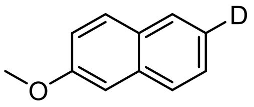

41

Product **41** was prepared according to General Procedure D at 50 °C for 6 h using 2-bromo-6-methoxynaphthalene (59.27 mg, 0.25 mmol) and $Ni(OAc)_2{\bullet}4H_2O$ (2.5 mg, 4 mol %) as catalyst, and TMPhen (4.73 mg, 8 mol %) as ligand. Chromatography conditions: 100% EtOAc/hexanes. Yield: 99%, 39.8 mg; white solid; $\mathbf{R_f}$ = 0.48 (100% EtOAc/hexanes.

^{1}H NMR (500 MHz, $CDCl_3$) δ 7.90 – 7.83 (m, 3H), 7.56 (d, *J* = 8.2 Hz, 1H), 7.27 (d, *J* = 9.6 Hz, 2H), 4.04 (s, 3H).

^{13}C NMR (126 MHz, $CDCl_3$) δ 157.73, 134.71, 129.52, 129.09, 127.68, 126.87, 126.40, 123.64, 123.44, 123.25, 118.85, 105.89, 77.36, 55.42.

HRMS (ESI+): Calcd for $C_{11}H_9DO$, $[M+H]^+$ 160.0873; found 160.0875

4-((Phenyl-4-d)sulfonyl)morpholine (42)

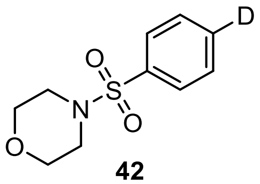

42

Product **42** was prepared according to General Procedure D at 50 °C for 6 h using 4-((4-bromophenyl)sulfonyl)morpholine (76.54 mg, 0.25 mmol) and $Ni(OAc)_2{\bullet}4H_2O$ (2.5 mg, 4 mol %) as catalyst, and TMPhen (4.73 mg, 8 mol %) as ligand. Chromatography conditions: 5-30% EtOAc/hexanes. Yield: 97%, 55.6 mg; white solid; $\mathbf{R_f}$ = 0.56 (30% EtOAc/hexanes.

^{1}H NMR (400 MHz, $CDCl_3$) δ 7.79 – 7.72 (m, 2H), 7.56 (d, *J* = 7.9 Hz, 2H), 3.77 – 3.70 (m, 4H), 3.03 – 2.97 (m, 4H).

^{13}C NMR (126 MHz, CDCl$_3$) δ 147.43, 147.20, 146.98, 138.91, 138.71, 138.50, 135.15, 133.09, 132.89, 132.70, 129.13, 127.91, 125.13, 124.93, 124.72, 77.36, 66.16, 46.08.

HRMS (ESI+): Calcd for $C_{10}H_{12}DNO_3S$, $[M+Na]^+$ 251.0577; found 251.0583

2-Methoxynaphthalene (17)

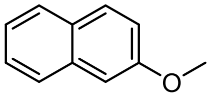

17

Product **17** was prepared according to General Procedure A at 45 °C for 3 h using 2-bromo-6-methoxynaphthalene (59.0 mg, 0.25 mmol) and $Ni(OAc)_2$•$4H_2O$ (2.5 mg, 4 mol %) as catalyst, and TMPhen (4.73 mg, 8 mol %) as ligand. Chromatography conditions: 100% hexanes. Yield: 93%, 36.7 mg; white solid; **R_f** = 0.49 (100% hexanes).

^{1}H NMR (500 MHz, CDCl$_3$) δ 7.77 (dd, *J* = 14.8, 8.4 Hz, 3H), 7.46 (t, *J* = 7.7 Hz, 1H), 7.35 (t, *J* = 7.9 Hz, 1H), 7.17 (d, *J* = 10.6 Hz, 2H), 3.94 (s, 3H).

^{13}C NMR (126 MHz, CDCl$_3$) δ 157.74, 134.71, 129.53, 129.10, 127.80, 126.87, 126.51, 123.72, 118.85, 105.89, 77.36, 55.42.

Spectral data matches that previously reported in the literature.[20]

1-Phenyl-1H-pyrrole (43)

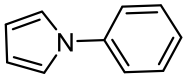

43

Product **43** was prepared according to General Procedure A at 45 °C for 6 h using 1-(4-chlorophenyl)-1H-pyrrole (35.7 mg, 0.25 mmol) and $Ni(OAc)_2$•$4H_2O$ (2.5 mg, 4 mol %) as catalyst, and TMPhen (4.73 mg, 8 mol %) as ligand. Chromatography conditions: 0-50% EtOAc/hexanes. Yield: 91%, 33.3 mg; white solid; **R_f** = 0.26 (50% EtOAc/hexanes).

^{1}**H NMR (500 MHz, CDCl$_3$)** δ 7.47 – 7.37 (m, 4H), 7.25 (td, *J* = 6.9, 1.8 Hz, 1H), 7.10 (t, *J* = 2.2 Hz, 2H), 6.36 (t, *J* = 2.2 Hz, 2H).

^{13}**C NMR (126 MHz, CDCl$_3$)** δ 140.92, 129.68, 125.75, 120.68, 119.46, 110.52, 77.36, 22.49, 14.21.

Spectral data matches that previously reported in the literature.[21]

4-(Pyridin-2-yl)morpholine (44)

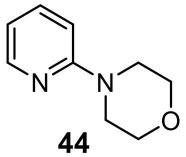

44

Product **44** was prepared according to General Procedure C at 65 °C for 12 h using 4-(3-fluoropyridin-2-yl)morpholine (36 μL, 0.25 mmol) and Ni(OAc)$_2$•4H$_2$O (2.5 mg, 4 mol %) as catalyst, and TMPhen (4.73 mg, 8 mol %) as ligand. Chromatography conditions: 10-40% EtOAc//hexanes. Yield: 74%, 23.0 mg; clear oil; **R$_f$** = 0.32 (20% EtOAc/ hexanes).

^{1}**H NMR (500 MHz, CDCl$_3$)** δ 8.20 (dd, *J* = 5.1, 2.0 Hz, 1H), 7.50 (ddd, *J* = 8.8, 7.1, 2.0 Hz, 1H), 6.69 – 6.61 (m, 2H), 3.85 – 3.80 (m, 4H), 3.52 – 3.47 (m, 4H).

^{13}**C NMR (126 MHz, CDCl$_3$)** δ 159.76, 148.11, 137.67, 113.96, 107.08, 66.92, 45.76, 29.83.

Spectral data matches that previously reported in the literature.[22]

(5-(Methylsulfonyl)-2-((1,1,1-trifluoropropan-2-yl)oxy)phenyl)(4-(5)(trifluoromethyl)pyridin-2-yl-3-d)piperazin-1-yl)methanone (54)

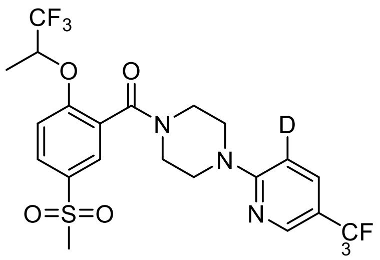

54

Product **54** was prepared according to the synthesis of deuteron-*rac*-bitopertin. Chromatography conditions: 0-100% EtOAc/hexanes, followed by pentane trituration; Yield: 44%, 36.6 mg; white crystallin solid; $\mathbf{R_f}$ = 0.25 (80% EtOAc/ hexanes).

^{1}H NMR (400 MHz, $CDCl_3$) δ 8.47 – 8.31 (m, 1H), 8.06 – 7.82 (m, 2H), 7.66 (d, *J* = 2.6 Hz, 1H), 7.13 (dd, *J* = 17.6, 8.7 Hz, 1H), 4.83 (tp, *J* = 12.3, 6.0 Hz, 1H), 4.01 – 3.83 (m, 2H), 3.83 – 3.52 (m, 4H), 3.35 (dp, *J* = 13.4, 4.6 Hz, 2H), 3.06 (s, 3H), 1.51 (d, *J* = 6.4 Hz, 3H).

^{13}C NMR (101 MHz, $CDCl_3$) δ 165.16, 164.93, 159.91, 156.24, 145.87, 145.83, 145.78, 145.74, 135.38, 135.20, 134.73, 134.70, 130.51, 128.93, 128.46, 128.36, 125.76, 125.40, 123.08, 122.61, 120.39, 119.81, 116.22, 115.88, 114.38, 114.06, 105.78, 105.53, 77.25, 73.80, 73.47, 73.15, 72.82, 46.34, 46.24, 44.69, 44.66, 44.52, 44.41, 41.56, 29.70, 13.94, 13.71.

^{19}F NMR (376 MHz, $CDCl_3$) δ -61.23, -78.05, -78.58.

HRMS (ESI+): Calcd for $C_{12}H_{20}DF_6N_3O_4S$, $[M+H]^+$ 527.1298; found 527.1302

8. References

[1] N.R. Lee, M. Cortes-Clerget, A. B. Wood, D. J. Lippincott, H. Pang, F. A. Moghadam, F. Gallou, B. H. Lipshutz, *ChemSusChem* **2019**, *12*, 3159–3165.

[2] I. Hermans, J. Peeters, P. A. Jacobs, *J. Org. Chem.* **2007**, *72*, 3057–3064.

[3] D. A. Abramowicz, *Critical Reviews in Biotechnology* **1990**, *10*, 241–251.

[4] B. R. Penfold, J. C. B. White, *Acta Cryst* **1959**, *12*, 130–135.

[5] PatentPak can be found under https://scifinder-n.cas.org/patent-viewer?docuri=2te67hCqm_h5sLvsJ2JBscZgwtjj9AuppY9-mEsSkBM&markedFullTextKey=C0AE3PkkhC_wnDLyAotYySvXMTeap2vcAwB12TfRidE.pdf&fullTextKey=gVAHPIRnYjb1SB2PaVYhqRbR0cgR7E-SMCNjKs5TmlY.pdf (accessed 2024-12-10).

[6] P. Dani, T. Karlen, R. A. Gossage, S. Gladiali, G. van Koten, *Angew. Che., Int. Ed.,* **2000**, *39*, 743–745.

[7] B. S. Lane, M. A. Brown, D. Sames, *J. Am. Chem. Soc*. **2005,** *127*, 8050–8057.

[8] Y. M. Chung, W. S. Ahn, P. K. Lim, *Journal of Catalysis*. **1998,** *173*, 210–218.

[9] G. R. Humphrey, J. T. Kuethe, *Chem. Rev.* **2006**, *106*, 2875–2911.

[10] M. P. Mertes, J. Zielinski, C. Pillar, J. Med. Chem. **1967**, *10*, 320‐325.

[11] G. Chelucci, S. Figus, *J.Mol. Catal. A: Chemical.* **2014**, *393*, 191-209.

[12] L. C. Marr, T. W. Kirchstetter, R. A. Harley, A. H. Miguel, S. V. Hering, S. K. Hammond, *Environ. Sci. Technol.* **1999**, *33*, 3091–3099.

[13] Z. Abdullah, N. M. Tahir, M. R. Abas, Z. Aiyub, B. K. Low, *Molecules.* **2004**, *9,* 520–526.

[14] M. H. S. A. Hamid, C. L. Allen, G. W. Lamb, A. C. Maxwell, H. C. Maytum, A. J. A. Watson, J. M. J. Williams, *J. Am. Chem. Soc.* **2009**, *131*, 1766–1774.

[15] K. Walsh, H. F. Sneddon, C. J. Moody, *ChemSusChem.* **2013**, *6,* 1455–1460.

[16] E. Fromentin, J.-M. Coustard, M. Guisnet, *J. Catal.* **2000**, *190*, 433–438.

[17] A. F. Chmiel, O. P. Williams, C. P. Chernowsky, C. S. Yeung, Z. K. Wickens, *J. Am. Chem. Soc.* **2021**, *143*, 10882–10889.

[18] Y. Nagai, N. Miyakawa, H. Takuwa, Y. Hori, K. Oyama, B. Ji, M. Takahashi, X.-P. Huang, S. T. Slocum, J. F. DiBerto, Y. Xiong, T. Urushihata, T. Hirabayashi, A. Fujimoto, K. Mimura, J. G. English, J. Liu, K. Inoue, K. Kumata, C. Seki, M. Ono, M. Shimojo, M.-R. Zhang, Y. Tomita, J. Nakahara, T. Suhara, M. Takada, M. Higuchi, J. Jin, B. L. Roth, T. Minamimoto, *Nat Neurosci.* **2020**, *23*, 1157–1167.

[19] N. Chatani, T. Asaumi, T. Ikeda, S. Yorimitsu, Y. Ishii, F. Kakiuchi, S. Murai, *J. Am. Chem. Soc.* **2000**, *122,* 12882–12883.

[20] B. Sahoo, A.-E. Surkus, M.-M. Pohl, J. Radnik, M. Schneider, S. Bachmann, M. Scalone, K. Junge, M. Beller, *Angew. Chem., Int. Ed.* **2017,** *56*, 11242–11247.

[21] J. Ke, H. Wang, L. Zhou, C. Mou, J. Zhang, L. Pan, Y. R. Chi, *Chem. Euro. J.* **2019**, *25*, 6911–6914.

[22] J. P. Wolfe, H. Tomori, J. P. Sadighi, J. Yin, S. L. Buchwald, *J. Org. Chem.* **2000**, *65,* 1158–1174.

9. NMR Spectra

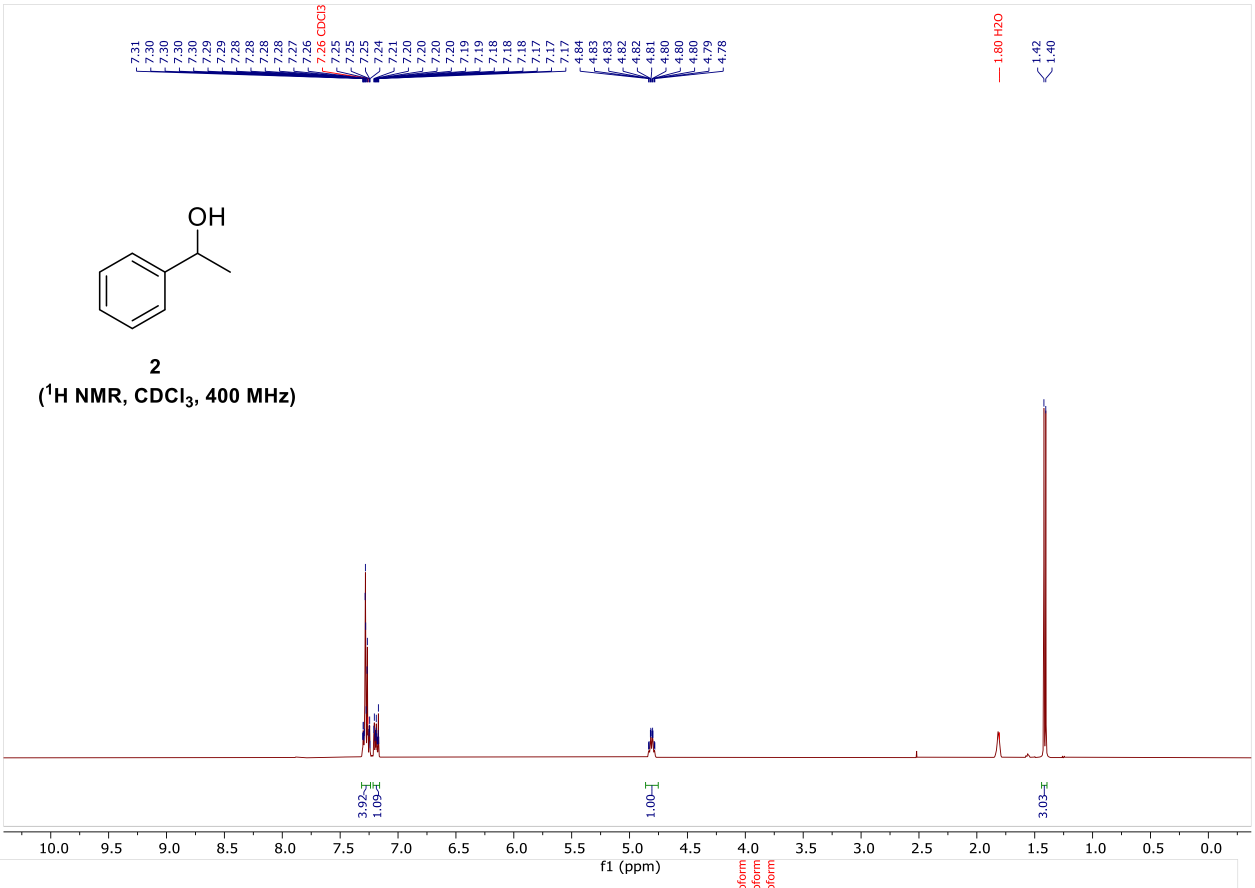
OH
2
(^{1}H NMR, $CDCl_3$, 400 MHz)
7.31 7.30 7.30 7.30 7.30 7.29 7.29 7.28 7.28 7.28 7.28 7.27 7.26 7.26 CDCl3 7.25 7.25 7.25 7.24 7.21 7.20 7.20 7.20 7.20 7.19 7.19 7.18 7.18 7.18 7.17 7.17 7.17
4.84 4.83 4.83 4.82 4.82 4.81 4.80 4.80 4.80 4.79 4.78
1.80 H2O
1.42 1.40
3.92 1.09 1.00 3.03
f1 (ppm)
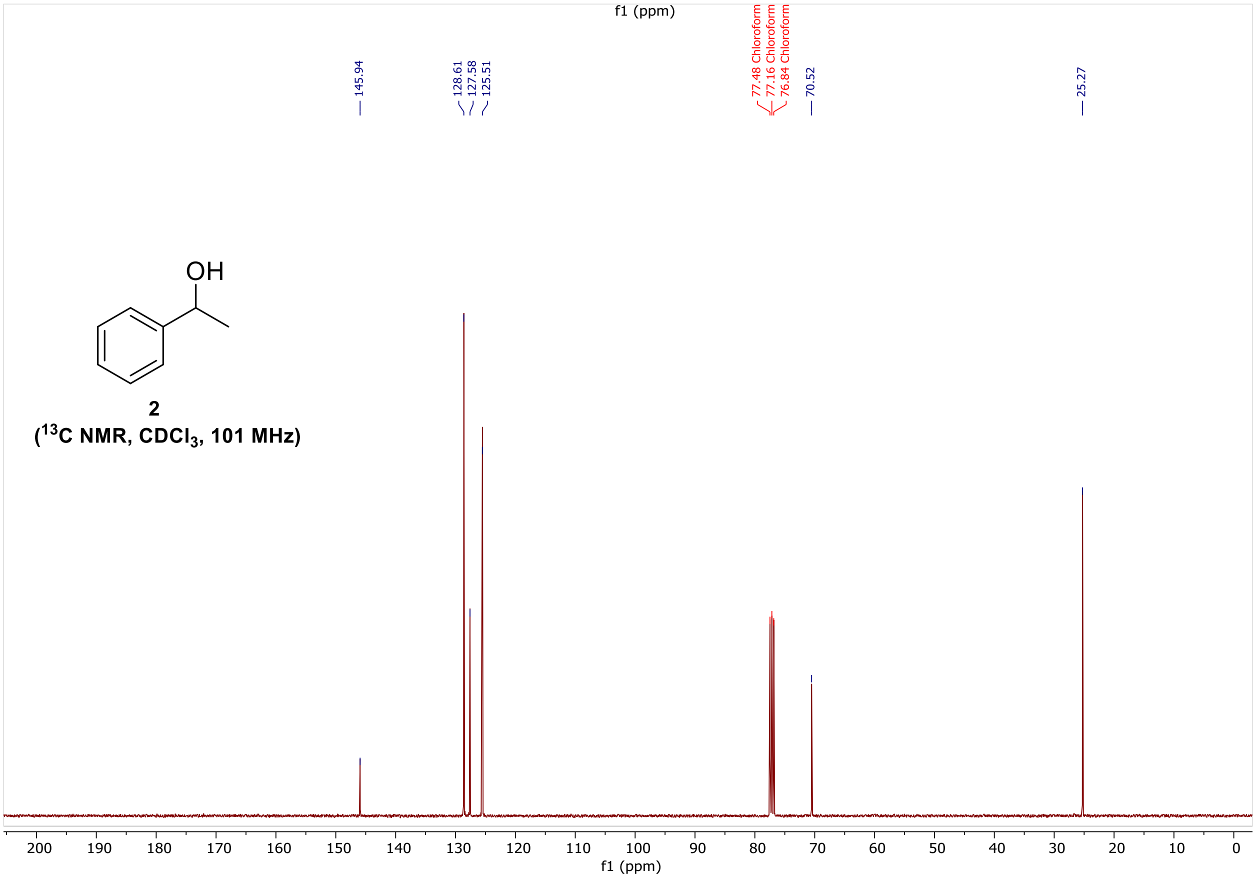
OH
2
(^{13}C NMR, $CDCl_3$, 101 MHz)
145.94
128.61 127.58 125.51
77.48 Chloroform 77.16 Chloroform 76.84 Chloroform
70.52
25.27
f1 (ppm)

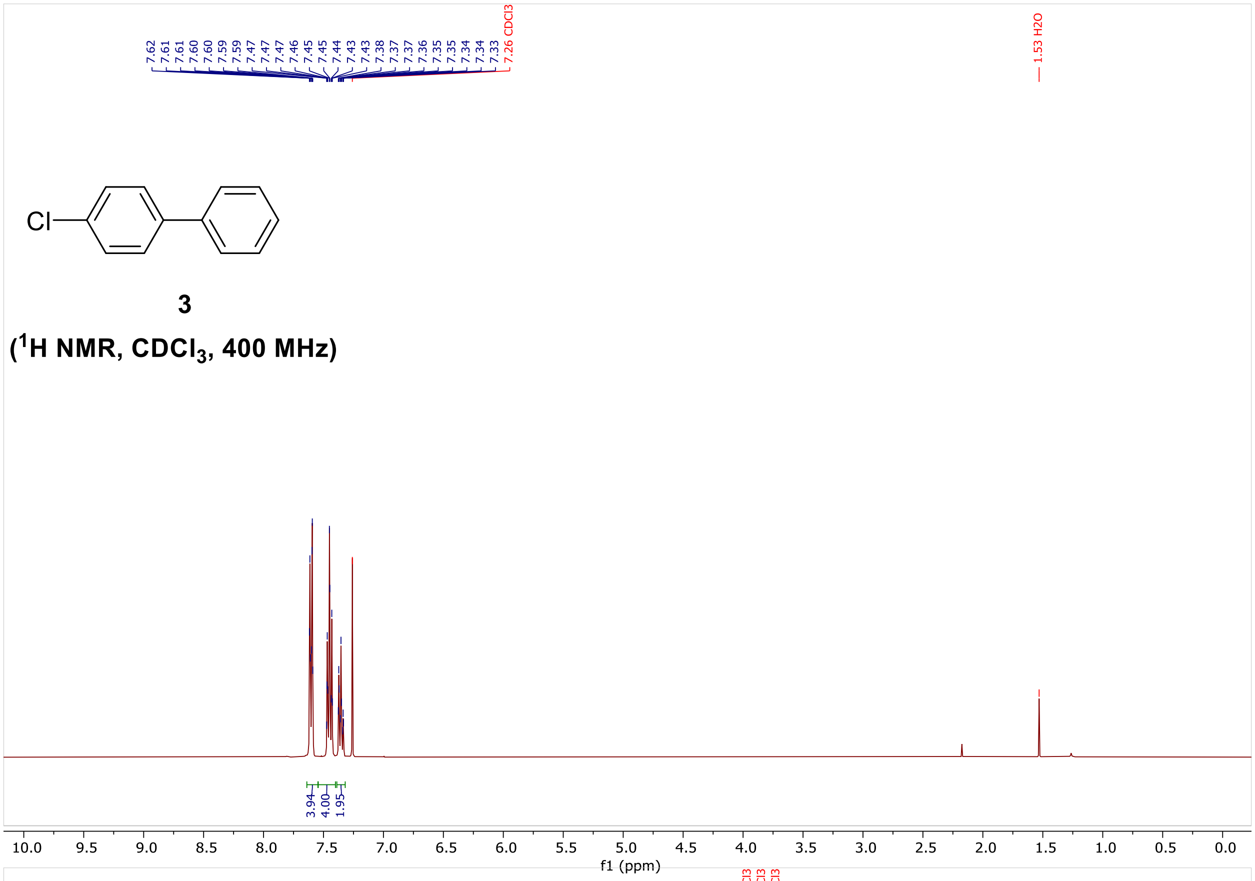
7.62
7.61
7.61
7.60
7.60
7.59
7.59
7.47
7.47
7.47
7.46
7.45
7.45
7.44
7.43
7.43
7.38
7.37
7.37
7.36
7.35
7.35
7.34
7.34
7.33
7.26 CDCl3
1.53 H2O
Cl
3
(1H NMR, CDCl3, 400 MHz)
3.94
4.00
1.95
10.0
9.5
9.0
8.5
8.0
7.5
7.0
6.5
6.0
5.5
5.0
4.5
4.0
3.5
3.0
2.5
2.0
1.5
1.0
0.5
0.0
f1 (ppm)

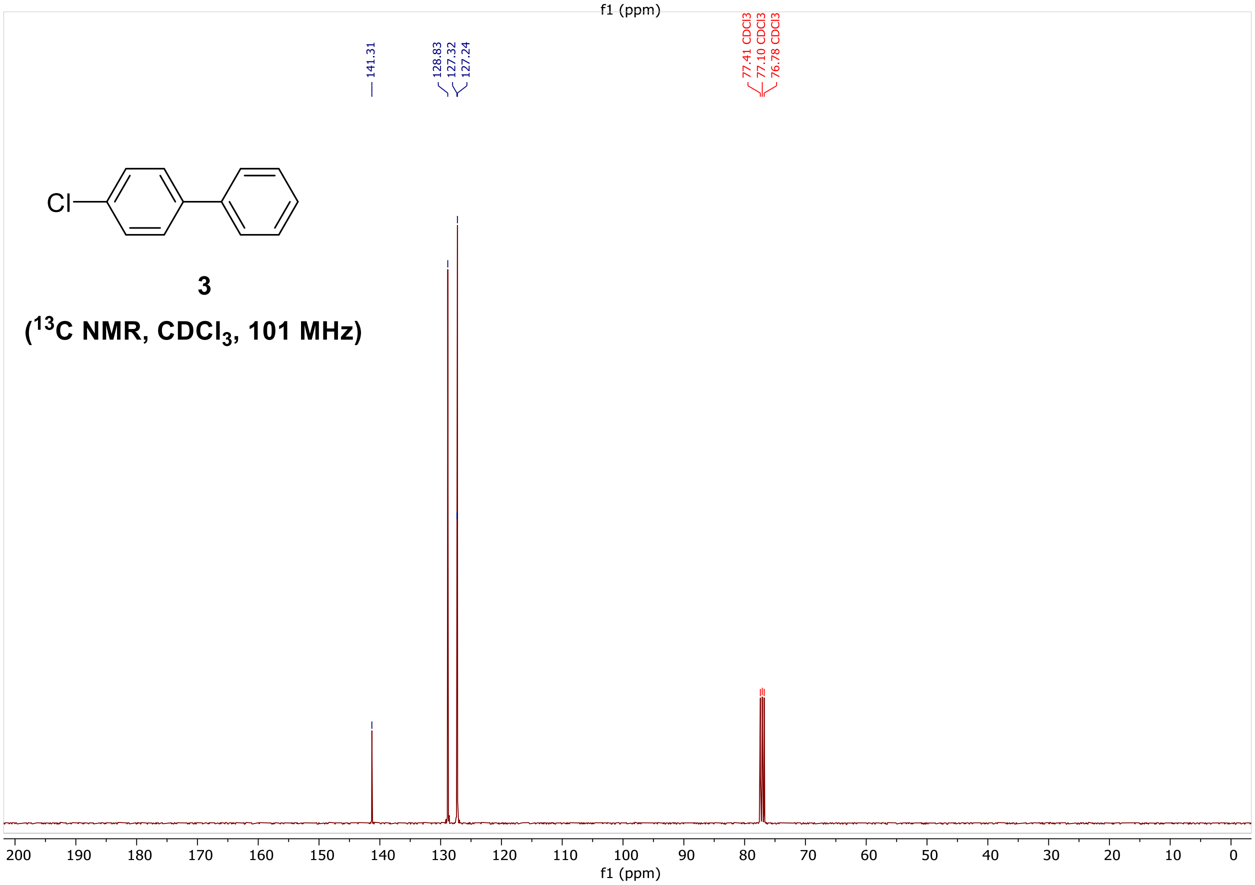
141.31
128.83
127.32
127.24
77.41 CDCl3
77.10 CDCl3
76.78 CDCl3
Cl
3
(13C NMR, CDCl3, 101 MHz)
200
190
180
170
160
150
140
130
120
110
100
90
80
70
60
50
40
30
20
10
0
f1 (ppm)

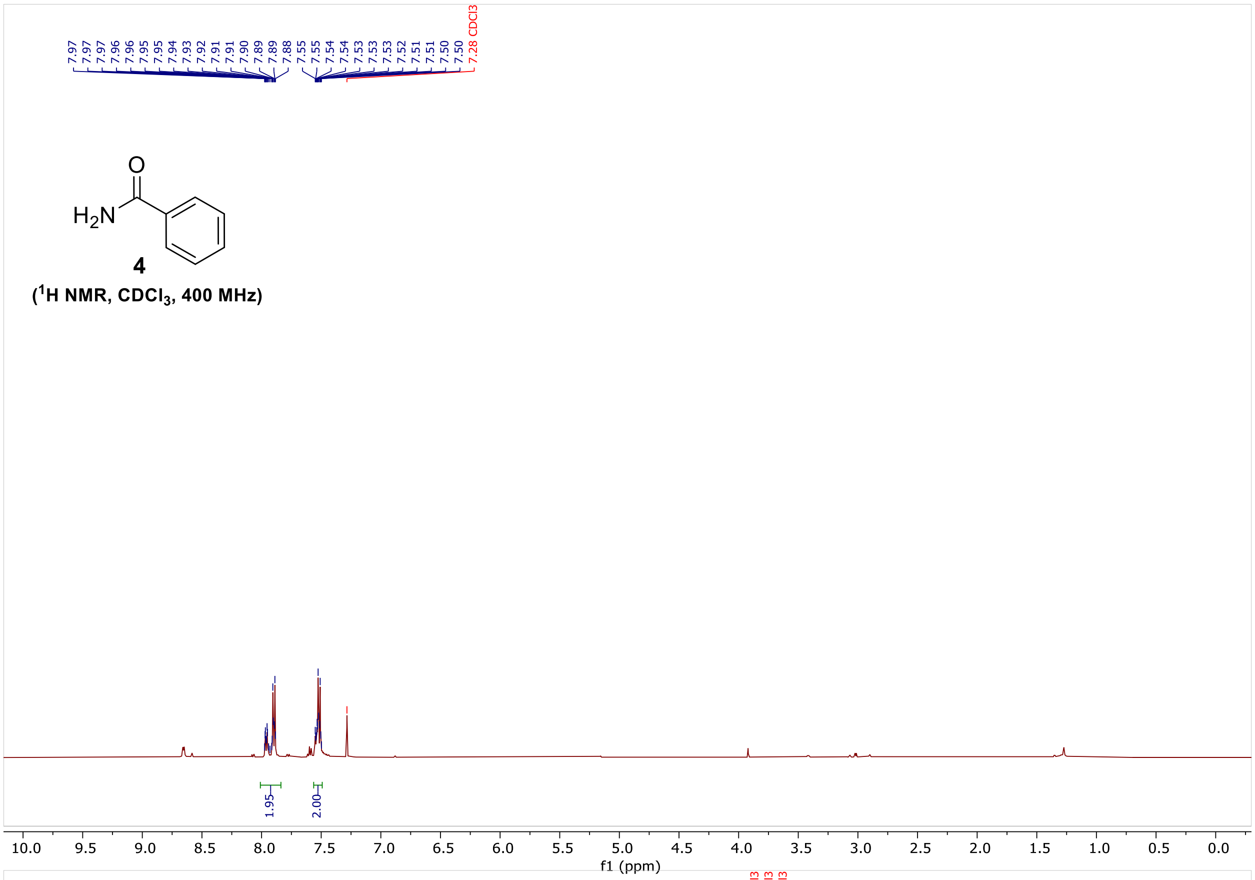
7.97
7.97
7.97
7.96
7.96
7.95
7.95
7.94
7.93
7.92
7.91
7.91
7.90
7.89
7.89
7.88
7.55
7.55
7.54
7.54
7.53
7.53
7.53
7.52
7.51
7.51
7.50
7.50
7.28 CDCl3
H2N
O
4
(1H NMR, CDCl3, 400 MHz)
1.95
2.00
10.0 9.5 9.0 8.5 8.0 7.5 7.0 6.5 6.0 5.5 5.0 4.5 4.0 3.5 3.0 2.5 2.0 1.5 1.0 0.5 0.0
f1 (ppm)

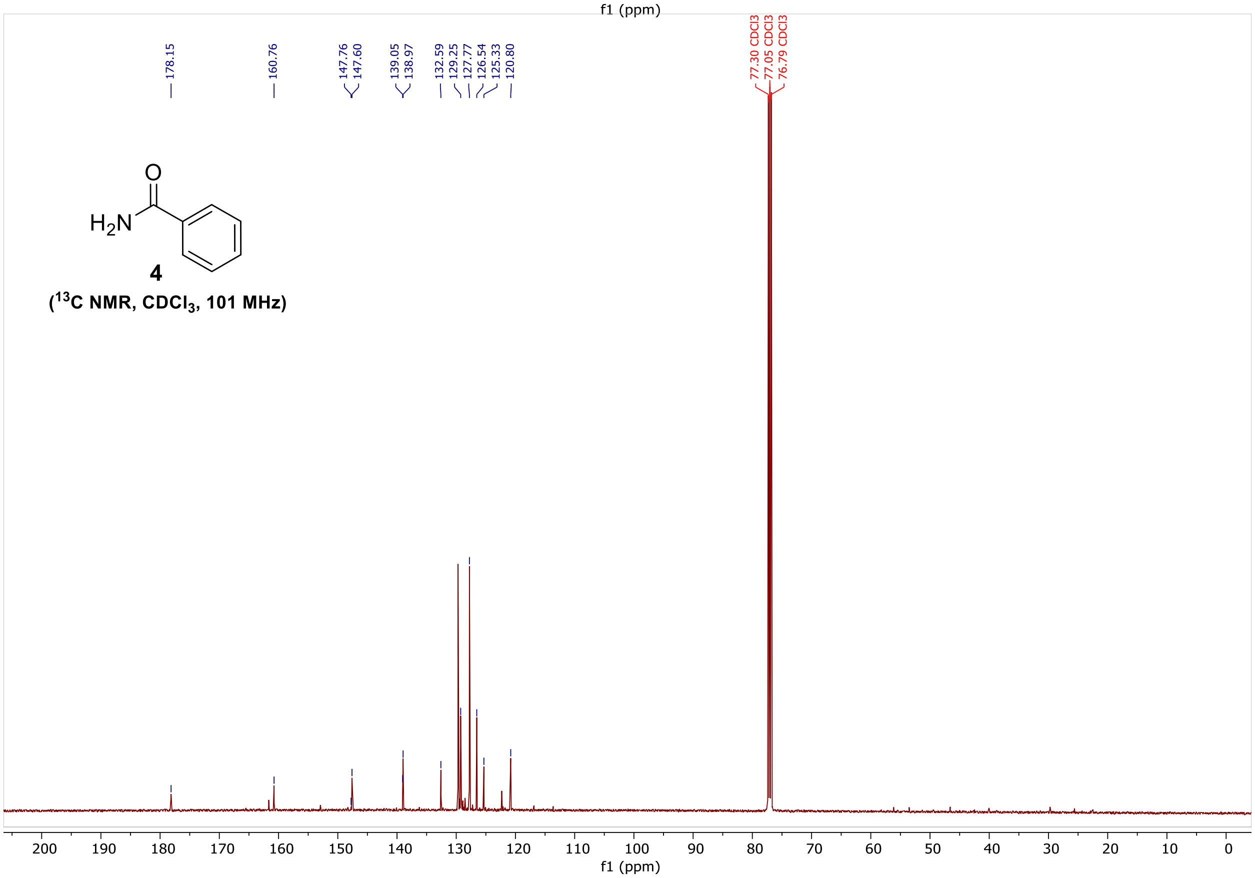
178.15
160.76
147.76
147.60
139.05
138.97
132.59
129.25
127.77
126.54
125.33
120.80
77.30 CDCl3
77.05 CDCl3
76.79 CDCl3
H2N
O
4
(13C NMR, CDCl3, 101 MHz)
200 190 180 170 160 150 140 130 120 110 100 90 80 70 60 50 40 30 20 10 0
f1 (ppm)

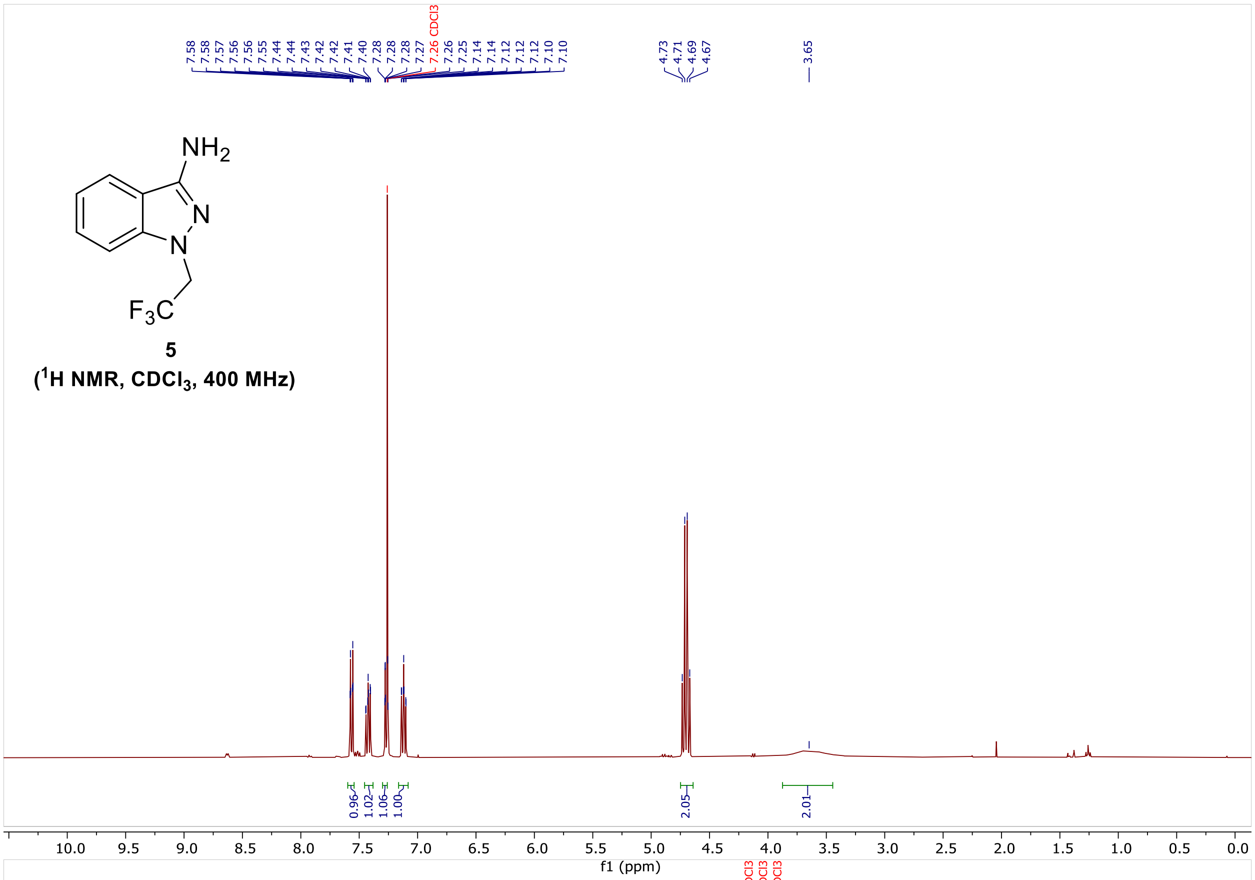

5

(^{1}H NMR, $CDCl_3$, 400 MHz)

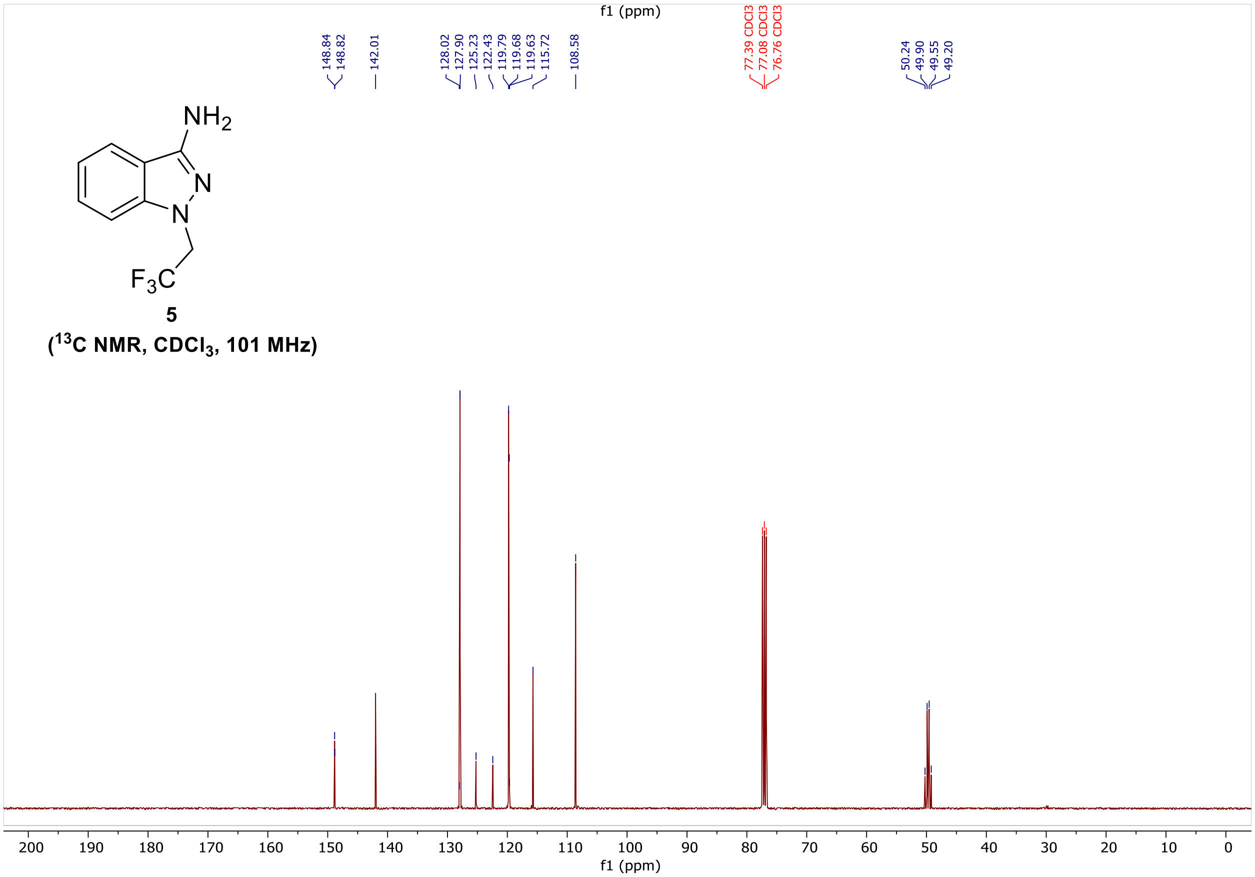

5

(^{13}C NMR, $CDCl_3$, 101 MHz)

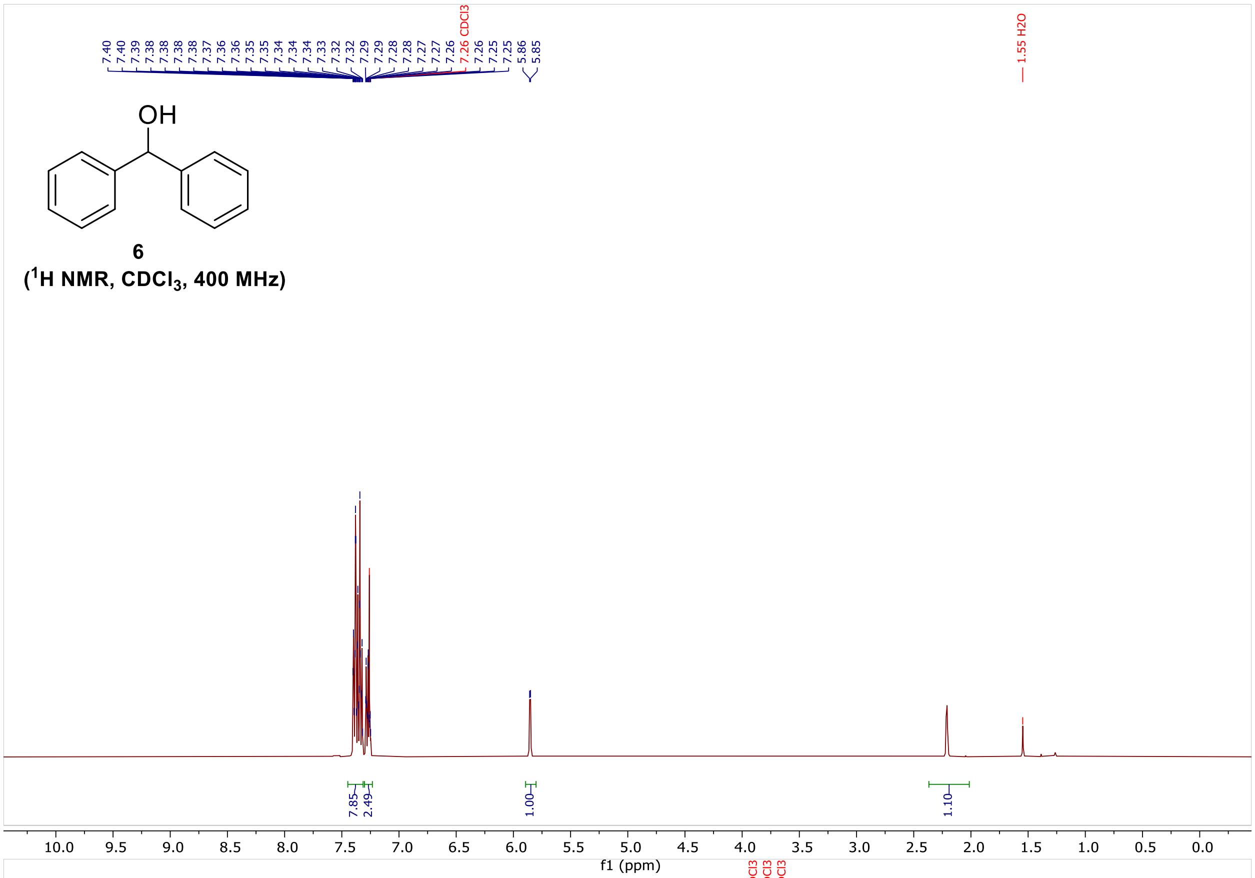
7.40 7.40 7.39 7.38 7.38 7.38 7.38 7.37 7.36 7.36 7.35 7.35 7.34 7.34 7.34 7.33 7.32 7.32 7.29 7.29 7.28 7.28 7.27 7.27 7.26 7.26 CDCl3 7.26 7.25 7.25 5.86 5.85
1.55 H2O
OH
6
(1H NMR, CDCl3, 400 MHz)
7.85 2.49 1.00 1.10
10.0 9.5 9.0 8.5 8.0 7.5 7.0 6.5 6.0 5.5 5.0 4.5 4.0 3.5 3.0 2.5 2.0 1.5 1.0 0.5 0.0
f1 (ppm)

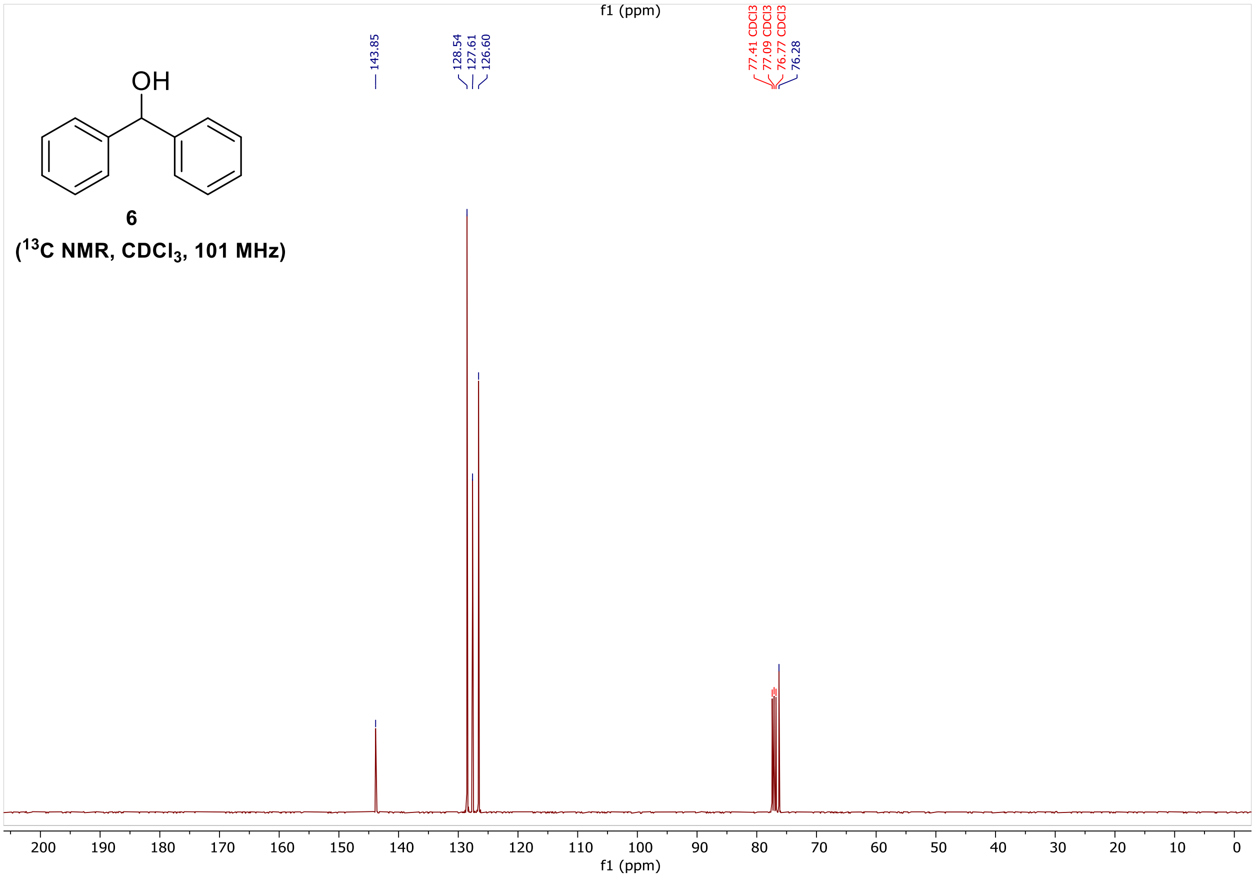
143.85
128.54 127.61 126.60
77.41 CDCl3 77.09 CDCl3 76.77 CDCl3 76.28
OH
6
(13C NMR, CDCl3, 101 MHz)
200 190 180 170 160 150 140 130 120 110 100 90 80 70 60 50 40 30 20 10 0
f1 (ppm)

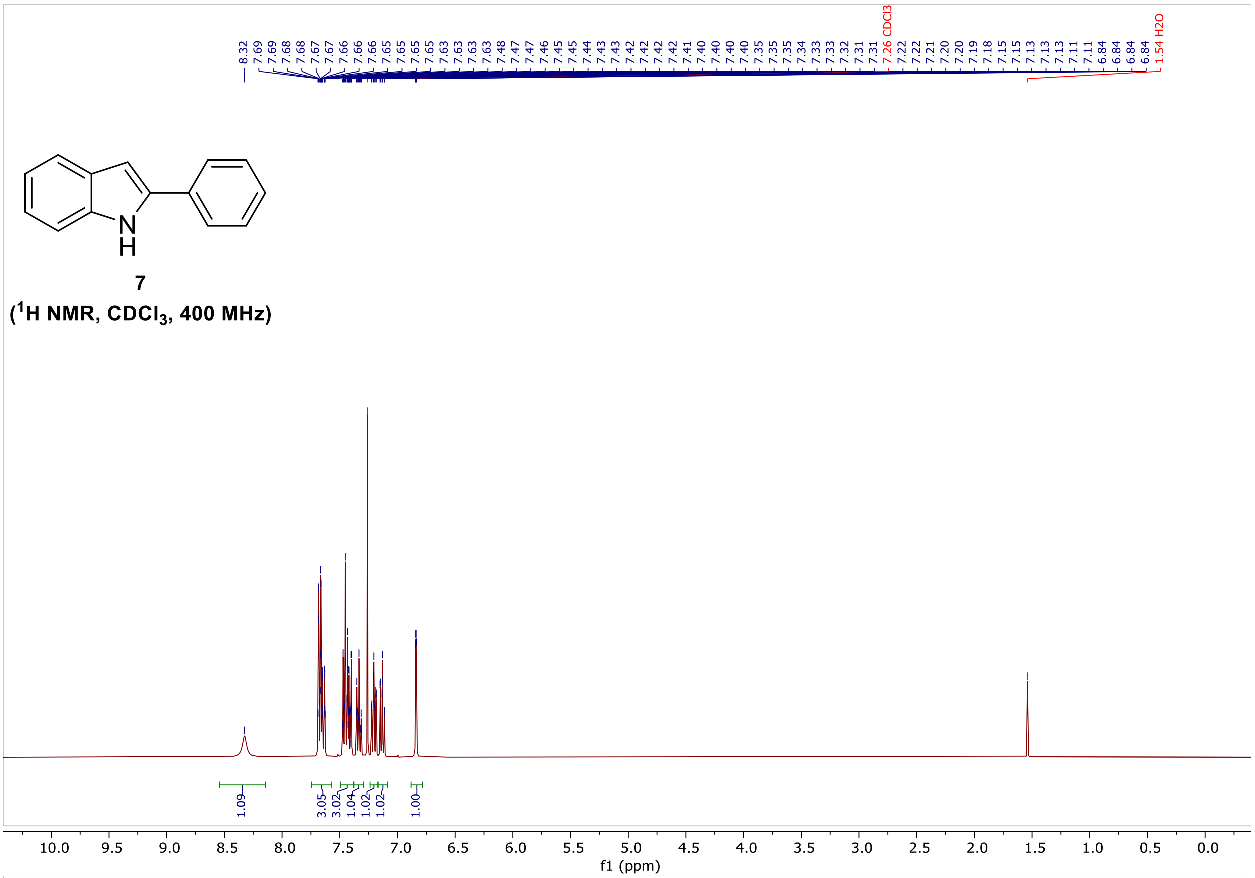
7
(^{1}H NMR, $CDCl_3$, 400 MHz)
f1 (ppm)

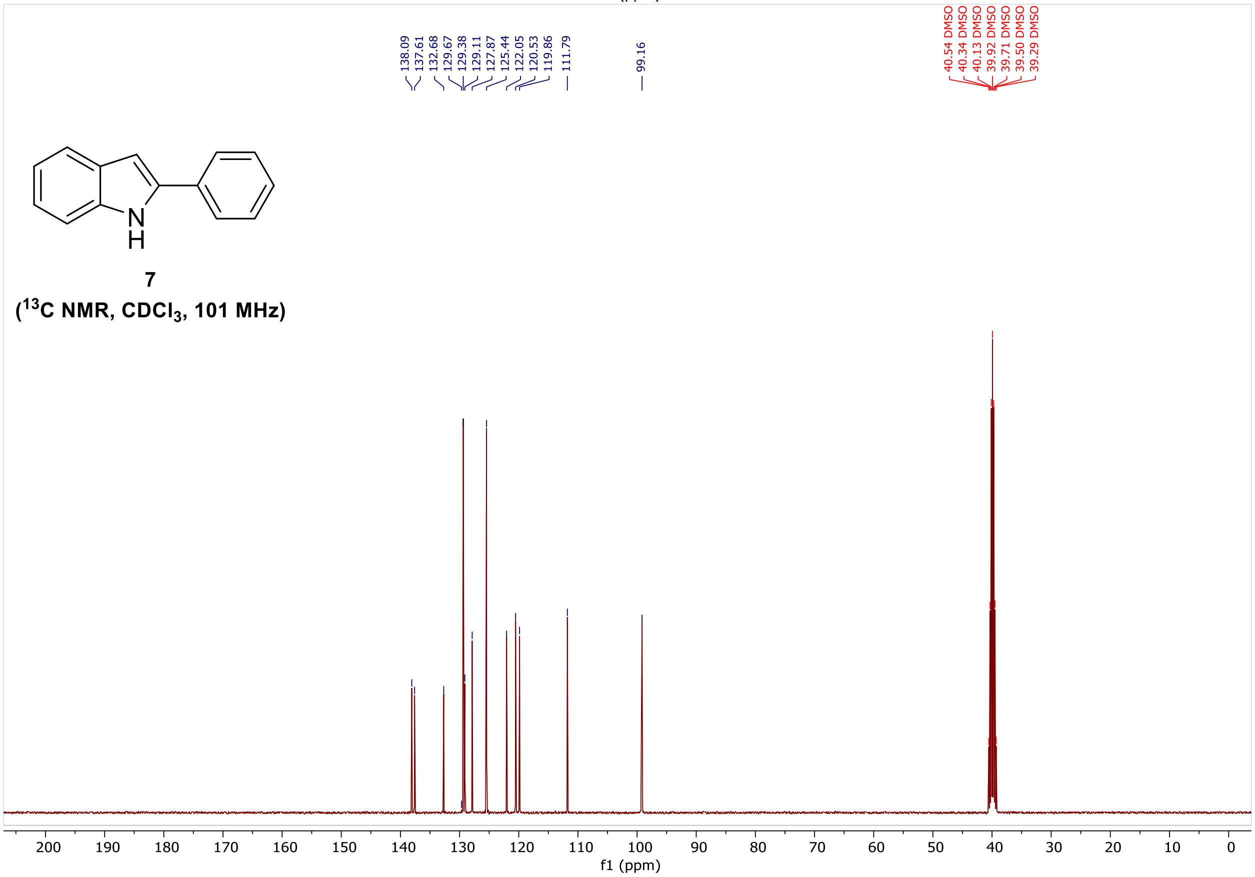
7
(^{13}C NMR, $CDCl_3$, 101 MHz)
f1 (ppm)

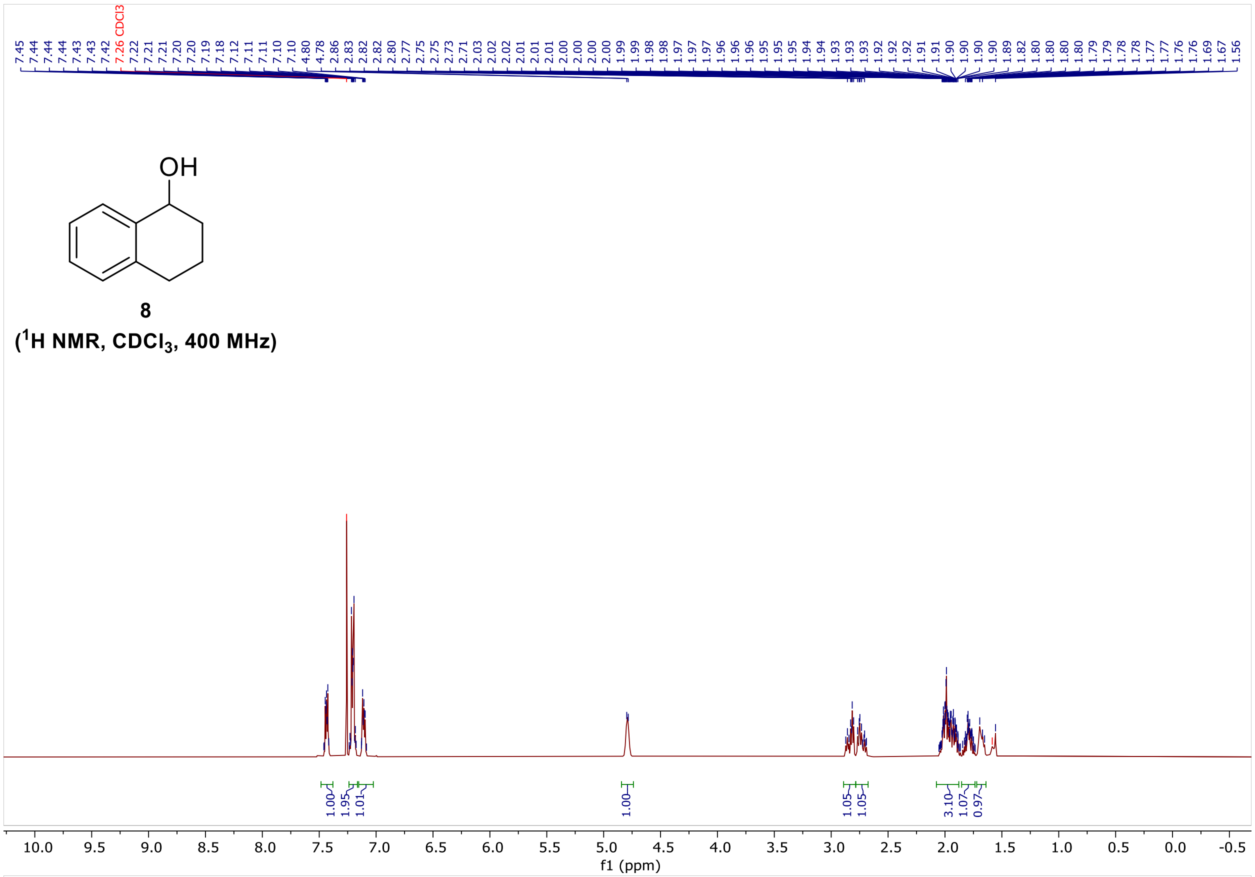
7.45 7.44 7.44 7.44 7.43 7.43 7.42 7.26 CDCl3 7.22 7.21 7.21 7.20 7.20 7.19 7.18 7.12 7.11 7.11 7.10 7.10 4.80 4.78 2.86 2.83 2.82 2.80 2.77 2.75 2.75 2.73 2.71 2.03 2.02 2.02 2.01 2.01 2.00 2.00 2.00 1.99 1.99 1.98 1.98 1.97 1.97 1.96 1.96 1.96 1.95 1.95 1.95 1.94 1.94 1.93 1.93 1.93 1.92 1.92 1.91 1.91 1.90 1.90 1.90 1.90 1.89 1.82 1.80 1.80 1.80 1.79 1.78 1.78 1.77 1.77 1.76 1.76 1.69 1.67 1.56
OH
8
(1H NMR, CDCl3, 400 MHz)
1.00 1.95 1.01 1.00 1.05 1.05 3.10 1.07 0.97
10.0 9.5 9.0 8.5 8.0 7.5 7.0 6.5 6.0 5.5 5.0 4.5 4.0 3.5 3.0 2.5 2.0 1.5 1.0 0.5 0.0 -0.5
f1 (ppm)

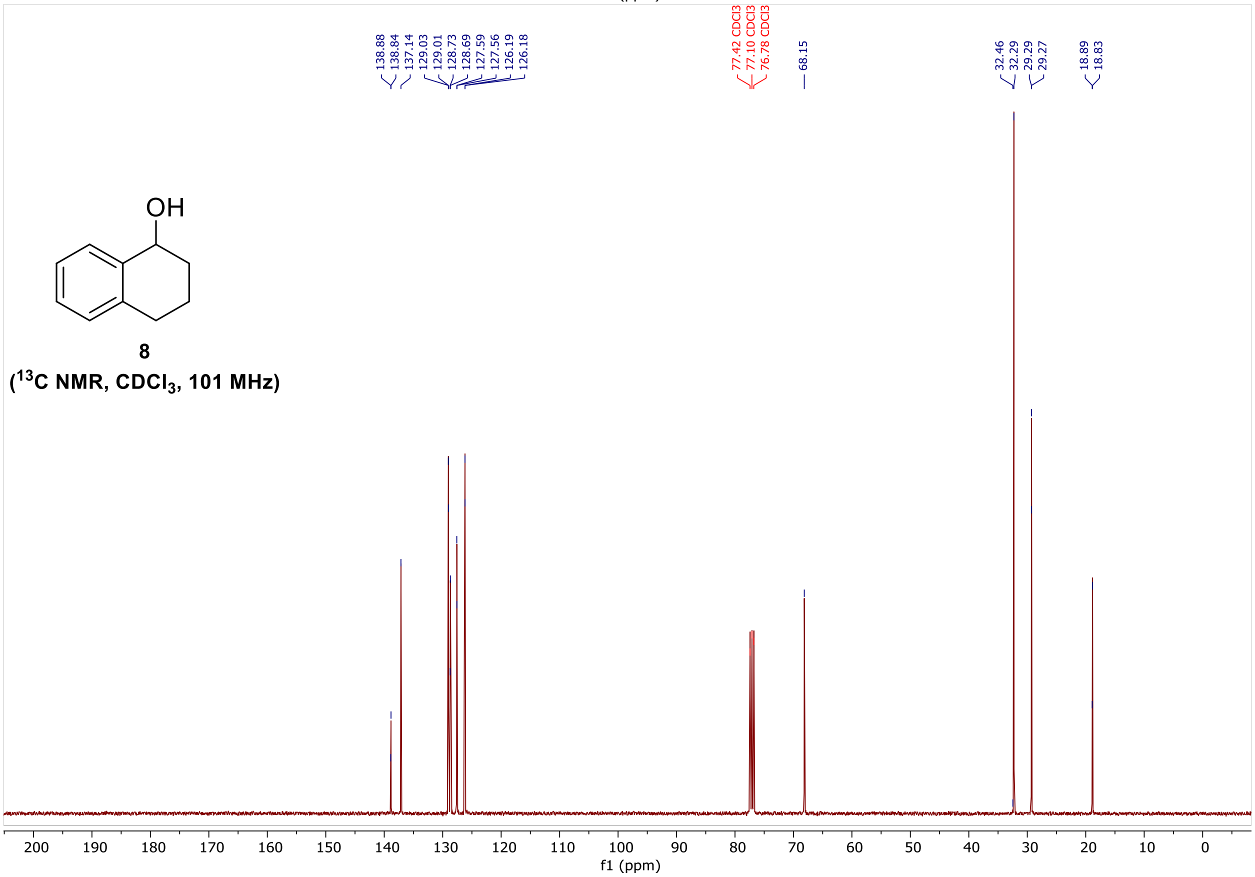
138.88 138.84 137.14 129.03 129.01 128.73 128.69 127.59 127.56 126.19 126.18
77.42 CDCl3 77.10 CDCl3 76.78 CDCl3
68.15
32.46 32.29 29.29 29.27
18.89 18.83
OH
8
(13C NMR, CDCl3, 101 MHz)
200 190 180 170 160 150 140 130 120 110 100 90 80 70 60 50 40 30 20 10 0
f1 (ppm)

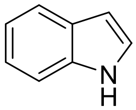

9

(^{1}H NMR, $CDCl_3$, 400 MHz)

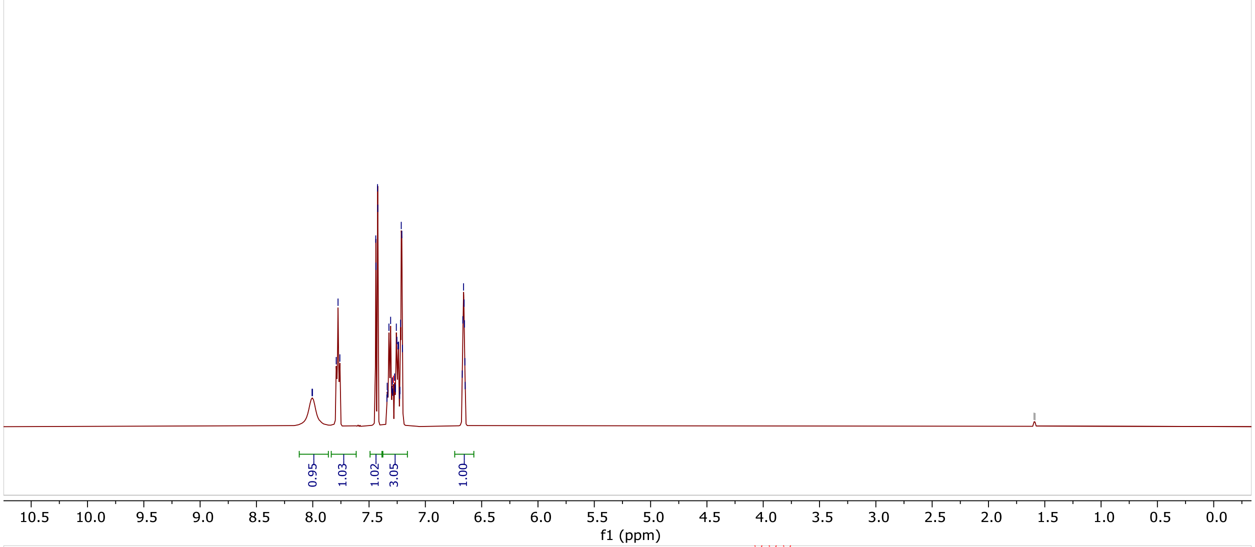

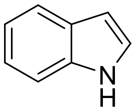

9

(^{13}C NMR, $CDCl_3$, 126 MHz)

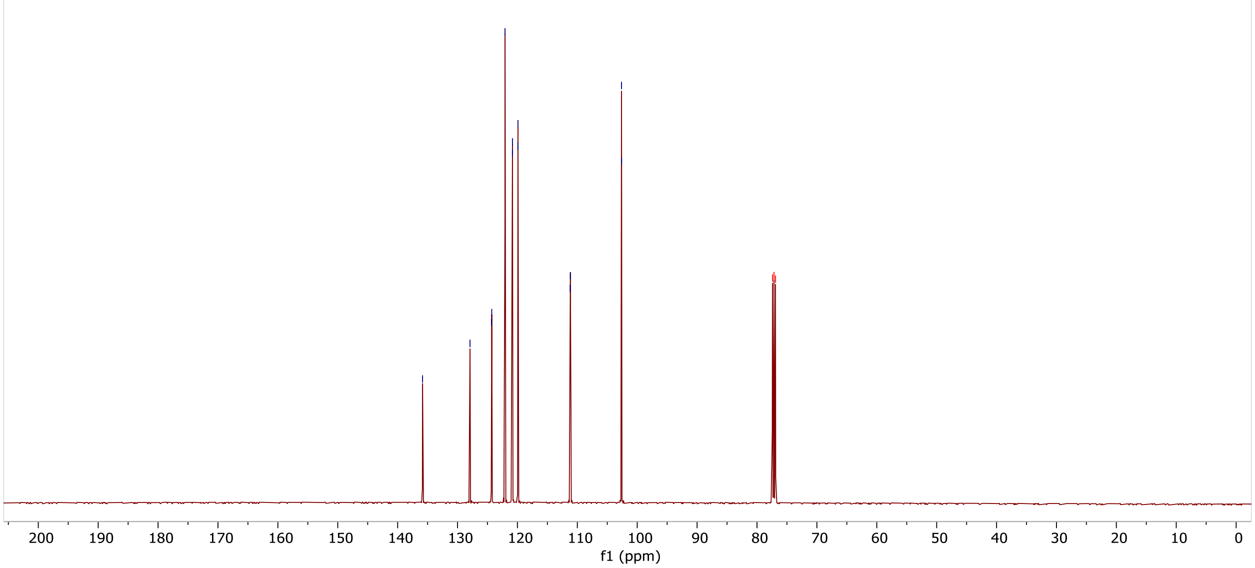

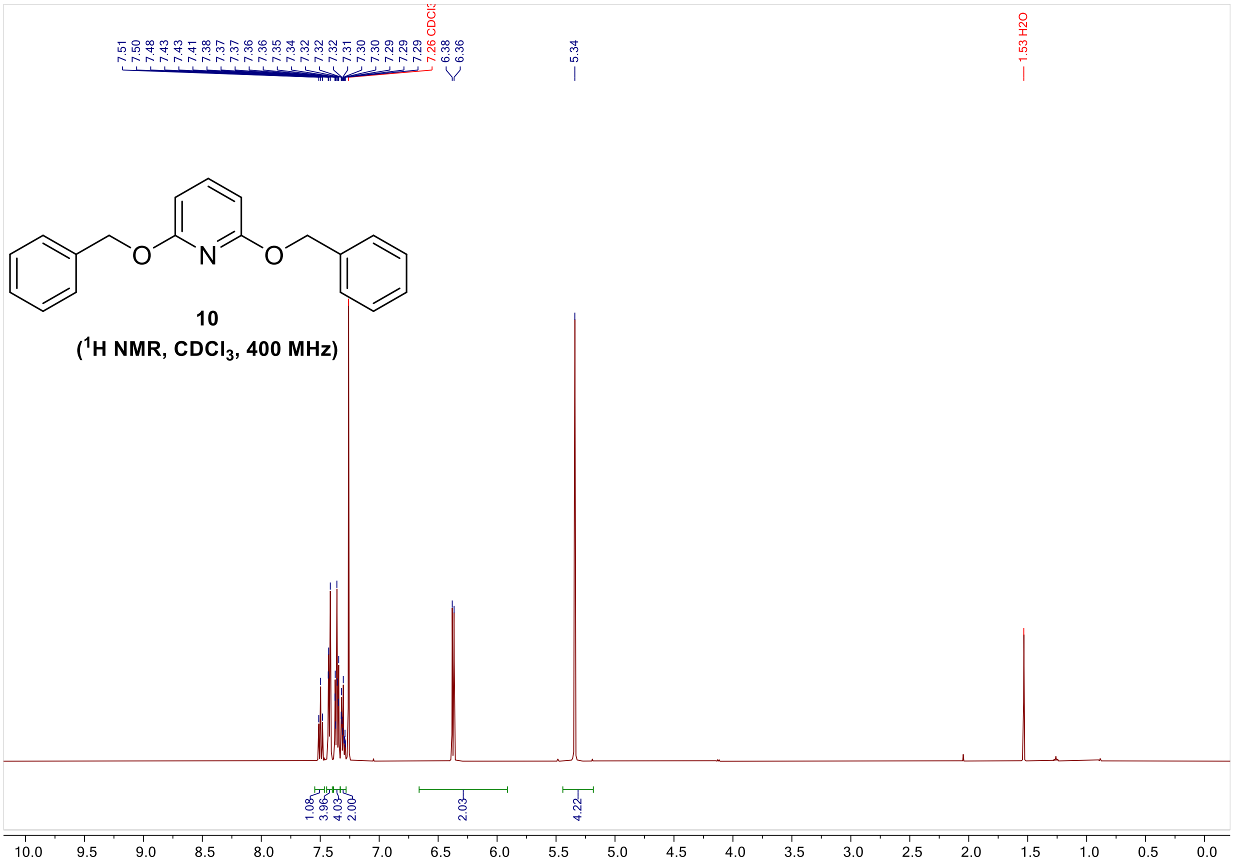

7.51
7.50
7.48
7.43
7.43
7.41
7.38
7.37
7.37
7.36
7.36
7.35
7.34
7.32
7.32
7.32
7.31
7.30
7.30
7.29
7.29
7.29
7.26 CDCl3
6.38
6.36
5.34
1.53 H2O
O
N
O
10
(1H NMR, CDCl3, 400 MHz)
1.08
3.96
4.03
2.00
2.03
4.22
10.0
9.5
9.0
8.5
8.0
7.5
7.0
6.5
6.0
5.5
5.0
4.5
4.0
3.5
3.0
2.5
2.0
1.5
1.0
0.5
0.0

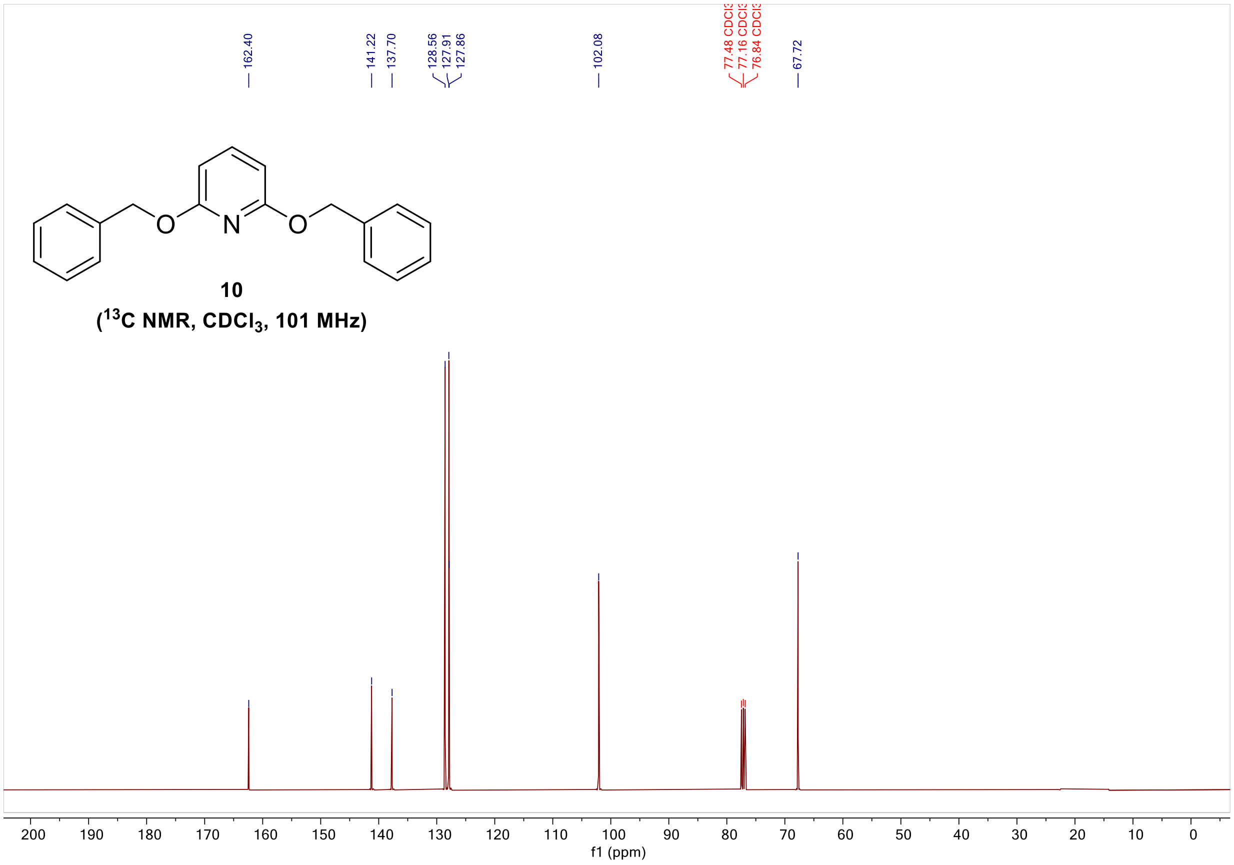

162.40
141.22
137.70
128.56
127.91
127.86
102.08
77.48 CDCl3
77.16 CDCl3
76.84 CDCl3
67.72
O
N
O
10
(13C NMR, CDCl3, 101 MHz)
200
190
180
170
160
150
140
130
120
110
100
90
80
70
60
50
40
30
20
10
0
f1 (ppm)

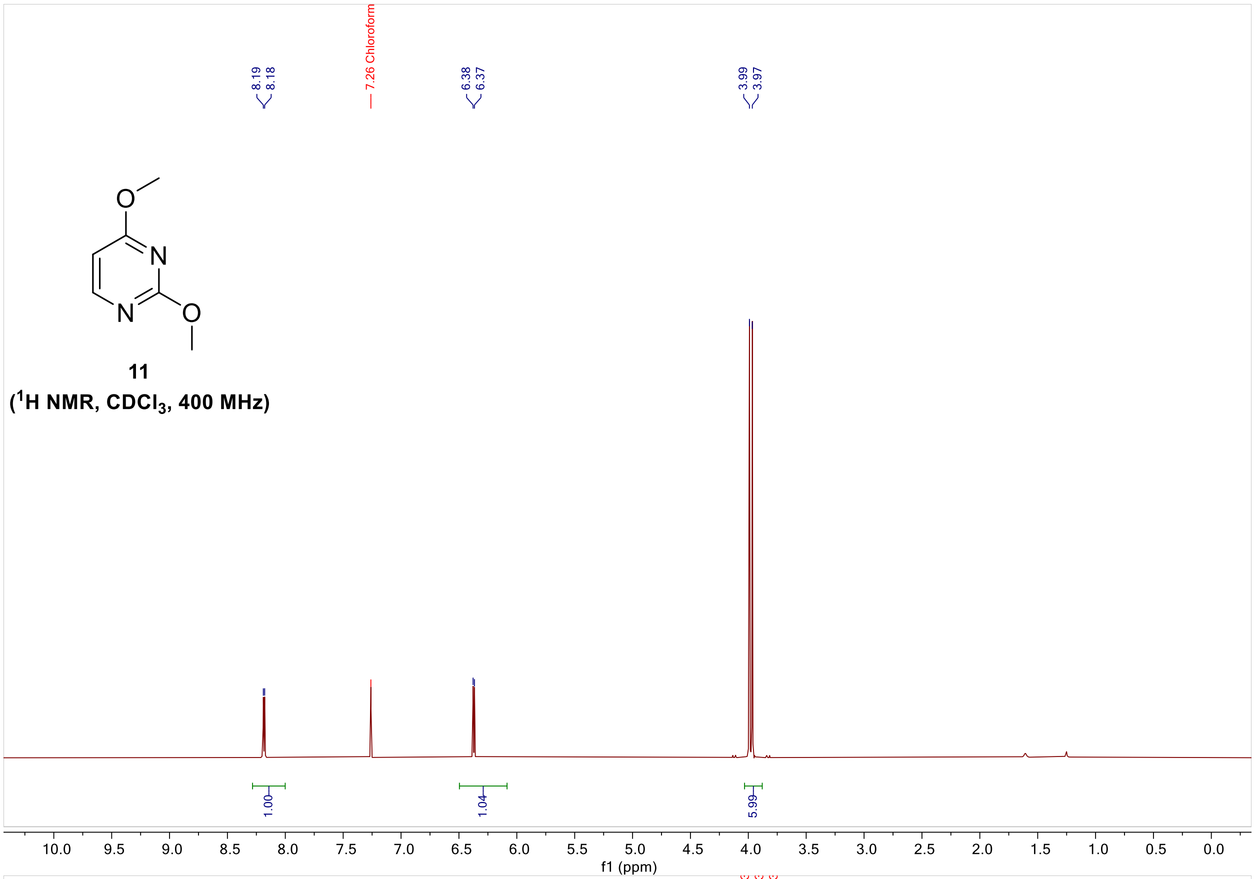
8.19
8.18
7.26 Chloroform
6.38
6.37
3.99
3.97
11
(¹H NMR, CDCl₃, 400 MHz)
1.00
1.04
5.99
10.0 9.5 9.0 8.5 8.0 7.5 7.0 6.5 6.0 5.5 5.0 4.5 4.0 3.5 3.0 2.5 2.0 1.5 1.0 0.5 0.0
f1 (ppm)

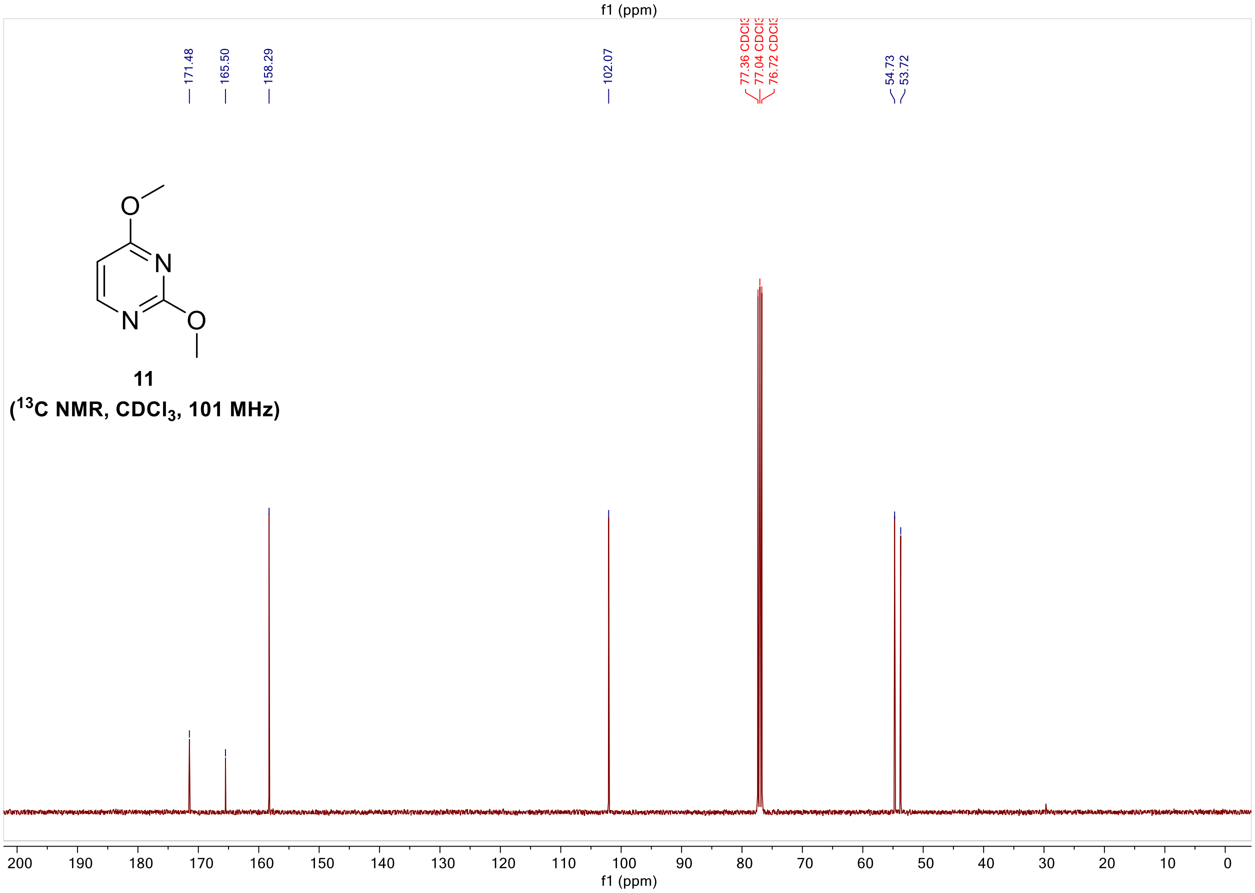
171.48
165.50
158.29
102.07
77.36 CDCl3
77.04 CDCl3
76.72 CDCl3
54.73
53.72
11
(¹³C NMR, CDCl₃, 101 MHz)
200 190 180 170 160 150 140 130 120 110 100 90 80 70 60 50 40 30 20 10 0
f1 (ppm)

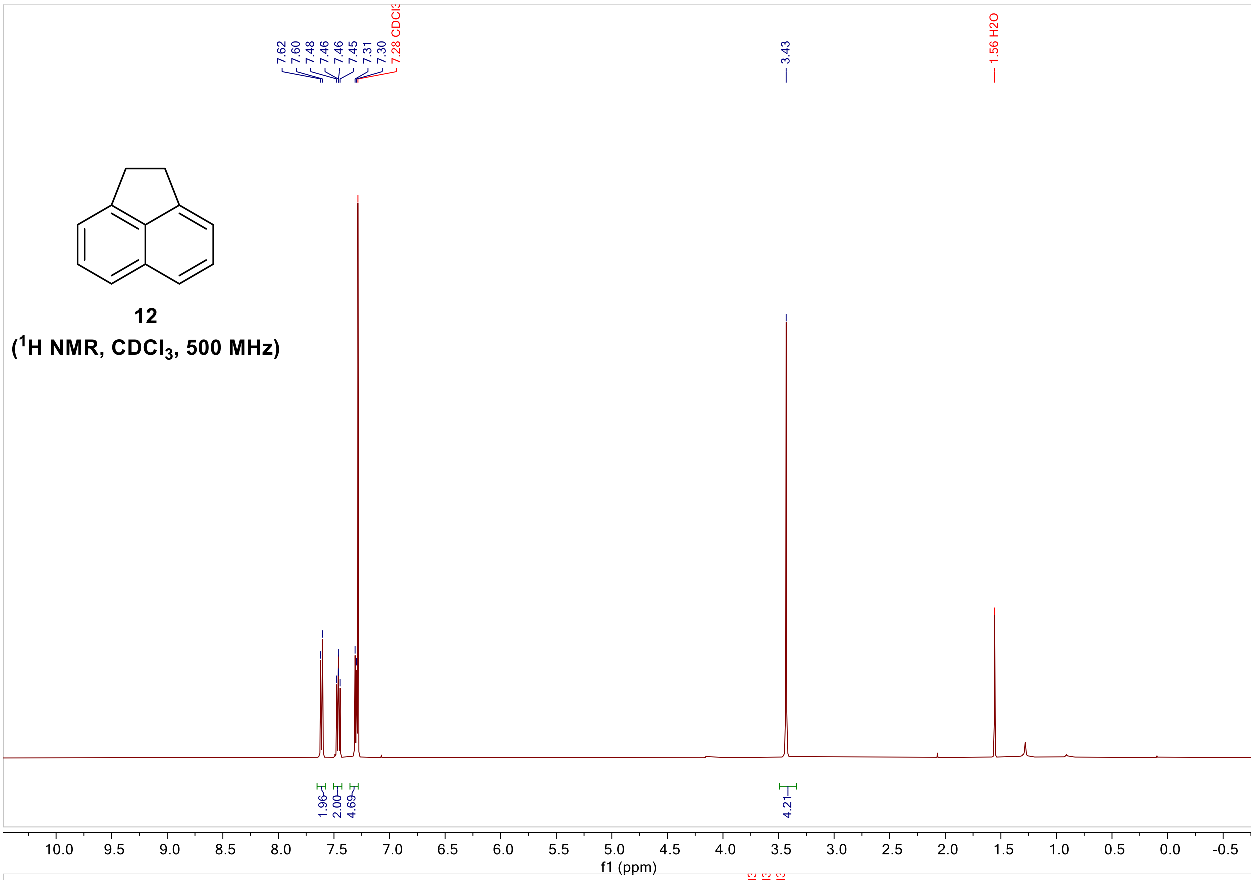

12
(^{1}H NMR, $CDCl_3$, 500 MHz)

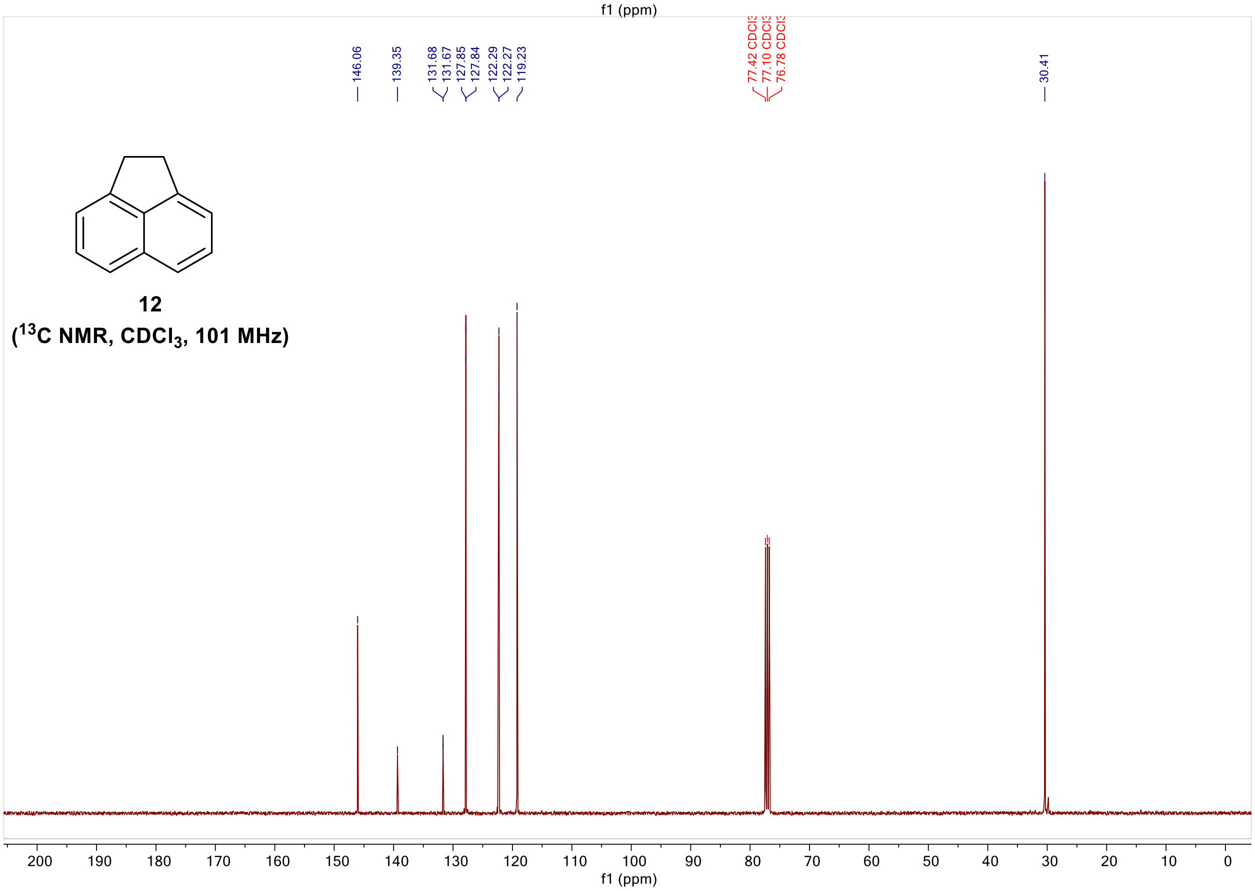

12
(^{13}C NMR, $CDCl_3$, 101 MHz)

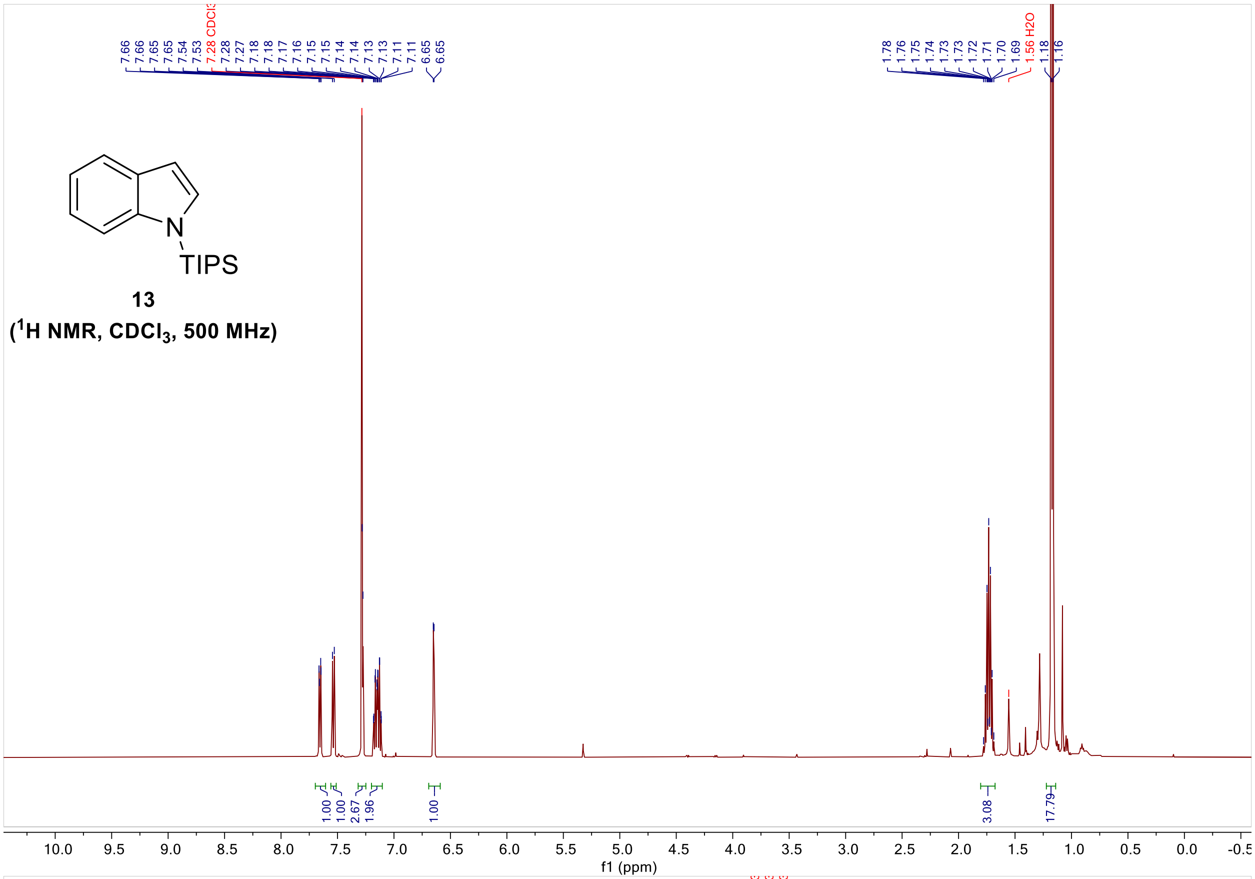

13
(^{1}H NMR, CDCl$_3$, 500 MHz)

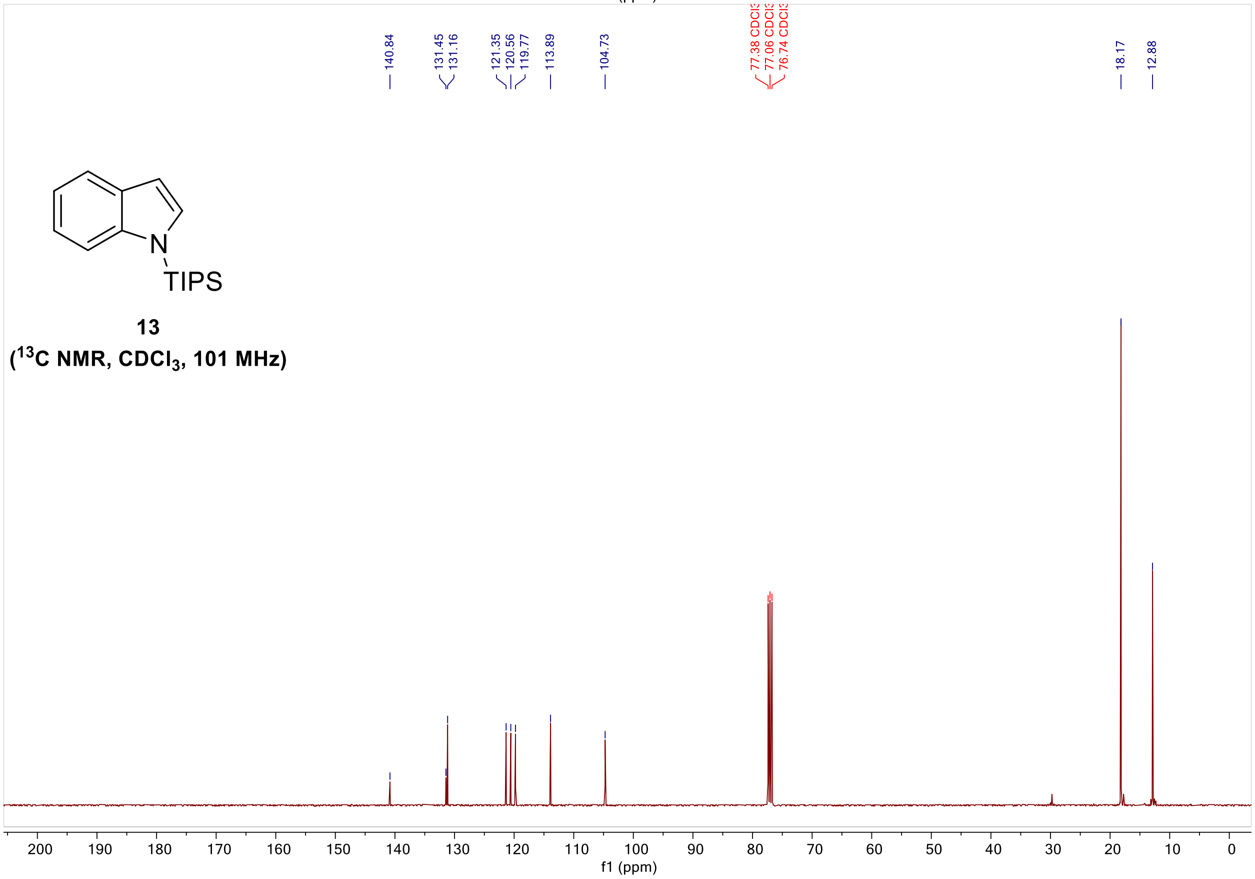

13
(^{13}C NMR, CDCl$_3$, 101 MHz)

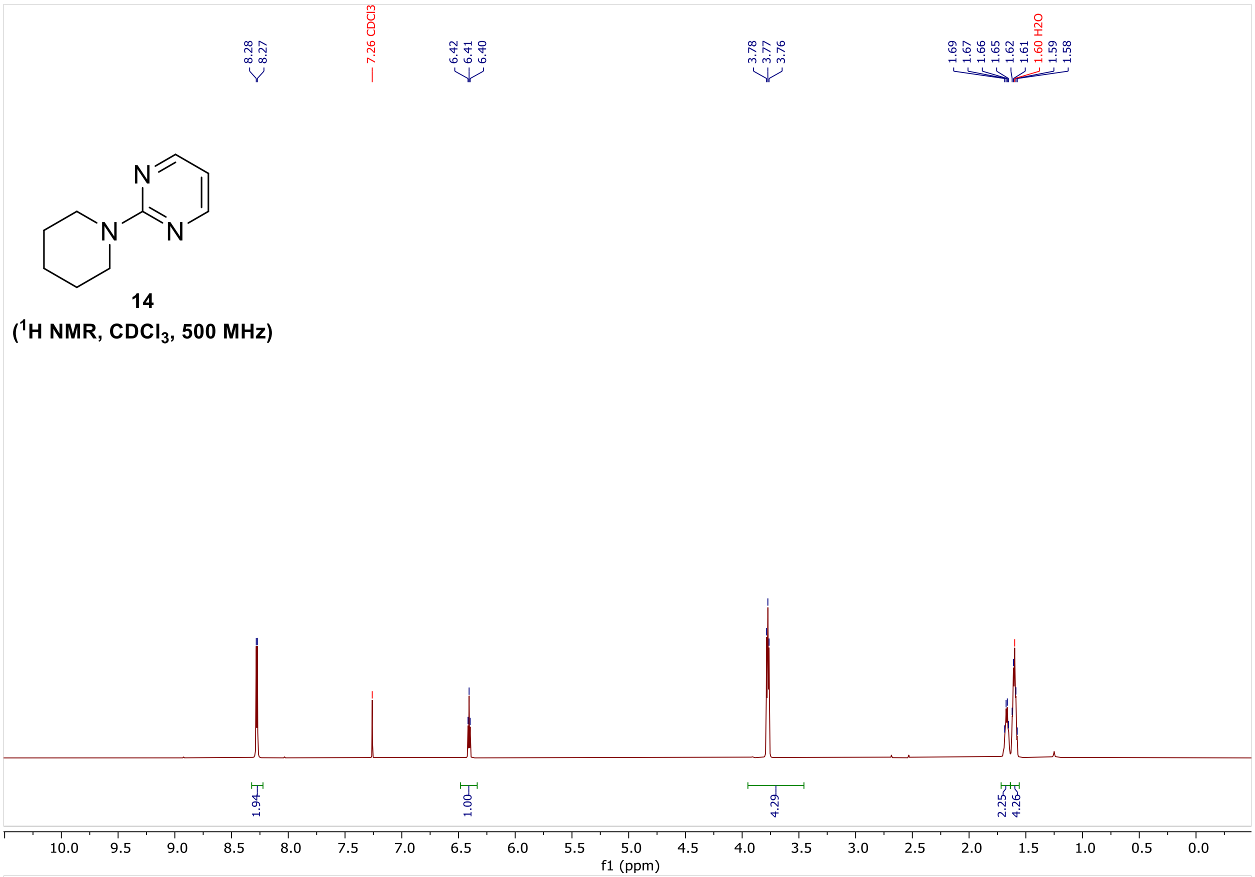

14
(^{1}H NMR, $CDCl_3$, 500 MHz)

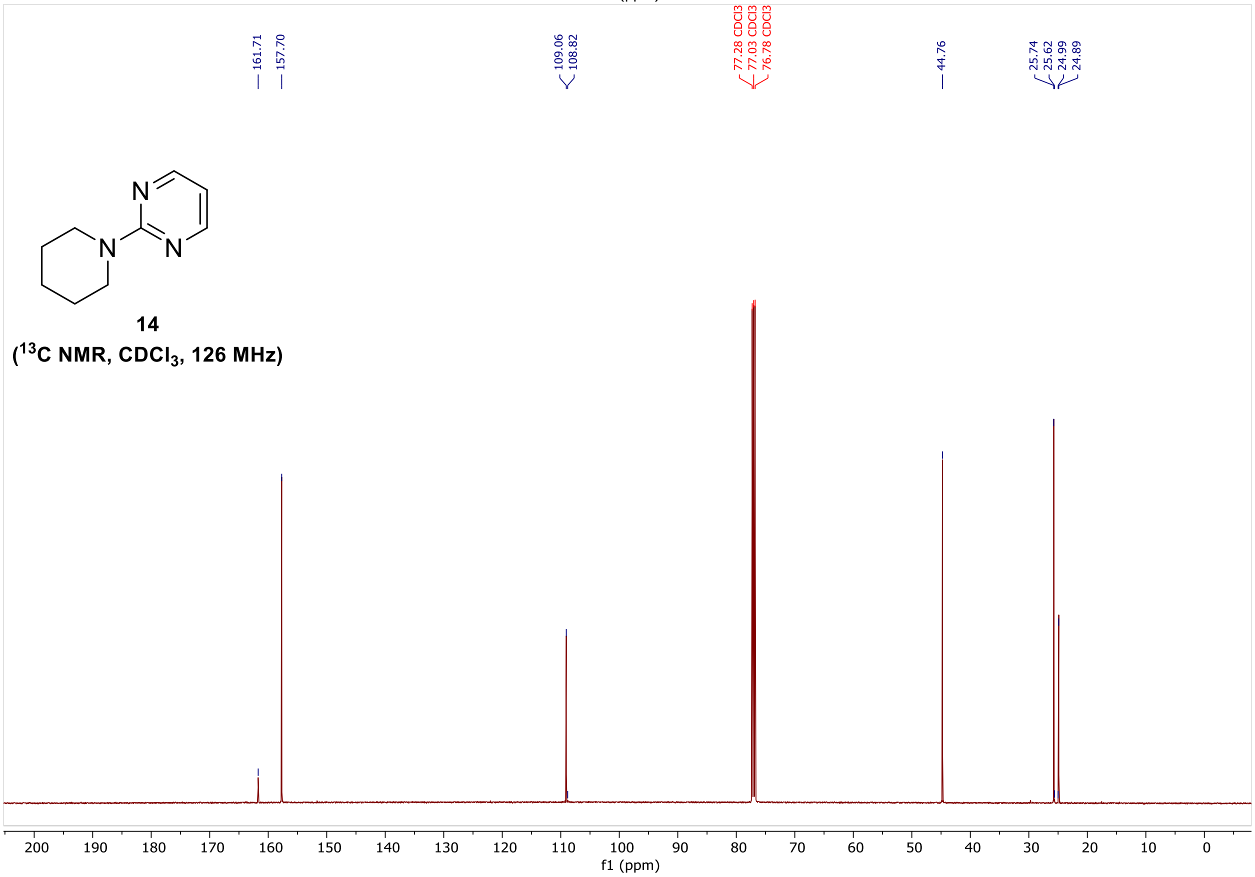

14
(^{13}C NMR, $CDCl_3$, 126 MHz)

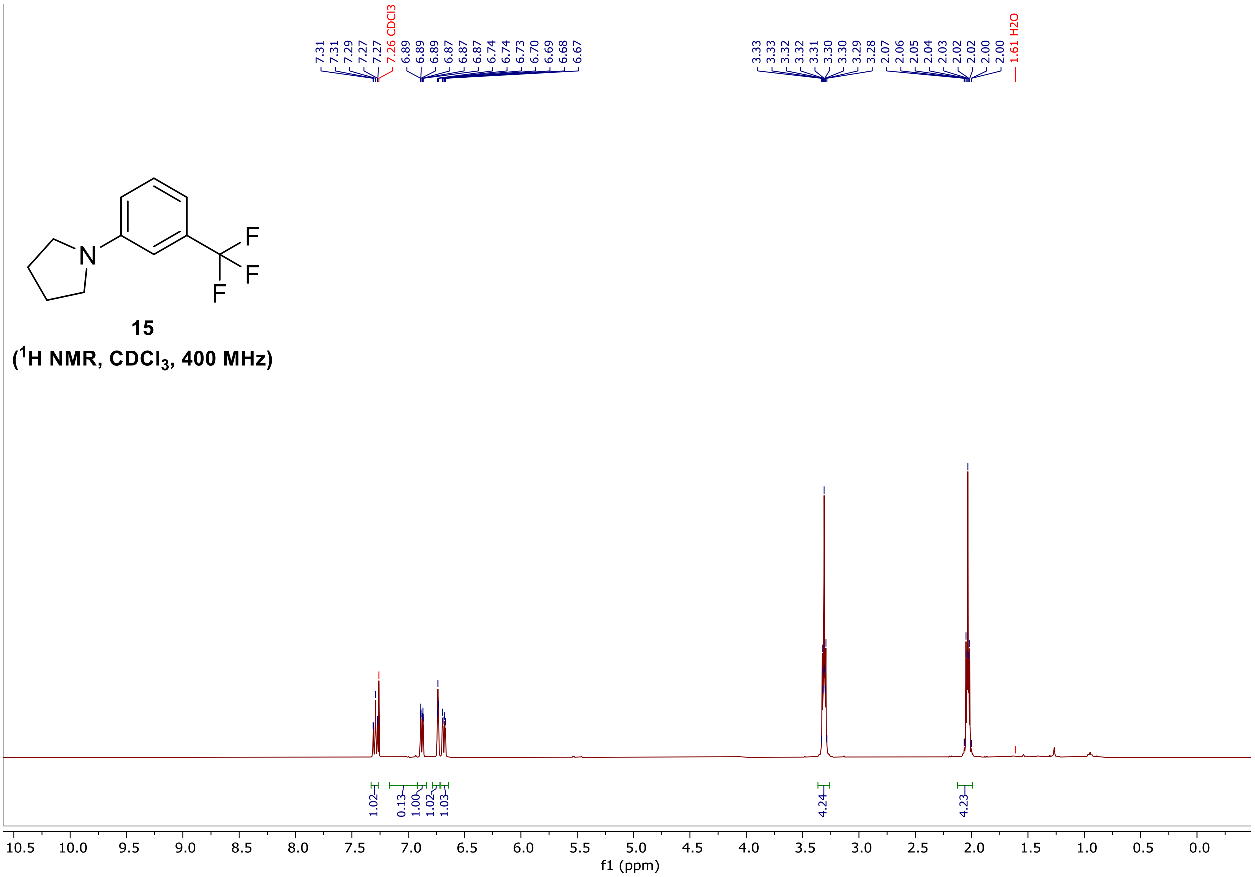
7.31
7.31
7.29
7.27
7.27
7.26 CDCl3
6.89
6.89
6.89
6.87
6.87
6.87
6.74
6.74
6.73
6.70
6.69
6.68
6.67
3.33
3.33
3.32
3.32
3.31
3.30
3.30
3.29
3.28
2.07
2.06
2.05
2.04
2.03
2.02
2.02
2.00
2.00
1.61 H2O
N
F
F
F
15
(1H NMR, CDCl3, 400 MHz)
1.02
0.13
1.00
1.02
1.03
4.24
4.23
f1 (ppm)

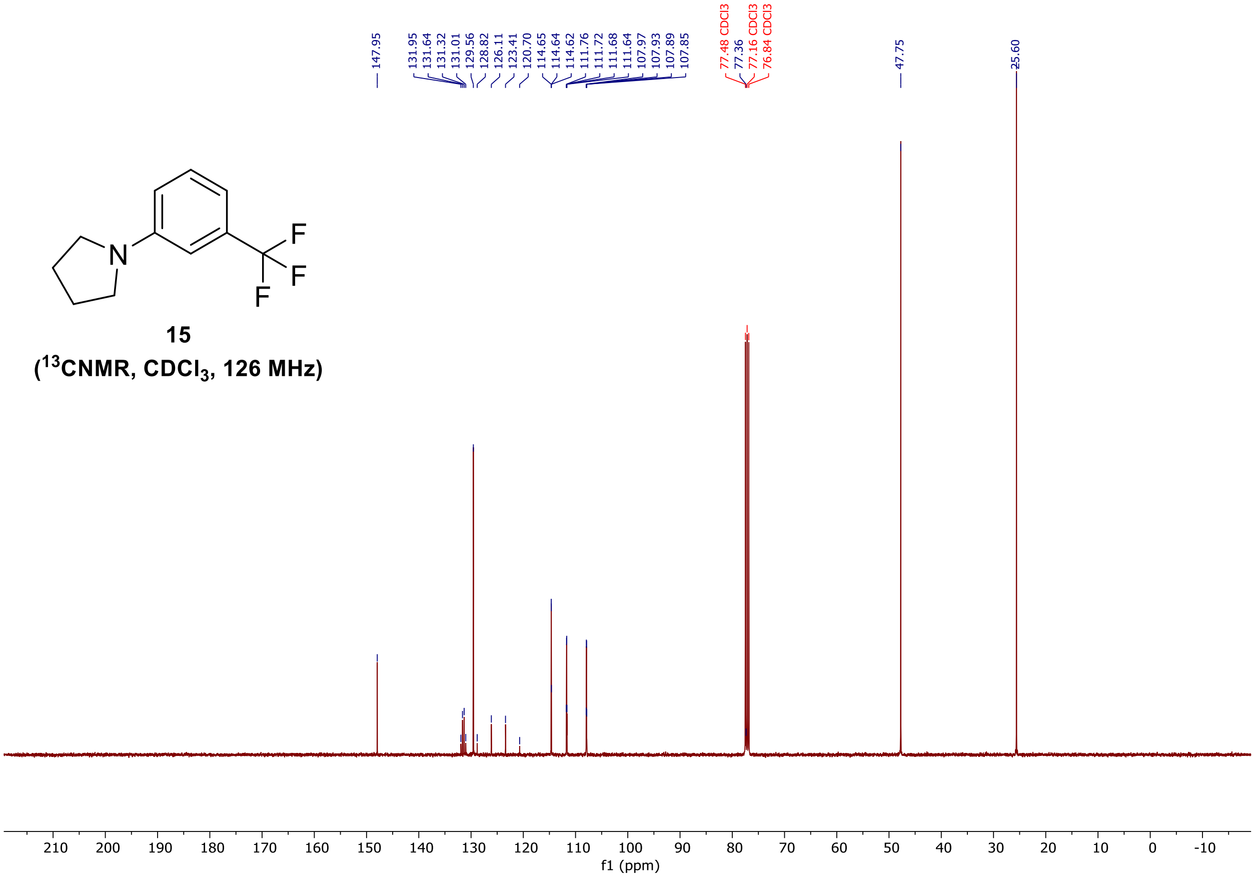
147.95
131.95
131.64
131.32
131.01
129.56
128.82
126.11
123.41
120.70
114.65
114.64
114.62
111.76
111.72
111.68
111.64
107.97
107.93
107.89
107.85
77.48 CDCl3
77.36
77.16 CDCl3
76.84 CDCl3
47.75
25.60
N
F
F
F
15
(13CNMR, CDCl3, 126 MHz)
f1 (ppm)

16
(^{1}HNMR, $CDCl_3$, 400 MHz)

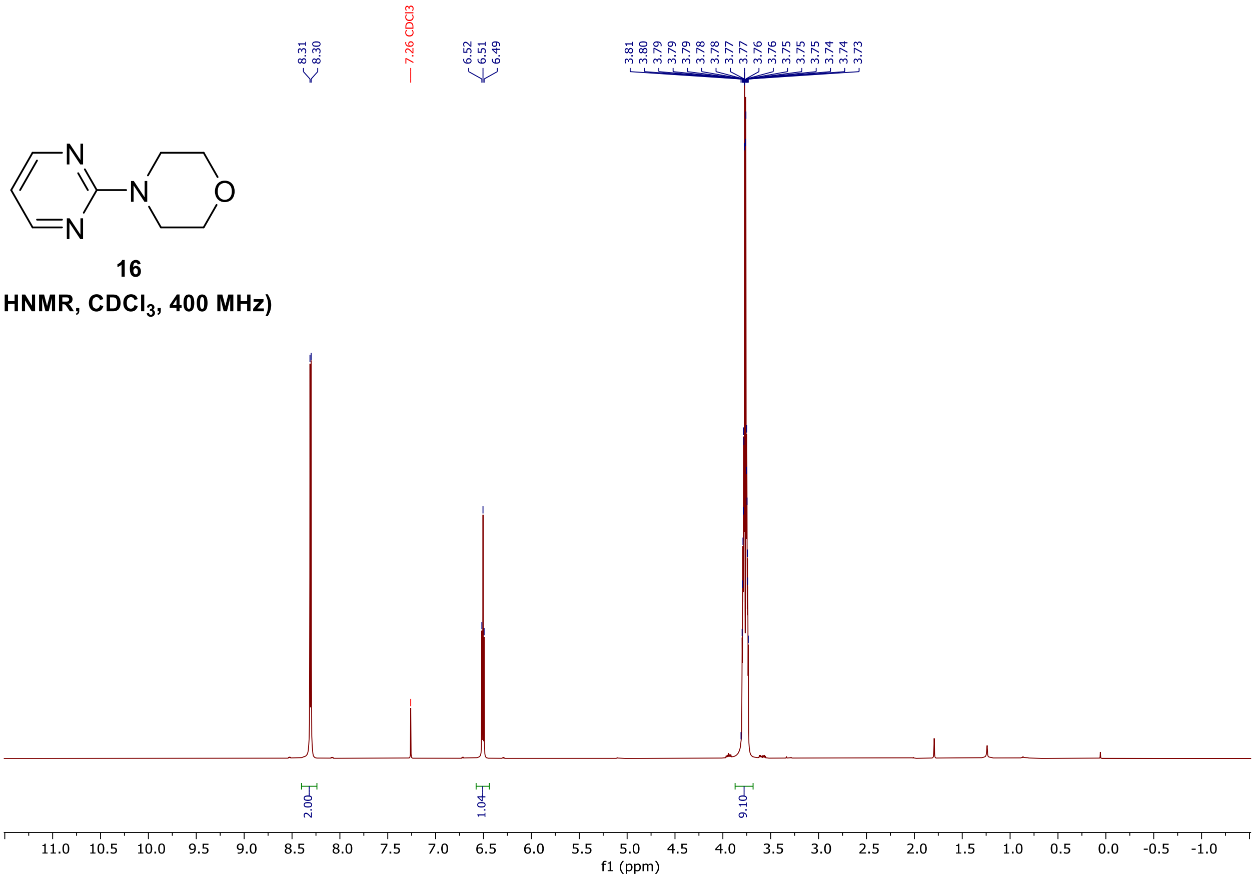

16
(^{13}CMR, $CDCl_3$, 126 MHz)

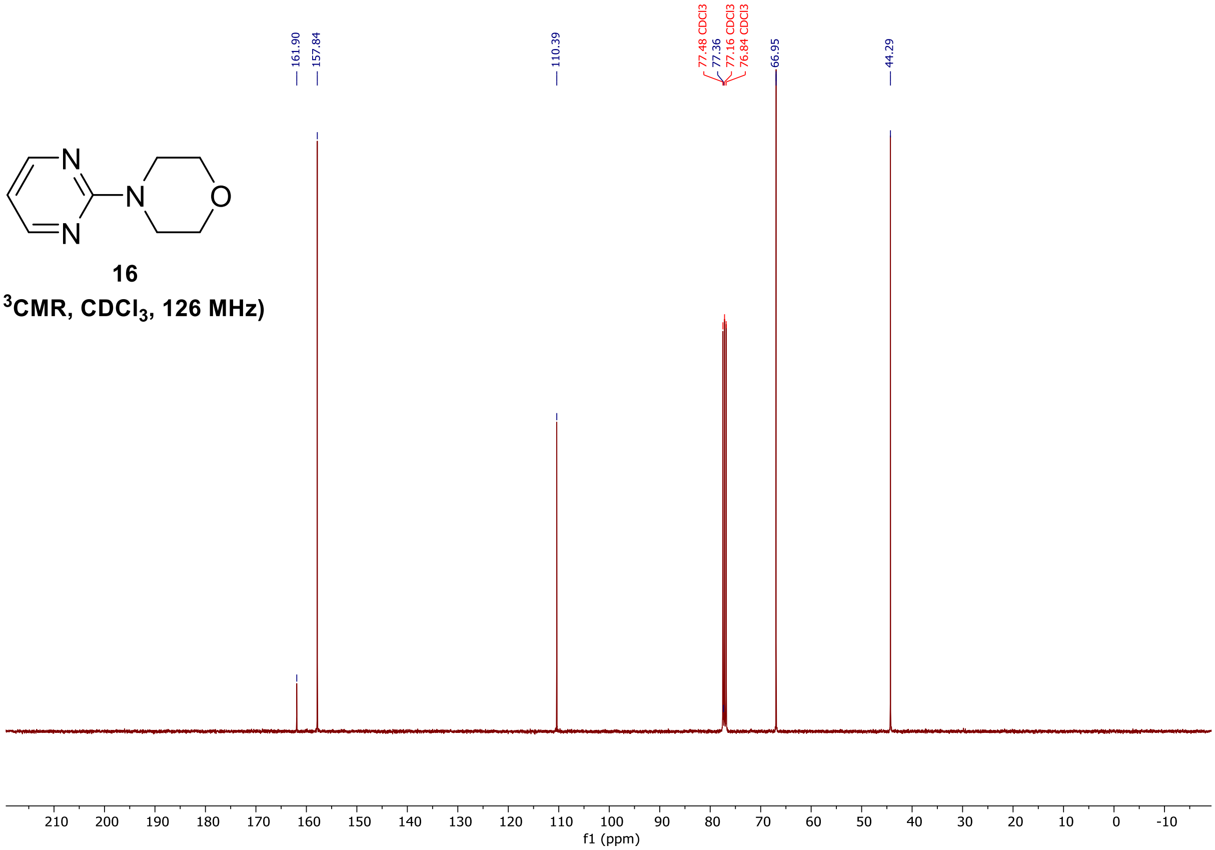

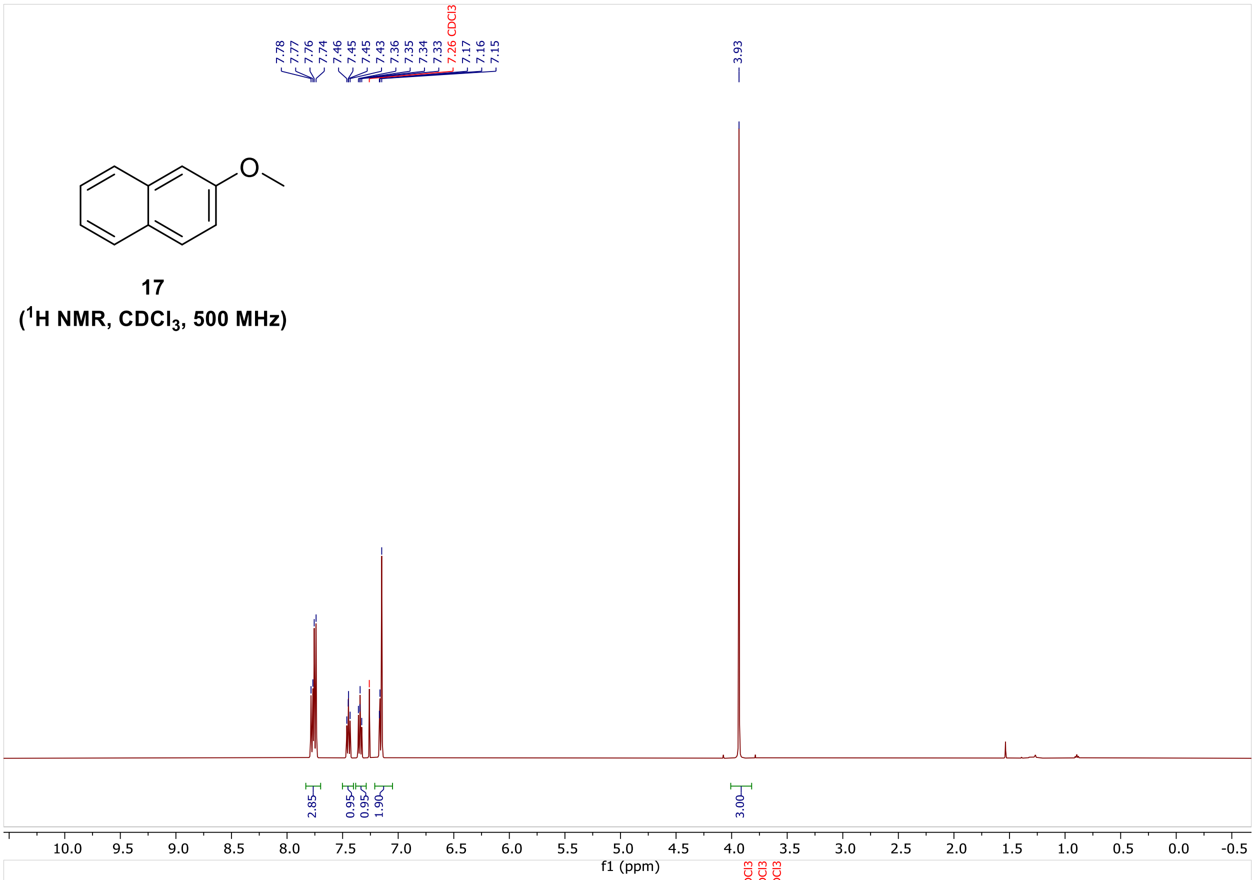
7.78
7.77
7.76
7.74
7.46
7.45
7.45
7.43
7.36
7.35
7.34
7.33
7.26 CDCl3
7.17
7.16
7.15
3.93
O
17
(1H NMR, CDCl3, 500 MHz)
2.85
0.95
0.95
1.90
3.00
10.0
9.5
9.0
8.5
8.0
7.5
7.0
6.5
6.0
5.5
5.0
4.5
4.0
3.5
3.0
2.5
2.0
1.5
1.0
0.5
0.0
-0.5
f1 (ppm)

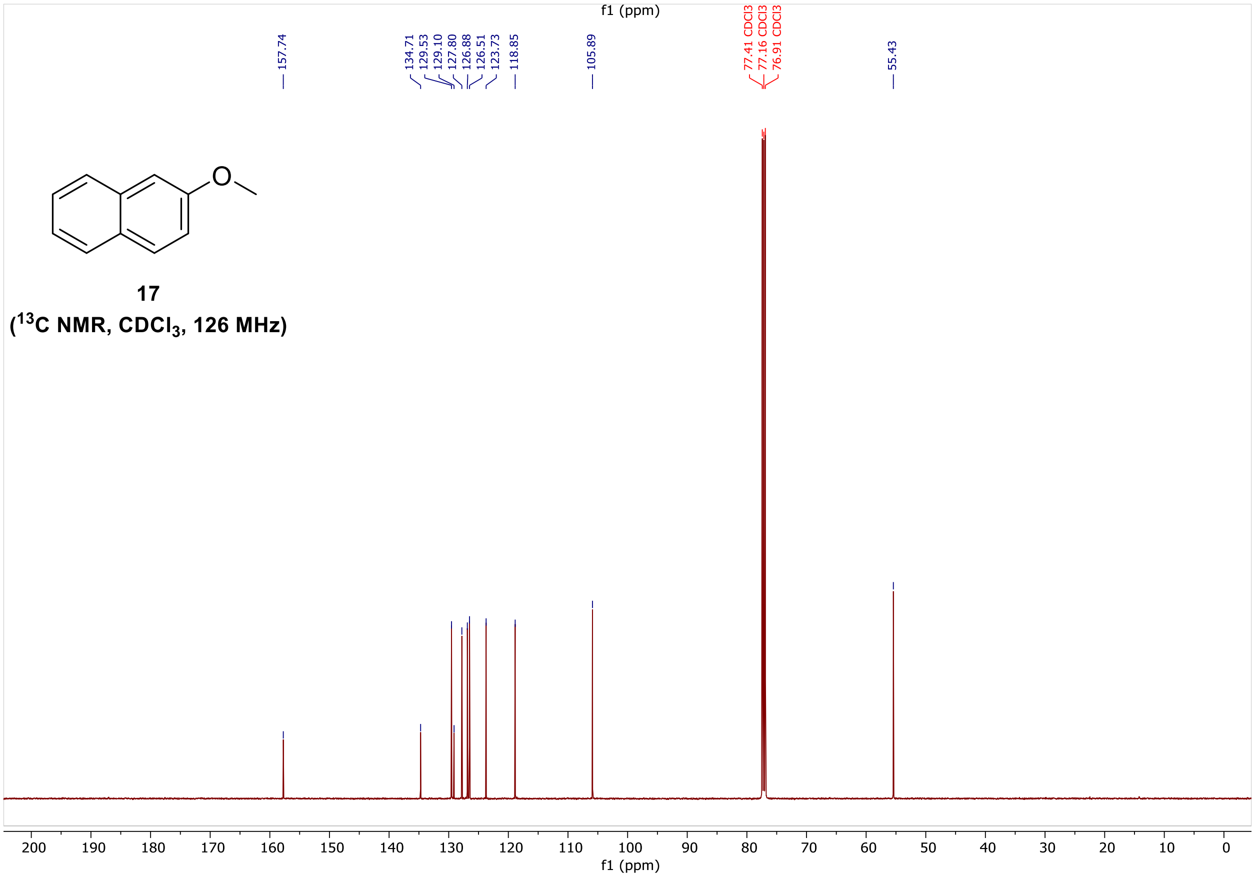
157.74
134.71
129.53
129.10
127.80
126.88
126.51
123.73
118.85
105.89
77.41 CDCl3
77.16 CDCl3
76.91 CDCl3
55.43
O
17
(13C NMR, CDCl3, 126 MHz)
200
190
180
170
160
150
140
130
120
110
100
90
80
70
60
50
40
30
20
10
0
f1 (ppm)

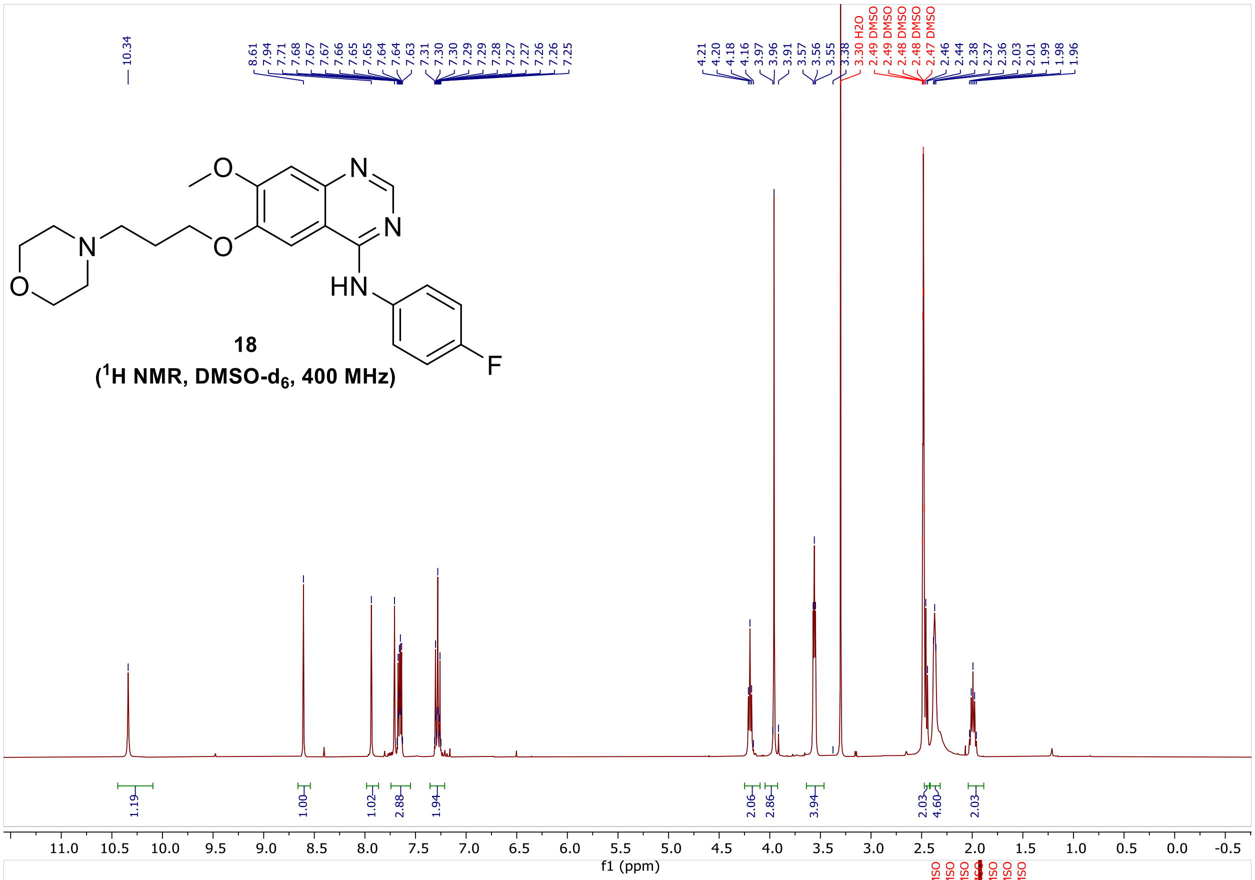

18

(^{1}H NMR, DMSO-d_6, 400 MHz)

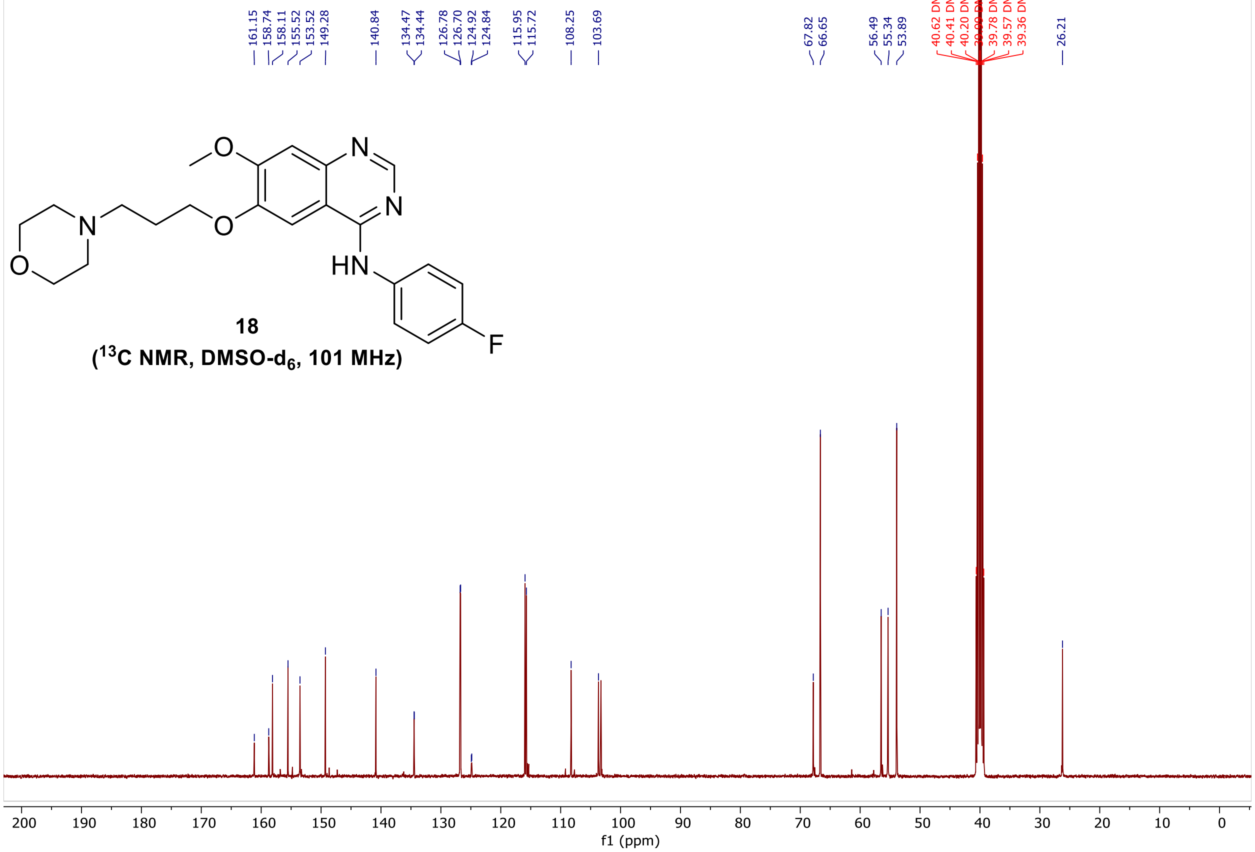

18

(^{13}C NMR, DMSO-d_6, 101 MHz)

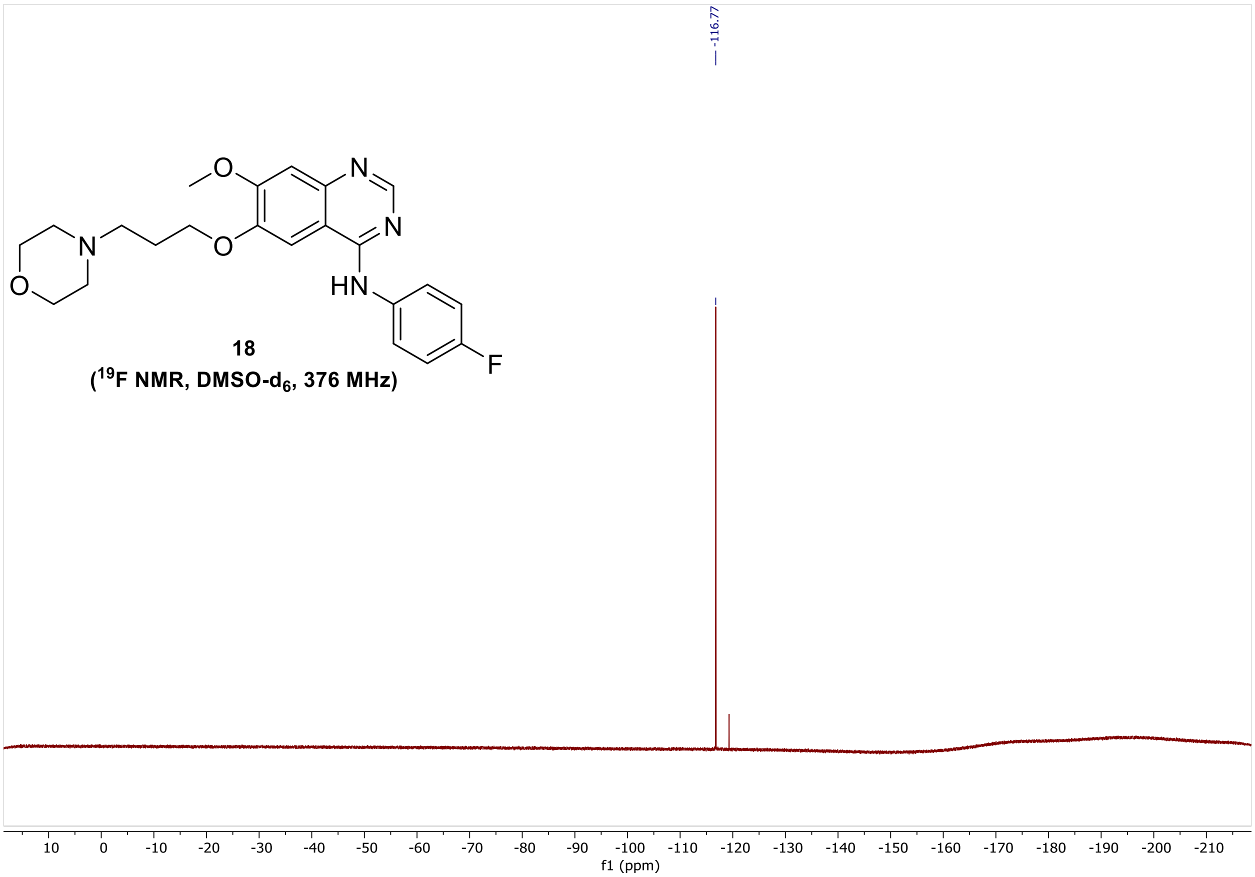

-116.77
18
(19F NMR, DMSO-d6, 376 MHz)
10
0
-10
-20
-30
-40
-50
-60
-70
-80
-90
-100
-110
-120
-130
-140
-150
-160
-170
-180
-190
-200
-210
f1 (ppm)

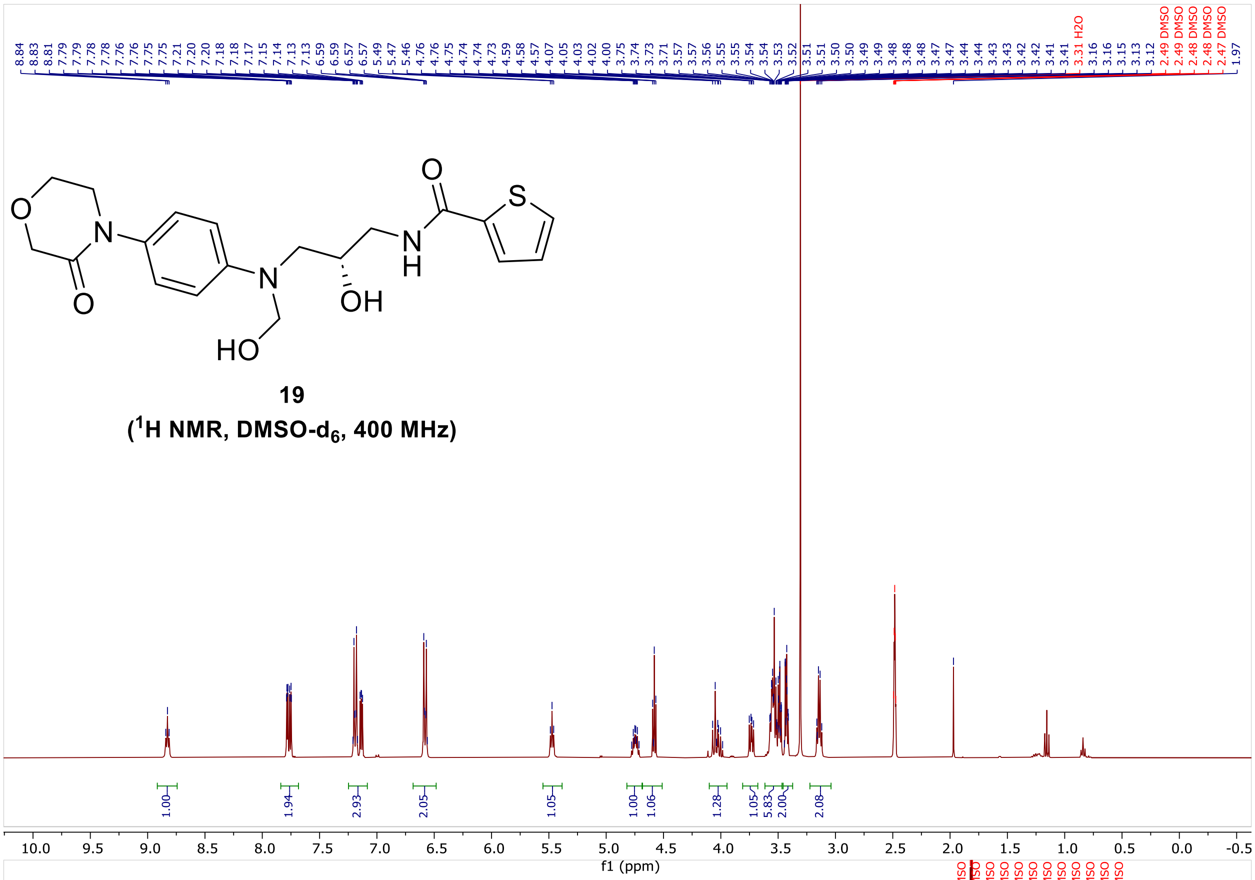
19
(¹H NMR, DMSO-d6, 400 MHz)

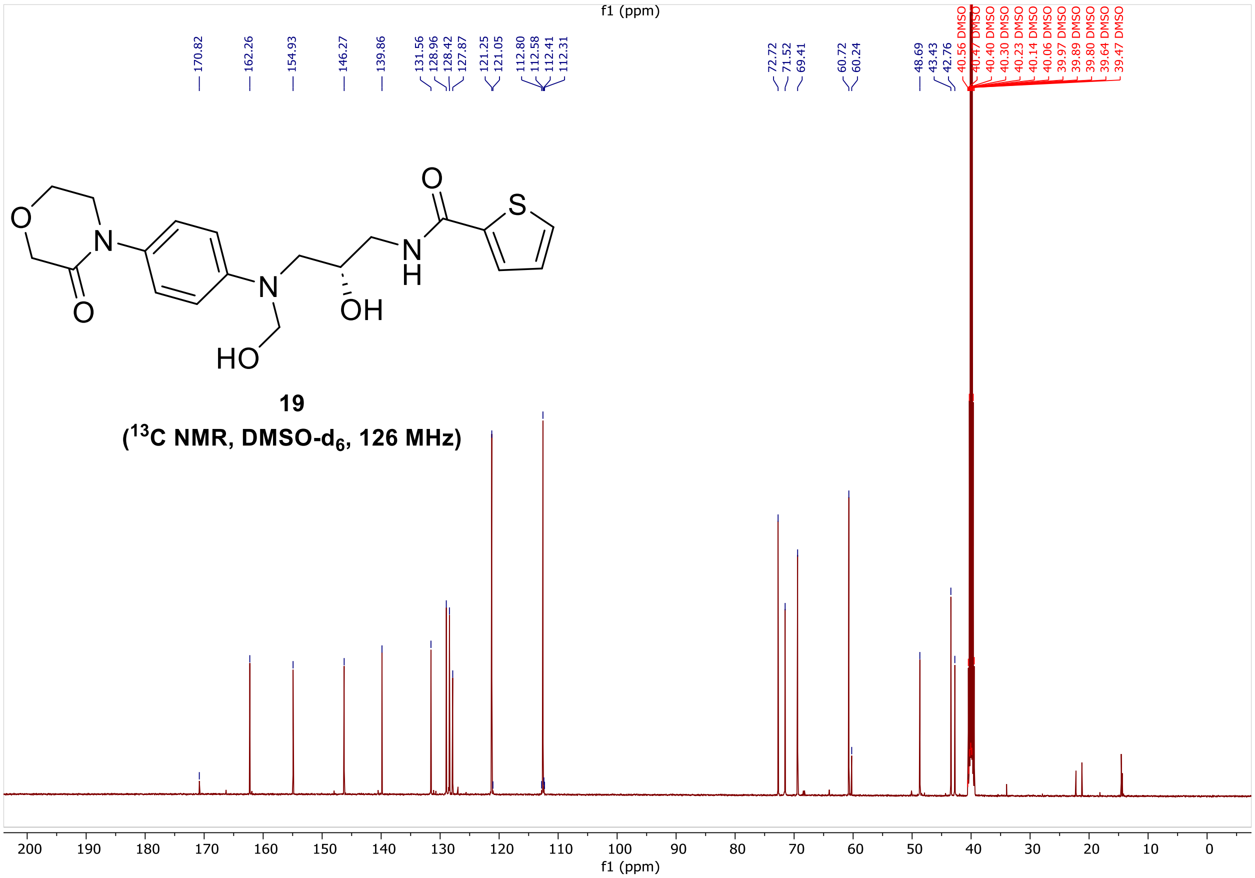
19
(¹³C NMR, DMSO-d6, 126 MHz)

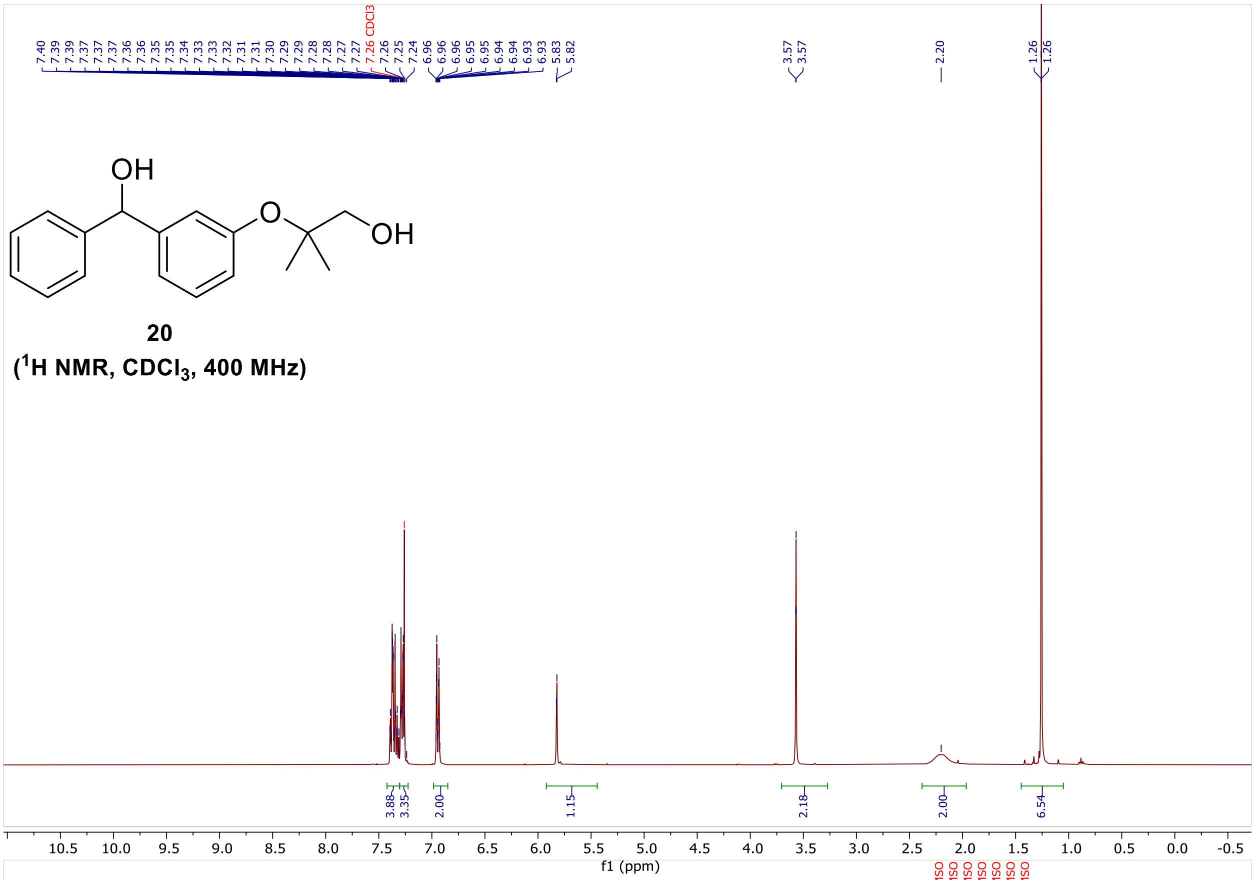

20
(^{1}H NMR, $CDCl_3$, 400 MHz)

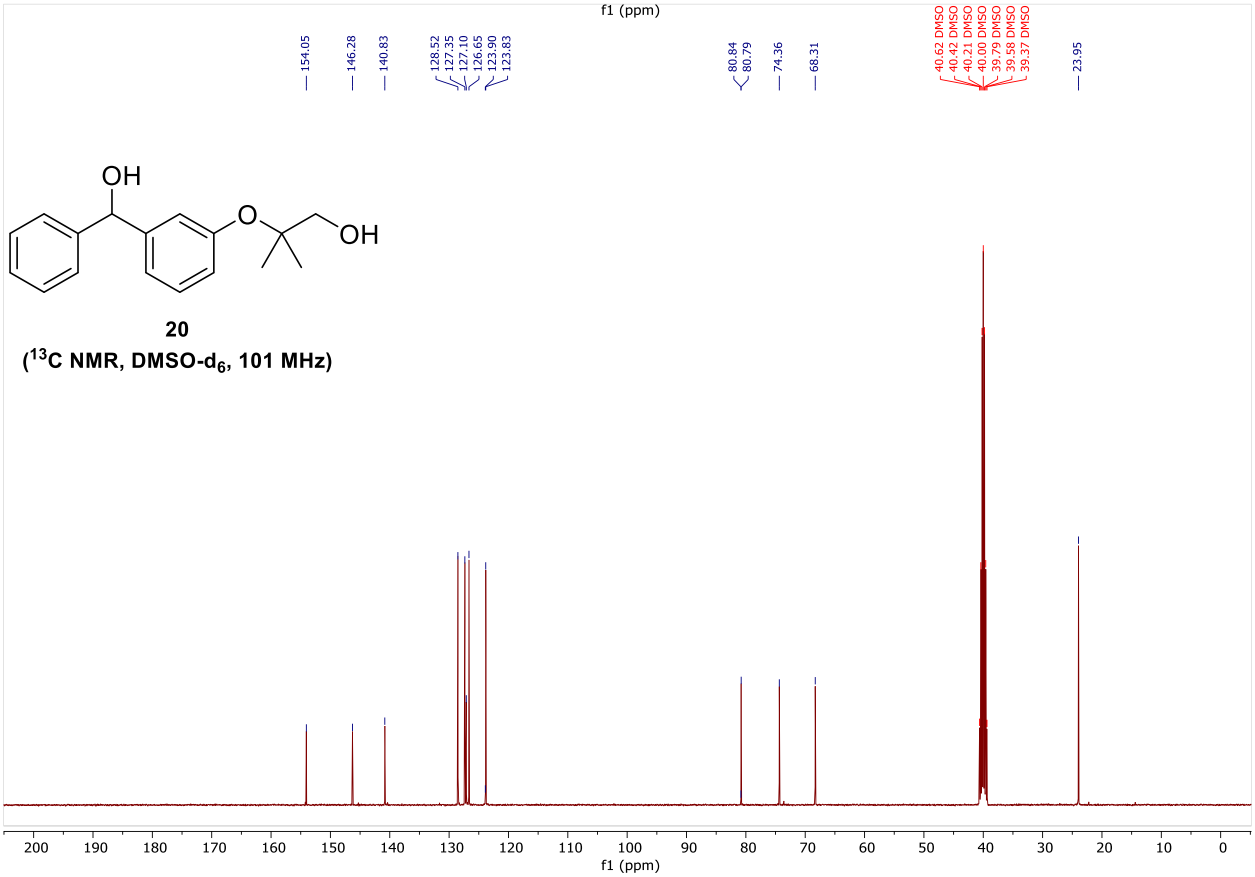

20
(^{13}C NMR, DMSO-d_6, 101 MHz)

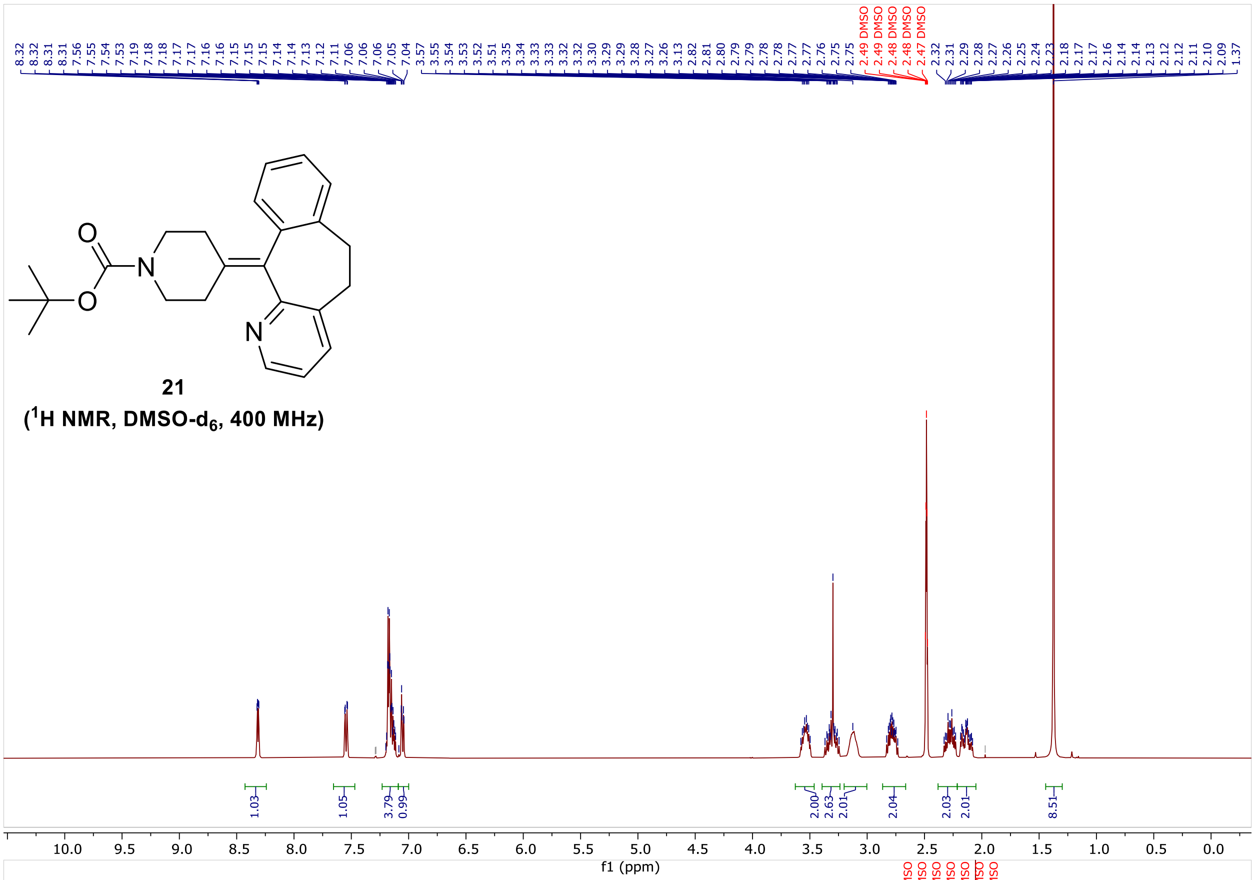

21

(^{1}H NMR, DMSO-d$_6$, 400 MHz)

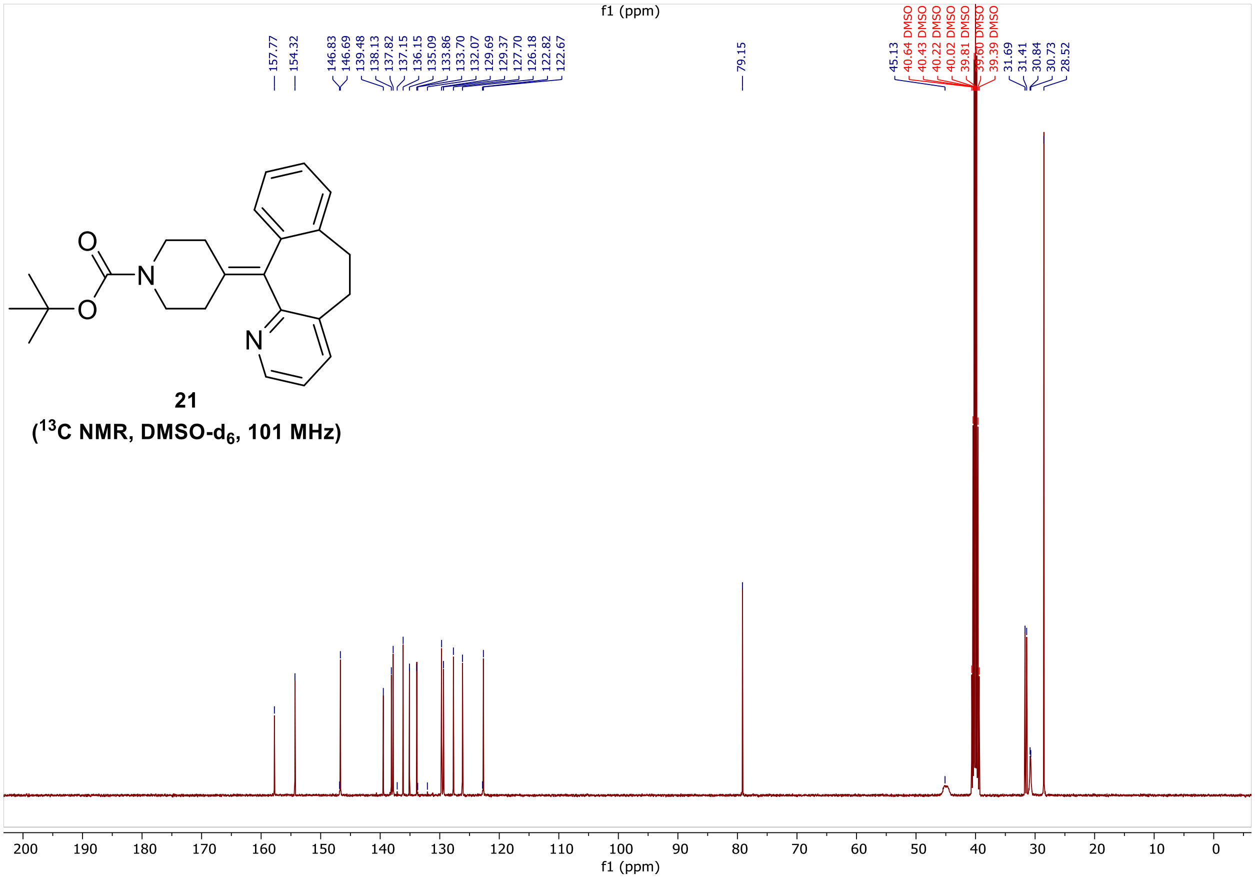

21

(^{13}C NMR, DMSO-d$_6$, 101 MHz)

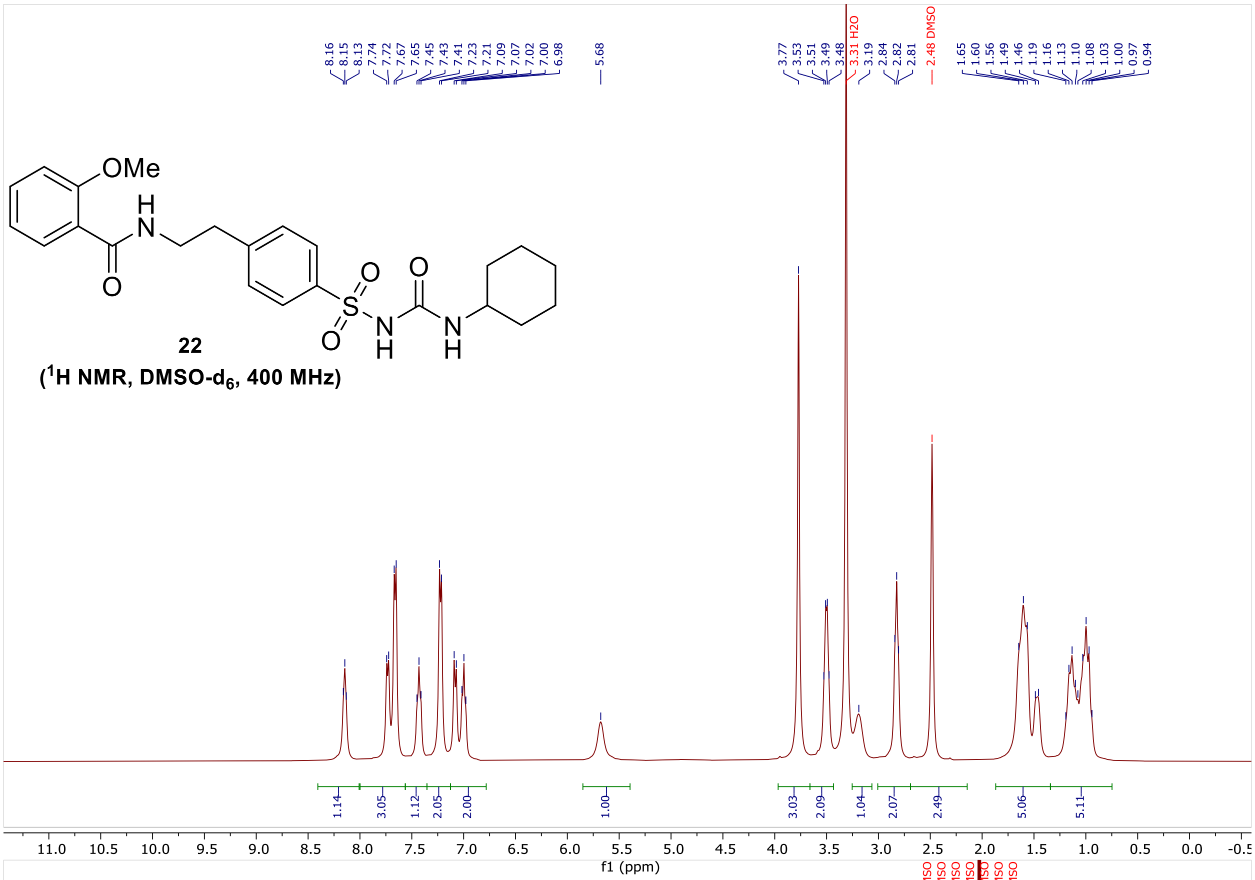
OMe
H
N
O
S
O
O
N
H
N
H
O
22
(1H NMR, DMSO-d6, 400 MHz)
8.16
8.15
8.13
7.74
7.72
7.67
7.65
7.45
7.43
7.41
7.23
7.21
7.09
7.07
7.02
7.00
6.98
5.68
3.77
3.53
3.51
3.49
3.48
3.31 H2O
3.19
2.84
2.82
2.81
2.48 DMSO
1.65
1.60
1.56
1.49
1.46
1.19
1.16
1.13
1.10
1.08
1.03
1.00
0.97
0.94
1.14
3.05
1.12
2.05
2.00
1.00
3.03
2.09
1.04
2.07
2.49
5.06
5.11
f1 (ppm)

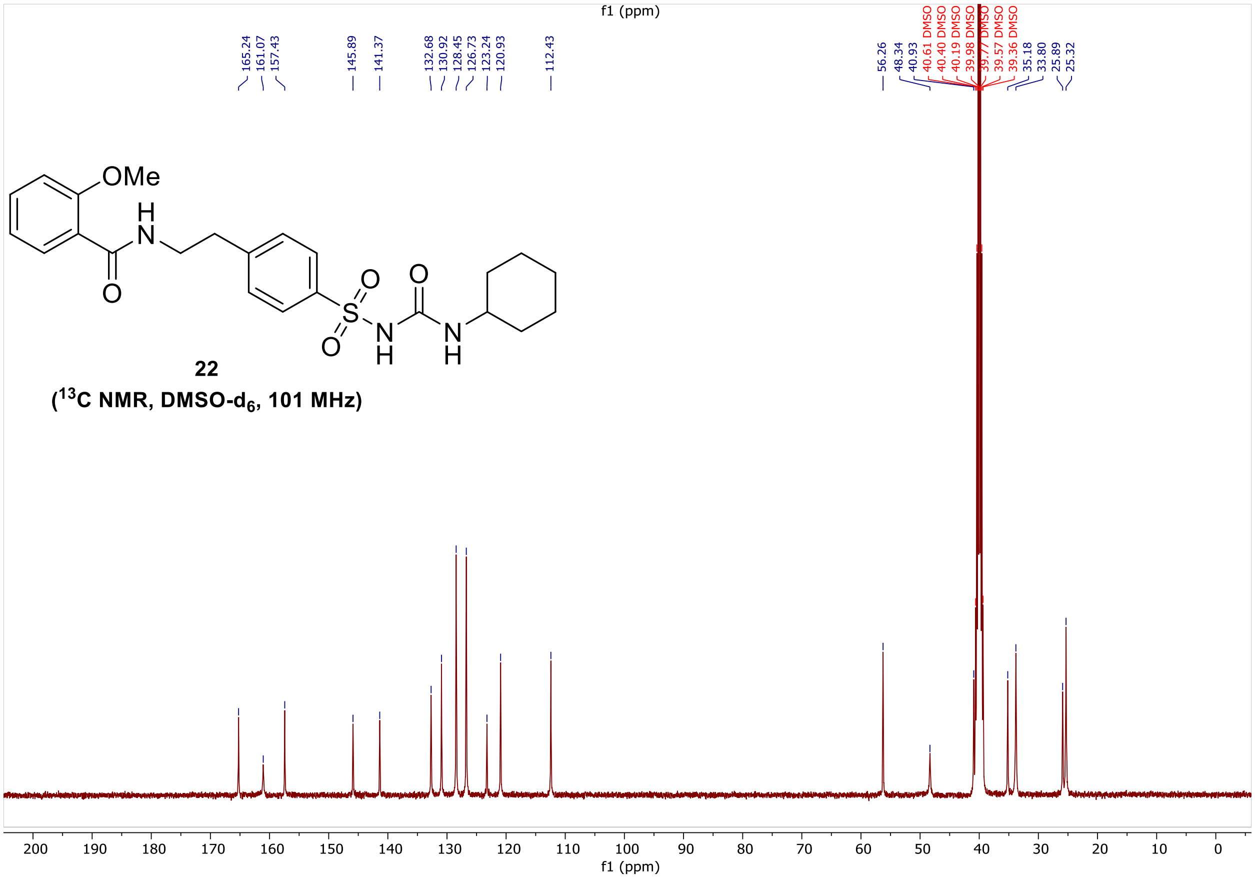
OMe
H
N
O
S
O
O
N
H
N
H
O
22
(13C NMR, DMSO-d6, 101 MHz)
165.24
161.07
157.43
145.89
141.37
132.68
130.92
128.45
126.73
123.24
120.93
112.43
56.26
48.34
40.93
40.61 DMSO
40.40 DMSO
40.19 DMSO
39.98 DMSO
39.77 DMSO
39.57 DMSO
39.36 DMSO
35.18
33.80
25.89
25.32
f1 (ppm)

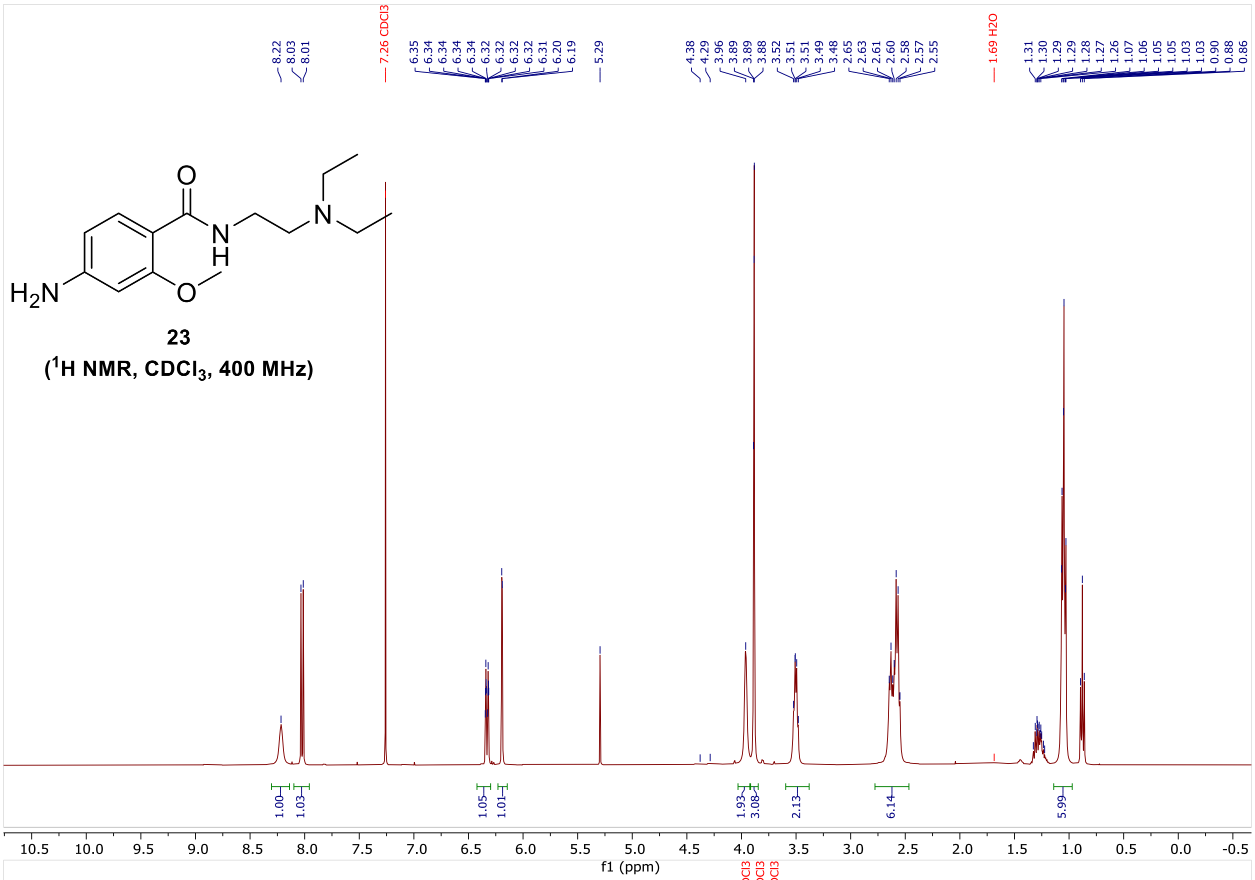
8.22
8.03
8.01
7.26 CDCl3
6.35 6.34 6.34 6.34 6.34 6.32 6.32 6.32 6.32 6.31 6.20 6.19
5.29
4.38 4.29 3.96 3.89 3.89 3.88 3.52 3.51 3.51 3.49 3.48 2.65 2.63 2.61 2.60 2.58 2.57 2.55
1.69 H2O
1.31 1.30 1.29 1.29 1.28 1.27 1.26 1.07 1.06 1.05 1.05 1.03 1.03 0.90 0.88 0.86
O
N
N
H
H2N
O
23
(1H NMR, CDCl3, 400 MHz)
1.00
1.03
1.05
1.01
1.93
3.08
2.13
6.14
5.99
10.5 10.0 9.5 9.0 8.5 8.0 7.5 7.0 6.5 6.0 5.5 5.0 4.5 4.0 3.5 3.0 2.5 2.0 1.5 1.0 0.5 0.0 -0.5
f1 (ppm)

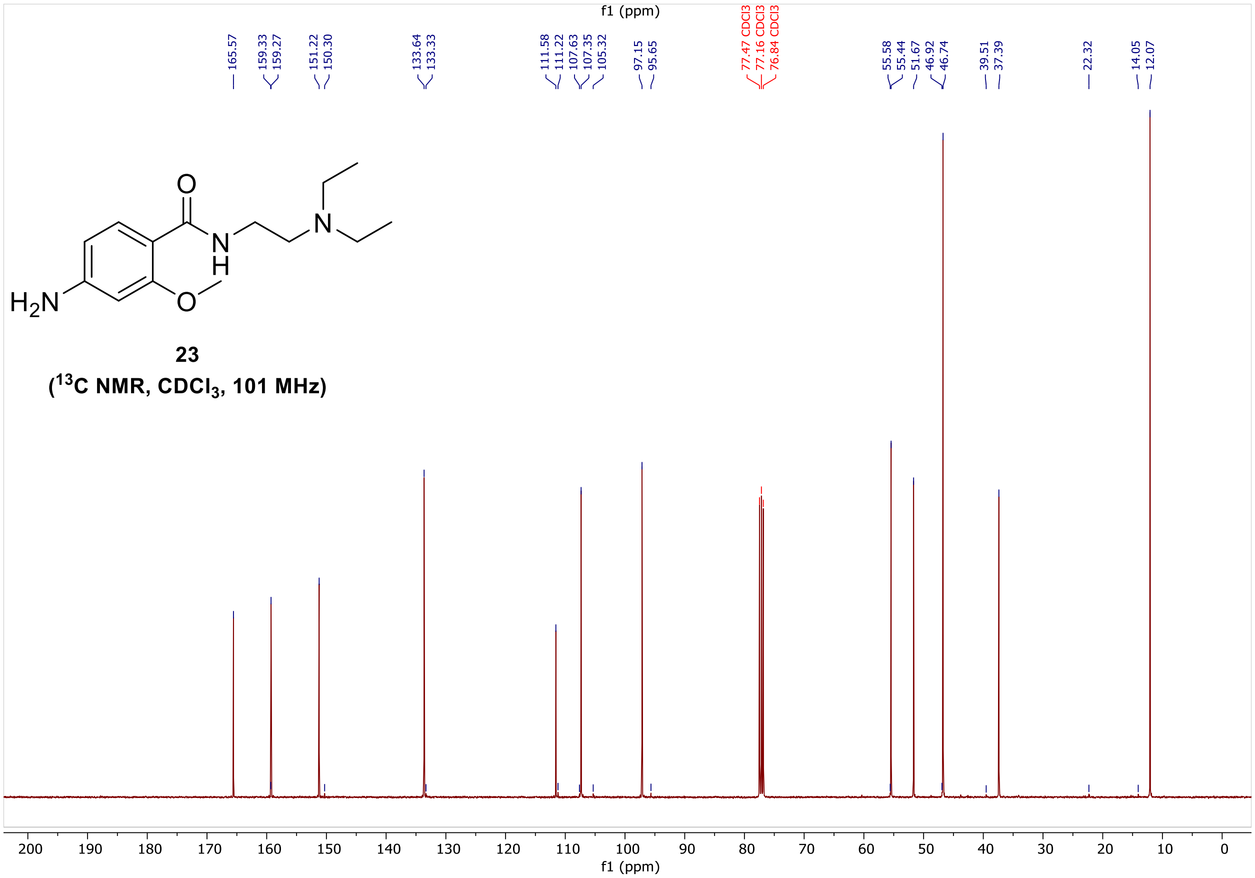
165.57
159.33
159.27
151.22
150.30
133.64
133.33
111.58
111.22
107.63
107.35
105.32
97.15
95.65
77.47 CDCl3
77.16 CDCl3
76.84 CDCl3
55.58
55.44
51.67
46.92
46.74
39.51
37.39
22.32
14.05
12.07
O
N
N
H
H2N
O
23
(13C NMR, CDCl3, 101 MHz)
200 190 180 170 160 150 140 130 120 110 100 90 80 70 60 50 40 30 20 10 0
f1 (ppm)

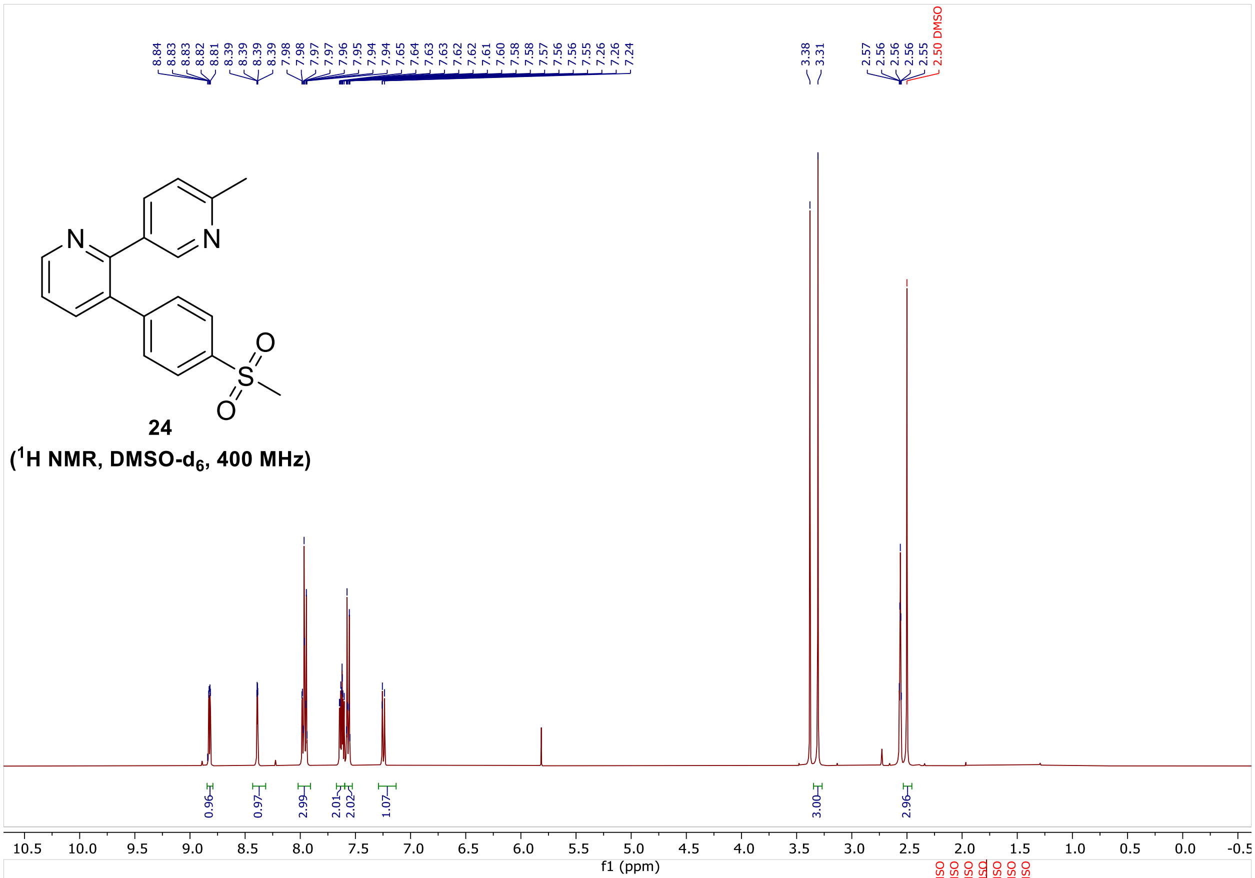

24

(^{1}H NMR, DMSO-d_6, 400 MHz)

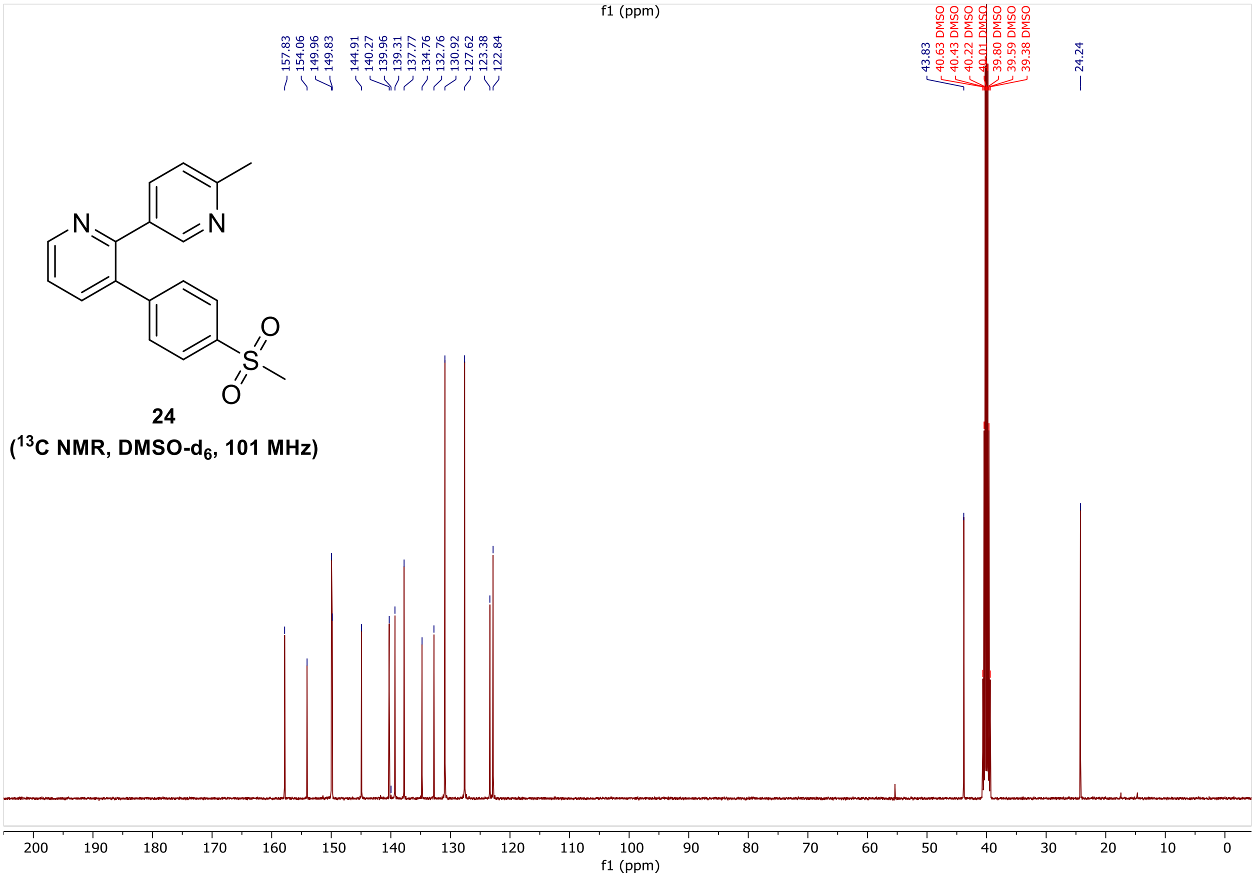

24

(^{13}C NMR, DMSO-d_6, 101 MHz)

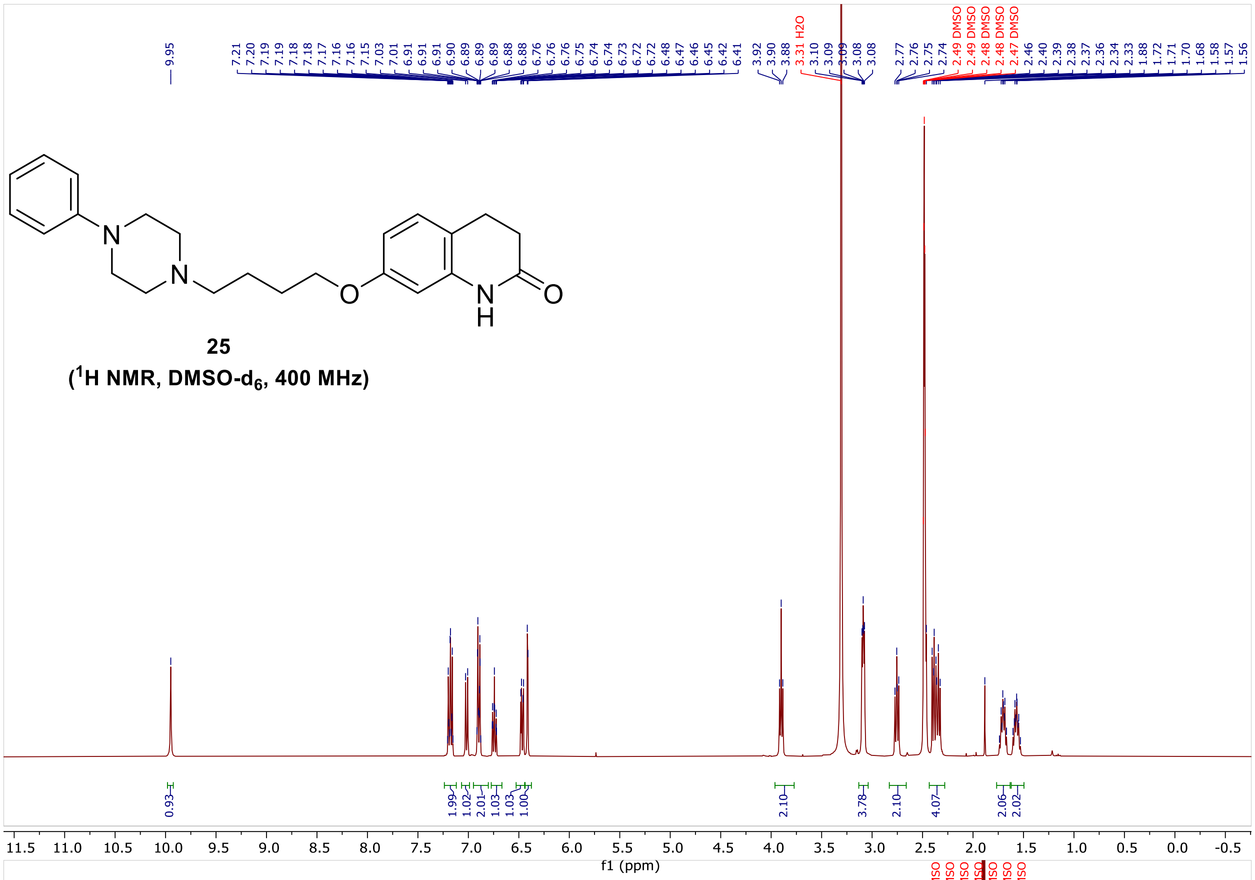

25

(^{1}H NMR, DMSO-d$_6$, 400 MHz)

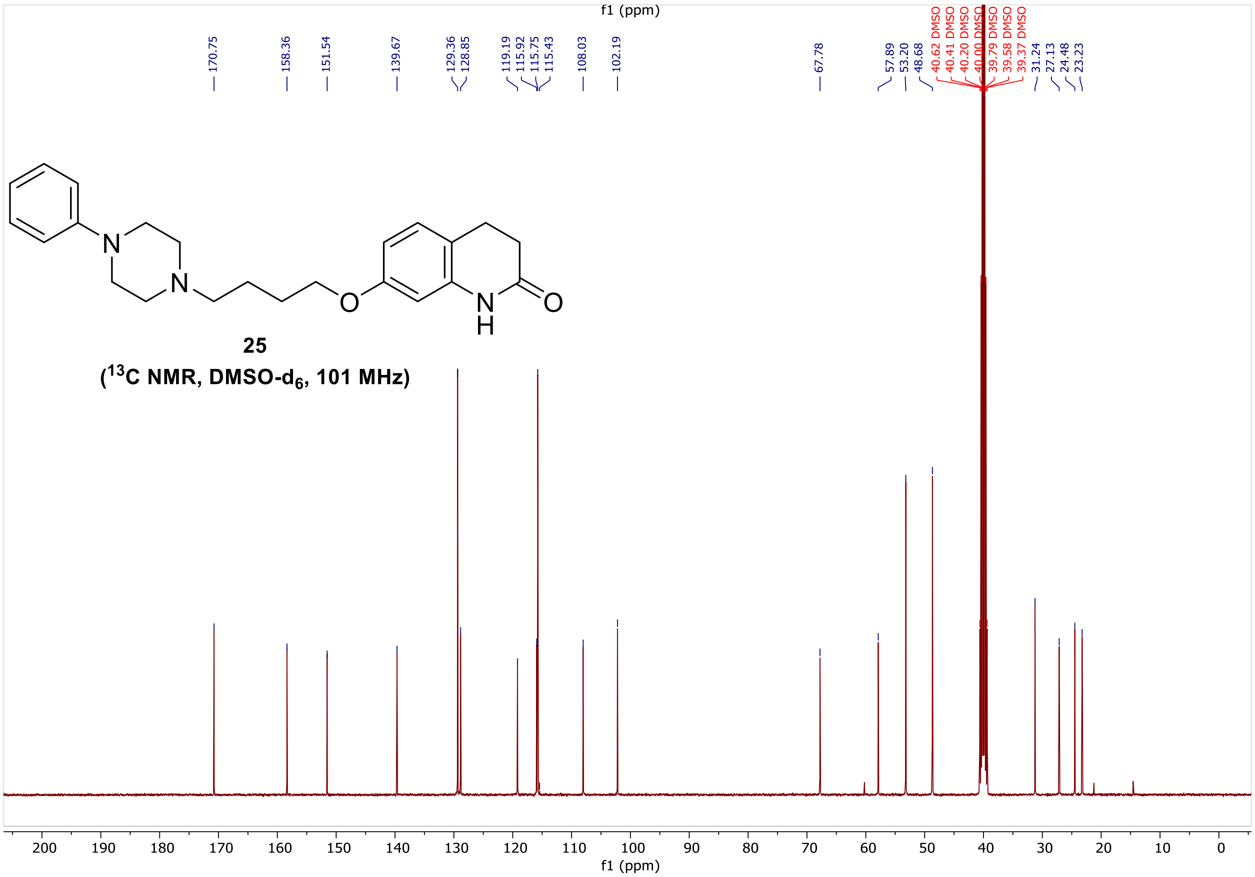

25

(^{13}C NMR, DMSO-d$_6$, 101 MHz)

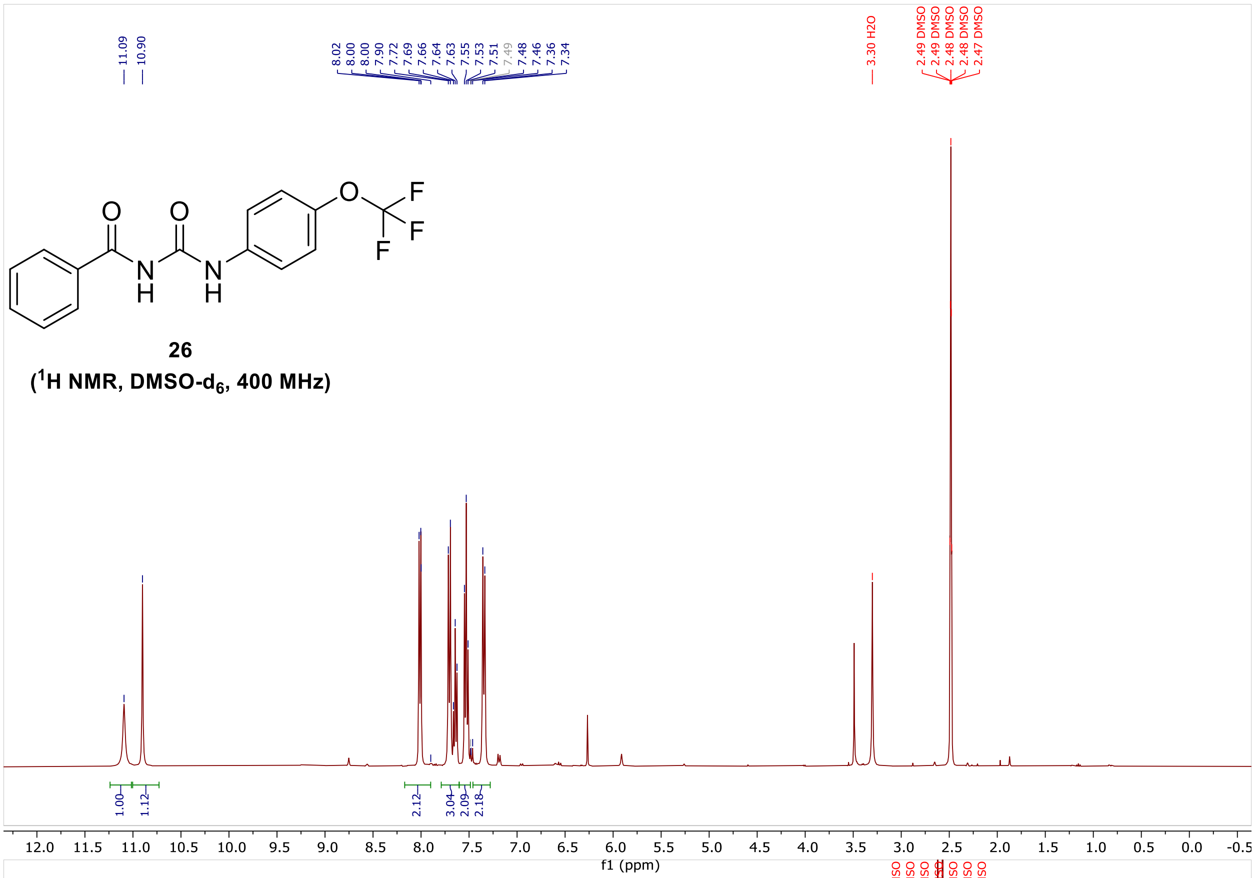

26
(^{1}H NMR, DMSO-d_6, 400 MHz)

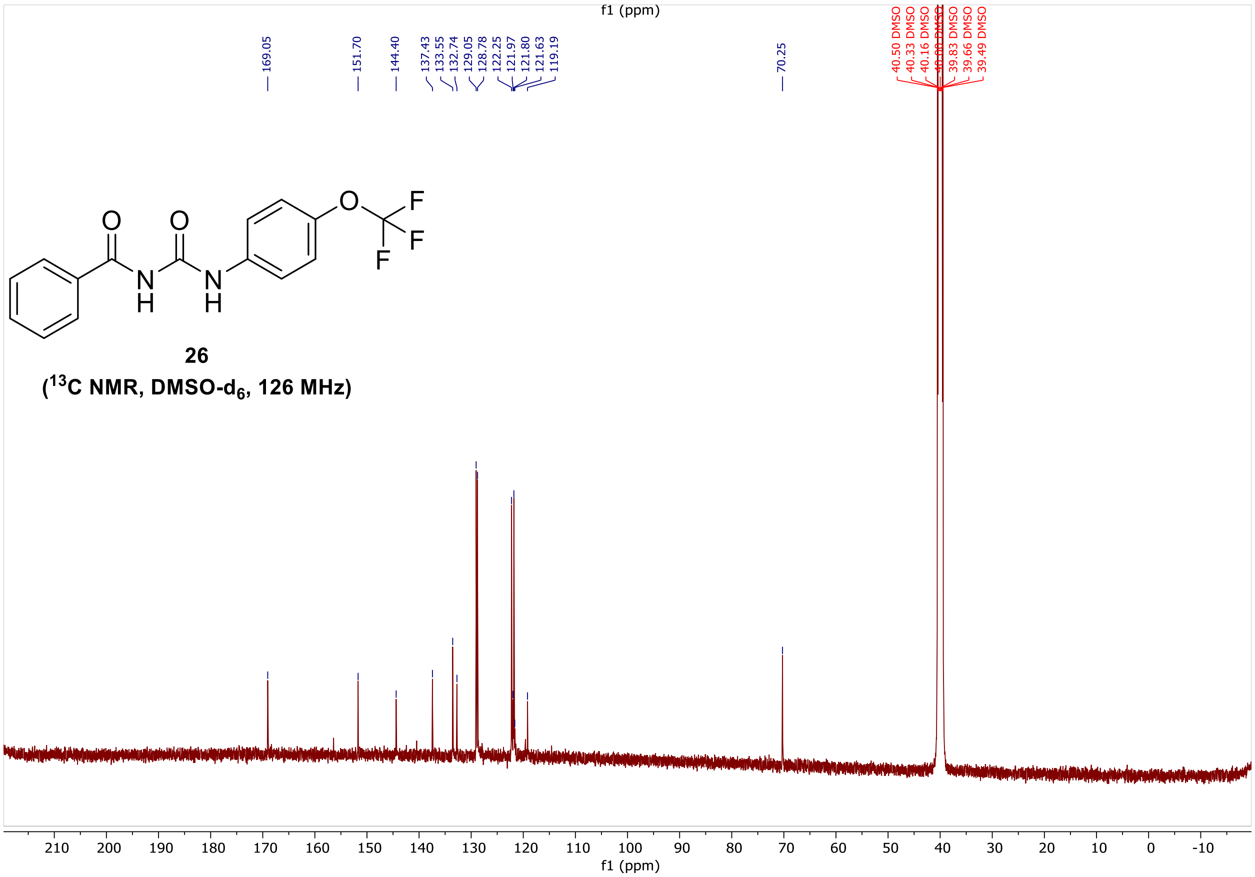

26
(^{13}C NMR, DMSO-d_6, 126 MHz)

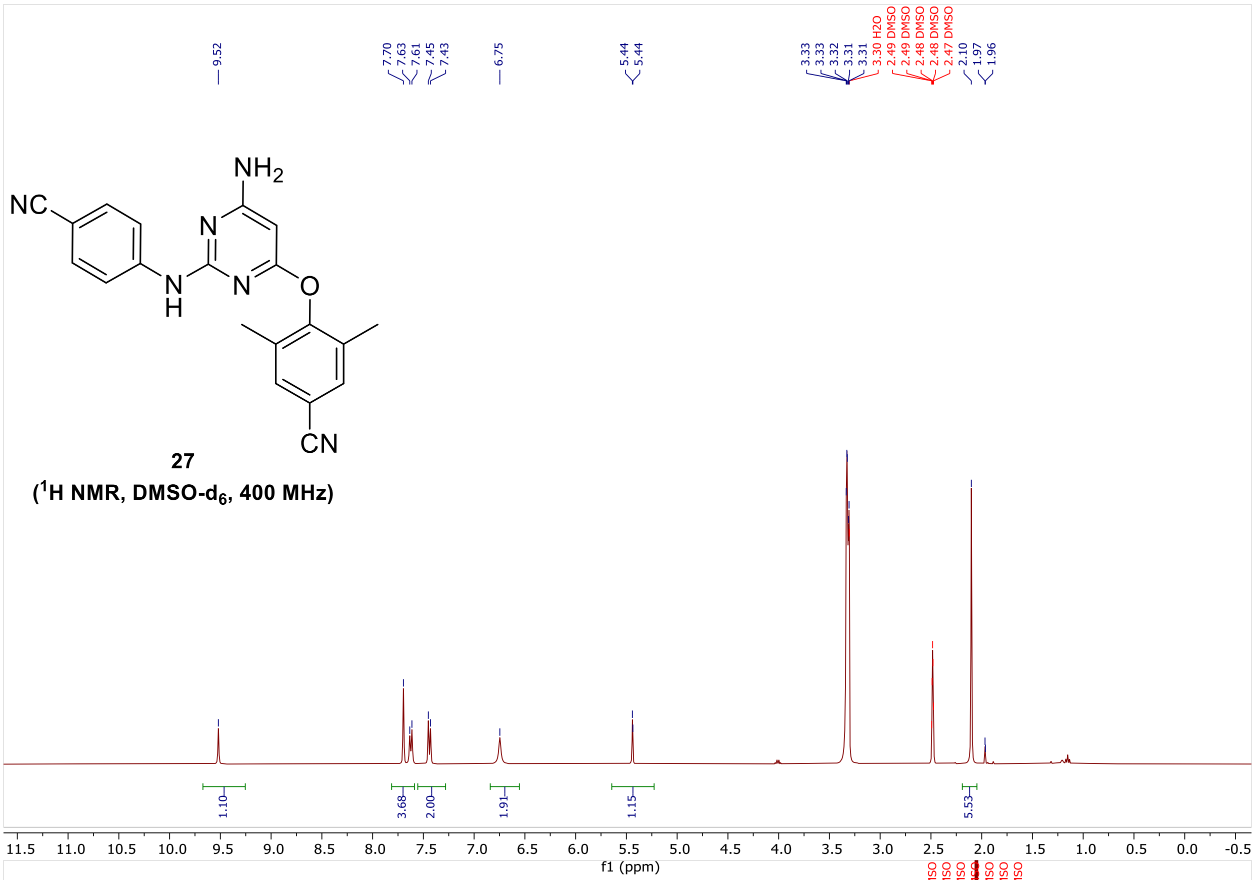
27
(^{1}H NMR, DMSO-d$_6$, 400 MHz)
f1 (ppm)

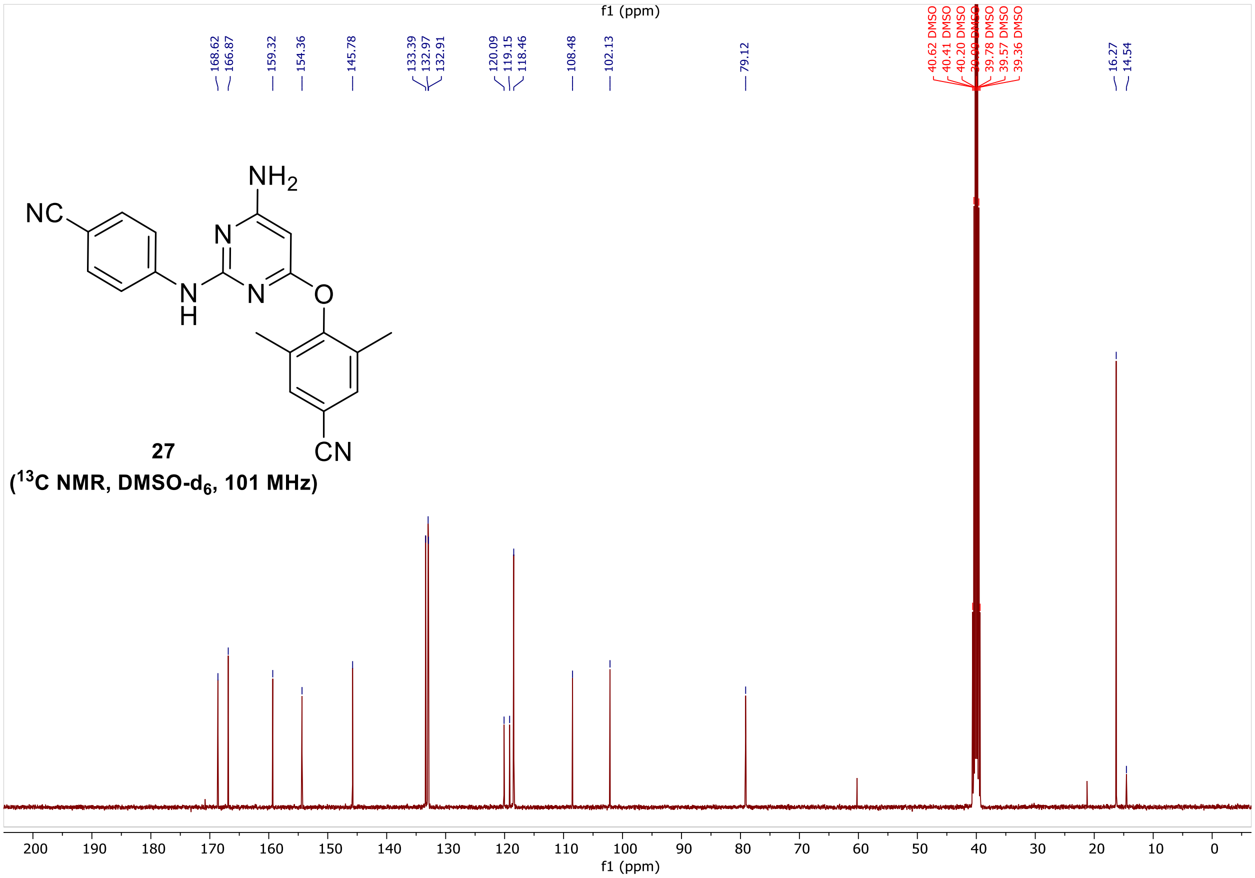
27
(^{13}C NMR, DMSO-d$_6$, 101 MHz)
f1 (ppm)

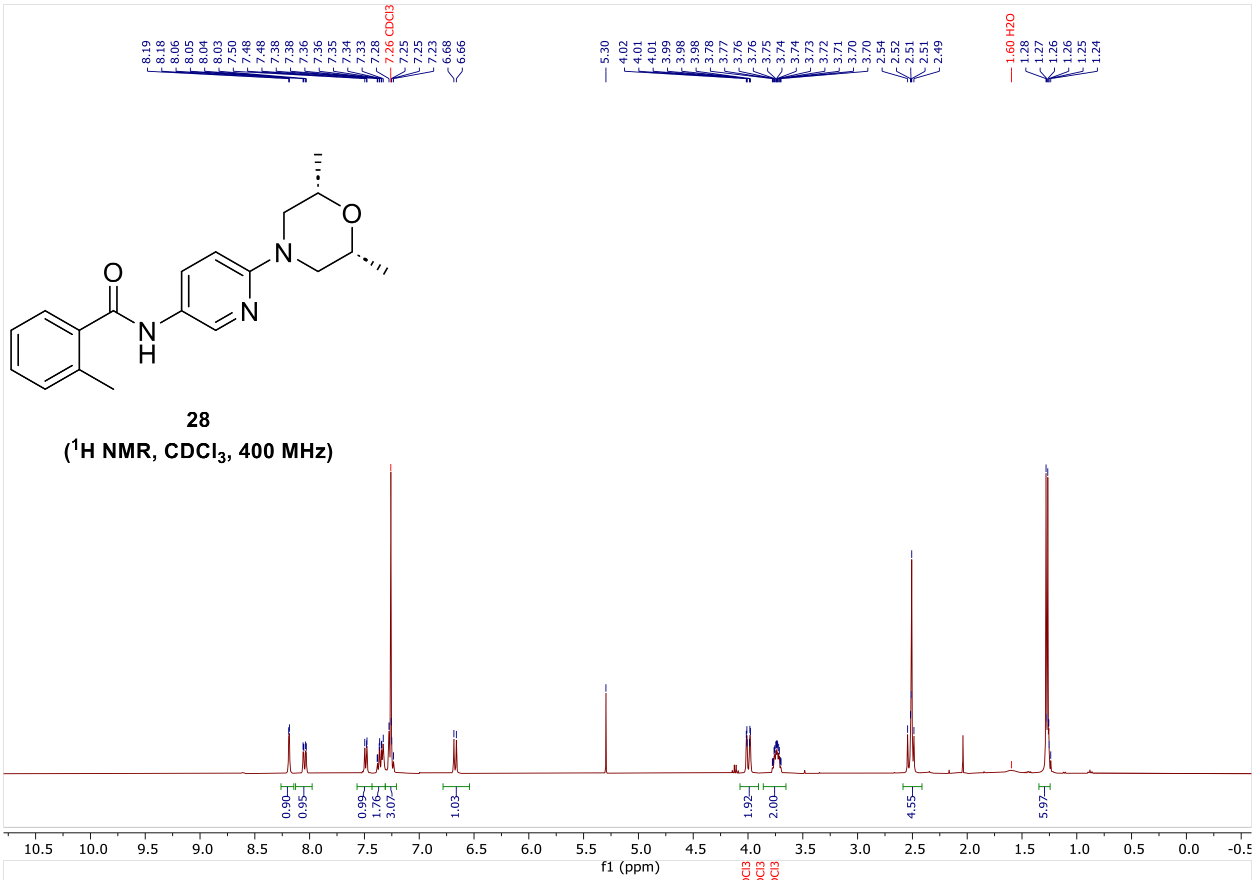
28
(¹H NMR, CDCl3, 400 MHz)
7.26 CDCl3
1.60 H2O
f1 (ppm)

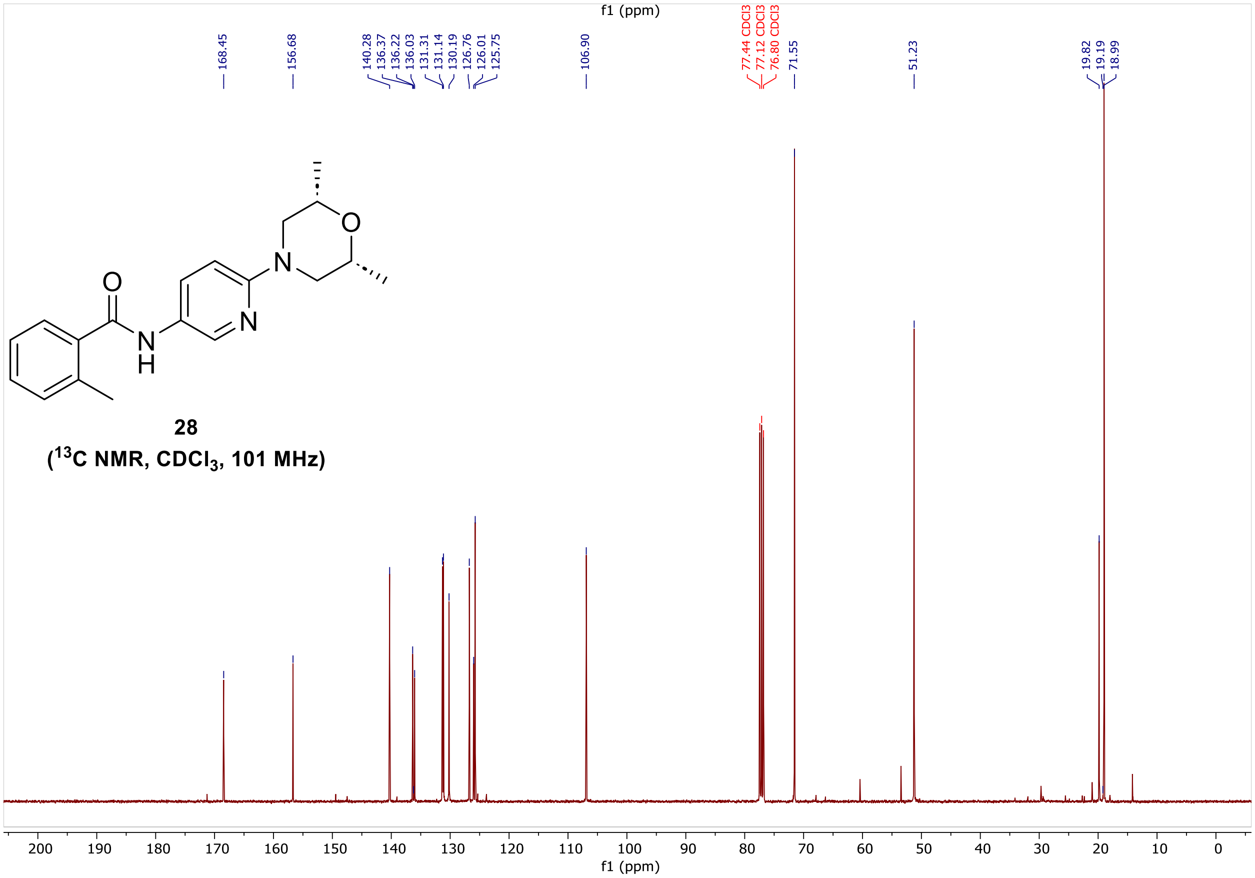
28
(¹³C NMR, CDCl3, 101 MHz)
77.44 CDCl3
77.12 CDCl3
76.80 CDCl3
f1 (ppm)

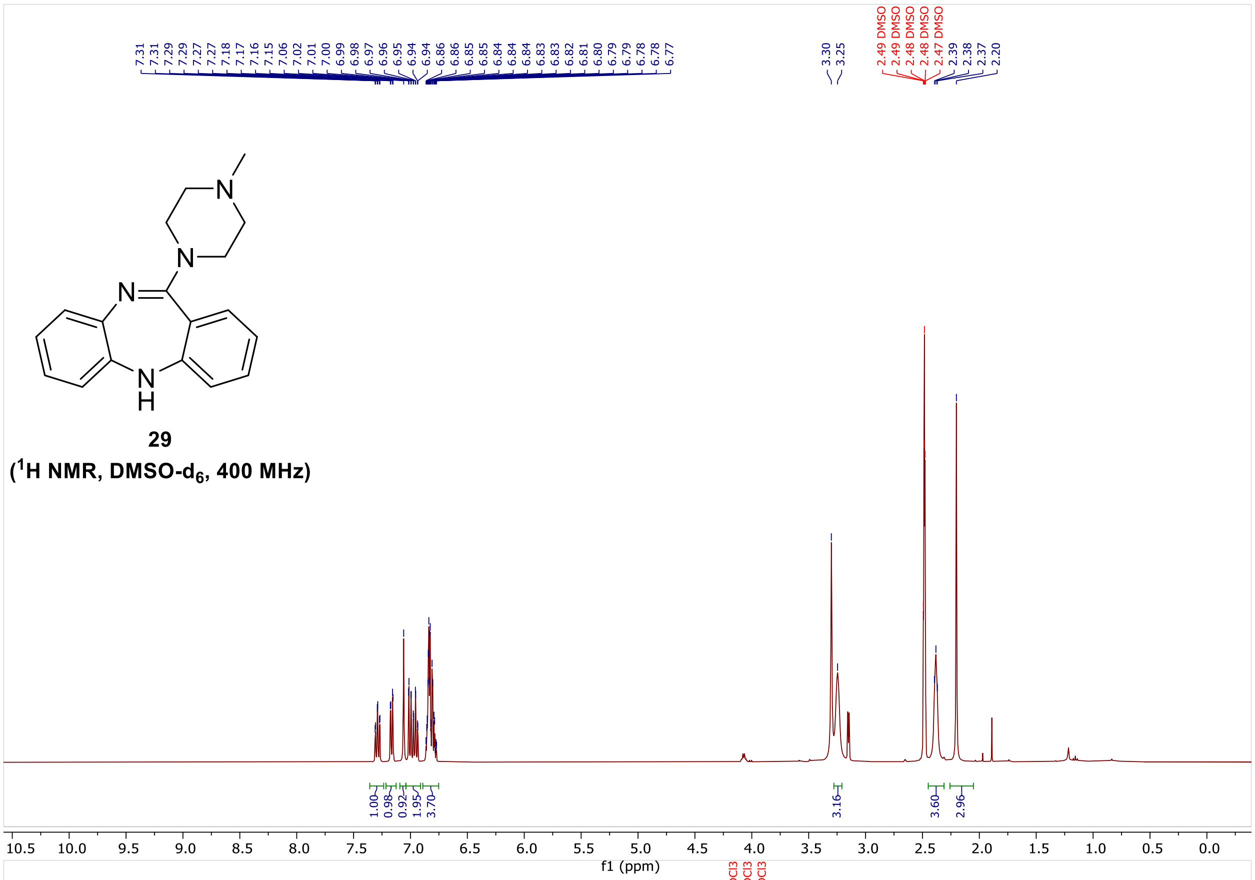

29

(^{1}H NMR, DMSO-d_6, 400 MHz)

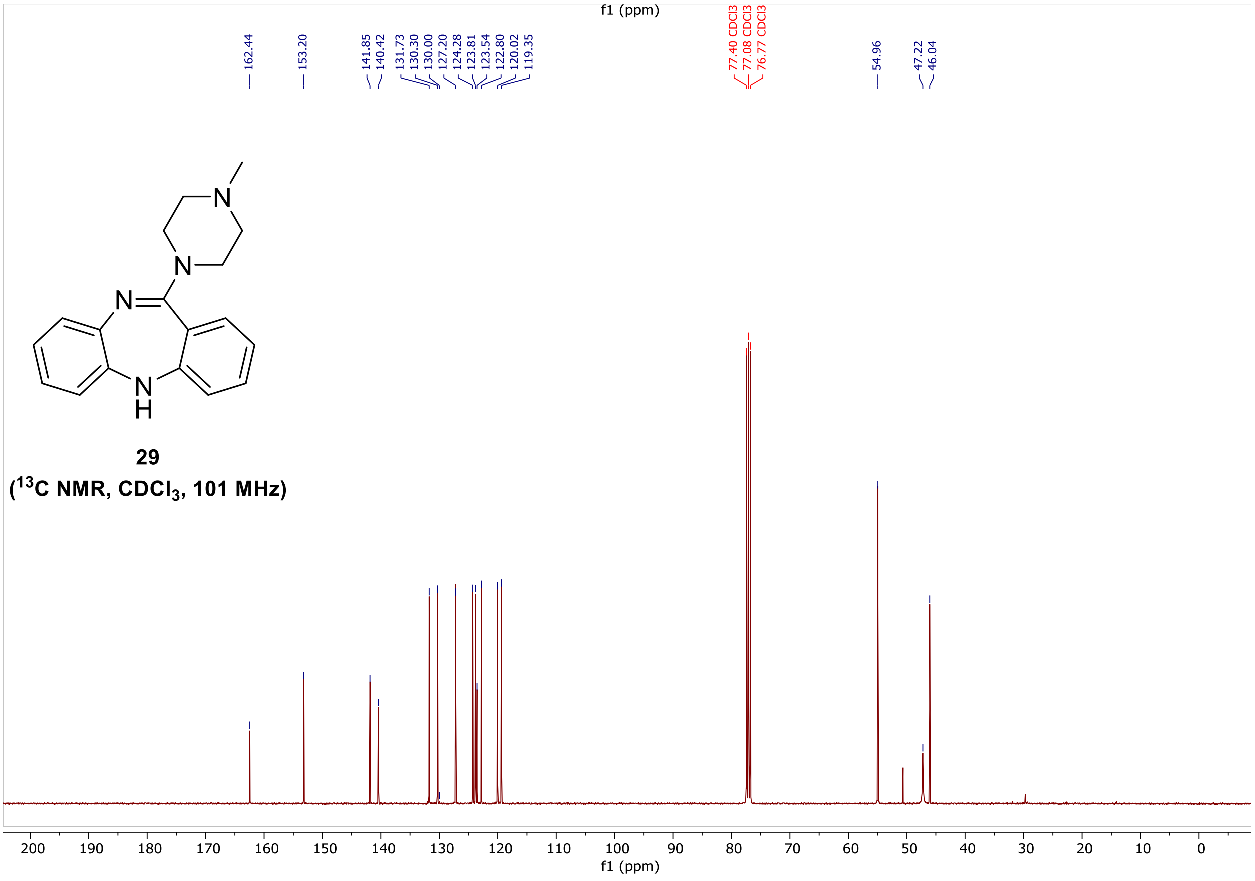

29

(^{13}C NMR, $CDCl_3$, 101 MHz)

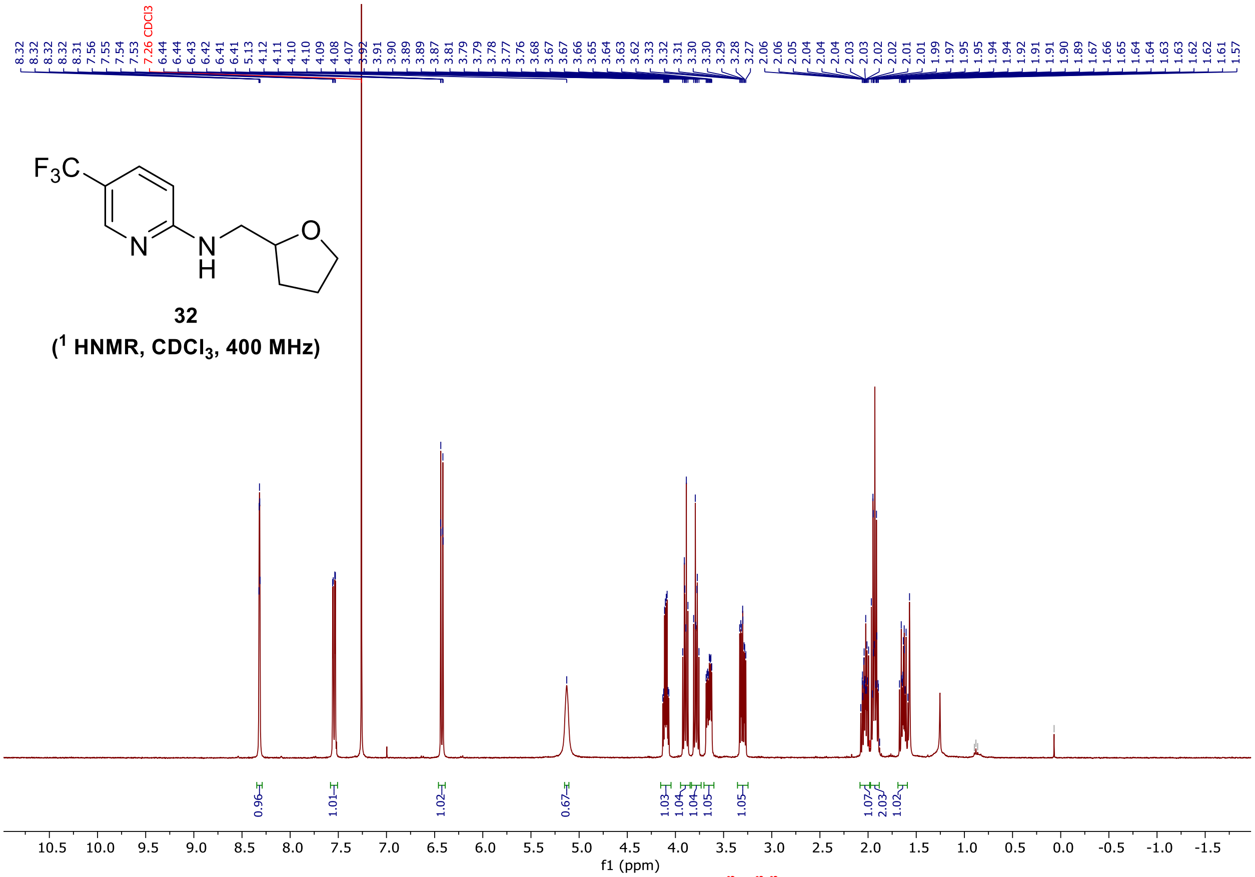

32

(^{1} HNMR, $CDCl_3$, 400 MHz)

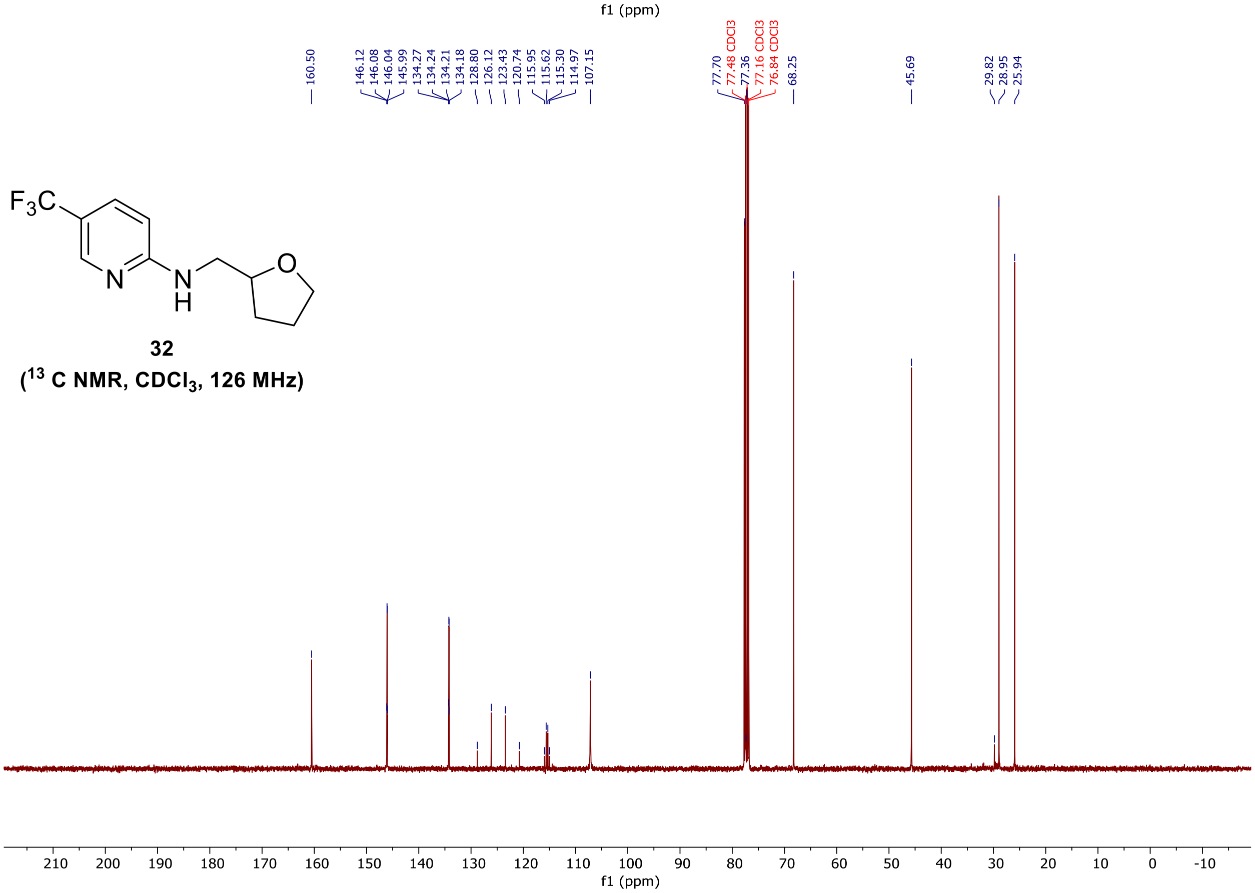

32

(^{13} C NMR, $CDCl_3$, 126 MHz)

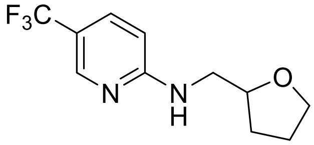

32

(^{19} F NMR, $CDCl_3$, 376 MHz)

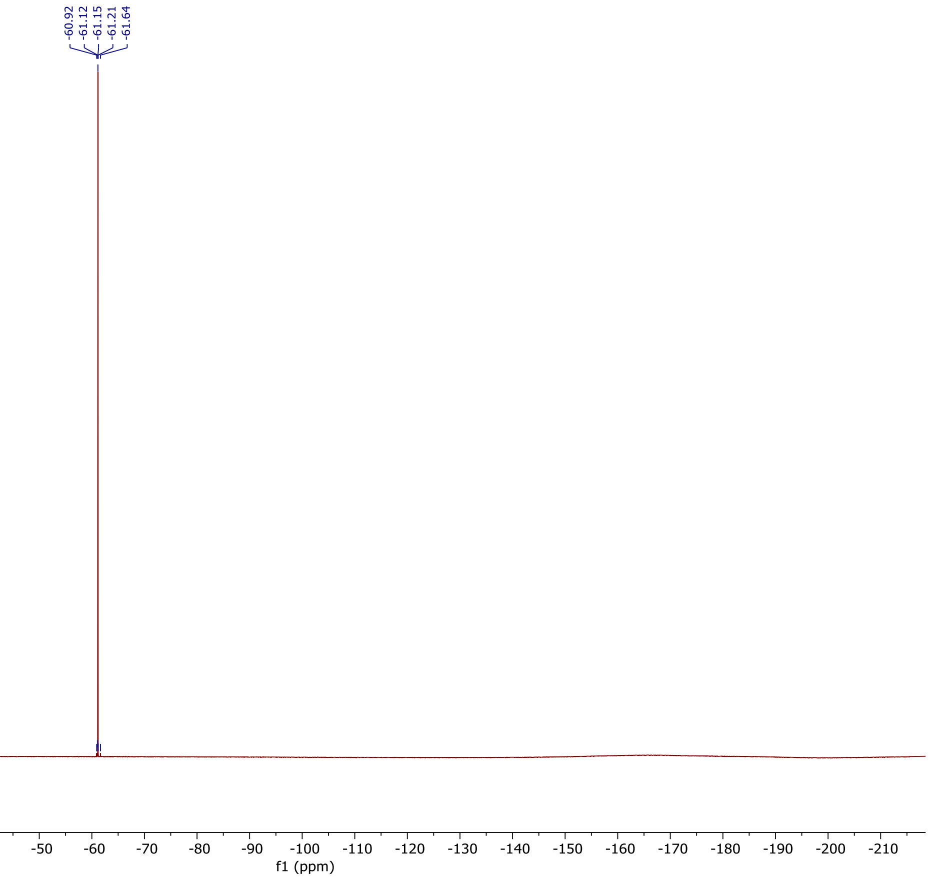

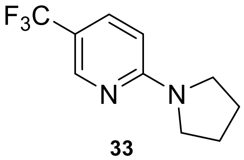

33

(^{1} HNMR, $CDCl_3$, 400 MHz)

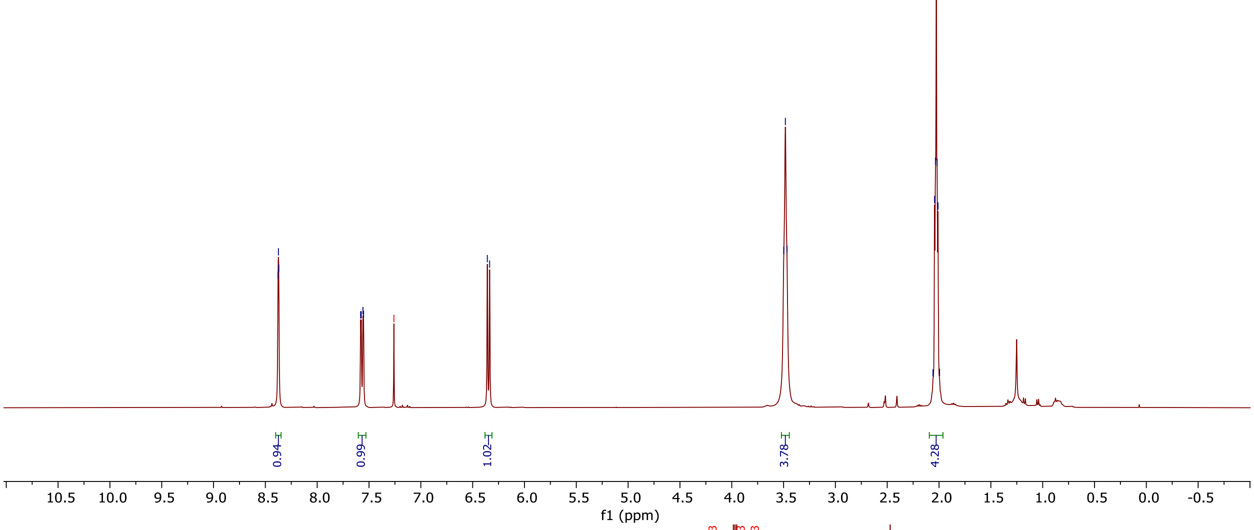

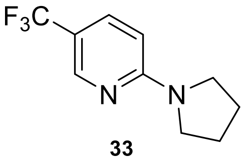

33

(^{13} C NMR, $CDCl_3$, 126 MHz)

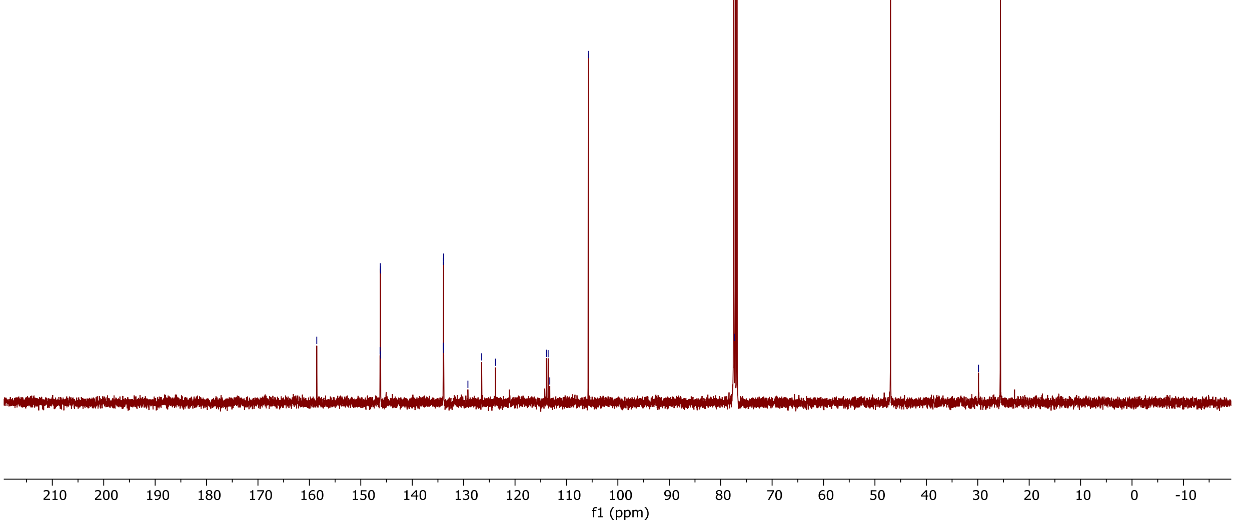

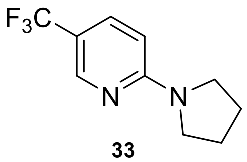

33

(^{19} FNMR, $CDCl_3$, 379 MHz)

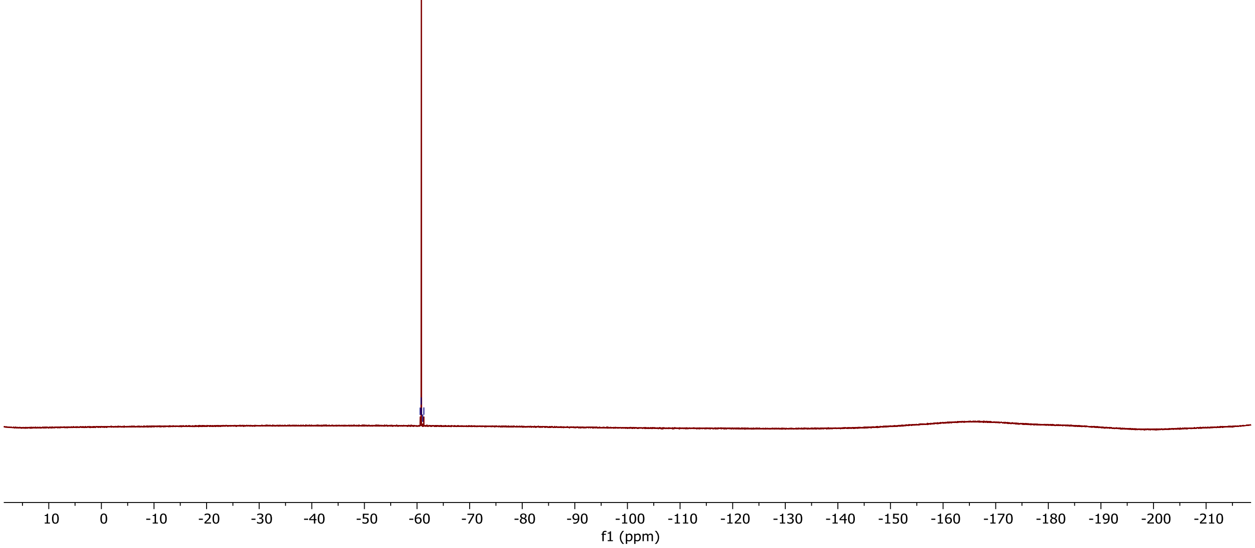

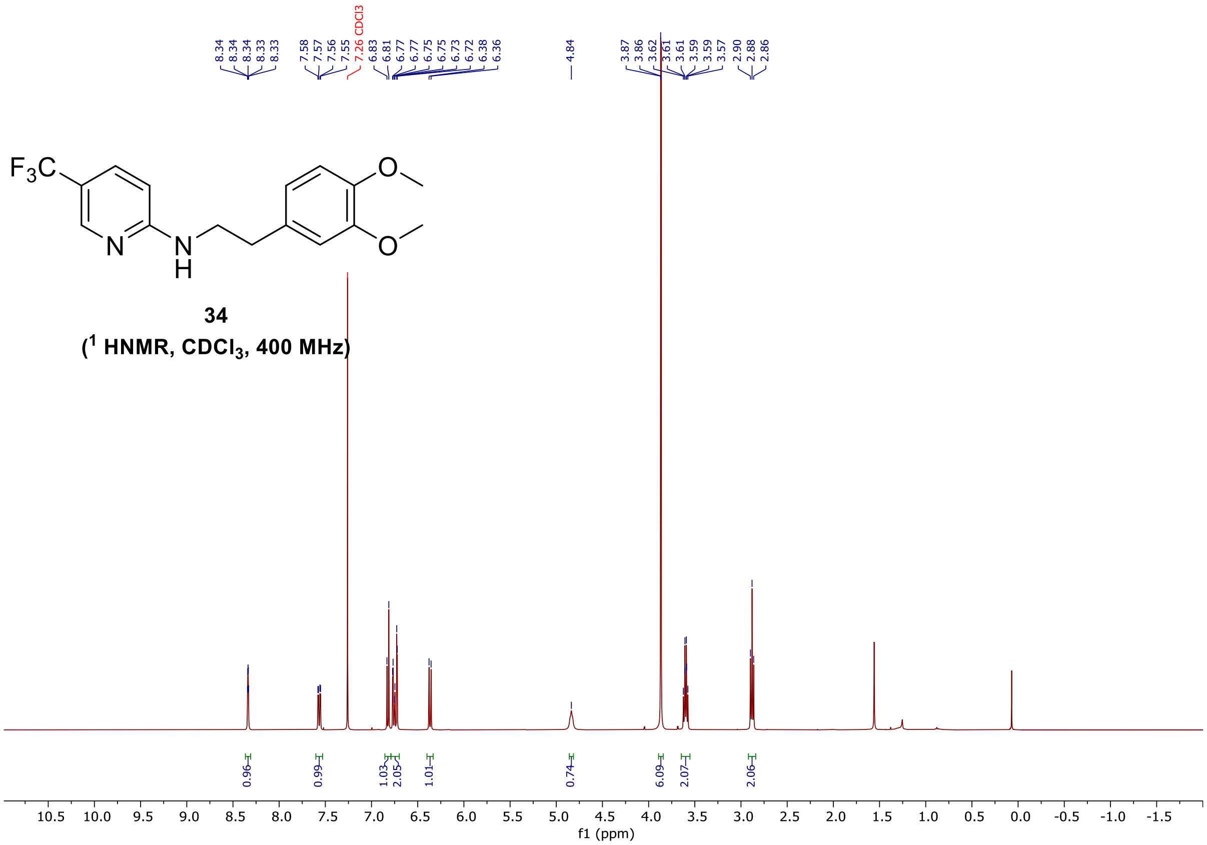
8.34
8.34
8.34
8.33
8.33
7.58
7.57
7.56
7.55
7.26 CDCl3
6.83
6.81
6.77
6.77
6.75
6.75
6.73
6.72
6.38
6.36
4.84
3.87
3.86
3.62
3.61
3.61
3.59
3.59
3.57
2.90
2.88
2.86
F3C
N
N
H
O
O
34
(1 HNMR, CDCl3, 400 MHz)
0.96
0.99
1.03
2.05
1.01
0.74
6.09
2.07
2.06
f1 (ppm)

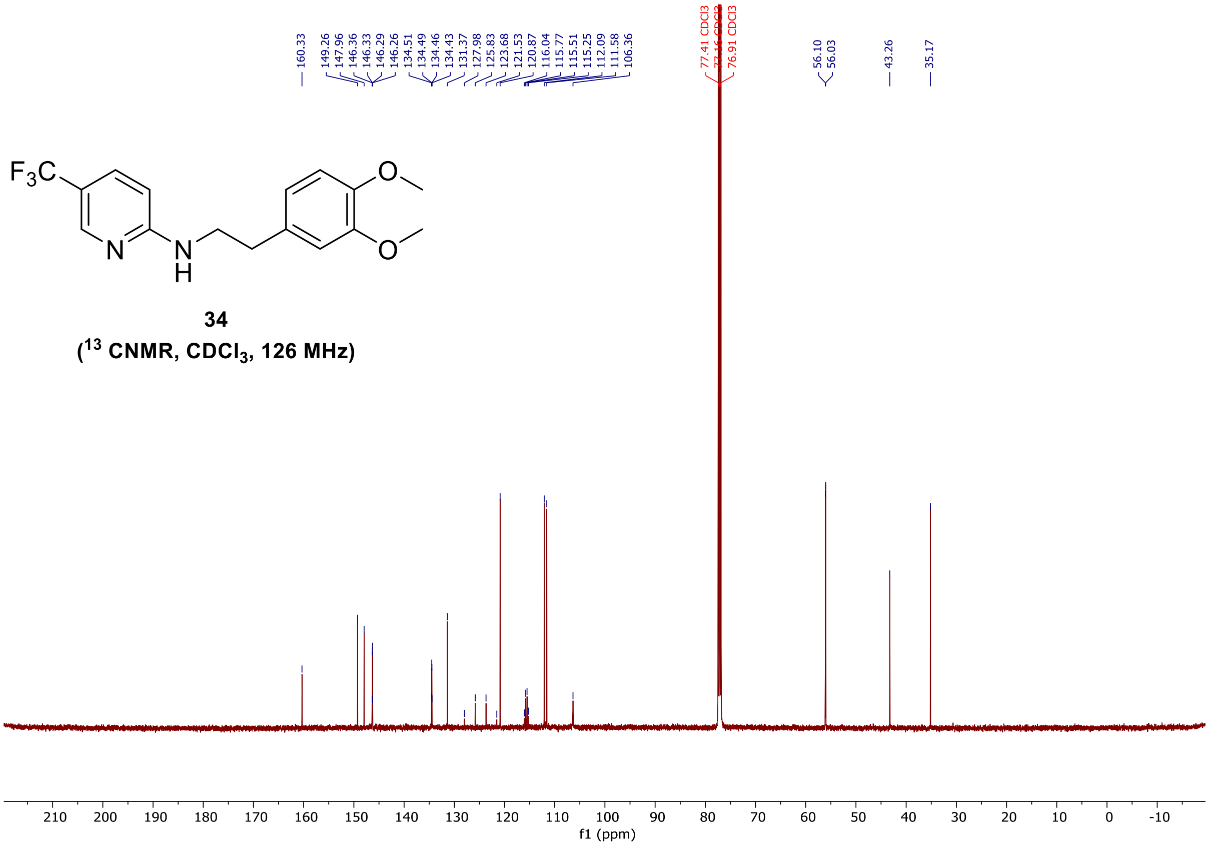
160.33
149.26
147.96
146.36
146.33
146.29
146.26
134.51
134.49
134.46
134.43
131.37
127.98
125.83
123.68
121.53
120.87
116.04
115.77
115.51
115.25
112.09
111.58
106.36
77.41 CDCl3
76.91 CDCl3
56.10
56.03
43.26
35.17
F3C
N
N
H
O
O
34
(13 CNMR, CDCl3, 126 MHz)
f1 (ppm)

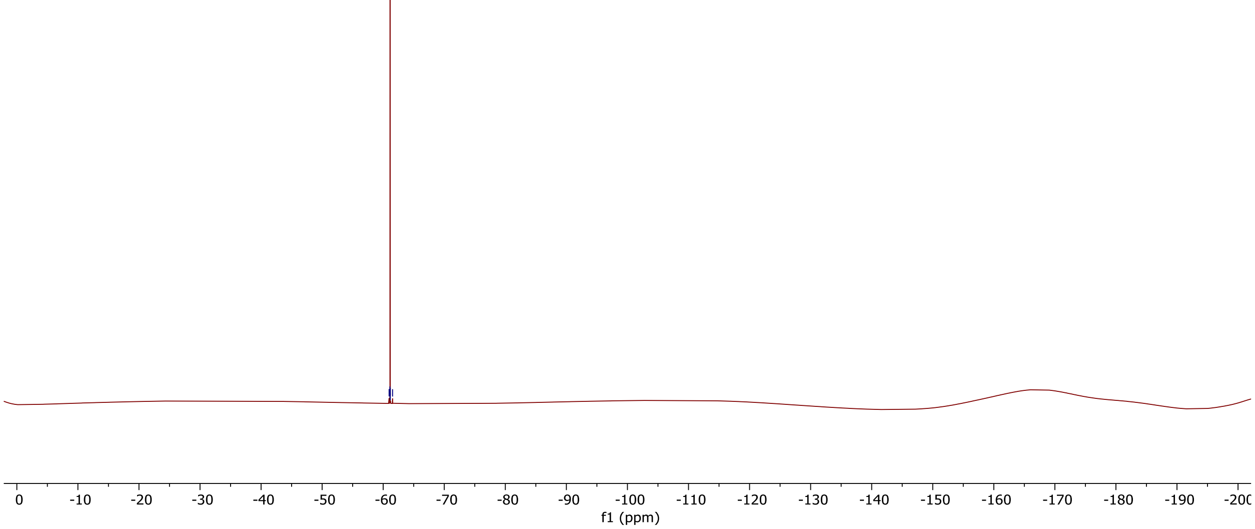

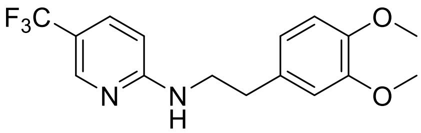

34

(^{19} FNMR, $CDCl_3$, 376 MHz)

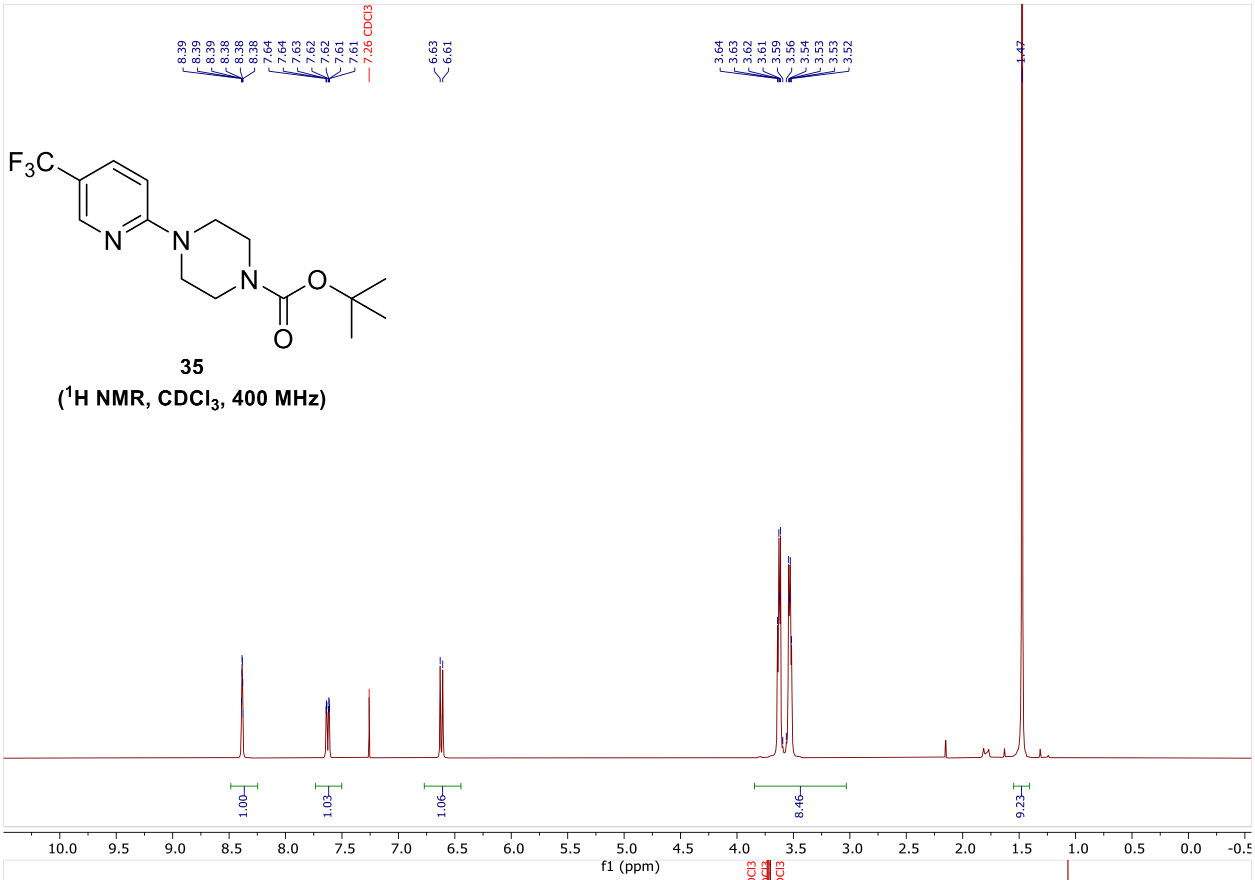

35
(^{1}H NMR, $CDCl_3$, 400 MHz)

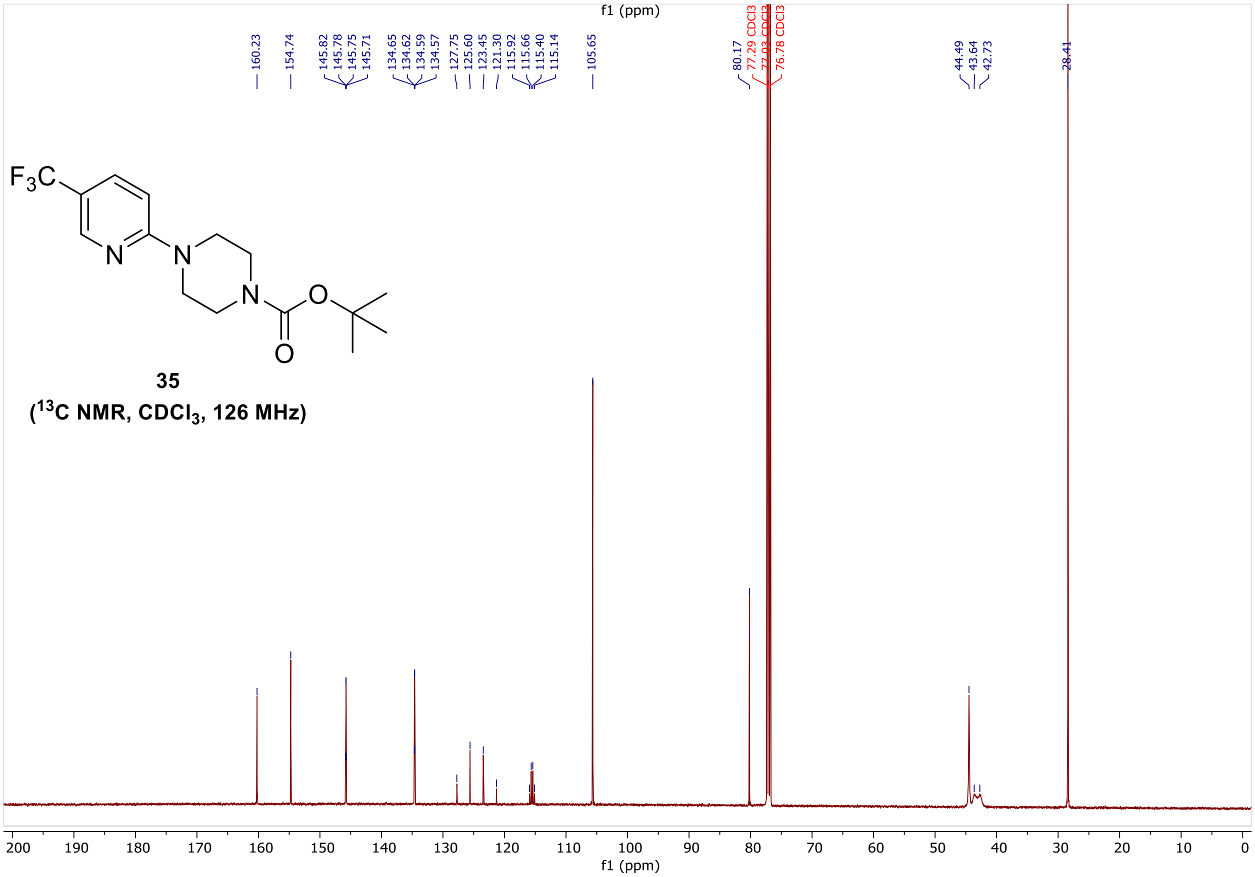

35
(^{13}C NMR, $CDCl_3$, 126 MHz)

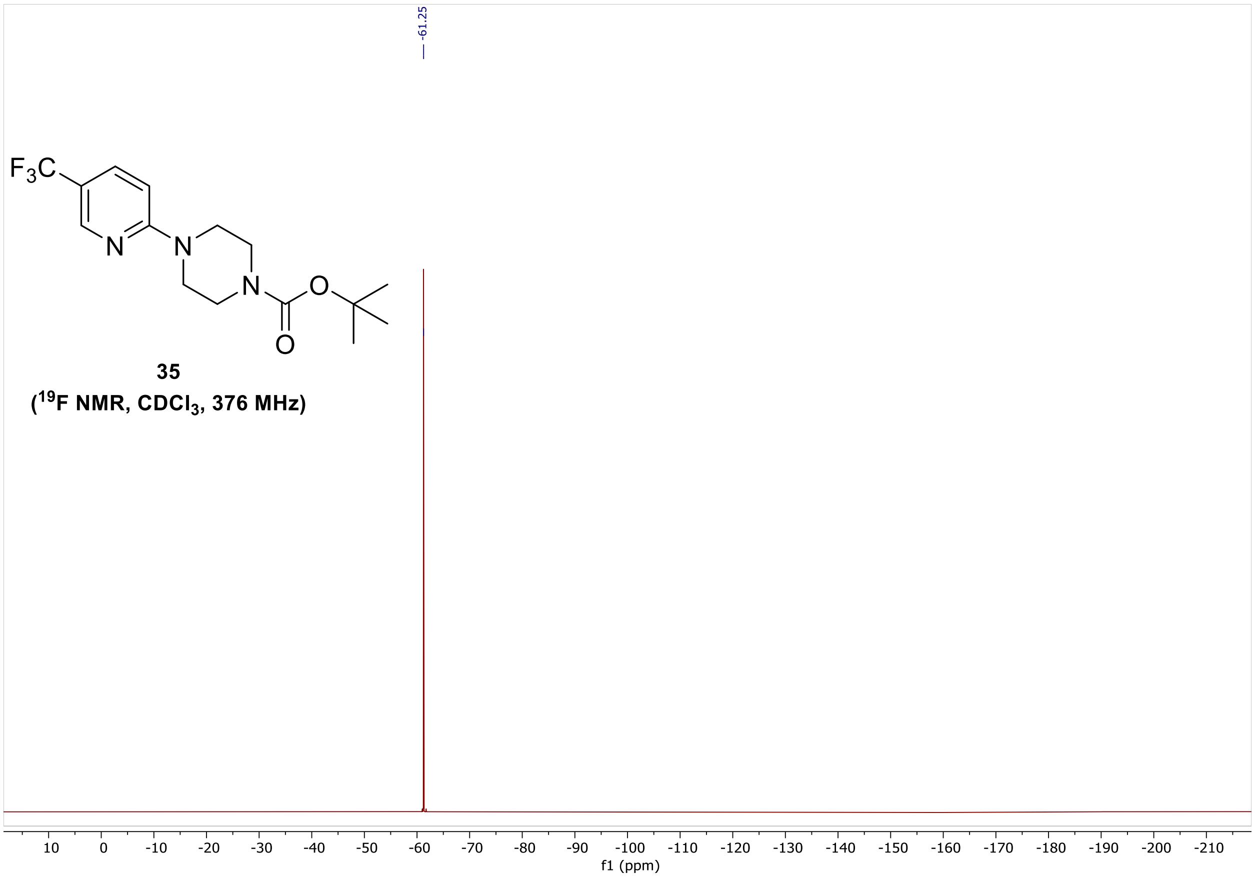

35

(^{19}F NMR, $CDCl_3$, 376 MHz)

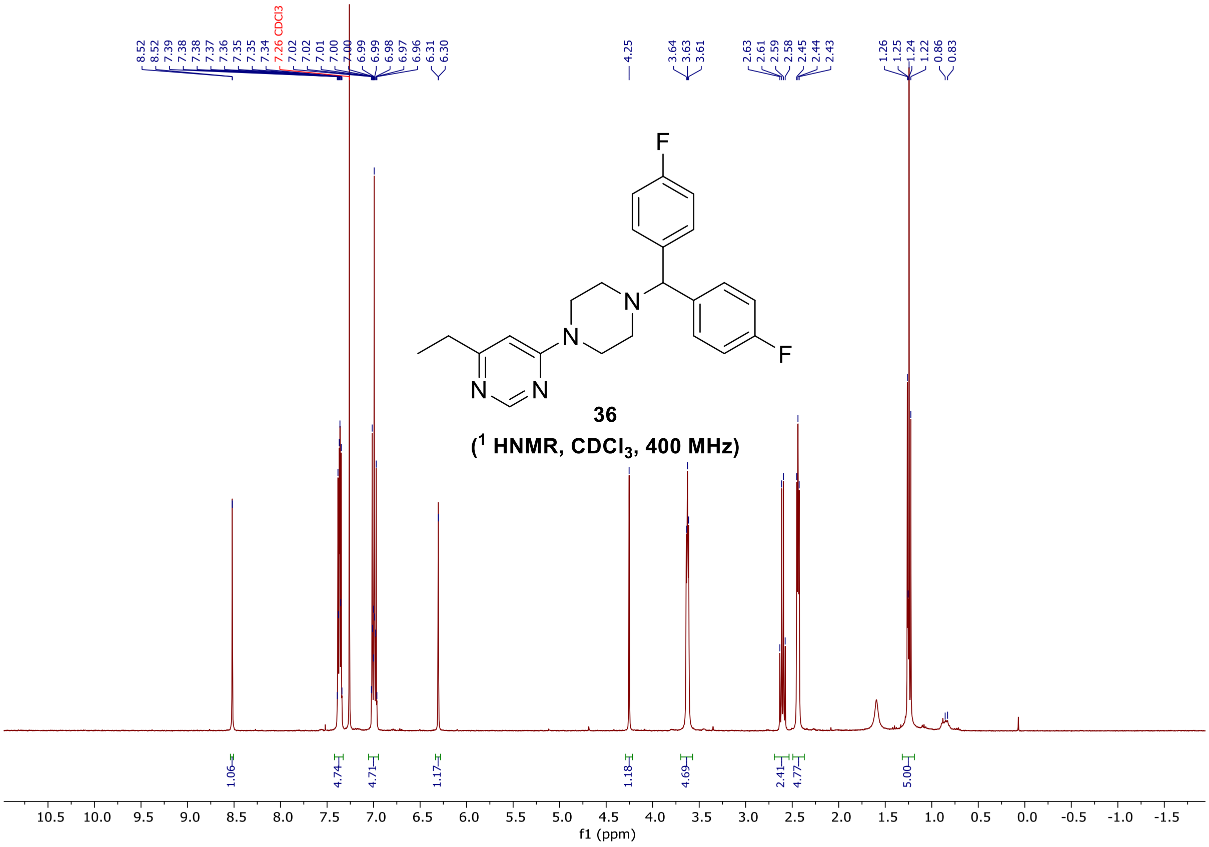
8.52
8.52
7.39
7.38
7.38
7.37
7.36
7.35
7.35
7.34
7.26 CDCl3
7.02
7.02
7.02
7.01
7.00
7.00
6.99
6.99
6.98
6.97
6.96
6.31
6.30
4.25
3.64
3.63
3.61
2.63
2.61
2.59
2.58
2.45
2.44
2.43
1.26
1.25
1.24
1.22
0.86
0.83
F
N
N
F
N
N
36
(1 HNMR, CDCl3, 400 MHz)
1.06
4.74
4.71
1.17
1.18
4.69
2.41
4.77
5.00
f1 (ppm)

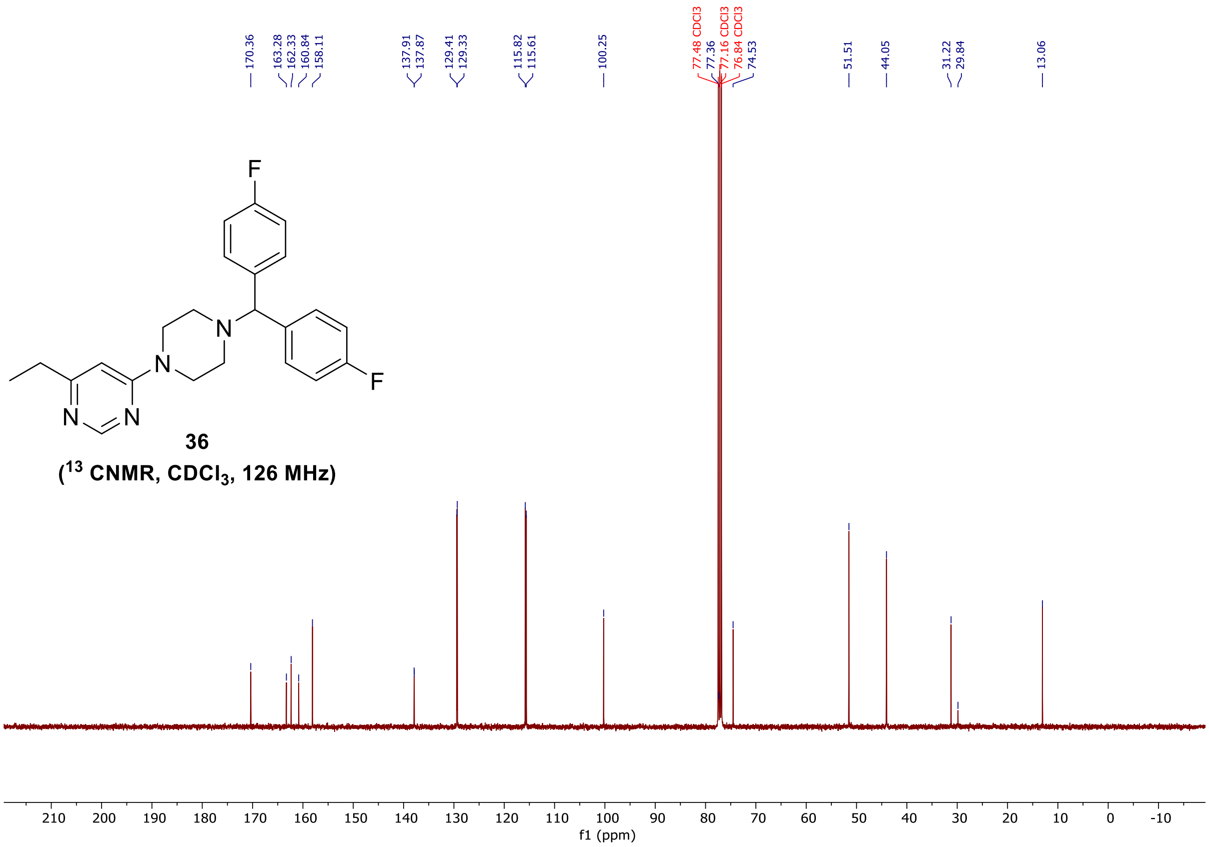
170.36
163.28
162.33
160.84
158.11
137.91
137.87
129.41
129.33
115.82
115.61
100.25
77.48 CDCl3
77.36
77.16 CDCl3
76.84 CDCl3
74.53
51.51
44.05
31.22
29.84
13.06
F
N
N
F
N
N
36
(13 CNMR, CDCl3, 126 MHz)
f1 (ppm)

36

(^{19} FNMR, $CDCl_3$, 376 MHz)

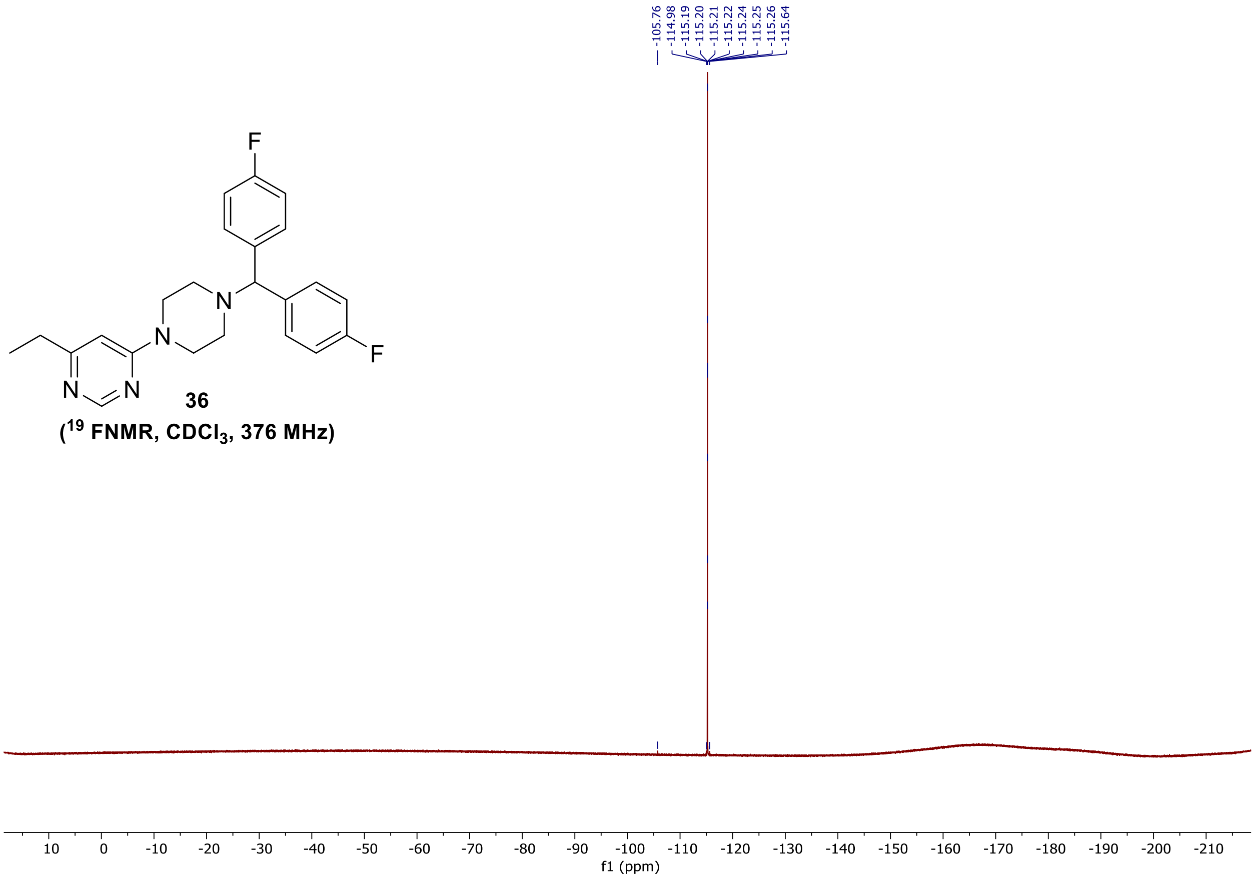

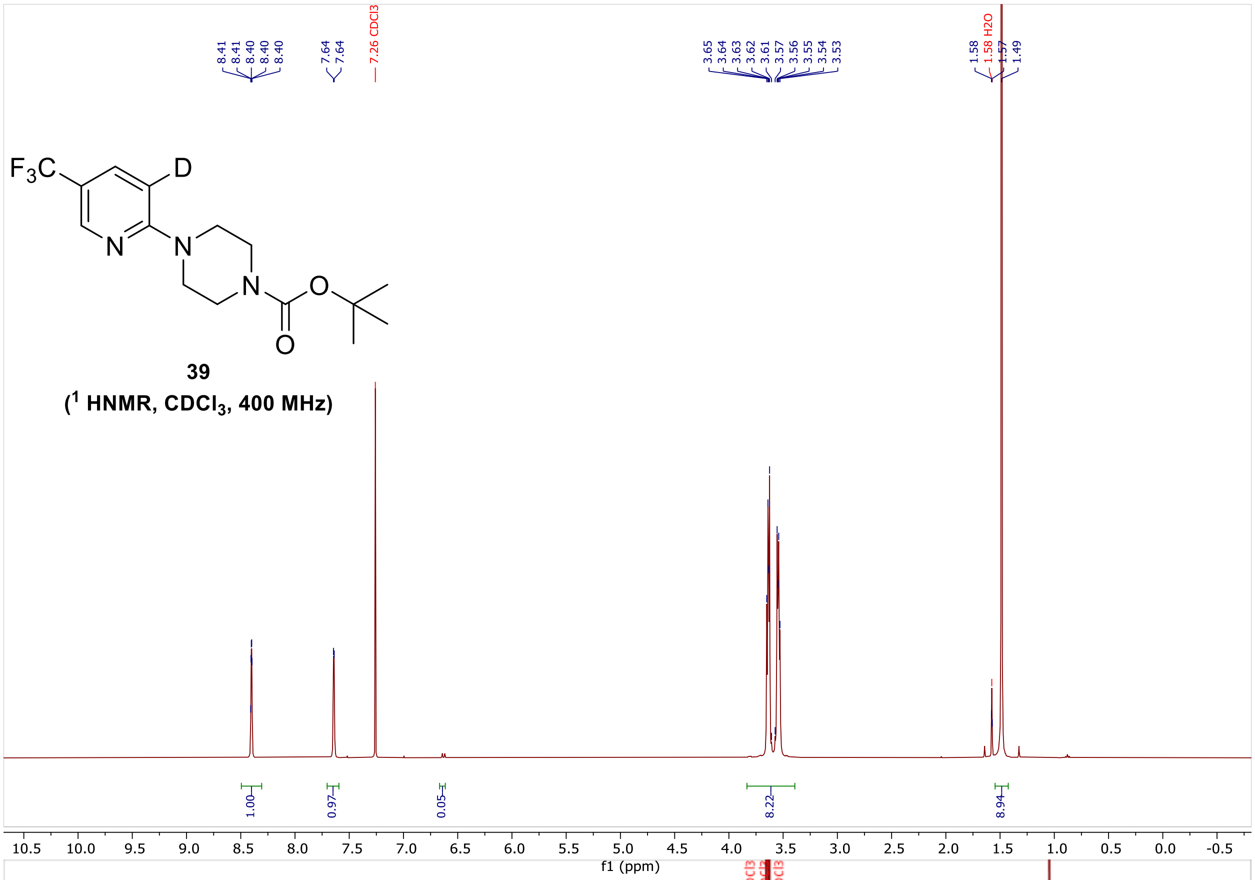
8.41
8.41
8.40
8.40
8.40
7.64
7.64
7.26 CDCl3
3.65
3.64
3.63
3.62
3.61
3.57
3.56
3.55
3.54
3.53
1.58
1.58 H2O
1.57
1.49
F3C
D
N
N
N
O
O
39
(1 HNMR, CDCl3, 400 MHz)
1.00
0.97
0.05
8.22
8.94
f1 (ppm)

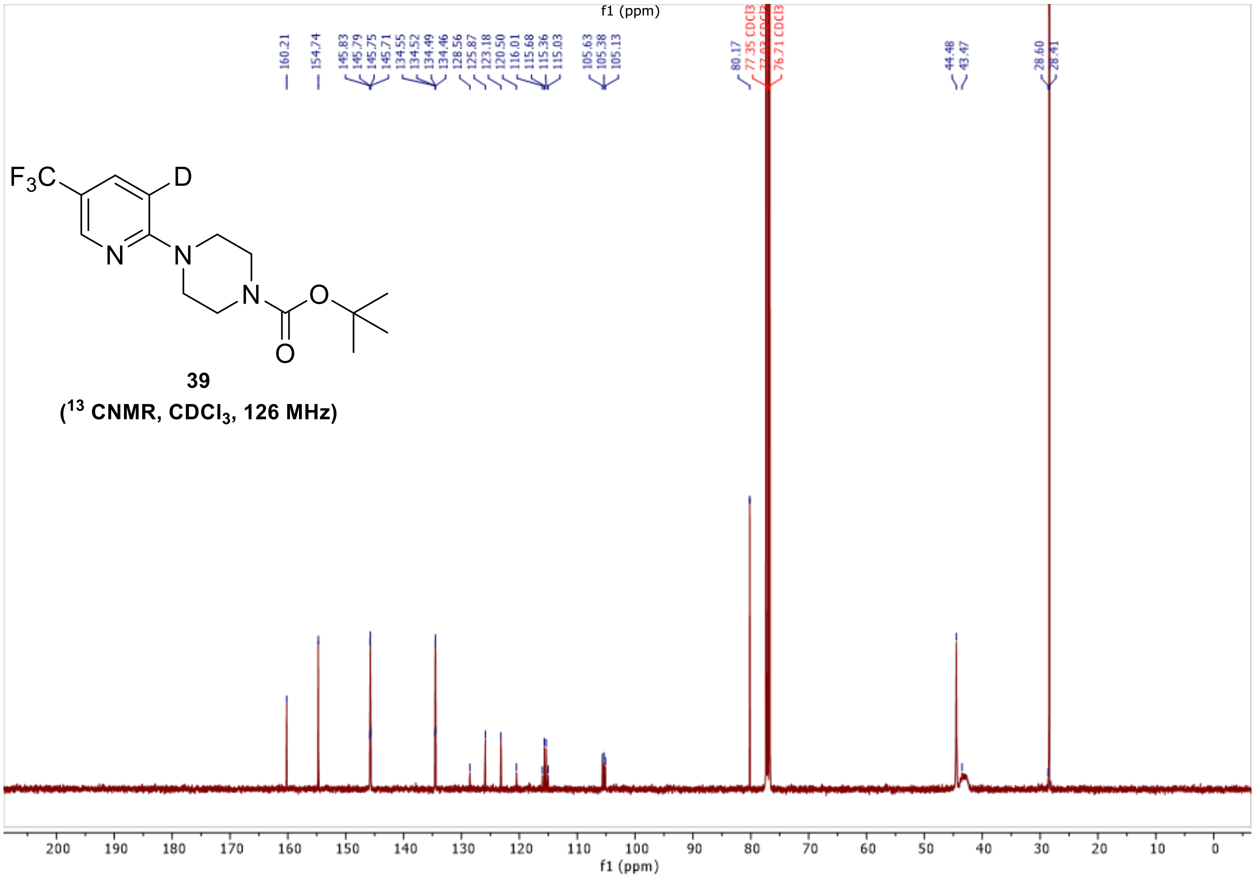
160.21
154.74
145.83
145.79
145.75
145.71
134.55
134.52
134.49
134.46
128.56
125.87
123.18
120.50
116.01
115.68
115.36
115.03
105.63
105.38
105.13
80.17
77.35 CDCl3
77.03 CDCl3
76.71 CDCl3
44.48
43.47
28.60
28.41
F3C
D
N
N
N
O
O
39
(13 CNMR, CDCl3, 126 MHz)
f1 (ppm)

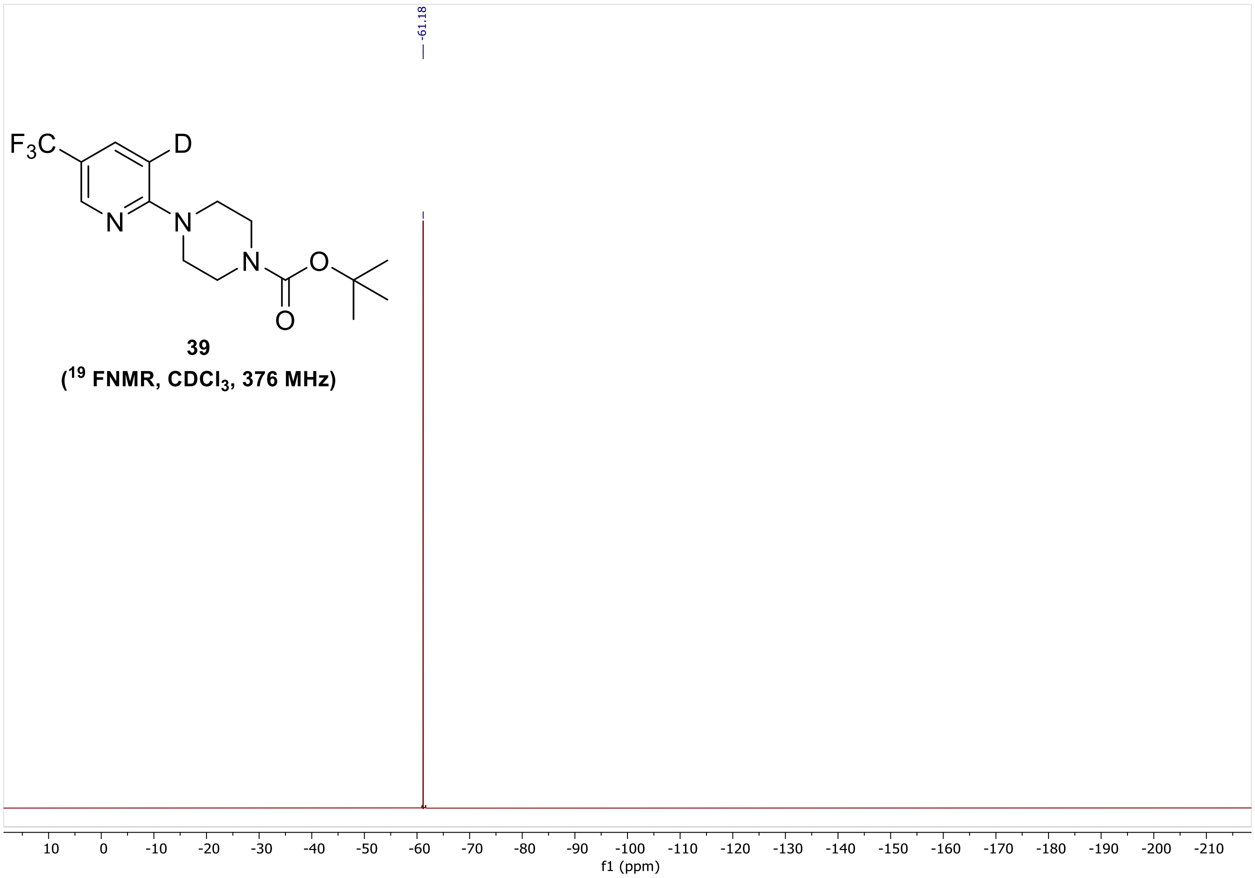
-61.18
F3C
D
N
N
N
O
O
39
(19 FNMR, CDCl3, 376 MHz)
10 0 -10 -20 -30 -40 -50 -60 -70 -80 -90 -100 -110 -120 -130 -140 -150 -160 -170 -180 -190 -200 -210
f1 (ppm)

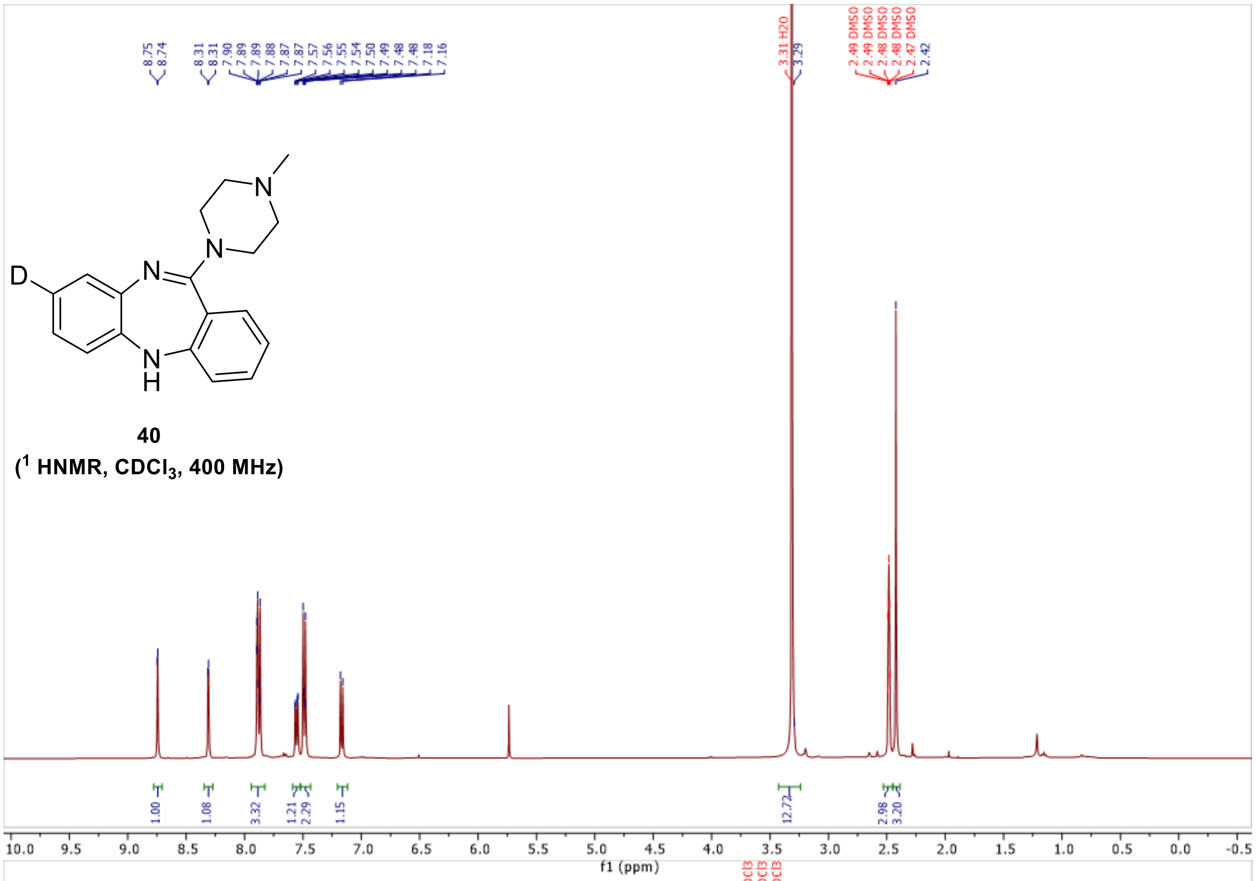

40
(^{1} HNMR, $CDCl_3$, 400 MHz)

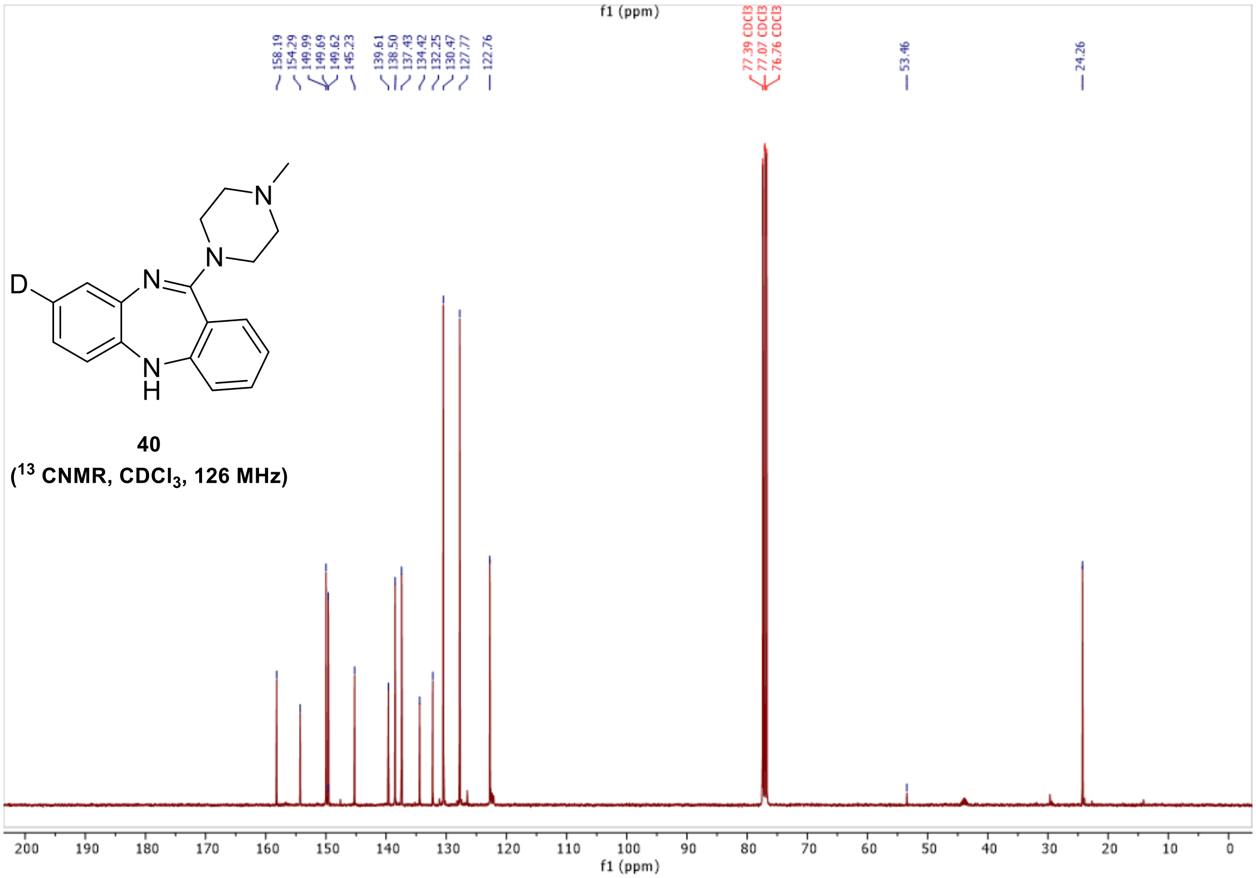

40
(^{13} CNMR, $CDCl_3$, 126 MHz)

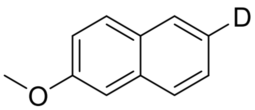

41

(^{1} HNMR, $CDCl_3$, 400 MHz)

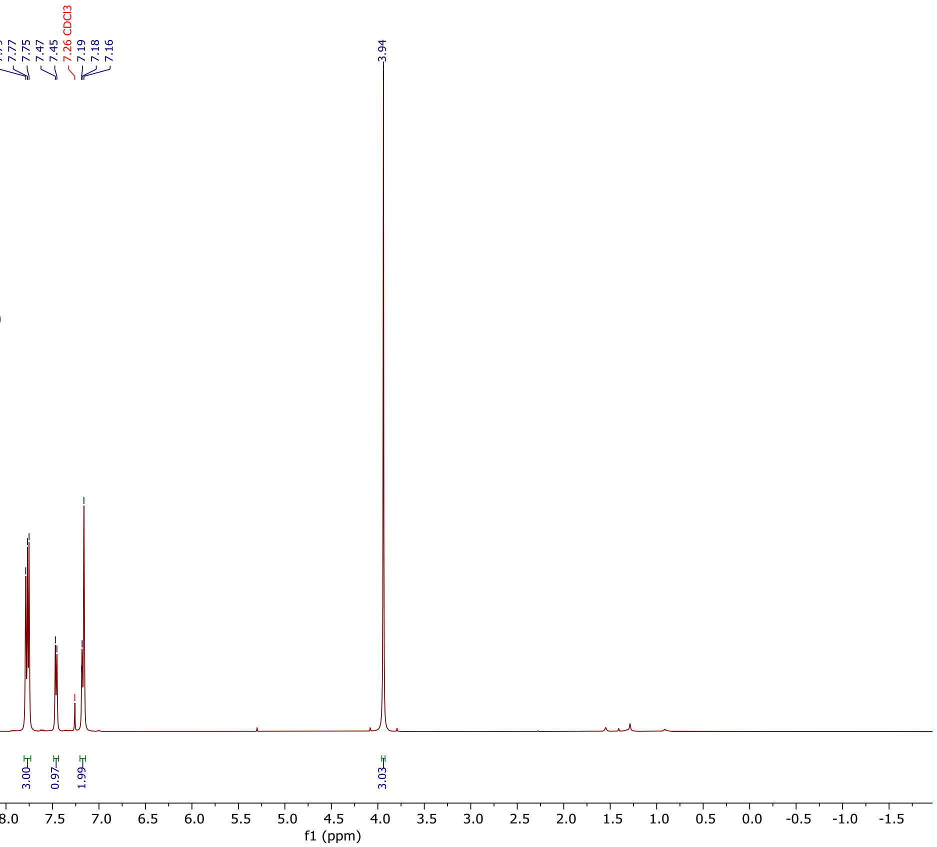

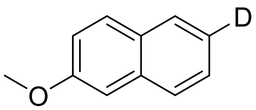

41

(^{13} CNMR, $CDCl_3$, 126 MHz)

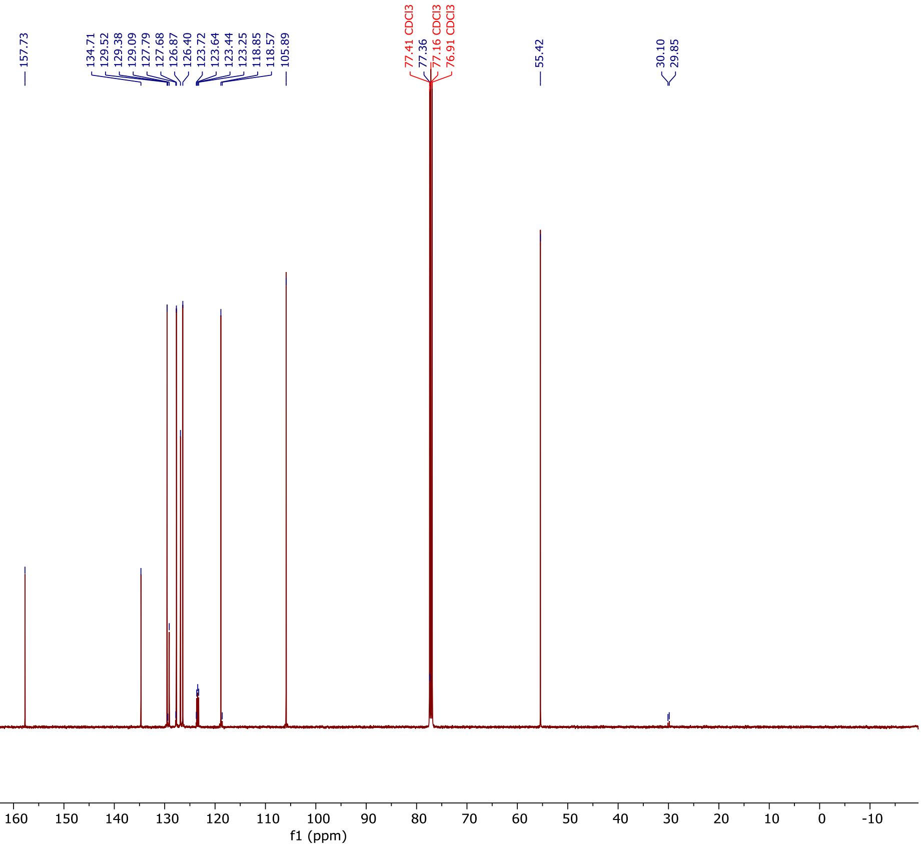

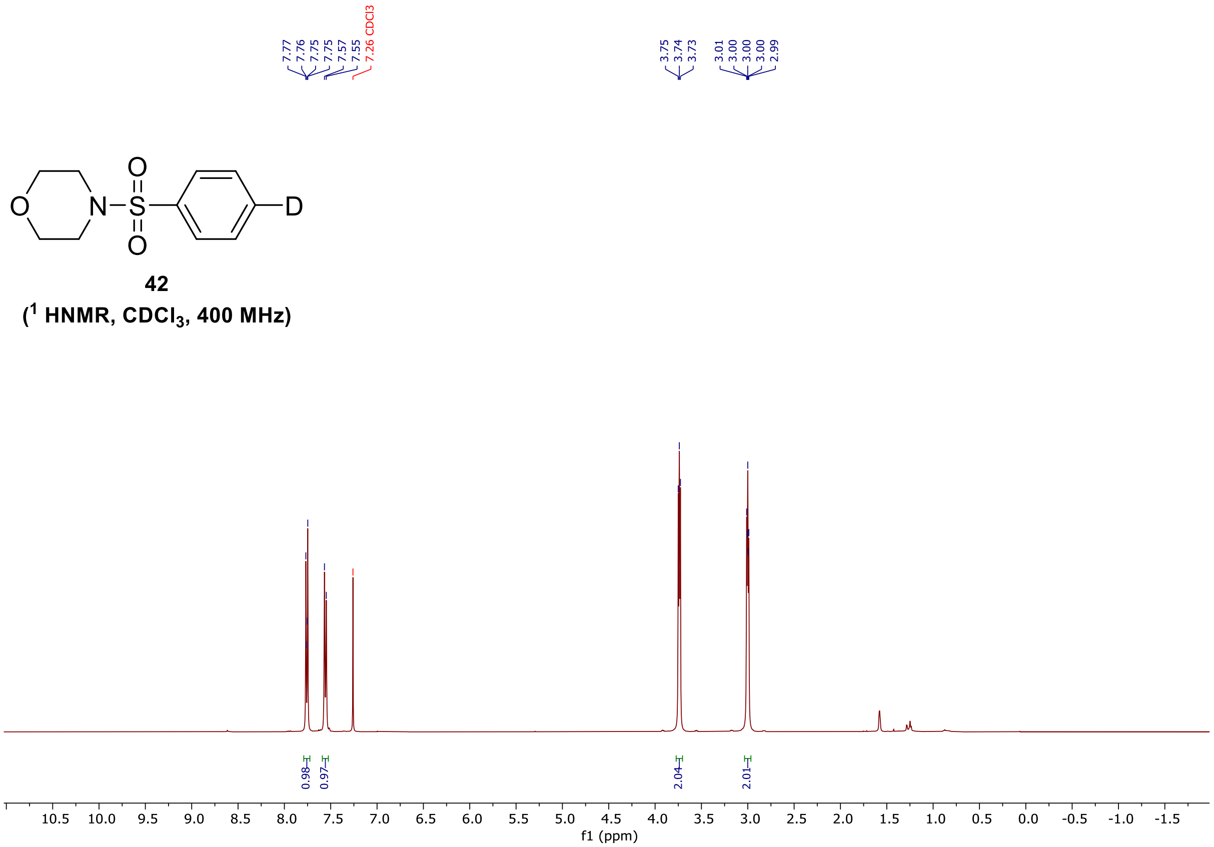

7.77
7.76
7.75
7.75
7.57
7.55
7.26 CDCl3
3.75
3.74
3.73
3.01
3.00
3.00
3.00
2.99
O
N
S
D
42
(1 HNMR, CDCl3, 400 MHz)
0.98
0.97
2.04
2.01
f1 (ppm)

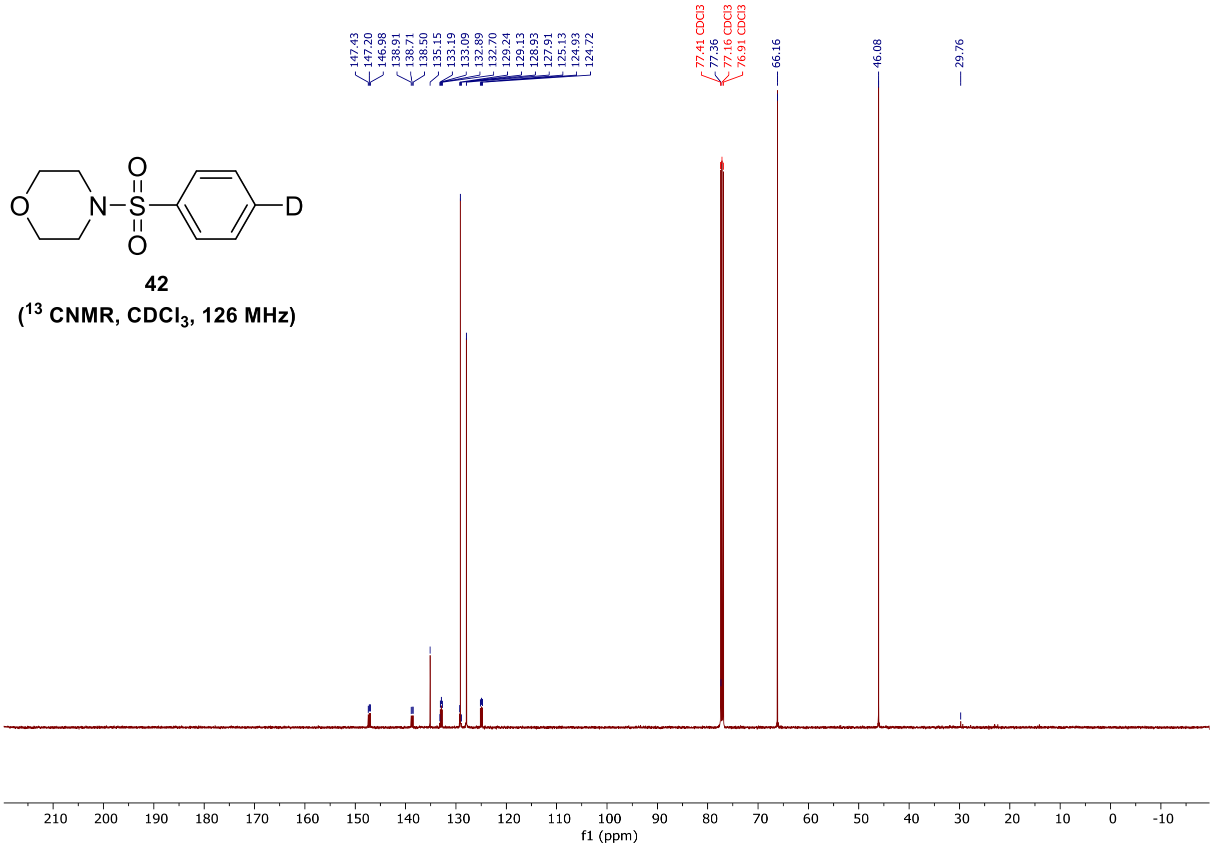

147.43
147.20
146.98
138.91
138.71
138.50
135.15
133.19
133.09
132.89
132.70
129.24
129.13
128.93
127.91
125.13
124.93
124.72
77.41 CDCl3
77.36
77.16 CDCl3
76.91 CDCl3
66.16
46.08
29.76
O
N
S
D
42
(13 CNMR, CDCl3, 126 MHz)
f1 (ppm)

43
(^{1} HNMR, $CDCl_3$, 400 MHz)

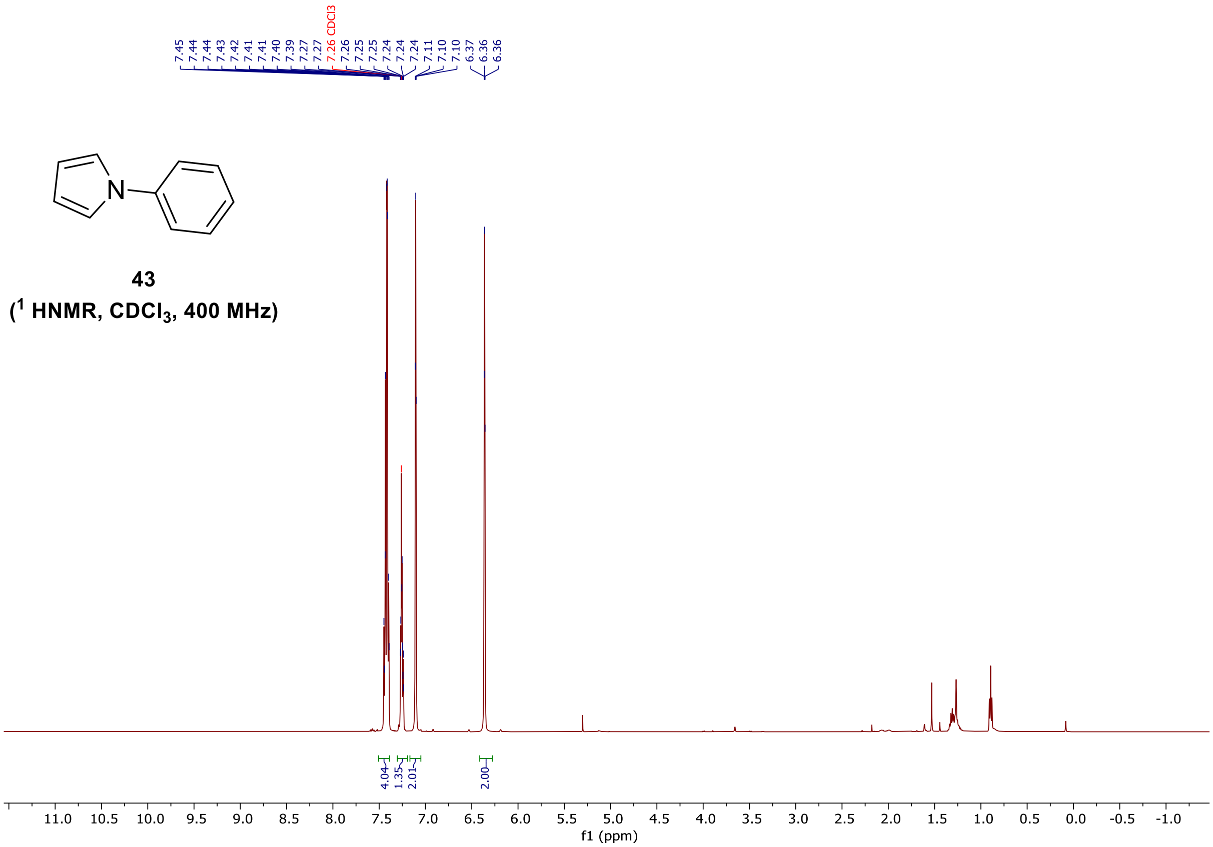

43
(^{13} CNMR, $CDCl_3$, 126 MHz)

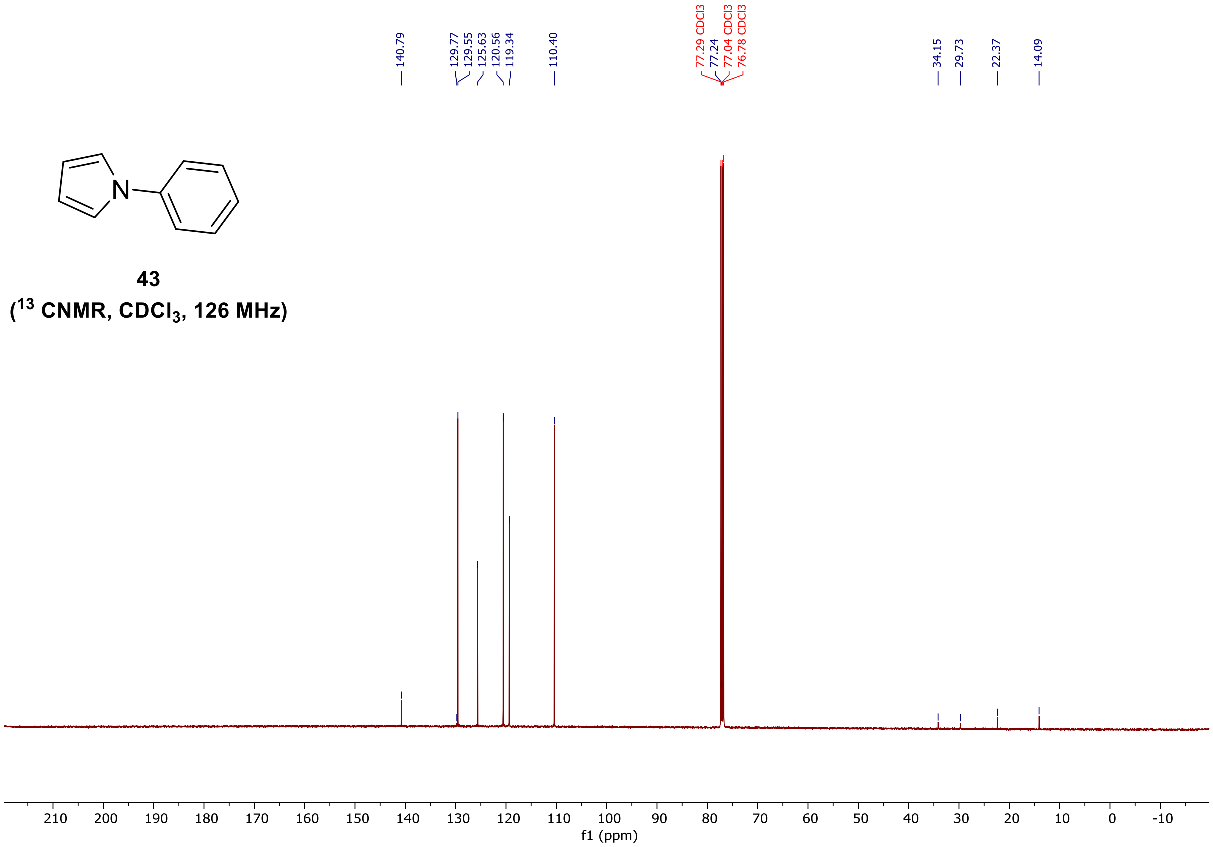

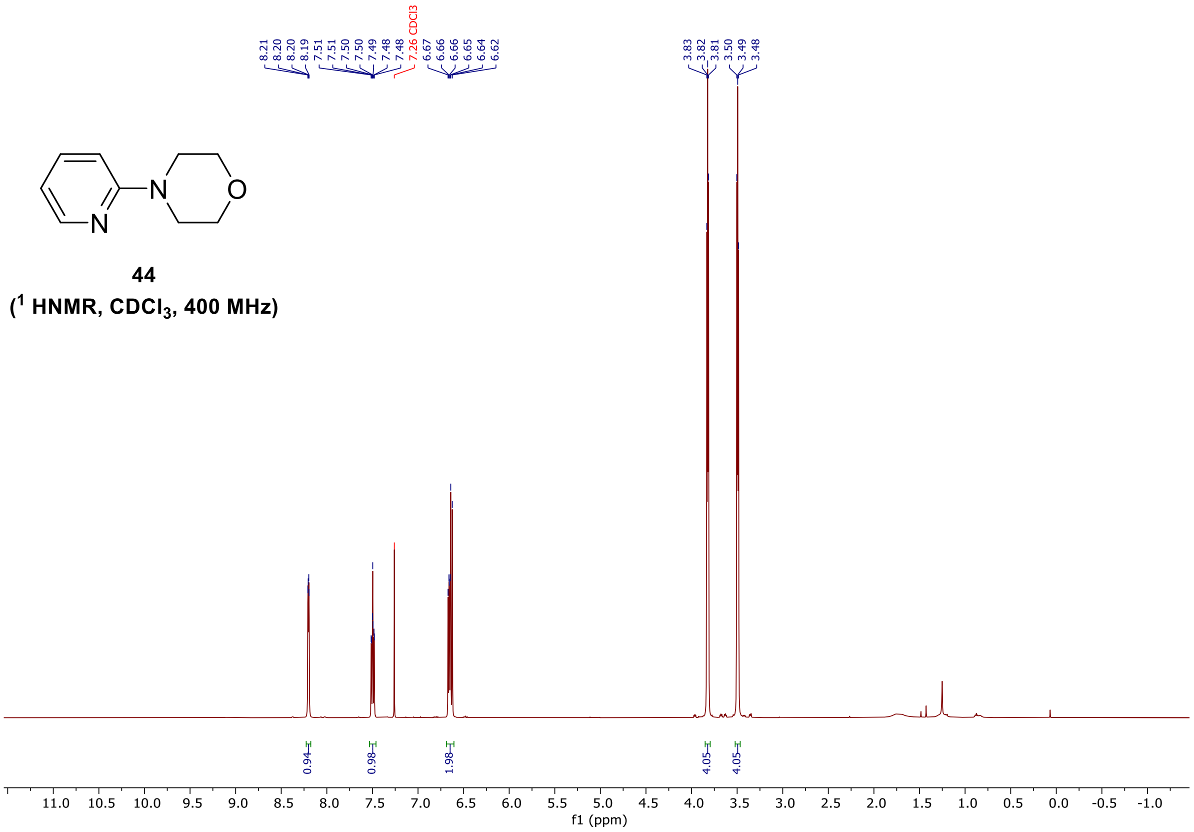

44
(^{1} HNMR, $CDCl_3$, 400 MHz)

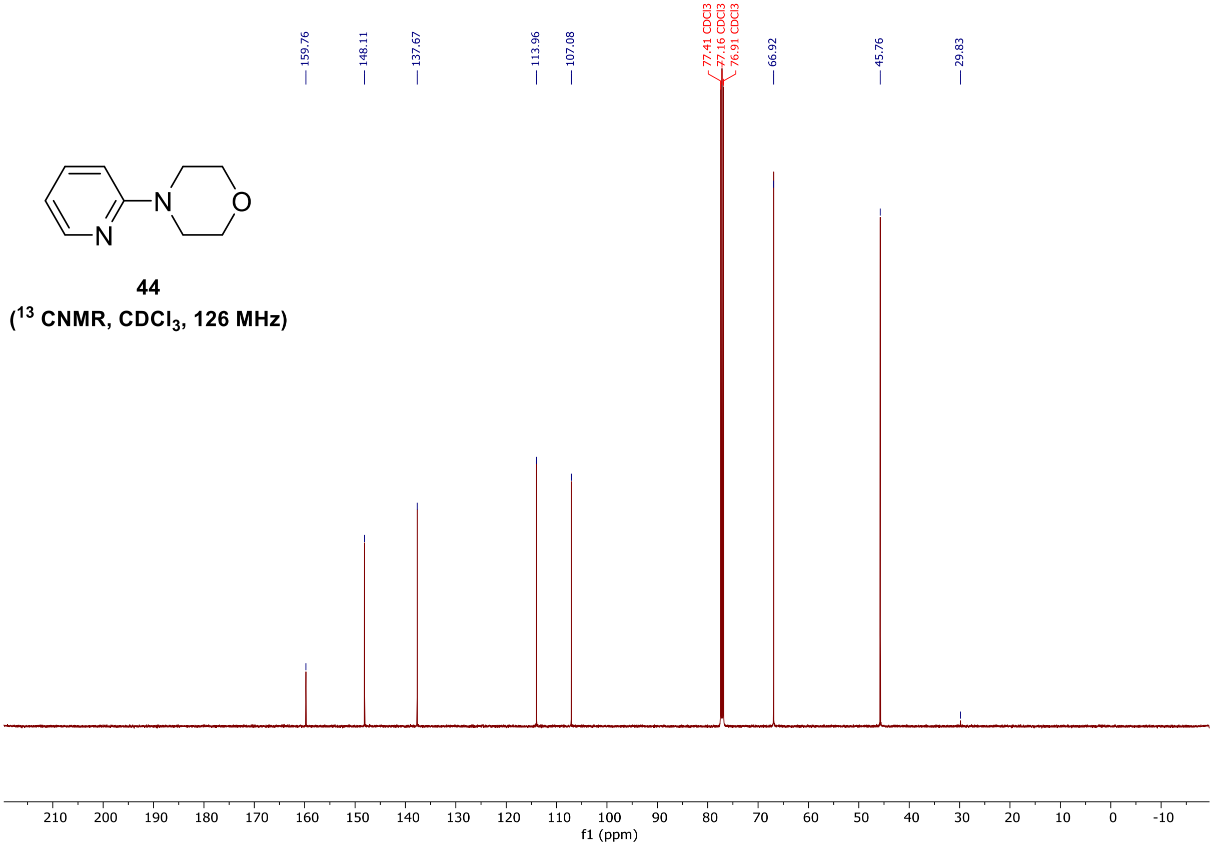

44
(^{13} CNMR, $CDCl_3$, 126 MHz)

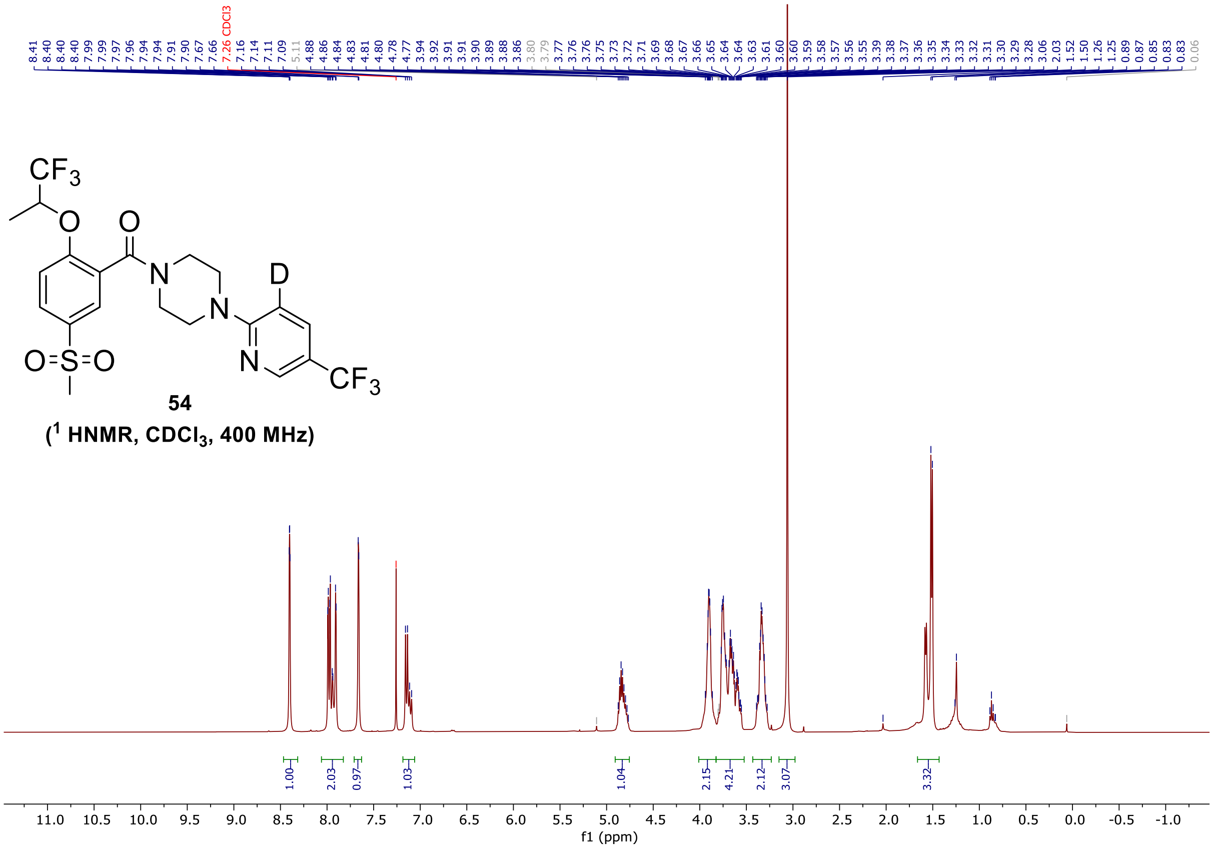

54

(^{1} HNMR, $CDCl_3$, 400 MHz)

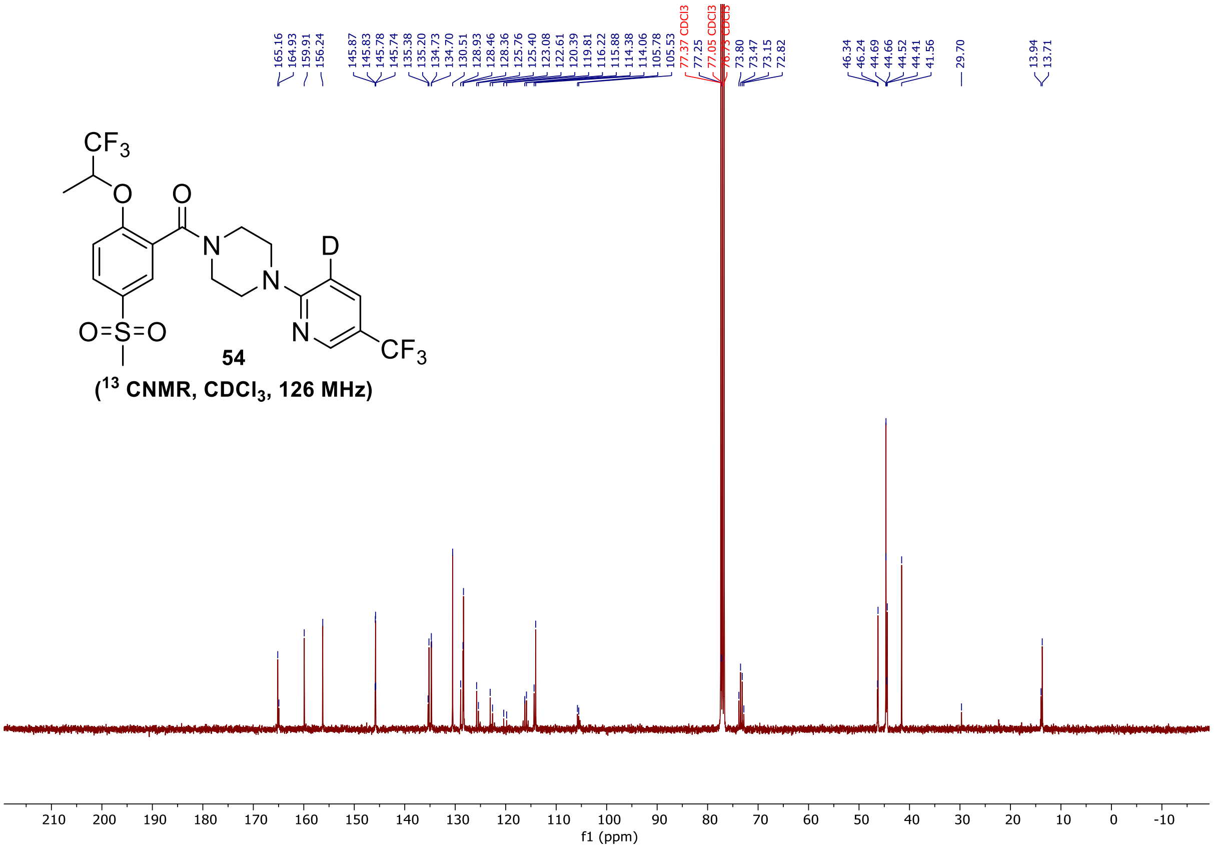

54

(^{13} CNMR, $CDCl_3$, 126 MHz)

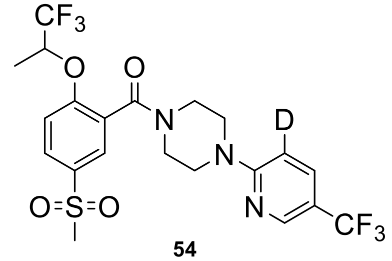

54

(^{19} FNMR, $CDCl_3$, 376 MHz)

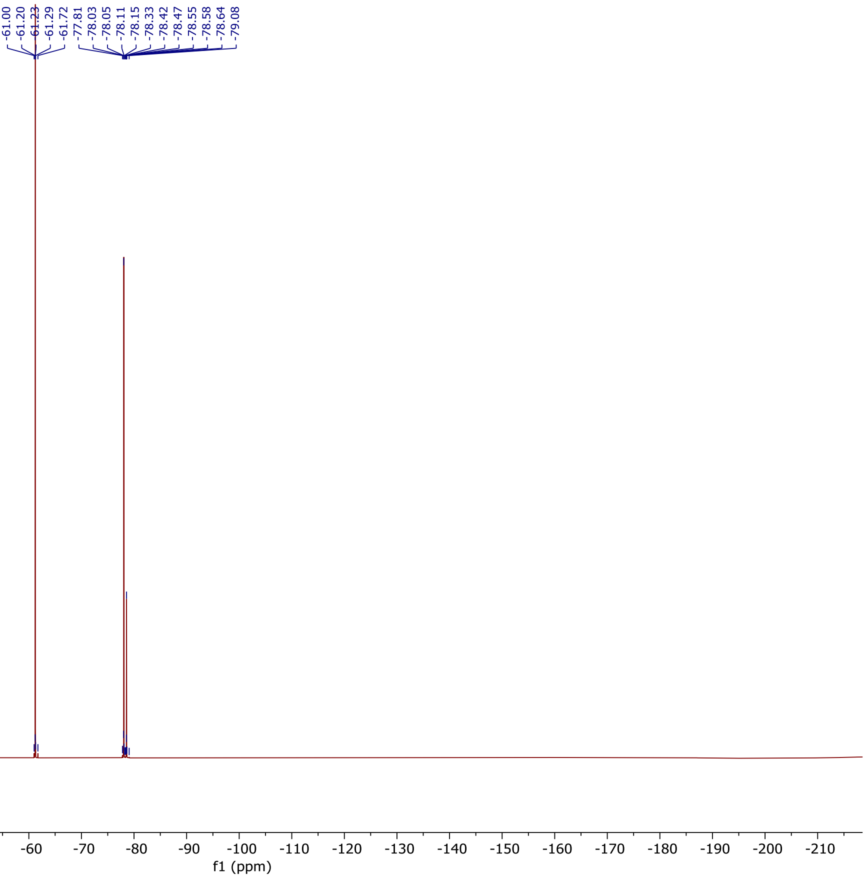

Supplement: Supplementary file 1 — Supporting Information [file CSSC-18-e202500043-s001.pdf]
